# Tandem Dearomatization/Enantioselective Allylic Alkylation of Pyridines

**DOI:** 10.1021/jacs.3c02470

**Published:** 2023-05-22

**Authors:** Steffen Greßies, Lars Süße, Tyler Casselman, Brian M. Stoltz

**Affiliations:** Warren and Katharine Schlinger Laboratory for Chemistry and Chemical Engineering, Division of Chemistry and Chemical Engineering, California Institute of Technology, Pasadena, California 91125, United States

## Abstract

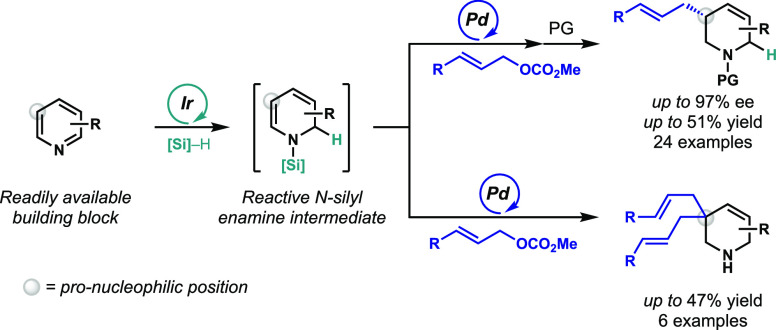

Herein, we report a multistep one-pot reaction of substituted pyridines leading to *N*-protected tetrahydropyridines with outstanding enantioselectivity (up to 97% ee). An iridium(I)-catalyzed dearomative 1,2-hydrosilylation of pyridines enables the use of *N*-silyl enamines as a new type of nucleophile in a subsequent palladium-catalyzed asymmetric allylic alkylation. This telescoped process overcomes the intrinsic nucleophilic selectivity of pyridines to synthesize enantioenriched, C-3-substituted tetrahydropyridine products that have been otherwise challenging to access.

Aromatic compounds are stable feedstock chemicals available with numerous substitution patterns that find application in various areas, such as material science,^1^ pharmaceuticals,^2^ and many others. However, the number of methods for converting these readily available aromatic chemicals into value-added, enantioenriched, saturated molecules with increased complexity remains sparse.^3^ Transition-metal-catalyzed asymmetric allylic alkylation (AAA) reactions are established and reliable transformations to form tertiary and quaternary stereocenters via numerous combinations of nucleophiles and electrophiles.^4,5^ Because of the versatility of this transformation, a vibrant area of research has emerged to convert readily available aromatic compounds into enantioenriched, saturated substrates via dearomative, transition-metal-catalyzed asymmetric allylic alkylation reactions.^6^ Trost et al. reported the first enantioselective palladium-catalyzed dearomative allylic alkylation at the C-3 position of indoles in 2006 (Scheme 1A).^7^ Since then, the reactivity of numerous electron-rich heteroaromatics^8a^ and electron-rich benzene derivates (phenols, anilines) have been explored.^5^ Recently in 2018, You et al. reported the first intramolecular, iridium-catalyzed dearomative allylation of simple benzenes, which further expanded this field (Scheme 1B).^9^ In 2014, You et al. published the intramolecular allylic *N*-alkylation of pyridines (Scheme 1C).^10^ However, the expansion of this field to electron-poor heterocycles, such as pyridines, remains elusive. In 2007, Hartwig et al. reported the allylic alkylation of terminal enamines under iridium catalysis, which leads to enantioenriched β-ketones after hydrolysis (Scheme 1D).^11^ The utilization of a similar enamine intermediate derived from the dearomative reduction of pyridines should allow for the asymmetric allylic alkylation of pyridines at the C-3 position, which would invert the inherent nucleophilic selectivity of the aromatic substrate. Among the most common motifs in pharmaceuticals are (partially) saturated *N*-heterocycles,^12^ while their synthesis remains challenging, especially in an enantioselective fashion. While the electrophilicity of the C-2 and C-4 positions of pyridines has been utilized with many nucleophiles, the functionalization of the C-3 position is less explored.^13^ Friedel–Crafts-like alkylations favor the 3-position, although they are mainly limited to pyridines bearing electron-donating substituents.

**Scheme 1:**
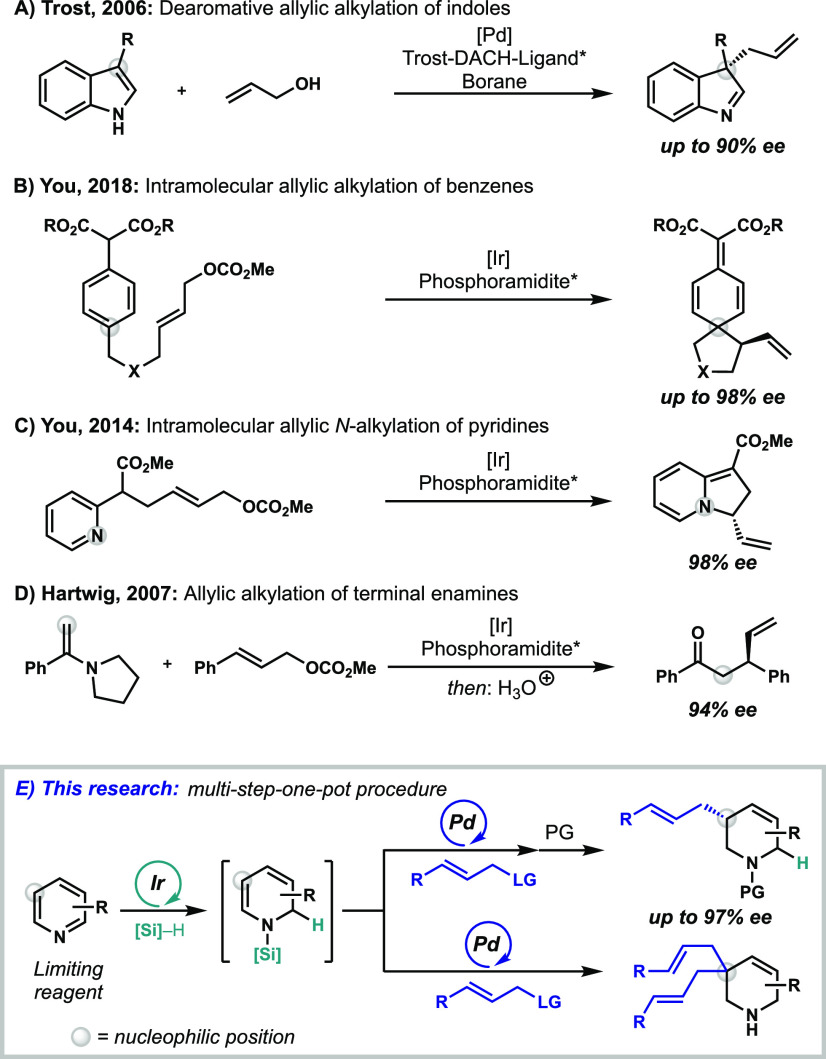
Transition-Metal-Catalyzed Allylic Alkylations

However, dearomative hydrosilylation reactions of pyridines and related heterocycles have been developed with several heterogeneous catalysts,^14^ transition metal catalysts (Ti, Ru, Ir, Zn),^15^ and main group metals (Ca)^16^ and organic catalysts (boranes).^17^ While the resulting *N*-silylated enamines are highly unstable and cannot be purified, their derivatization can lead to stable dihydropyridines. We hypothesized that *N*-silyl dihydropyridines, obtained by a dearomative 1,2-hydrosilylation, can act as enamine C-nucleophiles in an allylic alkylation reaction (Scheme 1E).

Here, we report a protocol that enables the dearomative C3-allylic alkylation of pyridines in high enantioselectivity. To the best of our knowledge, this is the first report on the utilization of pyridines as C-nucleophile precursors in enantioselective allylic alkylation reactions. For the initial reduction, we selected the iridium(I)-catalyzed hydrosilylation of aza-heteroaromatics that was reported by Chang et al. in 2016 (Scheme 2).^15g^

**Scheme 2:**
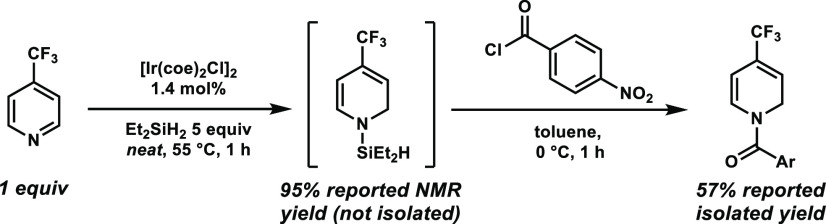
Ir-Catalyzed Hydrosilylation of Pyridines by Chang et al.^15g^

We tested our hypothesis by treating 3-fluoropyridine **1a** with 1 mol % of [Ir(coe)_2_Cl]_2_ and 5 equiv of diethyl silane at 50 °C for 3 h. In accordance with Chang et al.’s results, we found almost quantitative conversion to the *N*-silyl dihydropyridine. This mixture was then added to a solution of Pd_2_(dba)_3_ (2.5 mol %), PPh_3_ (15 mol %), and cinnamyl methyl carbonate **2a** (1.5 equiv) in CH_2_Cl_2_ (0.2 M) at 40 °C for 16 h. The desired dearomative allylation product **3a** was observed in trace quantities (Table 1, entry 1). However, two side products were also detected. Namely, the rearomatized 3-alkylated pyridine was observed as a major product (**4a**, 11%) together with a bisalkylated side product (**5a**, <5%). Initial screening of monodentate phosphines revealed that neither more electron-rich or electron-deficient phosphines nor sterically more demanding monodentate phosphines could improve the outcome of this reaction. Having established a proof of concept for this reaction pathway, we turned our attention to developing an asymmetric variant. To control the stereochemistry at the C3-postion, several privileged chiral monodentate and bidentate phosphorus-containing ligands, such as PHOX, phosphoramidite, BINAP, DTBM-SegPhos, and DIOP were investigated [for details, see the Supporting Information (SI)]. Unfortunately, less than 5% of the desired product was observed in all reactions, but enantioinduction could be observed with up to 30% and 48% ee for **L1** and Feringa’s phosphoramidite **L2**, respectively (Table 1, entries 2 and 3). Employing Trost-DACH ligand **L3** (7 mol %) resulted in significantly increased conversion (38% of **3a**) and excellent enantioselectivity (96% ee, entry 4). Modifications on the ligand did not result in an improved performance in the transformation. Pd(OAc)_2_ was found to be the optimal Pd source, while other precursors, such as Pd_2_dba_3_·CHCl_3_, gave similar results (Table 1, entries 4 and 5). CH_2_Cl_2_ was already the ideal solvent of the reaction, while lower conversion with slightly higher enantioselectivity was observed in benzene (Table 1, entry 6), THF, and 1,4-dioxane (for details, see the SI). Finally, an enhanced conversion for the desired product **3a** was achieved though the addition of catalytic amounts of sodium fluoride (Table 1, entry 7). Further exploration of additional bases or hydride sources for the allylic alkylation step had no beneficial effect. Interestingly, with a reduced amount of hydrosilane, the alkylated pyridine **4b** (Table 1, entry 8) was obtained as a major product (28% of **4a** could be isolated; for details, see the SI). The ratio between the synergistic catalysts was crucial for the desired reactivity. Altering the amount of iridium dimer to 0.5 or 2.5 mol % resulted in a significantly decreased amount of **3a** (Table 1, entries 9 and 10). Both catalysts are required for the reaction; otherwise, no product was observed in the control experiments without the Ir or Pd catalyst. In the reaction setting, the iridium-catalyzed hydrosilylation was performed under argon. After the indicated time, this reaction mixture was added to a prestirred mixture of palladium catalyst, ligand, additive, and carbonate in CH_2_Cl_2_ under argon and heated to 40 °C.

**Table 1:**
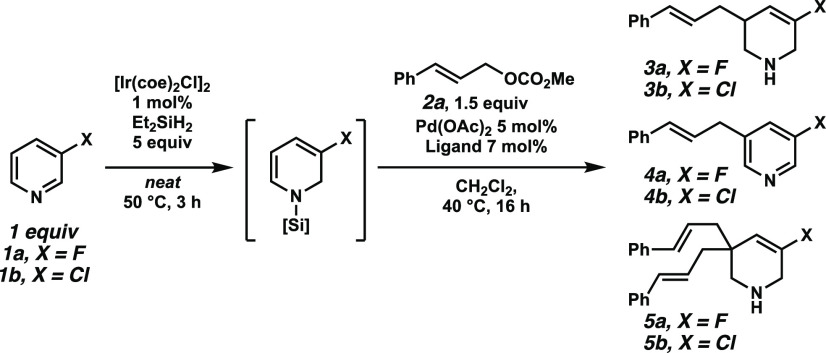
Selected Optimization Reactions^a^

#	deviation from standard reaction conditions	**L**	**3** (% ee)	**4**	**5**
1	Pd_2_(dba)_3_ 2.5 mol %	PPh_3_	2 (n.d.)	11	<5
2	Pd_2_(dba)_3_ 2.5 mol %	**L1**	<5 (30)	<5	<5
3	Pd_2_(dba)_3_ 2.5 mol %	**L2**	<5 (48)		<5
4		**L3**	38 (96)	8	5
5	Pd_2_dba_3_·CHCl_3_ 2.5 mol %	**L3**	38 (96)	9	10
6	Pd_2_dba_3_ 2.5 mol % in PhH	**L3**	18 (98)	11	<5
7	NaF 10 mol %	**L3**	49 (96)	8	6
8^b^	Et_2_SiH_2_ 2.5 equiv, NaF 10 mol %	**L3**	29 (n.d.)	38	29
9^b^	[Ir] 2.5 mol %, NaF 10 mol %	**L3**	5 (n.d.)	<5	<5
10^b^	[Ir] 0.5 mol %, NaF 10 mol %	**L3**	14 (n.d.)	34	45

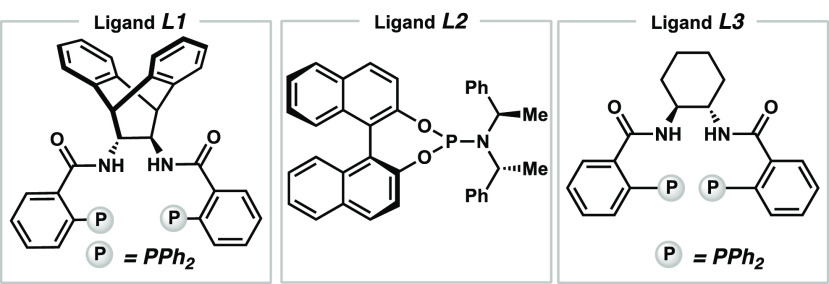

a Reaction conditions: first step = **1a** (0.2 mmol), Et_2_SiH_2_ (5 equiv), [Ir(coe)_2_Cl]_2_ (1 mol %), 50 °C, 3 h; second step = **2a** (1.5 equiv), Pd(OAc)_2_ (5 mol %), **L6** (7 mol %), CH_2_Cl_2_ (0.2 M), 40 °C, 16 h. LC/UV–vis yields were determined via calibration curve obtained using isolated products. **L** = ligand; n.d. = not determined.

b 3-Chloropyridine (**1b**) was used as substrate.

With the optimized conditions in hand, we investigated the substrate scope (Scheme 3). For simplified handling during purification, the product amines **3** were protected by *N*-acylation in an additional step in the same pot. Various electron-poor pyridines reacted smoothly to the desired products **6a**–**d** and **6f**–**k** in moderate yields between 21 and 54% but, in most cases, with excellent enantioselectivity of above 90% ee. Substituents were generally tolerated in the 3- and 4-position of the pyridine. Substituents on the 2- or 6-position of the pyridine did not yield any desired product likely because of the steric hindrance for the first hydrosilylation step. Motifs such as 4-aryl or 4-heteroaryl pyridines (**6e**, **6h**, **6l**, and **6m**) also gave the desired products in moderate yields and excellent enantioselectivity. More electron-rich pyridines were challenging in this reaction mainly because of the significantly slower iridium-catalyzed hydrosilylation reaction, as previously observed.^18^

**Scheme 3:**
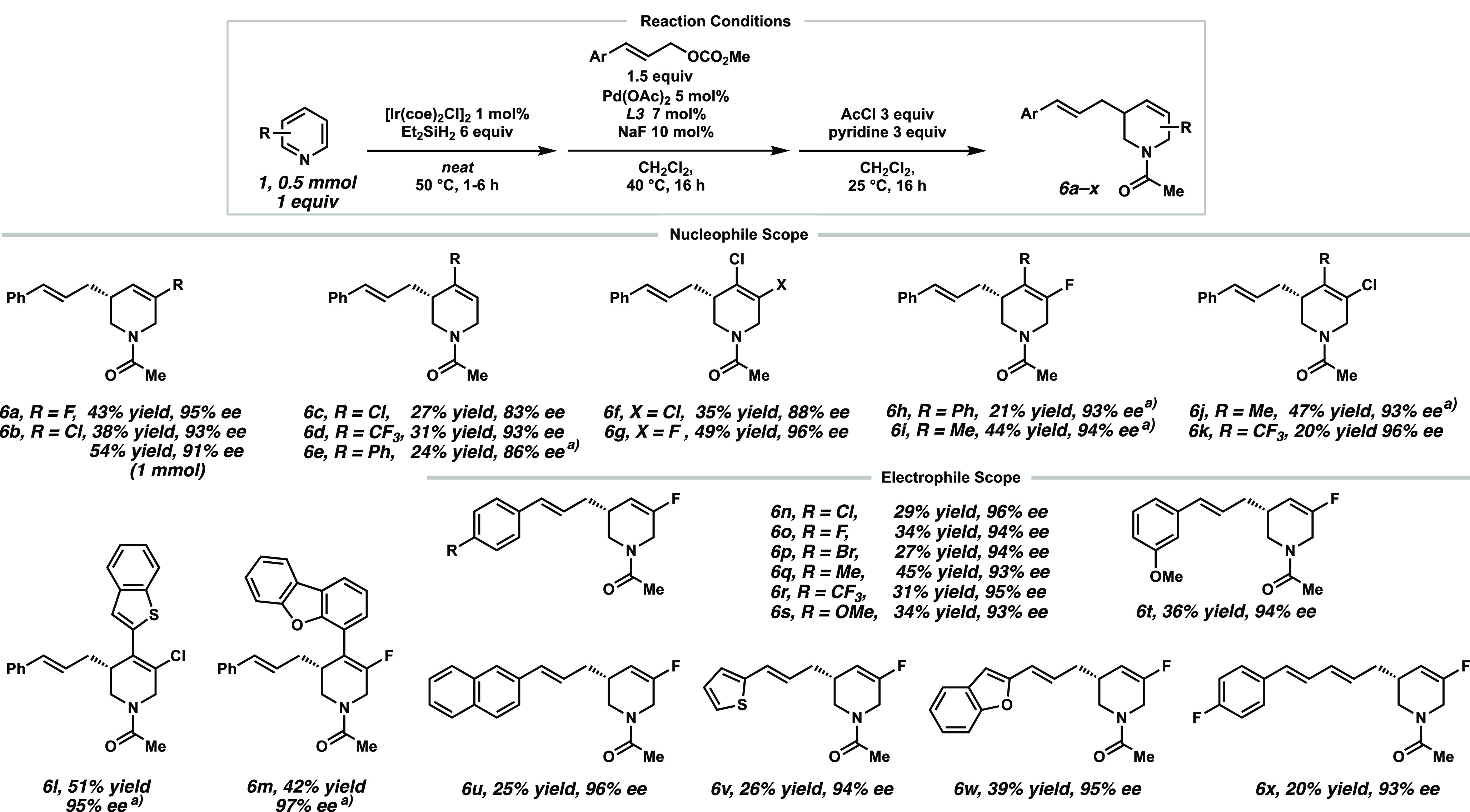
Substrate Scope for the Enantioselective Allylic Alkylation Towards Chiral Products **6**(19)^,^ The iridium catalyst (1.5 mol %) was used with increased reaction times for the first step and by stepwise addition for more electron-rich pyridines; for details see the SI. Standard reaction conditions: first step = **1** (0.5 mmol), Et_2_SiH_2_ (6.0 equiv), [Ir(coe)_2_Cl]_2_ (1 mol %), 50 °C, 1–6 h; second step = **2** (1.5 equiv) Pd(OAc)_2_ (5 mol %), **L3** (7 mol %), CH_2_Cl_2_ [0.2 M], 40 °C, 16 h; third step = AcCl (3.0 equiv), pyridine (3.0 equiv), CH_2_Cl_2_, 25 °C, 16 h.

Residual pyridine did not inhibit the Pd-catalyzed alkylation. A control experiment even revealed some influence on the reaction selectivity, which is under further mechanistic investigation (see the SI for details).^20^ The electrophile scope; however, tolerates electron-donating as well as electron-withdrawing substituents on the arene, thereby giving the corresponding products in moderate yields and excellent enantioselectivity (**6n**–**s**). *meta*-Substitution on the arene also delivered the desired product (**6t**), while *ortho*-OCF_3_-substituted cinnamyl carbonate showed no conversion in this transformation (not shown). Fortunately, heteroaromatic carbonates could be applied in this reaction, as shown by products **6v** and **6w**. While simple alkyl-substituted allylic carbonates did not afford any product (see Scheme S7 for details), we found that a conjugated diene precursor delivered the diene product **6x** in low yield but excellent enantioselectivity. The products **6** bearing a halogen handle also allowed further derivatization via cross-coupling chemistry, as demonstrated for **6b** in Scheme 4. Typical Stille conditions with PhSnBu_3_ delivered the C–C cross-coupled product **8** in 82% yield, while a Ni-catalyzed Kumada reaction provided the *N*-deprotected C–C cross-coupled product **9** with similar yield (Scheme 4), which shows the synthetic utility of these motifs.

**Scheme 4:**
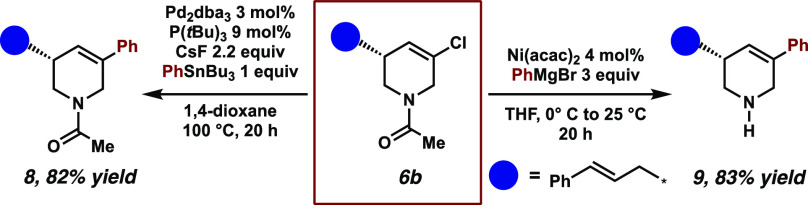
Further Derivatization of **6b** via Stille and Kumada Reactions

From a synthetic perspective, the free NH products, such as **9** or **3a**, are also of high interest. However, the direct purification by column chromatography after the second step, that is, the Pd-catalyzed allylic alkylation, proved to be challenging because of the polarity, as well as the complex reaction mixture. *N*-Boc protection after the allylic alkylation and a subsequent purification delivered an inseparable mixture of the *N*-Boc-protected desired product **3a**, as well as the bisalkylated *N*-Boc side product **5a** (Scheme 5, right). An acidic deprotection with TFA allowed the isolation of the pure NH product **3a** in 22% yield over all four steps. Alternatively, the *N*-acetylated compounds **6b** could be treated with PhMgBr to give the free NH product **3b** in 88% yield (47% yield over four steps, Scheme 5, left).

**Scheme 5:**
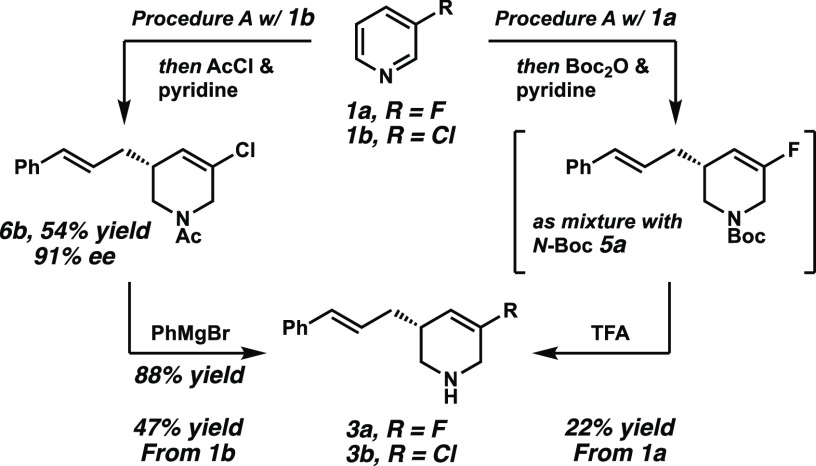
Isolation of the Free NH Product **3a,b**

On the basis of our results and understanding, the proposed reaction sequence (see Scheme 6) starts with the iridium-catalyzed 1,2-hydrosilylation of the pyridine substrate **A**, which leads to an *N*-silyl enamine **B**. The palladium-catalyzed allylic alkylation leads to a cyclic imine intermediate **C** (or as an *N*-silyl iminium ion). There are several plausible pathways from this key intermediate **C**. The excess of silane in the reaction mixture and the, therefore, overall reductive conditions in the presence of two transition metals can lead to a reduction of the imine, thereby leading to the enantioenriched products **3**. Another pathway, which could explain the formation of the bisalkylated products **5**, is the tautomerization of the imine **C** to the enamine **D**, which results in a nucleophile that participates in an additional alkylation event. Reduction of the product imine **E** leads to the bisalkylated derivatives **5**. The observed rearomatized product **4** can potentially form at different stages during the proposed sequence, but would require an oxidant (e.g., air or a hydride transfer to π-allyl complexes).

**Scheme 6:**
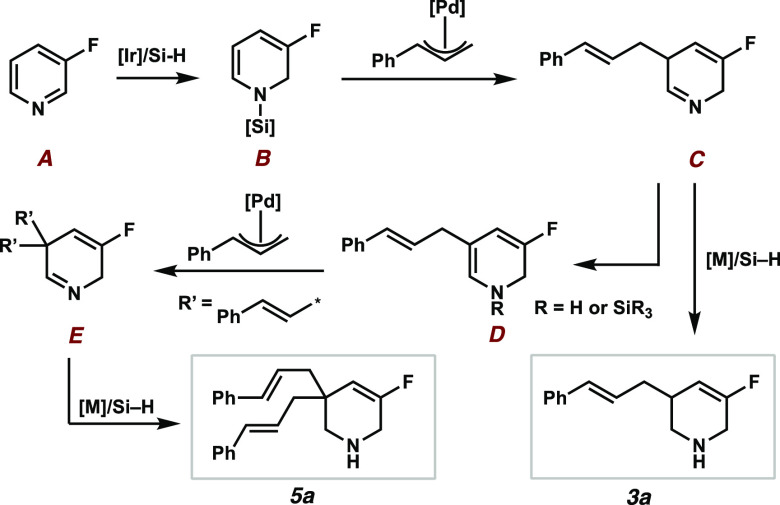
Proposed Reaction Sequence

During the investigation of the reaction scope, we observed that the bisalkylated side products **5** occur in different ratios on the basis of the substrate. Since those motifs can be of synthetic interest, as well, the transformation was optimized toward these scaffolds using 3-chloropyridine **1b** as the standard substrate.

The screening of alkylating reagents with leaving groups of variable basicity did not increase the overall yields of either **3b** or **5b** (for details, see the SI). It was found that catalytic amounts of benzoic acid (20 mol %) significantly shift the selectivity toward the bisalkylated product **5b** (Scheme 7).^21^ The electronic properties of the alkylating reagent seem to have less of an influence on the outcome of the reaction (**5c**–**f**).

**Scheme 7:**
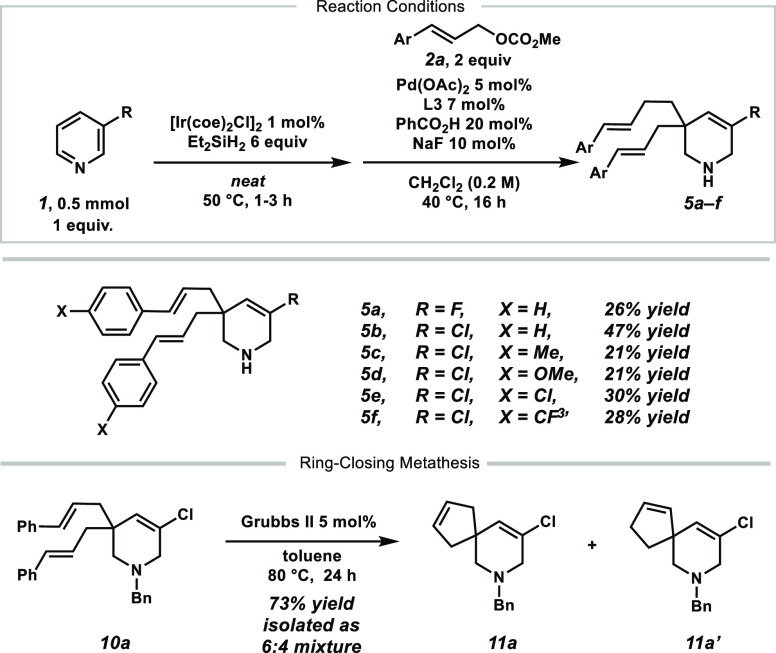
Bisalkylated Products **5** and RCM

The bisalkylated products **5** enable a potential ring-closing metathesis (RCM) to yield spiro compounds. Therefore, the *N*-benzyl product **10a** was treated with typical RCM conditions under ruthenium catalysis. The spiro compound **11a** was successfully formed in good yield of 73%, although as a 6:4 mixture of isomers that is formed by isomerization of the disubstituted olefin (see Scheme 7).

In conclusion, we have developed the first intermolecular asymmetric allylic alkylation (AAA) using electron-poor arenes, namely pyridines, as C-nucleophile precursors. A stepwise one-pot sequence allows rapid access to interesting molecular scaffolds in excellent enantioselectivities, although in moderate yields. The products are valuable building blocks for further exploration. The chlorine-substituted tetrahydropyridines are especially shown to be of particular use for the synthetic community as complex building blocks.

## Supporting Information Available

Experimental procedures and spectroscopic data (^1^H NMR, ^13^C NMR, IR, and HRMS) (PDF)

## ABBREVIATIONS

AAA: asymmetric allylic alkylation.
RCM: ring-closing metathesis
