# Supplementary material for: Tandem Dearomatization/Enantioselective Allylic Alkylation of Pyridines
Source: J Am Chem Soc. 2023 May 22;145(22):11907–13. doi: 10.1021/jacs.3c02470 (PMC10251512; doi:10.1021/jacs.3c02470)
Supplement: Supplementary file 1 — ja3c02470_si_001.pdf [file ja3c02470_si_001.pdf]

Supporting Information for

Tandem Dearomatization/Enantioselective Allylic Alkylation of Pyridines

Steffen Greßies, Lars Süße, Tyler Casselman, and Brian M. Stoltz*

Warren and Katharine Schlinger Laboratory of Chemistry and Chemical Engineering, Division of Chemistry and Chemical Engineering, California Institute of Technology, Pasadena, California 91125, United States

stoltz@caltech.edu

Table of Contents:

Materials and Methods S2
Supplementary Schemes S3
Synthesis of Allylating Reagents: Experimental Procedures S3
Synthesis of Ligands: Experimental Procedures S3
Synthesis of Pyridine Reagents: Experimental Procedures and Spectroscopic Data S5
Optimization for the Dearomatize Asymmetric Allylic Alkylation S7
Mixed Pyridine Control Experiment S11
List of Unsuccessful Substrates S12
Synthesis of Bisalkylated Products: Experimental Procedures and Spectroscopic Data S13
Synthesis of Monoalkylated Products: Experimental Procedures and Spectroscopic Data S17
Synthesis of Rearomatized Products: Experimental Procedures and Spectroscopic Data S54
Product Derivitizations S57
X-Ray Crystallographic Data for Mannich Product **7a** (V20222) S62
NMR and IR Spectra of New Compounds S83

Materials and Methods

Unless otherwise stated, reactions were performed in oven-dried glassware under an argon or nitrogen atmosphere using dry, deoxygenated solvents. Solvents were dried by passage through an activated alumina column under argon. Reaction temperatures stated in the manuscript or this document are reported as temperature of the surrounding metal heating blocks. TLC was performed using E. Merck silica gel 60 F254 precoated glass plates (0.25 mm) and visualized by UV fluorescence quenching or $KMnO_4$ staining. Silicycle Silia*Flash*® P60 Academic Silica gel (particle size 40–63 µm) was used for flash chromatography. ^{1}H NMR spectra were recorded on Varian Inova 500 MHz and Bruker 400 MHz spectrometers and are reported relative to residual $CHCl_3$ (δ 7.26 ppm) or MeOH (δ 4.87 ppm). ^{13}C NMR spectra were recorded on a Varian Inova 500 MHz spectrometer (125 MHz) and Bruker 400 MHz spectrometers (101 MHz) and are reported relative to $CHCl_3$ (δ 77.16 ppm) or MeOH (δ 49.00 ppm). ^{19}F NMR and ^{31}P NMR Spectra are reported without reference. Data for ^{1}H NMR are reported as follows: chemical shift (δ ppm) (multiplicity, coupling constant (Hz), integration). Multiplicities are reported as follows: s = singlet, d = doublet, t = triplet, q = quartet, p = pentet, sept = septuplet, m = multiplet, br s = broad singlet. Data for ^{13}C NMR are reported in terms of chemical shifts (δ ppm) plus (multiplicity, coupling constant (Hz)) in appropriate cases. IR spectra were obtained by use of a Perkin Elmer Spectrum BXII spectrometer or Nicolet 6700 FTIR spectrometer using thin films deposited on NaCl plates and reported in frequency of absorption (cm^{-1}). Optical rotations were measured with a Jasco P-2000 polarimeter operating on the sodium D-line (589 nm), using a 100 mm path-length cell. Analytical SFC was performed with a Mettler SFC supercritical CO_2 analytical chromatography system utilizing Chiralpak (AD-H, AS-H or IC) or Chiralcel (OD-H, OJ-H, or OB-H) columns (4.6 mm x 25 cm) obtained from Daicel Chemical Industries, Ltd. High resolution mass spectra (HRMS) were obtained from Agilent 6200 Series TOF with an Agilent G1978A Multimode source in electrospray ionization (ESI+), atmospheric pressure chemical ionization (APCI+), or mixed ionization mode (MM: ESI-APCI+). Absolute configuration of **7a** was determined by X-ray diffraction, and all other products are assigned by analogy. Reagents were purchased from commercial sources and used as received unless otherwise stated. 3-Fluoropyridine, 3-chloropyridine, 4-chloropyridine and 4-(trifluoromethyl)-pyridine were distilled over CaH_2 under nitrogen atmosphere prior to use. Diethylsilane was used as received. No significant differences in reactivity and yield were

observed from different commercial sources (SigmaAldrich, Gelest or Alfa Aesar). The used Iridium ($[Ir(coe)_2Cl]_2$) catalyst was purchased from Strem Chemicals, Inc, transferred to the glovebox and used as received. The used Palladium catalyst ($Pd(OAc)_2$) was purchased from SigmaAldrich, transferred to the glovebox and used as received.

Supplementary Schemes: Substrate/reagent and ligand preparation

Scheme S1: General synthetic route to carbonate reagents (**2**)

All carbonate reagents used in this study have previously been described in the literature and were prepared accordingly. The general synthetic route can be seen in Scheme 1. The analytical data agrees with the literature.

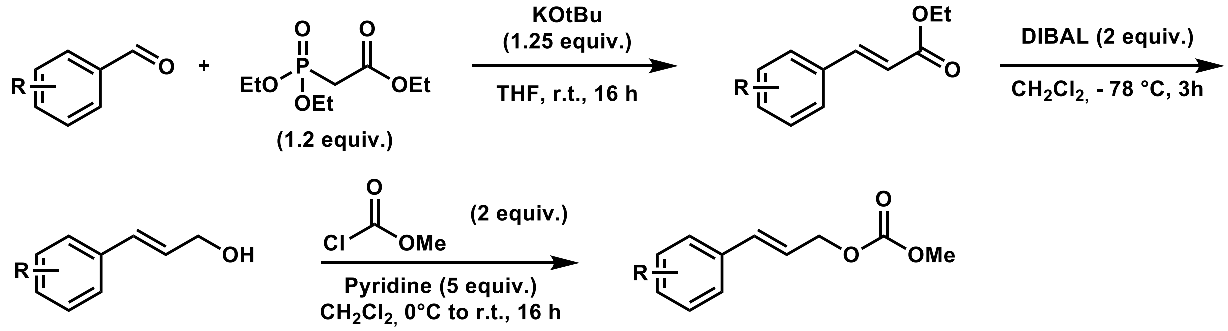

Scheme S2: General synthetic route to ligand **L3**

Ligands **L1-L10** were prepared either according to literature procedure, are commercially available or as described below. The detailed procedure for the optimized ligand **L3** that is used in this study can be seen below in Scheme 2.

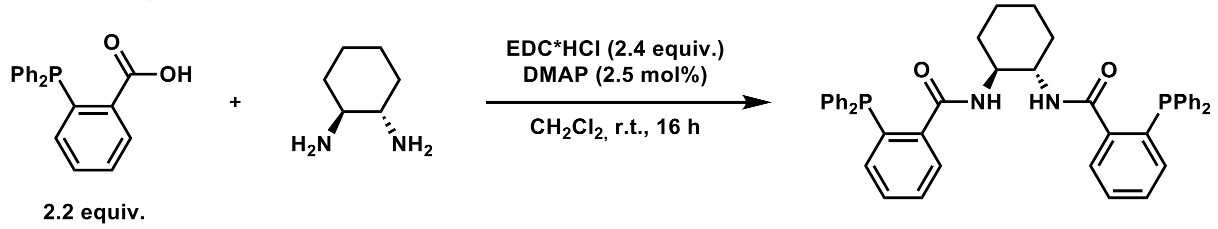

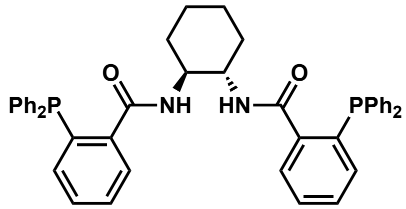

L3
Chemical Formula: $C_{44}H_{40}N_2O_2P_2$
Exact Mass: 690.2565
Molecular Weight: 690.7635

Ligand **L3** (DACH-Trost-Ligand) was prepared following the reaction in Scheme 2 according to a literature procedure (*European Journal of Organic Chemistry* **2007**, *7*, 1145). Therefore, commercial 2-(diphenylphosphaneyl)benzoic acid (15.48 g, 50.6 mmol, 2.2 equiv.) was dissolved in CH_2Cl_2 (120 mL). DMAP (70 mg, 0.575 mmol, 2.5 mol%) was added, followed by EDC*HCl (10.58 g, 55.2 mmol, 2.4 equiv.). The mixture was stirred at room temperature for 5 min. (1S,2S)-Cyclohexane-1,2-diamine (2.63 g, 23 mmol, 1.0 equiv.) in CH_2Cl_2 (30 mL) was added and the resulting mixture was stirred at room temperature for 16

hours. The reaction was quenched with saturated NH_4Cl solution (~100 mL). The phases were separated, and the organic phase was dried over magnesium sulfate and filtered. The crude reaction mixture was submitted to flash column chromatography over silica gel using hexane/EtOAc = 7/3 as the eluent to yield a white solid (11.5 g). The white solids were redissolved in boiling MeCN (~350 mL) and slowly cooled to room temperature over night to yield white crystals (9.5 g, 13.75 mmol, 60% yield) (Figure S1).

^{1}H NMR (300 MHz, $CDCl_3$): δ 7.60 – 7.55 (m, 2H), 7.34 – 7.16 (m, 24H), 6.94 – 6.87 (m, 2H), 6.32 (d, *J* = 7.2 Hz, 2H), 3.85 – 3.71 (m, 2H), 1.90 – 1.80 (m, 2H), 1.70 – 1.59 (m, 2H), 1.28 – 1.15 (m, 2H), 1.05 – 0.90 (m, 2H);

^{31}P NMR (121 MHz, $CDCl_3$): δ -9.75.

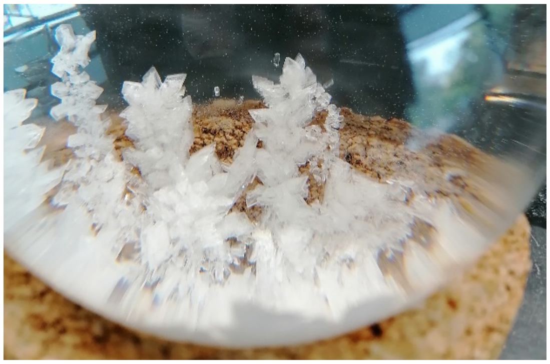

*Figure 1. Recrystallization of ligand **L3**.*

Scheme S3: General synthetic route to ligand **L9**

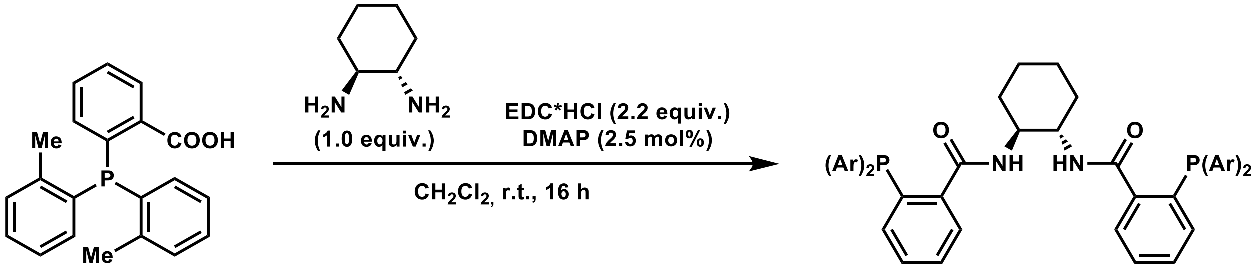

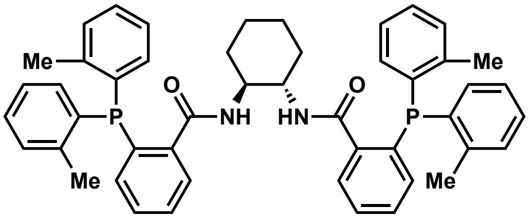

L9

Chemical Formula: $C_{48}H_{48}N_2O_2P_2$
Exact Mass: 746.3191
Molecular Weight: 746.8715

Ligand **L9** was prepared according to Scheme 3. Therefore 2-(di-o-tolylphosphaneyl)benzoic acid (0.97 g, 2.9 mmol, 2.1 equiv.) was dissolved in CH_2Cl_2 (10 mL). DMAP (4.3 mg, 0.035 mmol, 2.5 mol%) was added, followed by EDC*HCl (0.59 g, 3.08 mmol, 2.2 equiv.). The mixture was stirred at room temperature for 5 min. (1S,2S)-Cyclohexane-1,2-diamine (160 mg, 1.4 mmol, 1.0 equiv.) in CH_2Cl_2 (5 mL) was added and the resulting mixture was stirred at room temperature for 16 hours. The reaction was quenched with saturated NH_4Cl solution (~20 mL). The phases were separated, and the organic phase was dried over magnesium sulfate and filtered. The crude reaction mixture was

submitted to flash column chromatography over silica gel using hexane/EtOAc = 7/3 as the eluent to yield a white solid (0.81 g, 1.1 mmol, 79% yield).

^{1}H NMR (400 MHz, $CDCl_3$): δ 7.60 – 7.55 (m, 2H), 7.30 – 7.15 (m, 12H), 7.08 (t, *J* = 7.2 Hz, 2H), 7.02 – 6.96 (m, 2H), 6.87 (ddd, *J* = 7.5, 3.8, 1.5 Hz, 2H), 6.77 – 6.69 (m, 4H), 6.44 – 6.32 (bs, 2H), 3.77 – 3.66 (m, 2H), 2.33 (dd, *J* = 6.8, 1.6 Hz, 12H), 1.84 – 1.76 (m, 2H), 1.63 – 1.55 (m, 2H), 1.20 – 1.11 (m, 2H), 0.90 – 0.78 (m, 2H);

^{13}C NMR (101 MHz, $CDCl_3$): (several signals overlap, see spectra) δ 169.22, 169.21, 142.40, 142.14, 141.96, 141.68, 135.72, 135.60, 135.38, 135.26, 134.54, 134. 42, 134.35, 132.95, 132.90, 130.20, 130.18, 130.14, 130.10, 128.90, 128.75, 128.63, 127.80, 127.75, 126.42, 126.06, 53.71, 31.53, 24.56, 21.33, 21.25, 21.12, 21.04;

^{31}P NMR (121 MHz, $CDCl_3$): δ -24.79;

HRMS: m/z (ESI) calculated for $(M + H)^+$: 747.3269, found: 747.3271;

R$_f$ (Hexane/EtOAc = 7/3): 0.21;

ATR-IR ν (cm^{-1}): 3750, 3055, 3005, 2936, 2856, 2242, 1922, 1732, 1696, 1636, 1586, 1522, 1464, 1453, 1434, 1378, 1328, 1306, 1269, 1202, 1161, 1130, 1033, 910, 872, 829, 799, 751, 733.

General procedure 1: Suzuki-Miyaura cross coupling for the synthesis of substituted pyridines

Scheme S4: General procedure for the preparation of pyridine substrates

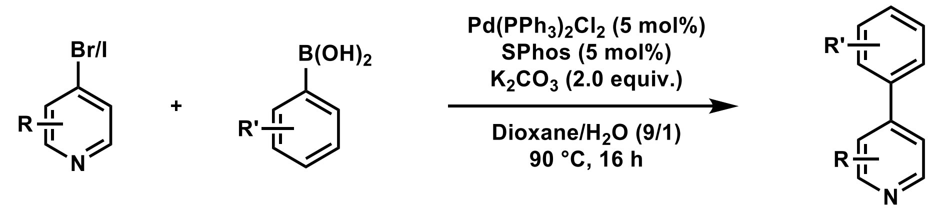

The pyridines (**1**) that were used in this study were either prepared according to literature procedure, were commercially available or were prepared as described above in Scheme 4. $Pd(PPh_3)_2Cl_2$ (5 mol%), SPhos (5 mol%) and K_2CO_3 (2.0 equiv.) were added to a 25 mL screw vial under air. The solid aryl boronic acid (1.2 equiv.) was added, followed by the pyridine (1.0 equiv.), if solid. The vial was sealed with a septum screw cap and evacuated using standard Schlenk line technology. The atmosphere was refilled with nitrogen. Dioxane and water (9/1) were added subsequentially (~0.2 M) and the mixture was stirred at room temperature. Pyridine (1.0 equiv.) was added, if liquid. The reaction mixture was then heated to 90 °C for 16 hours. The mixture was cooled to room temperature and diluted with EtOAc (~100 mL) and brine (~100 mL). The phases were separated and the organic phase was dried over magnesium sulfate and filtered. The pure aryl pyridines were separated by flash column chromatography over silica gel using hexane/EtOAc (typically 9/1 to 8/2) as eluent.

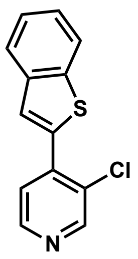

1l

Chemical Formula: $C_{13}H_8ClNS$
Exact Mass: 245.0066
Molecular Weight: 245.7240

4-(benzo[b]thiophen-2-yl)-3-chloropyridine **1l** was synthesized following the general procedure using 4-iodo-3-chloropyridine (1.0 g, 4.2 mmol, 1.0 equiv.) and benzo[b]thiophen-2-ylboronic acid (0.90 g, 5.04 mmol, 1.2 equiv.). The desired product **1l** was obtained as white solids (0.72 g, 2.93 mmol, 70% yield).

^{1}H NMR (400 MHz, CDCl$_3$): δ 8.71 (s, 1H), 8.51 (d, *J* = 5.1 Hz, 1H), 7.90 – 7.84 (m, 3H), 7.52 (dd, *J* = 5.1, 0.6 Hz, 1H), 7.43 – 7.38 (m, 2H);

^{13}C NMR (101 MHz, CDCl$_3$): δ 151.05, 148.00, 140.45, 140.13, 139.67, 137.18, 129.50, 126.42, 125.71, 124.97, 124.79, 124.58, 122.25;

HRMS: m/z (ESI) calculated for $(M + H)^+$: 246.0144, found: 246.0140;

R$_f$ (Hexane/EtOAc = 8.5/1.5): 0.18;

ATR-IR ν (cm^{-1}): 3054, 1578, 1473, 1435, 1396, 1239, 1103, 1040, 955, 864, 830, 761, 744, 722.

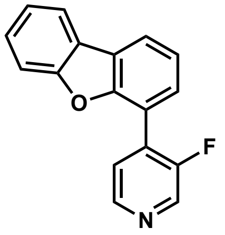

1m

Chemical Formula: $C_{17}H_{10}FNO$
Exact Mass: 263.0746
Molecular Weight: 263.2714

4-(dibenzo[b,d]furan-4-yl)-3-fluoropyridine **1m** was synthesized following the general procedure using 4-iodo-3-fluoropyridine (1.0 g, 4.5 mmol, 1.0 equiv.) and dibenzo[b,d]furan-4-ylboronic acid (1.14 g, 5.4 mmol, 1.2 equiv.). The desired product **1m** was obtained as white solids (0.5 g, 1.9 mmol, 42% yield).

^{1}H NMR (400 MHz, CDCl$_3$): δ 8.65 (d, *J* = 2.1 Hz, 1H), 8.58 (dd, *J* = 4.9, 1.0 Hz, 1H), 8.06 (dd, *J* = 7.7, 1.3 Hz, 1H), 8.01 (ddd, *J* = 7.7, 1.3, 0.7 Hz, 1H), 7.73 (dd, *J* = 6.3, 4.9 Hz, 1H), 7.63 – 7.57 (m, 2H), 7.52 – 7.44 (m, 2H), 7.39 (td, *J* = 7.5, 1.0 Hz, 1H);

^{13}C NMR (101 MHz, CDCl$_3$): δ 156.85 (d, *J* = 258.9 Hz), 156.30, 153.41, 145.93 (d, *J* = 5.2 Hz), 139.22 (d, *J* = 25.3 Hz), 131.78 (d, *J* = 12.0 Hz), 128.22 (d, *J* = 3.7 Hz), 127.81, 125.62, 125.26, 123.95, 123.27, 123.13, 121.92, 120.95, 117.58, 112.02;

^{19}F NMR (282 MHz, CDCl$_3$): δ -128.92 (d, *J* = 6.4 Hz);

HRMS: m/z (ESI) calculated for $(M + H)^+$: 264.0825, found: 264.0818;

R$_f$ (Hexane/EtOAc = 9/1): 0.38;

ATR-IR ν (cm^{-1}): 3055, 1601, 1470, 1450, 1421, 1406, 1264, 1206, 1188, 1153, 1050, 842, 831, 793, 743, 620.

Optimization for the Dearomative Asymmetric Allylic Alkylation

Scheme S5: General reaction scheme for optimization of the asymmetric allylic alkylation

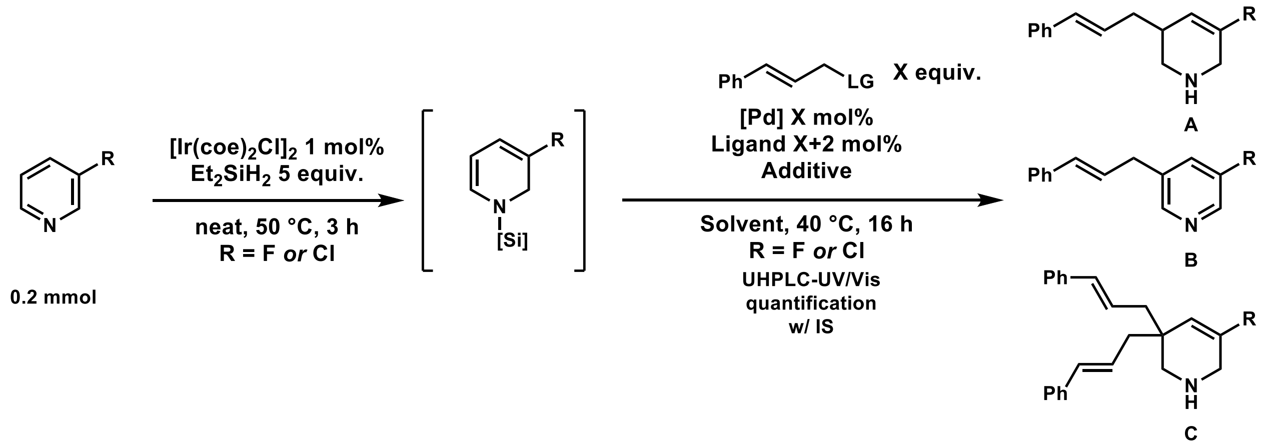

Optimization Procedure:

In a 2 mL screw vial, equipped with a magnetic stir bar was transferred to an argon filled glovebox. Chlor-bis-(cycloocten)-iridium(I) dimer ($[Ir(coe)_2Cl]_2$, 1.8 mg, 0.002 mmol, 1 mol%) was added to the vial. The vial was closed with a septum screw cap. The vial was transferred out of the glovebox. Diethyl silane (Et_2SiH_2, 130 µL, 1.0 mmol, 5.0 equiv.) was added and the resulting mixture was stirred at room temperature for 4 minutes. 3-Fluoroyridine (17 µL, 0.2 mmol, 1.0 equiv.) or 3-chloropyridine (19 µL, 0.2 mmol, 1.0 equiv.) was added and the resulting reaction mixture was stirred at 50 °C for 3 hours (R = F) or 1 hours (R = Cl). A separate 2 mL screw vial equipped with a magnetic stir bar was transferred to the glovebox. Ligand L$$ (X+2 mol%), palladium(II) acetate ($Pd(OAc)_2$, X mol%) and the additive, if solid were added. The vial was closed with a septum screw cap and transferred out of the glovebox. Dichloromethane (CH_2Cl_2, 1 mL) was added at room temperature under nitrogen. The mixture was stirred for 5 minutes and the additive was added, if liquid. The alkylation reagent was added, followed by the reaction mixture from the first 2 mL vial. The resulting mixture was then stirred at 40 °C for 16 h. The mixture was then cooled to room temperature and diluted with additional EtOAc (2 mL). 4-*tert*Butylpyridine (14.6 µL, 0.1 mmol) was added as internal standard. The mixture was homogenized and a small sample was prepared (~5 µL) that was diluted with additional EtOAc. The ratio between the product **A**, side products **B** and **C** and the internal standard was determined via UV/Vis absorption after separation via UHPLC.

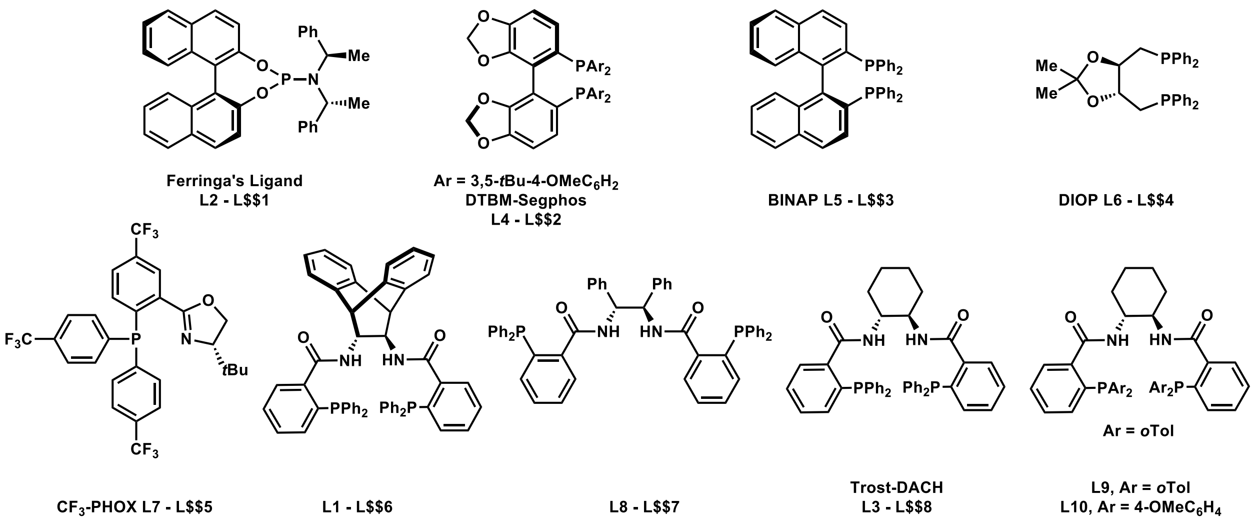

Figure 2. Molecular structure of ligands used in this study.

Table S1: Using 3-fluoroyridine (**1a**) as substrate (LG = OCO_2Me)

#	R=	Solvent	[Pd] (mol%)	Ligand (7 mol%)	Additive (mol%) and/or Other variation	A /%	ee A /%	B /%	C /%
1	F	CH_2Cl_2	Pd_2dba_3 (2.5)	PPh_3 (15)	-	2	-	11	<5
2	F	CH_2Cl_2	Pd_2dba_3 (2.5)	Ferringa L2	-	<5	48	n.d.	<5
3	F	CH_2Cl_2	Pd_2dba_3 (2.5)	DTBM Segphos L4	-	<5	42	n.d.	<5
4	F	CH_2Cl_2	Pd_2dba_3 (2.5)	BINAP L5	-	<5	28	17	<5
5	F	CH_2Cl_2	Pd_2dba_3 (2.5)	DIOP L6	-	<5	rac	n.d.	<5
6	F	CH_2Cl_2	Pd_2dba_3 (2.5)	CF_3Phox L7	-	n.r.	-	-	-
7	F	CH_2Cl_2	Pd_2dba_3 (2.5)	**L3**	-	**34**	**96**	**12**	**7**
8	F	CH_2Cl_2	Pd_2dba_3 (2.5)	oTol L9		n.r.	-	-	-
9	F	CH_2Cl_2	Pd_2dba_3 (2.5)	L8	-	n.r.	-	-	-
10	F	CH_2Cl_2	Pd_2dba_3 (2.5)	Anden L1	-	<5	30	<5	<5
11	F	PhH	Pd_2dba_3 (2.5)	L3	-	18	98	11	<5
12	F	THF	Pd_2dba_3 (2.5)	L3	-	15	98	24	<5
13	F	Diox.	Pd_2dba_3 (2.5)	L3	-	20	98	15	3
14	F	MeCN	Pd_2dba_3 (2.5)	L3	-	n.r.	-	-	-
15	F	Hexane	Pd_2dba_3 (2.5)	L3	-	n.r.	-	-	-
16	F	DMF	Pd_2dba_3 (2.5)	L3	-	n.r.	-	-	-
17	F	HFIP	Pd_2dba_3 (2.5)	L3	-	n.r.	-	-	-
18	F	DCE	Pd_2dba_3 (2.5)	L3	-	22	n.d.	15	<5
19	F	CH_2Cl_2	$Pd_2dba_3*CHCl_3$ (2.5)	L3	-	38	96	9	10
20	F	CH_2Cl_2	Pd_2dmba_3 (2.5)	L3	-	23	n.d.	5	<5
21	F	CH_2Cl_2	$Pd(OAc)_2$ (5)	L3	-	**38**	**96**	**8**	**5**
22	F	CH_2Cl_2	$[Pd(cinnamyl)Cl]_2$ (2.5)	L3	-	28	n.d.	12	<5
23	F	CH_2Cl_2	$Pd(OAc)_2$ (5)	L3	@ room temp.	38	n.d.	20	<5
24	F	CH_2Cl_2	$Pd(OAc)_2$ (5)	L3	LiO*t*Bu 1 eq.	n.r.	-	-	-
25	F	CH_2Cl_2	$Pd(OAc)_2$ (5)	L3	LiOAc 1 eq.	32	n.d.	17	<5
26	F	CH_2Cl_2	$Pd(OAc)_2$ (5)	L3	Na_2CO_3 1 eq.	30	n.d.	16	<5
27	F	CH_2Cl_2	$Pd(OAc)_2$ (5)	L3	DBU 1 eq.	5	n.d.	12	<5
28	F	CH_2Cl_2	$Pd(OAc)_2$ (5)	L3	Et_3N 1 eq.	38	n.d.	12	<5
29	F	CH_2Cl_2	$Pd(OAc)_2$ (5)	L3	CsF 1 eq.	**44**	**96**	**14**	**<5**

30	F	CH_2Cl_2	$Pd(OAc)_2$ (5)	L3	AcOH 0.5 eq.	35	n.d.	8	n.d.
31	F	CH_2Cl_2	$Pd(OAc)_2$ (5)	L3	$MesCO_2H$ 0.5 eq.	26	n.d.	7	n.d.
32	F	CH_2Cl_2	$Pd(OAc)_2$ (5)	L3	$ZnOTf_2$ 0.2 eq.	n.r.	-	-	-
33	F	CH_2Cl_2	$Pd(OAc)_2$ (5)	L3	Ph_3B 0.2 eq.	n.r.	-	-	-
34	F	CH_2Cl_2	$Pd(OAc)_2$ (5)	L3	Hantzsch 0.5 eq.	44	n.d.	13	<5
35	F	CH_2Cl_2	$Pd(OAc)_2$ (5)	L3	$NaBH(OAc)_3$	11	n.d.	20	<5
36	F	CH_2Cl_2	$Pd(OAc)_2$ (5)	L3	CsF 0.2 eq.	**48**	**n.d.**	**10**	**<5**
37	F	CH_2Cl_2	$Pd(OAc)_2$ (5)	L3	CsF 2.0 eq.	35	n.d.	34	<5
38	F	CH_2Cl_2	$Pd(OAc)_2$ (5)	L3	CsF 0.4 eq.	45	n.d.	14	<5
39	F	CH_2Cl_2	$Pd(OAc)_2$ (5)	L3	ZnF_2 0.2 eq.	20	n.d.	9	<5
40	F	CH_2Cl_2	$Pd(OAc)_2$ (5)	L3	AgF 0.2 eq.	n.r.	-	-	-
41	F	CH_2Cl_2	$Pd(OAc)_2$ (5)	L3	KF 0.2 eq.	41	n.d.	13	4
42	F	CH_2Cl_2	$Pd(OAc)_2$ (5)	L3	NaF 0.2 eq.	49	96	9	8
43	F	CH_2Cl_2	$Pd(OAc)_2$ (5)	L3	NaF 0.1 eq.	**49**	**96**	**8**	**6**
44	F	CH_2Cl_2	$Pd(OAc)_2$ (10)	L3	NaF 0.1 eq.	46	n.d.	8	4
45	F	CH_2Cl_2	$Pd(OAc)_2$ (5)	L3	LiCl 0.2 eq.	36	n.d.	<5	17
46	F	CH_2Cl_2	$Pd(OAc)_2$ (5)	L3	LiBr 0.2 eq.	24	n.d.	<5	10
47	F	CH_2Cl_2	$Pd(OAc)_2$ (5)	L3	LiI 0.2 eq.	n.r.	-	-	-
48	F	CH_2Cl_2	$Pd(OAc)_2$ (5)	4OMe L10	NaF 0.2 eq.	20	n.d.	12	7
49	F	CH_2Cl_2	$Pd(OAc)_2$ (5)	oTol L9	NaF 0.2 eq.	n.r.	-	-	-

Table S2: Using 3-chloropyridine (**1b**) as substrate (model substrate for compounds with increased substrate-specific selectivity for product C (dialkylated compounds)) (LG = OCO_2Me, if not mentioned otherwise):

#	R=	Solvent	[Pd] (mol%)	Ligand (mol%)	Additive (mol%) and/or Other variation	A /%	ee A /%	B /%	C /%
1	Cl	CH_2Cl_2	$Pd(OAc)_2$ (5)	L3	NaF 0.1 eq.	27	n.d.	24	43
2	Cl	CH_2Cl_2	$Pd(OAc)_2$ (5)	L3	NaF 0.1 eq. @ 60 °C	11	n.d.	<5	<5
3	Cl	PhH	$Pd(OAc)_2$ (5)	L3	NaF 0.1 eq.	24	n.d.	22	21
4	Cl	THF	$Pd(OAc)_2$ (5)	L3	NaF 0.1 eq.	18	n.d.	18	28
5	Cl	EtOAc	$Pd(OAc)_2$ (5)	L3	NaF 0.1 eq.	n.r.	-	-	-
6	Cl	Acetone	$Pd(OAc)_2$ (5)	L3	NaF 0.1 eq.	n.r.	-	-	-
7	Cl	MeOH	$Pd(OAc)_2$ (5)	L3	NaF 0.1 eq.	21	n.d.	30	11
8	Cl	CCl_4	$Pd(OAc)_2$ (5)	L3	NaF 0.1 eq.	n.r.	-	-	-

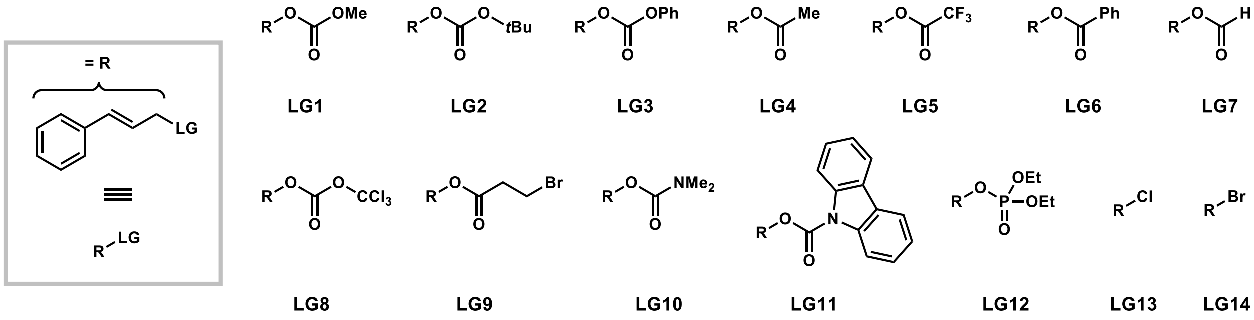

Figure 3. Optimization of leaving groups.

#	R=	Solvent	[Pd] (mol%)	Ligand (mol%)	Additive (mol%) and/or Other variation	A /%	ee A /%	B /%	C /%
9	Cl	CH_2Cl_2	$Pd(OAc)_2$ (5)	L3	NaF 0.1 eq./ **LG1**	27	n.d.	24	43
10	Cl	CH_2Cl_2	$Pd(OAc)_2$ (5)	L3	NaF 0.1 eq./ **LG2**	20	n.d.	18	38
11	Cl	CH_2Cl_2	$Pd(OAc)_2$ (5)	L3	NaF 0.1 eq./ **LG3**	31	n.d.	10	32

12	Cl	CH_2Cl_2	$Pd(OAc)_2$ (5)	L3	NaF 0.1 eq./ **LG4**	22	n.d.	21	24
13	Cl	CH_2Cl_2	$Pd(OAc)_2$ (5)	L3	NaF 0.1 eq./ **LG5**	4	n.d.	17	<5
14	Cl	CH_2Cl_2	$Pd(OAc)_2$ (5)	L3	NaF 0.1 eq./ **LG6**	14	n.d.	18	38
15	Cl	CH_2Cl_2	$Pd(OAc)_2$ (5)	L3	NaF 0.1 eq./ **LG7**	20	n.d.	21	13
16	Cl	CH_2Cl_2	$Pd(OAc)_2$ (5)	L3	NaF 0.1 eq./ **LG8**	28	n.d.	7	38
17	Cl	CH_2Cl_2	$Pd(OAc)_2$ (5)	L3	NaF 0.1 eq./ **LG9**	n.r.	-	-	-
18	Cl	CH_2Cl_2	$Pd(OAc)_2$ (5)	L3	NaF 0.1 eq./ **LG10**	n.r.	-	-	-
19	Cl	CH_2Cl_2	$Pd(OAc)_2$ (5)	L3	NaF 0.1 eq./ **LG11**	n.r.	-	-	-
20	Cl	CH_2Cl_2	$Pd(OAc)_2$ (5)	L3	NaF 0.1 eq./ **LG12**	13	n.d.	31	20
21	Cl	CH_2Cl_2	$Pd(OAc)_2$ (5)	L3	NaF 0.1 eq./ **LG13**	n.r.	-	-	-
22	Cl	CH_2Cl_2	$Pd(OAc)_2$ (5)	L3	NaF 0.1 eq./ **LG14**	n.r.	-	-	-
23	Cl	CH_2Cl_2	$Pd(OAc)_2$ (5)	L3	NaF 0.1 eq./ under air	n.r.	-	-	-
24	Cl	CH_2Cl_2	$Pd(OAc)_2$ (5)	L3	NaF 0.1 eq./ 2.5 eq. Et_2SiH_2 in step 1	29	n.d.	38	29
25	Cl	CH_2Cl_2	$Pd(OAc)_2$ (5)	L3	NaF 0.1 eq./ 7.5 eq. Et_2SiH_2 in step 1	37	n.d.	16	24
26	Cl	CH_2Cl_2	$Pd(OAc)_2$ (5)	L3	NaF 0.1 eq./ 0.5 mol% $[Ir]_2$ in step 1	14	n.d.	34	45
27	Cl	CH_2Cl_2	$Pd(OAc)_2$ (5)	L3	NaF 0.1 eq./ 2.5 mol% $[Ir]_2$ in step 1	5	n.d.	<5	<5
28	Cl	CH_2Cl_2	$Pd(OAc)_2$ (5)	L3	NaF 0.1 eq./ $PhCO_2H$ 0.2 eq.	**17**	**n.d.**	**7**	**61**

Scheme S6: Mixed pyridine control experiments

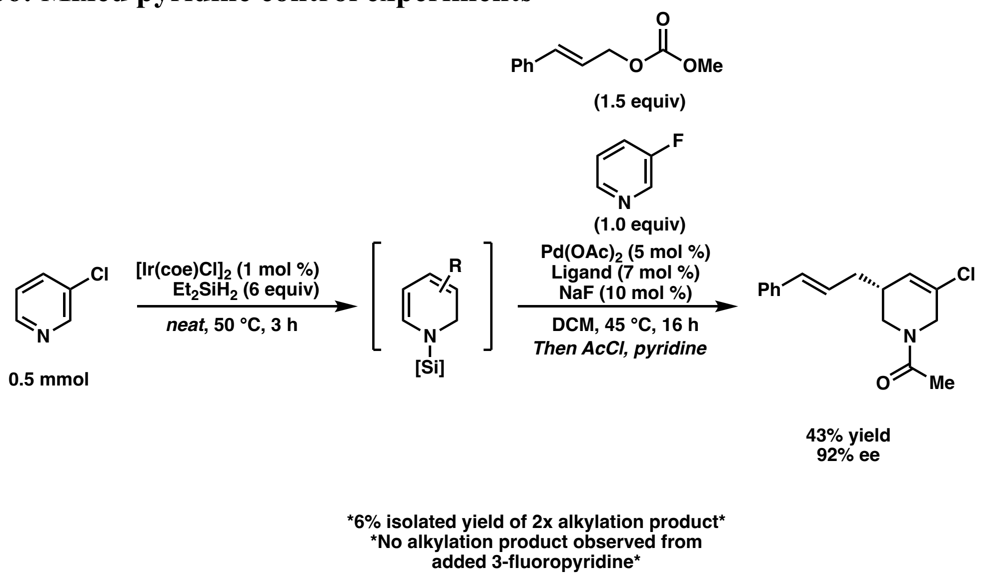

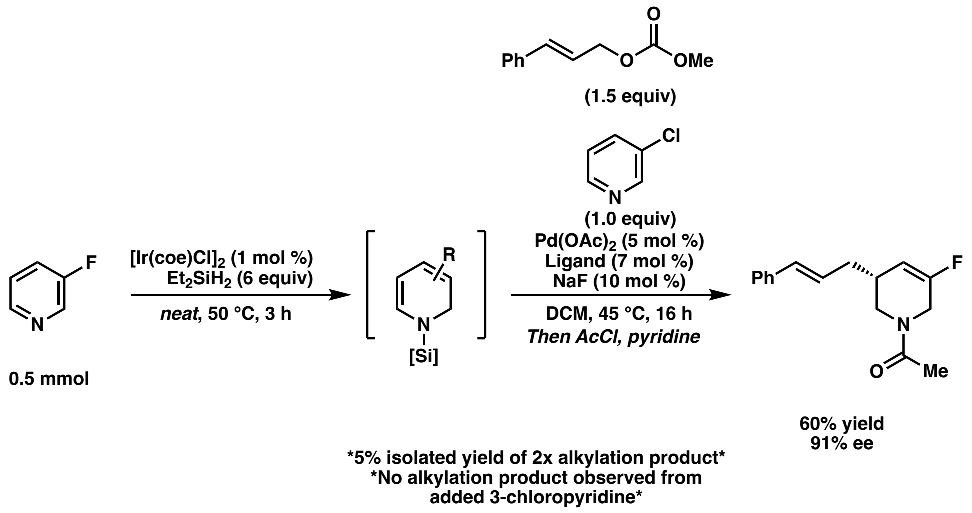

Mixed pyridine control experiments were performed as followed. Chlor-bis-(cycloocten)-iridium(I) dimer ([Ir(coe)$_2$Cl]$_2$, 4.5 mg, 0.005 mmol, 1 mol%) was added to a 1 dram vial equipped with a magnetic stir bar. Diethyl silane (Et_2SiH_2, 389 µL, 3.0 mmol, 6.0 equiv.) was added and the resulting mixture was stirred at room temperature for 15 minutes. 3-fluoropyridine or 3-chloropyridine (0.5 mmol, 1.0 equiv.) was added and the resulting reaction mixture was stirred at 50 °C for 3 hours. A separate 1 dram screw vial equipped with a magnetic stir bar was transferred to the glovebox. Ligand **L3** (24.2 mg, 0.035 mmol, 7 mol%), NaF (2.1 mg, 0.05 mmol, 10 mol%) and palladium(II) acetate ($Pd(OAc)_2$, 5.6 mg, 0.025 mmol, 5 mol%) were added. The vial was closed with a septum screw cap and transferred out of the glovebox. Dichloromethane (CH_2Cl_2, 2.5 mL) was added at room temperature under nitrogen. The mixture was stirred for 5 minutes. Cinnamyl methyl carbonate (144.2 mg, 0.75 mmol, 1.5 equiv.) was added, followed by the reaction mixture from the 1 dram vial. An additional equivalent of the alternative 3-halopyridine was added (0.5 mmol, 1.0 equiv). The resulting mixture was then

stirred at 40 °C for 24 h. The mixture was then cooled to room temperature and diluted with additional dichloromethane (2.5 mL). Pyridine (121 µL, 1.5 mmol, 3.0 equiv.) was added as a base, followed by acetyl chloride (107 µL, 1.5 mmol, 3.0 equiv.). The mixture was stirred at room temperature for 16 h. Afterwards, acetic acid (0.5 mL) was added and the mixture was stirred at room temperature for 2 hours. The mixture was diluted with dichloromethane and an aqueous work up with 4 M NaOH solution was performed to neutralize the acetic acid. The combined organic fractions were dried over magnesium sulfate. The solvent was evaporated and the residue was submitted to flash column chromatography over silica gel using a solvent mixture of hexane/ethyl acetate (typically 7/3 to 1/1) as the eluent.

Note 1: The ratios of monoalkylation and bisalkylation were obtained via isolation from column chromatography.

Note 2: No allylic alkylation product of any kind was obtained from the pyridine added at the Pd-catalyzed allylic alkylation step, suggesting that the iridium catalyzed dearomative hydrosilylation is not active during the Pd-catalyzed step.

Note 3: The hydrosilylation of the starting pyridines was incomplete, suggesting the yield could be increased through further optimization of this step.

Scheme S7: List of Unsuccessful Substrates

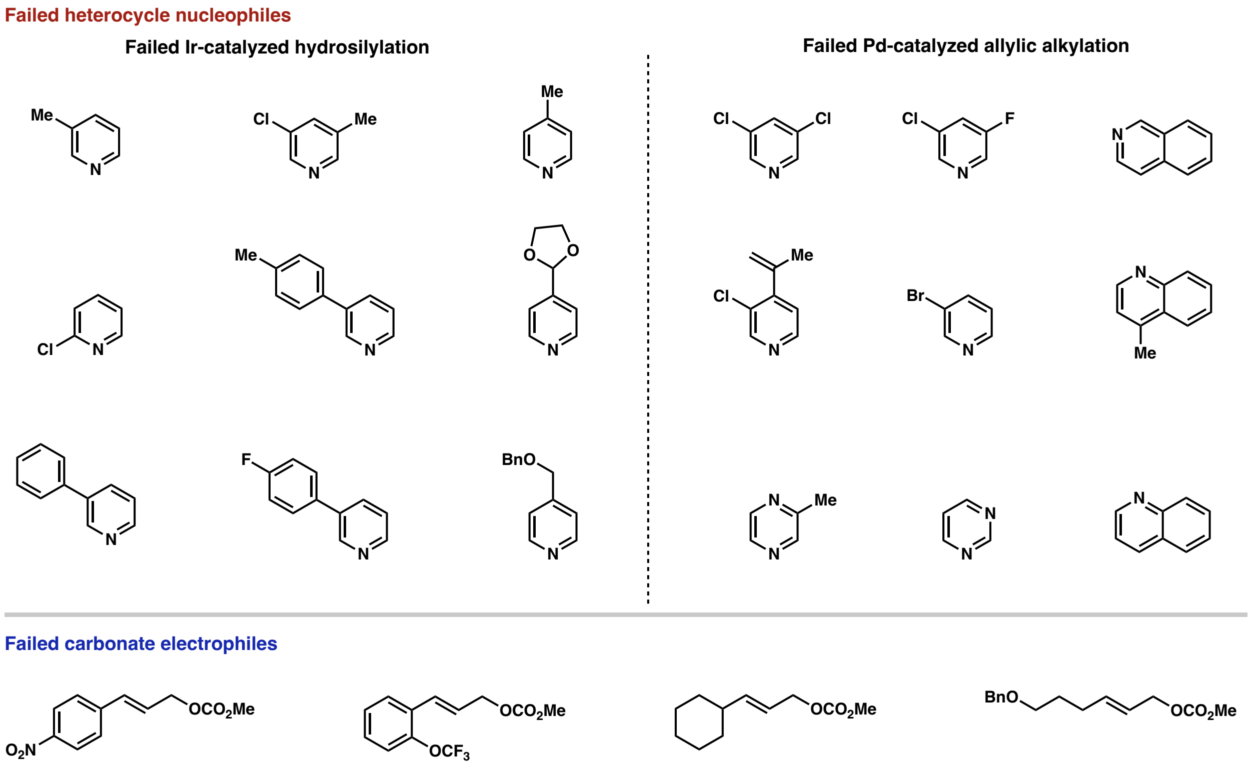

Product Characterization: Bisalkylated products

General procedure A for the synthesis of bisalkylated products (5)

Unless otherwise mentioned, compounds (**5**) were synthesized as followed: In a 2 mL screw vial, equipped with a magnetic stir bar, the corresponding pyridine (0.5 mmol, 1.0 equiv.) if solid was added to the vial. The vial was then transferred to an argon filled glovebox. Chlor-bis-(cycloocten)-iridium(I) dimer ($[Ir(coe)_2Cl]_2$, 4.5 mg, 0.005 mmol, 1 mol%) was added to the vial. The vial was closed with a septum screw cap. The vial was transferred out of the glovebox. Diethyl silane (Et_2SiH_2, 389 μL, 3.0 mmol, 6.0 equiv.) was added and the resulting mixture was stirred at room temperature for 4 minutes. Pyridine (0.5 mmol, 1.0 equiv.) if liquid was added and the resulting reaction mixture was stirred at 50 °C for the appropriate time (1 – 3 hours). A separate 10 mL screw vial equipped with a magnetic stir bar was transferred to the glovebox. Ligand **L3** (24.2 mg, 0.035 mmol, 7 mol%), NaF (2.1 mg, 0.05 mmol, 10 mol%), PhCOOH (12.2 mg, 0.1 mmol, 20 mol%) and palladium(II) acetate ($Pd(OAc)_2$, 5.6 mg, 0.025 mmol, 5 mol%) were added. The vial was closed with a septum screw cap and transferred out of the glovebox. Dichloromethane (CH_2Cl_2, 2.5 mL) was added at room temperature under nitrogen. The mixture was stirred for 5 minutes. Cinnamyl methyl carbonate (192.2 mg, 1.0 mmol, 2.0 equiv.) was added, followed by the reaction mixture from the 2 mL vial. The resulting mixture was then stirred at 40 °C for 24 h. Afterwards, the reaction mixture was cooled to room temperature and acetic acid (1 mL) was added and the mixture was stirred at room temperature for 2 hours. The mixture was diluted with dichloromethane and an aqueous work up with 4 M NaOH solution was performed to neutralize the acetic acid. The aqueous phase was extracted with dichloromethane once and ethyl acetate once. The combined organic fractions were dried over magnesium sulfate. The solvent was evaporated and the residue was submitted to flash column chromatography over silica gel using a solvent mixture of hexane/acetone = 7/3 as the eluent.

Product characterization

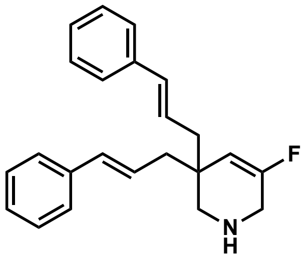

5a
Chemical Formula: $C_{23}H_{24}FN$
Exact Mass: 333.1893
Molecular Weight: 333.4504

3,3-dicinnamyl-5-fluoro-1,2,3,6-tetrahydropyridine 5a was synthesized following the general procedure A using 3-fluoropyridine (43 μL, 0.5 mmol, 1.0 equiv.) as substrate. The desired compound was obtained as colorless oil (43.7 mg, 0.131 mmol, 26% yield over two steps).

^{1}H NMR (400 MHz, CDCl$_3$): δ 7.29 – 7.20 (m, 8H), 7.16 – 7.11 (m, 2H), 6.36 (d, *J* = 15.7 Hz, 2H), 6.13 (dt, *J* = 15.5, 7.5 Hz, 2H), 5.15 (d, *J* = 18.3 Hz, 1H), 3.48 (bs, 1H), 3.25 (d, *J* = 1.5 Hz, 2H), 2. 68 (s, 2H), 2.28 – 2.17 (m, 4H);

^{13}C NMR (101 MHz, CDCl$_3$): δ 158.98 (d, *J* = 264.0 Hz), 137.39, 133.55, 128.72, 127.44, 126.25, 125.71, 108.17 (d, *J* = 10.2 Hz), 51.98 (d, *J* = 2.0 Hz), 43.70 (d, *J* = 30.2 Hz), 42.32 (d, *J* = 2.3 Hz), 39.76 (d, *J* = 4.7 Hz);

^{19}F NMR (282 MHz, CDCl$_3$): δ –110.42 (d, *J* = 18.3 Hz);

HRMS: m/z (ESI) calculated for $(M + H)^+$: 334.1971, found: 334.1958;

R$_f$ (Hexane/acetone = 7/3): 0.23;

ATR-IR ν (cm^{-1}): 3058, 3025, 2919, 2850, 1698, 1652, 1598, 1576, 1558, 1495, 1448, 1372, 1270, 1159, 1092, 1027, 967, 922, 853, 745, 694.

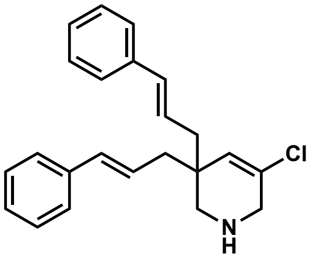

5b
Chemical Formula: $C_{23}H_{24}ClN$
Exact Mass: 349.1597
Molecular Weight: 349.9020

5-chloro-3,3-dicinnamyl-1,2,3,6-tetrahydropyridine 5b was synthesized following the general procedure A using 3-chloropyridine (47 µL, 0.5 mmol, 1.0 equiv.) as substrate. The desired compound was obtained as colorless oil (82.2 mg, 0.235 mmol, 47% yield over two steps).

^{1}H NMR (400 MHz, CDCl$_3$): δ 7.28 – 7.20 (m, 8H), 7.16 – 7.11 (m, 2H), 6.36 (d, *J* = 15.8 Hz, 2H), 6.11 (dt, *J* = 15.5, 7.5 Hz, 2H), 5.76 – 5.72 (m, 1H), 3.28 (d, *J* = 1.7 Hz, 2H), 2.71 (s, 2H), 2.42 (bs, 1H), 2.28 – 2.16 (m, 4H);

^{13}C NMR (101 MHz, CDCl$_3$): δ 137.32, 133.68, 132.23, 130.07, 128.71, 127.47, 126.25, 125.41, 51.48, 50.00, 41.90, 41.58;

HRMS: m/z (ESI) calculated for $(M + H)^+$: 350.1676, found: 350.1671;

R$_f$ (Hexane/acetone = 7/3): 0.23;

ATR-IR ν (cm^{-1}): 3054, 3025, 2916, 1651, 1599, 1494, 1448, 1072, 1004, 966, 856, 744, 692.

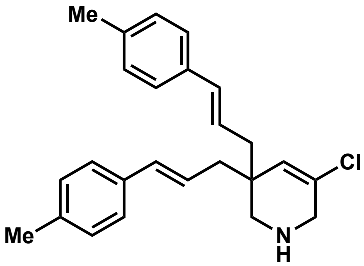

5c
Chemical Formula: $C_{25}H_{28}ClN$
Exact Mass: 377.1910
Molecular Weight: 377.9560

5-chloro-3,3-bis((*E*)-3-(*p*-tolyl)allyl)-1,2,3,6-tetrahydropyridine 5c was synthesized following the general procedure A using 3-chloropyridine (47.5 µL, 0.5 mmol, 1.0 equiv.) as substrate and (*E*)-methyl (3-(*p*-tolyl)allyl) carbonate (206 mg, 1.0 mmol, 2.0 equiv.). The desired compound was obtained as colorless oil (40 mg, 0.106 mmol, 21% yield over two steps).

^{1}H NMR (400 MHz, CDCl$_3$): δ 7.26 (d, *J* = 8.1 Hz, 4H), 7.13 (d, *J* = 7.8 Hz, 4H), 6.42 (d, *J* = 15.8 Hz, 2H), 6.14 (dt, *J* = 15.5, 7.5 Hz, 2H), 5.82 (s, 1H), 3.36 (d, *J* = 1.7 Hz, 2H), 2.79 (s, 2H), 2.34 (s, 6H), 2.29 (ddd, *J* = 8.0, 4.9, 1.3 Hz, 4H), 2.03 (s, 1H);

^{13}C NMR (101 MHz, CDCl$_3$): δ 137.3, 134.6, 133.5, 132.2, 130.2, 129.4, 126.2, 124.4, 51.5, 49.2, 41.9, 41.6, 21.3;

^{19}F NMR (282 MHz, CDCl$_3$): δ –110.42 (d, *J* = 18.3 Hz);

HRMS: m/z (ESI) calculated for $(M + H)^+$: 378.1989, found: 378.2011;

Rf (*n*-hexane/ethyl acetate = 7/2): 0.27;

ATR-IR ν (cm^{-1}): 3023, 2920, 1747, 1699, 1651, 1512, 1435, 1264, 1108, 968, 795.

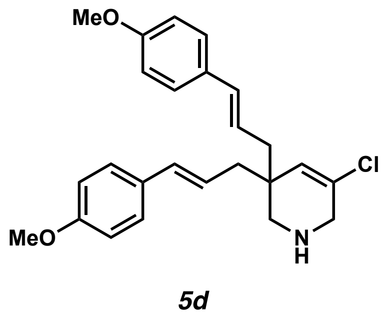

5d
Chemical Formula: $C_{25}H_{28}ClNO_2$
Exact Mass: 409.1809
Molecular Weight: 409.9540

5-chloro-3,3-bis((E)-3-(4-methoxyphenyl)allyl)-1,2,3,6-tetrahydropyridine 5d was synthesized following the general procedure A using 3-chloropyridine (47.5 µL, 0.5 mmol, 1.0 equiv.) as substrate and (*E*)-3-(4-methoxyphenyl)allyl methyl carbonate (222 mg, 1.0 mmol, 2.0 equiv.). The desired compound was obtained as colorless oil (57 mg, 0.139 mmol, 28% yield over two steps).

^{1}H NMR (400 MHz, CDCl$_3$): δ 7.21 (d, *J* = 8 Hz, 4H), 6.78 (d, *J* = 8 Hz, 4H), 6.31 (d, *J* = 15.8 Hz, 2H), 5.98 (ddd, *J* = 15.4, 8.0, 7.1 Hz, 2H), 5.75 (s, 1H), 3.73 (s, 6H), 3.29 (s, 2H), 2.87 (br s, 1H), 2.72 (s, 2H), 2.26 – 2.14 (m, 4H);

^{13}C NMR (101 MHz, CDCl$_3$): δ 159.1, 133.0, 130.3, 130.2, 128.3, 127.4, 123.2, 114.1, 55.4, 51.5, 50.0, 41.9, 41.6;

HRMS: m/z (ESI) calculated for (M + H)$^+$: 410.1887, found: 410.1861;

Rf (*n*-hexane/ethyl acetate/acetone = 7/2/1): 0.12;

ATR-IR ν (cm^{-1}): 3029, 3002, 2931, 2834, 1653, 1606, 1576, 1510, 1461, 1441, 1298, 1248, 1174, 1107, 1034, 1034, 967, 905, 839, 753, 644.

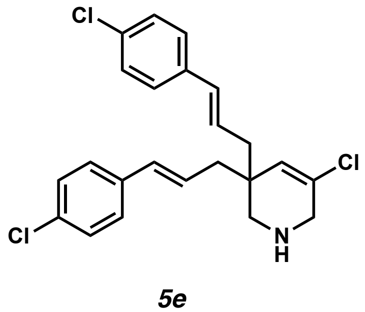

5e
Chemical Formula: $C_{23}H_{22}Cl_3N$
Exact Mass: 417.0818
Molecular Weight: 418.7860

5-chloro-3,3-bis((*E*)-3-(4-chlorophenyl)allyl)-1,2,3,6-tetrahydropyridine 5e was synthesized following the general procedure A using 3-chloropyridine (47.5 µL, 0.5 mmol, 1.0 equiv.) as substrate and (*E*)-3-(4-chlorophenyl)allyl methyl carbonate (226 mg, 1.0 mmol, 2.0 equiv.). The desired compound was obtained as yellowish oil (63 mg, 0.150 mmol, 30% yield over two steps).

^{1}H NMR (400 MHz, CDCl$_3$): δ 7.27 (s, 8H), 6.40 (d, *J* = 15.8 Hz, 2H), 6.25 – 6.03 (m, 2H), 5.81 (s, 1H), 3.37 (d, *J* = 1.7 Hz, 2H), 2.79 (s, 2H), 2.35 – 2.27 (m, 4H), 2.21 (br s, 1H);

^{13}C NMR (101 MHz, CDCl$_3$): δ 135.7, 133.1, 132.5, 132.5, 129.8, 128.9, 127.5, 126.1, 51.5, 49.6, 41.9, 41.6;

HRMS: m/z (ESI) calculated for $(M + H)^+$: 418.0896, found: 418.0882;

R$_f$ (*n*-hexane/ethyl acetate/acetone = 7/2/1): 0.19;

ATR-IR ν (cm^{-1}): 3027, 2917, 2839, 1651, 1489, 1434, 1404, 1264, 1093, 1012, 969, 896, 846, 736.

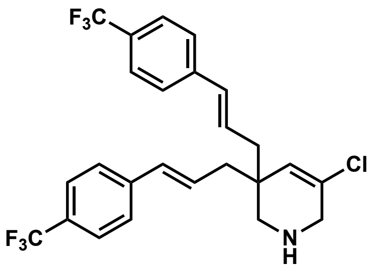

5f
Chemical Formula: $C_{25}H_{22}ClF_6N$
Exact Mass: 485.1345
Molecular Weight: 485.8984

5-chloro-3,3-bis((*E*)-3-(4-(trifluoromethyl)phenyl)allyl)-1,2,3,6-tetrahydropyridine 5f was synthesized following the general procedure A using 3-chloropyridine (47.5 µL, 0.5 mmol, 1.0 equiv.) as substrate and (*E*)-methyl (3-(4-(trifluoromethyl)phenyl)allyl) carbonate (260 mg, 1.0 mmol, 2.0 equiv.). The desired compound was obtained as colorless oil (52 mg, 0.107 mmol, 21% yield over two steps).

^{1}H NMR (400 MHz, CDCl$_3$): δ 7.57 (d, *J* = 8.2 Hz, 4H), 7.44 (d, *J* = 8.3 Hz, 4H), 6.50 (d, *J* = 15.8 Hz, 2H), 6.30 (dt, *J* = 15.6, 7.5 Hz, 2H), 5.83 (s, 1H), 3.40 (s, 2H) 2.82 (s, 2H), 2.53–2.28 (m, 5H);

^{13}C NMR (101 MHz, CDCl$_3$): δ 140.1, 132.7, 132.4, 129.6, 129.4 (q, *J* = 32.3 Hz), 128.1, 126.4, 125.7 (q, *J* = 3.9 Hz), 124.3 (q, *J* = 272 Hz), 51.3, 49.9, 42.0, 41.6;

^{19}F NMR (282 MHz, CDCl$_3$): δ –62.5;

HRMS: m/z (ESI) calculated for $(M + H)^+$: 486.1423, found: 486.1413;

R$_f$ (*n*-hexane/ethyl acetate/acetone = 7/2/1): 0.19;

ATR-IR ν (cm^{-1}): 2921, 1652, 1615, 1415, 1326, 1170, 1160, 1123, 1068, 1016, 971, 953, 861.

Product Characterization: Monoalkylated products

General procedure B for the synthesis of 6 (electron poor pyridine substrates)

Some compounds **6** were synthesized as followed (as mentioned in the product characterization): In a 2 mL screw vial, equipped with a magnetic stir bar, the corresponding pyridine (0.5 mmol, 1.0 equiv.) if solid was added to the vial. The vial was then transferred to an argon filled glovebox. Chlor-bis-(cycloocten)-iridium(I) dimer ($[Ir(coe)_2Cl]_2$, 4.5 mg, 0.005 mmol, 1 mol%) was added to the vial. The vial was closed with a septum screw cap. The vial was transferred out of the glovebox. Diethyl silane (Et_2SiH_2, 389 µL, 3.0 mmol, 6.0 equiv.) was added and the resulting mixture was stirred at room temperature for 4 minutes. Pyridine (0.5 mmol, 1.0 equiv.) if liquid was added and the resulting reaction mixture was stirred at 50 °C for the appropriate time (0.5 – 3 hours). A separate 10 mL screw vial equipped with a magnetic stir bar was transferred to the glovebox. Ligand **L3** (24.2 mg, 0.035 mmol, 7 mol%), NaF (2.1 mg, 0.05 mmol, 10 mol%) and palladium(II) acetate ($Pd(OAc)_2$, 5.6 mg, 0.025 mmol, 5 mol%) were added. The vial was closed with a septum screw cap and transferred out of the glovebox. Dichloromethane (CH_2Cl_2, 2.5 mL) was added at room temperature under nitrogen. The mixture was stirred for 5 minutes. Cinnamyl methyl carbonate (144.2 mg, 0.75 mmol, 1.5 equiv.) was added, followed by the reaction mixture from the 2 mL vial. The resulting mixture was then stirred at 40 °C for 24 h. The mixture was then cooled to room temperature and diluted with additional dichloromethane (2.5 mL). Pyridine (121 µL, 1.5 mmol, 3.0 equiv.) was added as a base, followed by acetyl chloride (107 µL, 1.5 mmol, 3.0 equiv.). The mixture was stirred at room temperature for 16 h. Afterwards, acetic acid (0.5 mL) was added and the mixture was stirred at room temperature for 2 hours. The mixture was diluted with dichloromethane and an aqueous work up with 4 M NaOH solution was performed to neutralize the acetic acid. The aqueous phase was extracted with dichloromethane once and ethyl acetate once. The combined organic fractions were dried over magnesium sulfate. The solvent was evaporated and the residue was submitted to flash column chromatography over silica gel using a solvent mixture of hexane/ethyl acetate (typically 7/3 to 1/1) as the eluent.

General procedure C for the synthesis of 6 (neutral or electron rich pyridines)

Some compounds **6** were synthesized as followed (as mentioned in the product characterization): In a 2 mL screw vial, equipped with a magnetic stir bar, the corresponding pyridine (0.5 mmol, 1.0 equiv.) if solid was added to the vial. The vial was then transferred to an argon filled glovebox. Chlor-bis-(cyclooncten)-iridium(I) dimer ($[Ir(coe)_2Cl]_2$, 2.3 mg, 0.0025 mmol, 0.5 mol%) was added to the vial. Diethyl silane (Et_2SiH_2, 389 µL, 3.0 mmol, 6.0 equiv.) was added and the resulting mixture was stirred at room temperature for 4 minutes. Pyridine (0.5 mmol, 1.0 equiv.) if liquid was added and the vial was closed with a screw cap and the resulting reaction mixture was stirred at 45 °C for 6 h. The mixture was cooled to room temperature and additional $[Ir(coe)_2Cl]_2$ catalyst (2.3 mg, 0.0025 mmol, 0.5 mol%) and Et_2SiH_2 (195 µL, 1.5 mmol, 3 equiv.) were added and the mixture was again stirred at 45 °C for 12 hours. The mixture was again cooled to room temperature and additional $[Ir(coe)_2Cl]_2$ catalyst (2.3 mg, 0.0025 mmol, 0.5 mol%) and Et_2SiH_2 (195 µL, 1.5 mmol, 3 equiv.) were added for the third time and the mixture again stirred at 45 °C for 2 hours. Afterwards, the mixture was transferred out of the glovebox. A separate 10 mL screw vial equipped with a magnetic stir bar was transferred to the glovebox.

Ligand **L3** (24.2 mg, 0.035 mmol, 7 mol%), NaF (2.1 mg, 0.05 mmol, 10 mol%) and palladium(II) acetate ($Pd(OAc)_2$, 5.6 mg, 0.025 mmol, 5 mol%) were added. The vial was closed with a septum screw cap and transferred out of the glovebox. Dichloromethane (CH_2Cl_2, 2.5 mL) was added at room temperature under nitrogen. The mixture was stirred for 5 minutes. Cinnamyl methyl carbonate (144.2 mg, 0.75 mmol, 1.5 equiv.) was added, followed by the reaction mixture from the 2 mL vial. The resulting mixture was then stirred at 40 °C for 24 h. The mixture was then cooled to room temperature and diluted with additional dichloromethane (2.5 mL). Pyridine (121 µL, 1.5 mmol, 3.0 equiv.) was added as a base, followed by acetyl chloride (107 µL, 1.5 mmol, 3.0 equiv.). The mixture was stirred at room temperature for 16 h. Afterwards, acetic acid (0.5 mL) was added and the mixture stirred at room temperature for 2 hours. The mixture was diluted with dichloromethane and an aqueous work up with 4 M NaOH solution was performed to neutralize the acetic acid. The aqueous phase was extracted with dichloromethane once and ethyl acetate once. The combined organic fractions were dried over magnesium sulfate. The solvent was evaporated and the residue was submitted to flash column chromatography over silica gel using a solvent mixture of hexane/ethyl acetate (typically 7/3 to 1/1) as the eluent.

General procedure D for the synthesis of 6 (alternative for neutral or electron rich pyridines)

Some compounds **6** were synthesized as followed (as mentioned in the product characterization): In a 4 mL screw vial, equipped with a magnetic stir bar, the corresponding pyridine (0.5 mmol, 1.0 equiv.) if solid was added to the vial. The vial was then transferred to an argon filled glovebox. Chlor-bis-(cycloocten)-iridium(I) dimer ($[Ir(coe)_2Cl]_2$, 6.9 mg, 0.0075 mmol, 1.5 mol%) was added to the vial. Diethyl silane (Et_2SiH_2, 518 µL, 4.0 mmol, 8.0 equiv.) was added followed by dichloromethane (CH_2Cl_2, 1.0 mL), and the resulting mixture was stirred at room temperature for 20 minutes. Pyridine (0.5 mmol, 1.0 equiv.) if liquid was added neat to the reaction mixture and the vial was closed with a screw cap. The resulting reaction mixture was stirred at 45 °C for 6–16 h (depending on the pyridine). Afterwards, the mixture was transferred out of the glovebox. A separate 10 mL screw vial equipped with a magnetic stir bar was transferred to the glovebox. Ligand **L3** (24.2 mg, 0.035 mmol, 7 mol%), NaF (2.1 mg, 0.05 mmol, 10 mol%) and palladium(II) acetate ($Pd(OAc)_2$, 5.6 mg, 0.025 mmol, 5 mol%) were added. The vial was closed with a septum screw cap and transferred out of the glovebox. Dichloromethane (CH_2Cl_2, 2.5 mL) was added at room temperature under nitrogen. The mixture was stirred for 5 minutes. Cinnamyl methyl carbonate (144.2 mg, 0.75 mmol, 1.5 equiv.) was added, followed by the reaction mixture from the 2 mL vial. The resulting mixture was then stirred at 40 °C for 24 h. The mixture was then cooled to room temperature and diluted with additional dichloromethane (2.5 mL). Pyridine (121 µL, 1.5 mmol, 3.0 equiv.) was added as a base, followed by acetyl chloride (107 µL, 1.5 mmol, 3.0 equiv.). The mixture was stirred at room temperature for 16 h. Afterwards, acetic acid (0.5 mL) was added and the mixture stirred at room temperature for 2 hours. The mixture was diluted with dichloromethane and an aqueous work up with 4 M NaOH solution was performed to neutralize the acetic acid. The aqueous phase was extracted with dichloromethane once and ethyl acetate once. The combined organic fractions were dried over magnesium sulfate. The solvent was evaporated and the residue was submitted to flash column chromatography over silica gel using a solvent mixture of hexane/ethyl acetate (typically 7/3 to 2/8) as the eluent.

Product characterization for mono alkylated products (6)

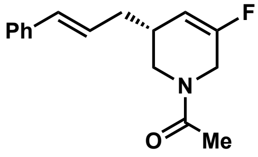

6a

Chemical Formula: $C_{16}H_{18}FNO$
Exact Mass: 259.1372
Molecular Weight: 259.3244

(R)-1-(3-cinnamyl-5-fluoro-3,6-dihydropyridin-1(2H)-yl)ethan-1-one 6a was synthesized following the general procedure B. Therefore, 3-fluoropyridine (43 µL, 0.5 mmol, 1.0 equiv.) was used. The desired product was isolated as colorless oil (55.3 mg, 0.213 mmol, 43% yield over three steps).

^{1}H NMR (400 MHz, $CDCl_3$) (as mixture of E/Z amide bond isomers): δ 7.37 – 7.27 (m, 4H), 7.25 – 7.18 (m, 1H), 6.48 – 6.40 (m, 1H), 6.20 – 6.10 (m, 1H), 5.47 – 5.40 (m, 0.4H), 5.36 (ddt, *J* = 16.1, 3.5, 1.5 Hz, 0.6H), 4.18 – 4.05 (m, 1.4H), 3.97 (dt, *J* = 4.0, 2.0 Hz, 0.6H), 3.90 (dd, *J* = 13.1, 4.6 Hz, 0.4H), 3.53 (dd, *J* = 13.4, 4.5 Hz, 0.6H), 3.26 (dt, *J* = 13.4, 6.8 Hz, 1H), 2.56 – 2.44 (m, 1H), 2.34 – 2.17 (m, 2H), 2.13 – 2.11 (m, 3H);

^{13}C NMR (101 MHz, $CDCl_3$) (as mixture of E/Z amide bond isomers, signals of minor isomer are indicated with *): δ 169.81, 169.64*, 156.33 (d, *J* = 255.5 Hz), 154.84 (d *J* = 255.2 Hz)*, 137.34*, 137.04, 133.01, 132.75*, 128.75, 128.63*, 127.62, 127.35*, 126.68, 126.24*, 126.19, 106.12 (d, *J* = 11.0 Hz)*, 104.49 (d, *J* = 12.3 Hz), 47.65, 44.84 (d, *J* = 39.2 Hz)*, 42.99*, 41.16 (d, *J* = 39.9 Hz), 36.86 (d *J* = 2.0 Hz)*, 36.77 (d, *J* = 2.3 Hz), 34.29 (d, *J* = 6.3 Hz), 33.37 (d, *J* = 5.9 Hz)*, 22.02*, 21.55;

^{19}F NMR (282 MHz, CD_3OD) (as mixture of E/Z amide bond isomers, signals of minor isomer are indicated with *): δ –114.85 (dd, *J* = 16.4, 5.0 Hz), –116.04 (dd, *J* = 16.4, 5.1 Hz)*;

HRMS: m/z (ESI) calculated for $(M + H)^+$: 260.1451, found: 260.1438;

R_f (Hexane/EtOAc = 6/4): 0.15;

ATR-IR ν (cm^{-1}): 3853, 3745, 3675, 3648, 3026, 2914, 1707, 1652, 1491, 1436, 1380, 1361, 1274, 1230, 1162, 1106, 1070, 1032, 969, 831, 745, 695, 682, 618;

$[\alpha]_D^{25}$: – 42.82 (c 1.0, $CHCl_3$);

Chiral SFC Separation: 20% MeOH, 2.5 mL/min, AD-H column, λ = 254 nm, t_R (min): minor = 5.75 (area: 2.74%), major = 7.61 (area: 97.26%), 94.5% enantiomeric excess.

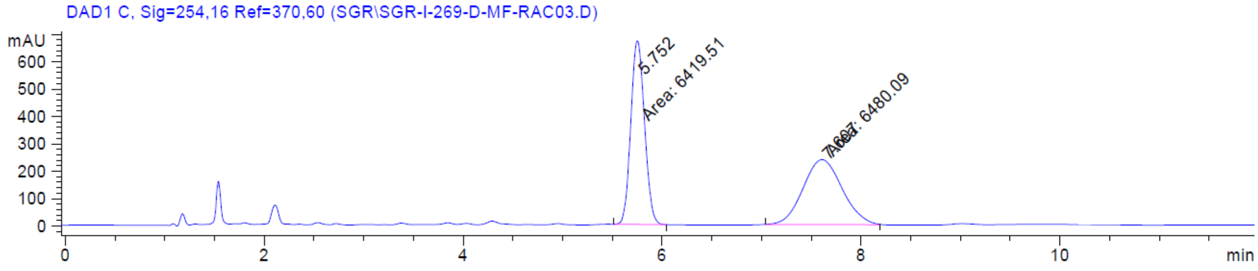

Signal 2: DAD1 C, Sig=254,16 Ref=370,60

Peak #	RetTime [min]	Type	Width [min]	Area [mAU*s]	Height [mAU]	Area %
1	5.752	MM	0.1593	6419.50684	671.81226	49.7652
2	7.607	MM	0.4550	6480.09131	237.34406	50.2348

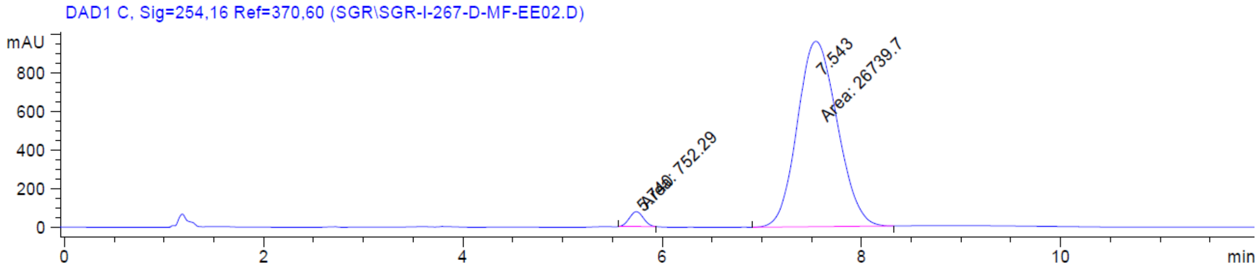

Signal 2: DAD1 C, Sig=254,16 Ref=370,60

Peak #	RetTime [min]	Type	Width [min]	Area [mAU*s]	Height [mAU]	Area %
1	5.740	MM	0.1632	752.29022	76.81909	2.7364
2	7.543	MM	0.4648	2.67397e4	958.75690	97.2636

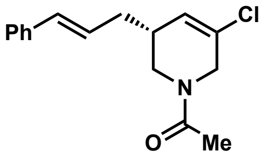

6b
Chemical Formula: $C_{16}H_{18}ClNO$
Exact Mass: 275.1077
Molecular Weight: 275.7760

(R)-1-(5-chloro-3-cinnamyl-3,6-dihydropyridin-1(2H)-yl)ethan-1-one 6b was synthesized following the general procedure B. Therefore, 3-chloropyridine (48 µL, 0.5 mmol, 1.0 equiv.) was used. The desired product was isolated as colorless oil (52.0 mg, 0.138 mmol, 38% yield over three steps).

^{1}H NMR (400 MHz, CD_3OD) (as mixture of E/Z amide bond isomers): δ 7.40 – 7.35 (m, 2H), 7.32 – 7.25 (m, 2H), 7.23 – 7.16 (m, 1H), 6.53 – 6.43 (m, 1H), 6.29 – 6.19 (m, 1H), 6.03 (dt, J = 3.8, 1.9 Hz, 0.4H), 5.99 (dt, J = 3.7, 1.8 Hz, 0.6H), 4.23 – 4.02 (m, 2H), 3.75 (dd, J = 13.1, 4.7 Hz, 0.4H), 3.69 (dd, J = 13.7, 4.7 Hz, 0.6 H), 3.45 (dd, J = 13.1, 6.3 Hz, 0.4H), 3.37 – 3.32 (m, 0.6H), 2.65 – 2.57 (m, 0.6H), 2.52 – 2.45 (m,0.4H), 2.34 – 2.18 (m, 2H), 2.13 – 2.11 (m, 3H);

^{13}C NMR (101 MHz, CD_3OD) (as mixture of E/Z amide bond isomers, signals of minor isomer are indicated with *): δ 172.01, 172.00*, 138.77*, 138.58, 134.01, 133.86*, 129.59, 129.52*, 129.22*, 129.07, 128.44*, 128.36, 128.21*, 128.11, 127.72, 127.15, 51.06*, 48.16, 47.26, 43.15*, 38.22, 37.74*, 37.30*, 37.11, 21.69*, 21.23;

HRMS: m/z (ESI) calculated for $(M + H)^+$: 276.1155, found: 276.1174;

R_f (Hexane/EtOAc = 6/4): 0.15;

ATR-IR ν (cm^{-1}): 3852, 3734, 3673, 3648, 3025, 2920, 1716, 1647, 1490, 1424, 1369, 1272, 1231, 968, 847, 780, 768, 746, 695, 678;

$[\alpha]_D^{25}$: – 22.17 (c 1.0, $CHCl_3$);

Chiral SFC Separation: 10% *i*PrOH, 2.5 mL/min, AD-H column, λ = 254 nm, t_R (min): major = 11.94 (area: 96.32%), minor = 13.46 (area: 3.68%), 92.6% enantiomeric excess.

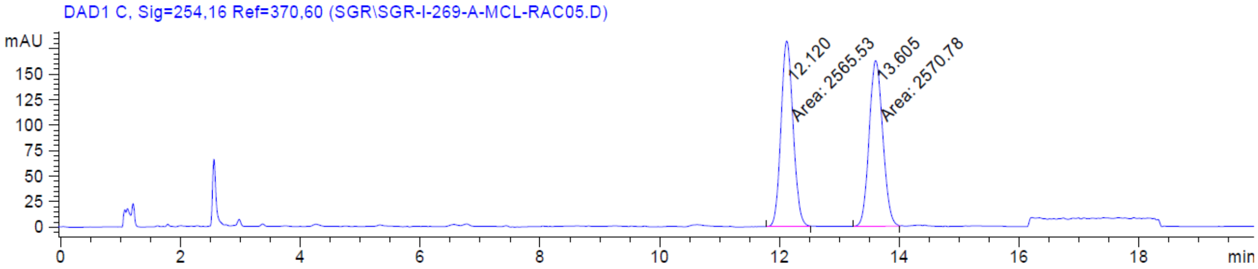

Signal 2: DAD1 C, Sig=254,16 Ref=370,60

Peak #	RetTime [min]	Type	Width [min]	Area [mAU*s]	Height [mAU]	Area %
1	12.120	MM	0.2348	2565.52881	182.12392	49.9488
2	13.605	MM	0.2635	2570.78491	162.62581	50.0512

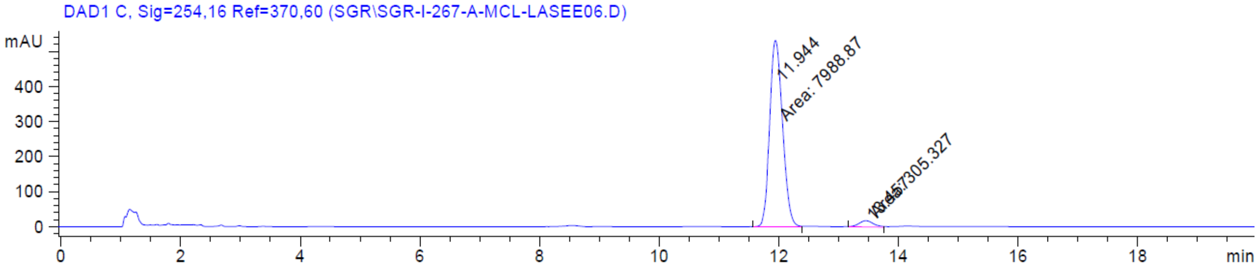

Signal 2: DAD1 C, Sig=254,16 Ref=370,60

Peak #	RetTime [min]	Type	Width [min]	Area [mAU*s]	Height [mAU]	Area %
1	11.944	MM	0.2497	7988.87451	533.30609	96.3188
2	13.457	MM	0.2841	305.32715	17.91254	3.6812

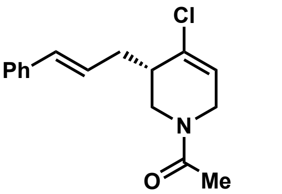

6c
Chemical Formula: $C_{16}H_{18}ClNO$
Exact Mass: 275.1077
Molecular Weight: 275.7760

(S)-1-(4-chloro-3-cinnamyl-3,6-dihydropyridin-1(2H)-yl)ethan-1-one 6c was synthesized following the general procedure B. Therefore, 4-chloropyridine (57 mg, 0.5 mmol, 1.0 equiv.) was used. The desired product was isolated as colorless oil (37.5 mg, 0.136 mmol, 27% yield over three steps).

^{1}H NMR (400 MHz, CD_3OD) (as mixture of E/Z amide bond isomers): δ 7.39 – 7.34 (m, 2H), 7.31 – 7.24 (m, 2H), 7.22 – 7.15 (m, 1H), 6.57 – 6.45 (m, 1H), 6.29 – 6.19 (m, 1H), 5.94 – 5.89 (m, 1H), 4.41 – 4.34 (m, 1H), 4.16 (dd, *J* = 17.7, 3.9 Hz, 0.5H), 3.96 (dt, *J* = 17.7, 2.6 Hz, 0.5H), 3.81 (dd, *J* = 13.7, 3.3 Hz, 0.5H), 3.73 (dt, *J* = 18.8, 2.6 Hz, 0.5H), 3.54 (dd, *J* = 13.7, 4.2 Hz, 0.5H), 3.19 (dd, *J* = 13.2, 4.3 Hz, 0.5H), 2.74 – 2.47 (m, 2H), 2.42 – 2.21 (m, 1H), 2.11 (s, 1.5H), 2.06 (s, 1.5H);

^{13}C NMR (101 MHz, CD_3OD) (as mixture of E/Z amide bond isomers, approx. 1/1 mixture): δ 172.49, 172.33, 138.88, 138.56, 135.14, 134.17, 134.10, 134.05, 129.60, 129.50, 128.40, 128.18, 127.81, 127.39, 127.17, 127.16, 123.17, 122.71, 48.03, 47.05, 43.74, 43.72, 43.48, 43.14, 35.18, 35.13, 21.61, 21.26;

HRMS: m/z (ESI) calculated for $(M + H)^+$: 276.1155, found: 276.1131;

R_f (Hexane/EtOAc = 6/4): 0.11;

ATR-IR ν (cm^{-1}): 3866, 3733, 3648, 2922, 1646, 1425, 1360, 1237, 1032, 1010, 969, 822, 770, 746, 718, 699;

$[\alpha]_D^{25}$: – 31.97 (c 0.33, $CHCl_3$);

Chiral SFC Separation: 7% MeOH, 2.5 mL/min, AS-H column, λ = 254 nm, t_R (min): major = 8.49 (area: 91.60%), minor = 9.43 (area: 8.40%), 83.2% enantiomeric excess.

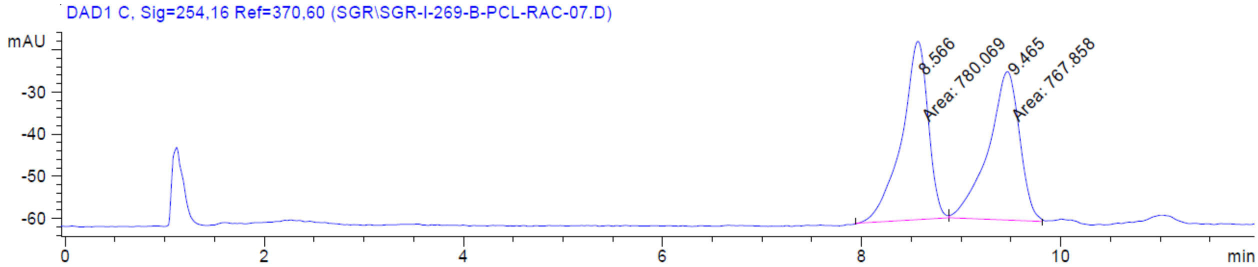

Signal 2: DAD1 C, Sig=254,16 Ref=370,60

Peak #	RetTime [min]	Type	Width [min]	Area [mAU*s]	Height [mAU]	Area %
1	8.566	MM	0.3074	780.06921	42.29282	50.3944
2	9.465	MM	0.3637	767.85803	35.18404	49.6056

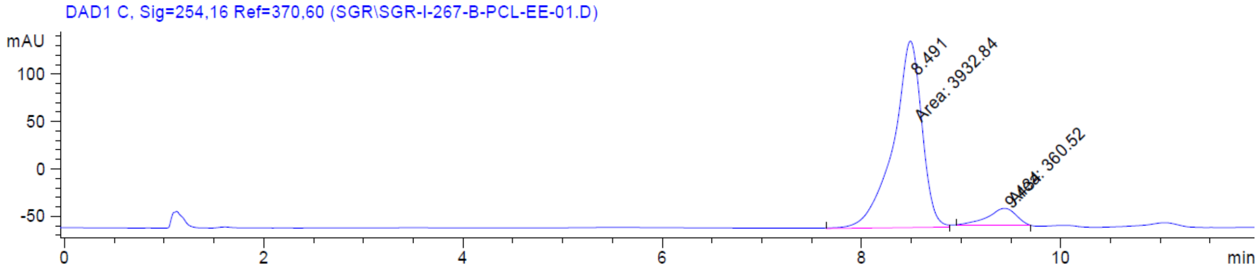

Signal 2: DAD1 C, Sig=254,16 Ref=370,60

Peak #	RetTime [min]	Type	Width [min]	Area [mAU*s]	Height [mAU]	Area %
1	8.491	MM	0.3327	3932.83862	197.04034	91.6028
2	9.434	MM	0.3316	360.51987	18.12246	8.3972

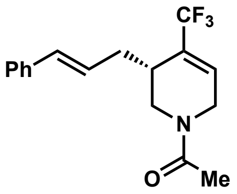

6d
Chemical Formula: $C_{17}H_{18}F_3NO$
Exact Mass: 309.1340
Molecular Weight: 309.3322

(R)-1-(3-cinnamyl-4-(trifluoromethyl)-3,6-dihydropyridin-1(2H)-yl)ethan-1-one 6d was synthesized following the general procedure B. Therefore, 4-(trifluoromethyl)pyridine (58 µL, 0.5 mmol, 1.0 equiv.) was used. The desired product was isolated as colorless oil (47.3 mg, 0.153 mmol, 31% yield over three steps).

^{1}H NMR (400 MHz, CD_3OD) (as mixture of E/Z amide bond isomers): δ 7.39 – 7.35 (m, 2H), 7.32 – 7.25 (m, 2H), 7.23 – 7.16 (m, 1H), 6.55 – 6.42 (m, 2H), 6.28 – 6.19 (m, 1H), 4.67 (dd, *J* = 13.2, 1.8 Hz, 0.5H),4.59 (dt, *J* = 20.7, 3.2 Hz, 0.5H), 4.34 (dt, *J* = 19.4, 3.3 Hz, 0.5H), 4.08 – 4.00 (m, 0.5H), 3.95 (dd, *J* = 13.5, 2.2 Hz, 0.5H), 3.75 – 3.66 (m, 0.5H), 3.30 – 3.18 (m, 0.5H), 2.83 – 2.73 (m, 1H), 2.66 – 2.58 (m, 1H), 2.49 (ddd, *J* = 13.8, 7.2, 2.5 Hz, 0.5H), 2.33 – 2.15 (m, 1H), 2.10 (s, 1.5H), 2.08 (s, 1.5H);

^{13}C NMR (101 MHz, CD_3OD) (as mixture of E/Z amide bond isomers, approx. 1/1 mixture, § = E/Z signal overlapping): δ 172.95, 172.71, 138.84, 138.45, 134.25, 134.16, 131.50 (q, *J* = 30.1 Hz), 130.76 (q, *J* = 30.1 Hz), 130.33 (q, *J* = 6.1 Hz), 129.99 (q, *J* = 6.2 Hz), 129.63, 129.51, 128.48, 128.21, 127.96, 127.41, 127.20, 127.17, 125.03 (q, *J* = 271.8 Hz), 124.97 (q, *J* = 271.6 Hz), 46.89, 45.92, 42.67, 41.88, 36.04§, 35.27§, 21.73, 21.34;

^{19}F NMR (282 MHz, CD_3OD) (as mixture of E/Z amide bond isomers, roughly 1/1 mixture): δ –67.86 (q, *J* = 2.7 Hz), – 67.98 (q, *J* = 3.0 Hz);

HRMS: m/z (ESI) calculated for $(M + H)^+$: 310.1419, found: 310.1417;

R_f (Hexane/EtOAc = 6/4): 0.20;

ATR-IR ν (cm^{-1}): 3674, 3026, 2922, 1734, 1652, 1490, 1423, 1373, 1337, 1296, 1261, 1241, 1206, 1162, 1115, 1072, 1019, 986, 836, 825, 742, 695, 676, 658;

$[\alpha]_D^{25}$: – 47.62 (c 1.0, $CHCl_3$);

Chiral SFC Separation: 5% MeOH, 2.5 mL/min, AS-H column, λ = 210 nm, t_R (min): major = 4.14 (area: 96.60%), minor = 5.22 (area: 3.40%), 93.2% enantiomeric excess.

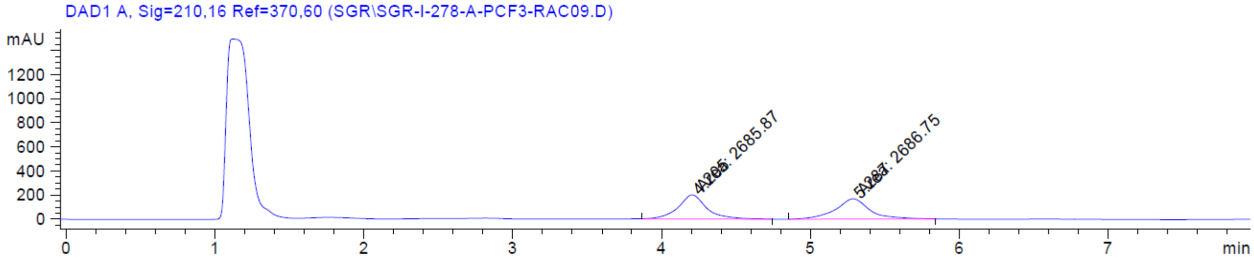

Signal 1: DAD1 A, Sig=210,16 Ref=370,60

Peak #	RetTime [min]	Type	Width [min]	Area [mAU*s]	Height [mAU]	Area %
1	4.205	MM	0.2237	2685.87476	200.10240	49.9918
2	5.287	MM	0.2657	2686.75220	168.53705	50.0082

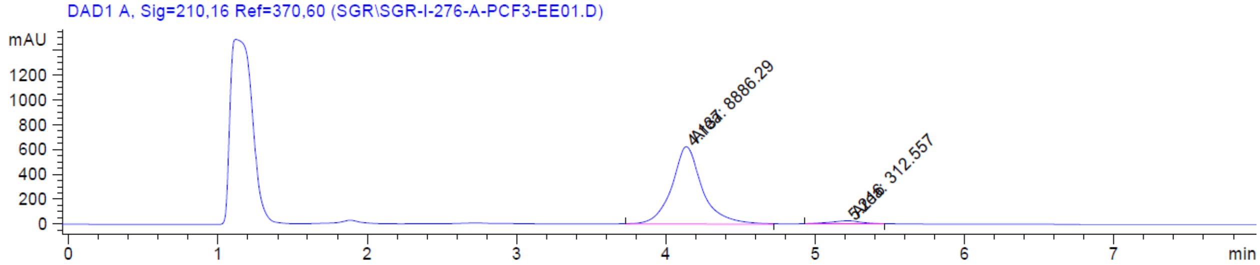

Signal 1: DAD1 A, Sig=210,16 Ref=370,60

Peak #	RetTime [min]	Type	Width [min]	Area [mAU*s]	Height [mAU]	Area %
1	4.137	MM	0.2372	8886.29492	624.26160	96.6022
2	5.216	MM	0.2225	312.55685	23.41279	3.3978

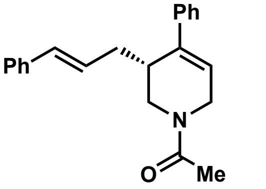

6e
Chemical Formula: $C_{22}H_{23}NO$
Exact Mass: 317.1780
Molecular Weight: 317.4320

(R)-1-(3-cinnamyl-4-phenyl-3,6-dihydropyridin-1(2H)-yl)ethan-1-one 6e was synthesized following the general procedure C or D. Therefore, 4-phenylpyridine (78.1 mg, 0.5 mmol, 1.0 equiv.) was used. The desired product was isolated as pale-yellow oil (38.3 mg, 0.120 mmol, 24% yield over three steps).

^{1}H NMR (400 MHz, CD_3OD) (as mixture of E/Z amide bond isomers): δ 7.45 – 7.40 (m, 2H), 7.38 – 7.32 (m, 2H), 7.30 – 7.20 (m, 5H), 7.18 – 7.11 (m, 1H), 6.37 (d, J = 15.8 Hz, 0.5H), 6.32 (d, J = 15.8 Hz, 0.5H), 6.21 – 6.12 (m, 1H), 6.02 (t, J = 3.4 Hz, 0.5H), 5.98 (t, J = 3.3 Hz, 0.5H), 4.62 (d, J = 12.6 Hz, 0.5H), 4.56 (dd, J = 19.7, 3.6 Hz, 0.5H), 4.27 (dd, J = 18.4, 3.9 Hz, 0.5H), 4.13 – 4.02 (m, 0.5H), 3.99 – 3.93 (m, 0.5H), 3.80 – 3.72 (m, 0.5H), 3.46 (dd, J = 13.3, 3.4 Hz, 0.5H), 3.14 – 3.08 (m, 0.5H), 3.06 – 2.95 (m, 1H), 2.40 – 2.14 (m, 2H), 2.12 (s, 1.5H), 2.10 (s, 1.5H);

^{13}C NMR (101 MHz, CD_3OD) (as mixture of E/Z amide bond isomers, roughly 1/1 mixture, § = E/Z signal overlapping): δ 172.64, 172.48, 141.71, 141.14, 141.04, 140.93, 139.03, 138.66, 133.43, 133.24, 129.67§, 129.53, 129.41, 129.12, 128.67, 128.61, 128.57, 128.23, 127.97, 127.09§, 127.07, 127.01, 121.92, 121.64, 47.82, 47.13, 43.69, 42.94, 38.72, 38.59, 36.34, 36.28, 21.74, 21.34;

HRMS: m/z (ESI) calculated for $(M + H)^+$: 318.1858, found: 318.1854;

R$_f$ (Hexane/EtOAc = 4/6): 0.30;

ATR-IR ν (cm^{-1}): 3216, 3056, 3027, 2929, 1633, 1494, 1435, 1370, 1331, 1265, 1075, 1035, 971, 882, 766, 740, 698;

$[\alpha]_D^{25}$: – 4.93 (c 0.85, $CHCl_3$);

Chiral SFC Separation: 20% iPrOH, 2.5 mL/min, AD-H column, λ = 254 nm, t_R (min): major = 6.680 (area: 93.0%), minor = 11.213 (area: 7.0%), 86.0% enantiomeric excess.

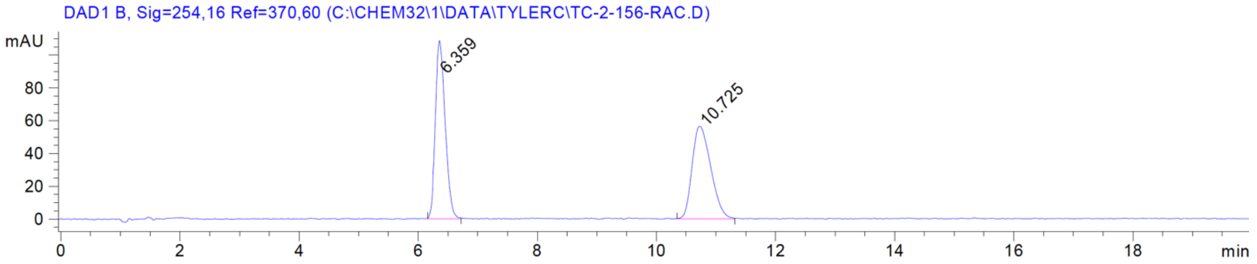

Signal 2: DAD1 B, Sig=254,16 Ref=370,60

Peak #	RetTime [min]	Type	Width [min]	Area [mAU*s]	Height [mAU]	Area %
1	6.359	BB	0.1777	1244.61230	108.63536	50.4051
2	10.725	BB	0.3412	1224.60437	56.42291	49.5949

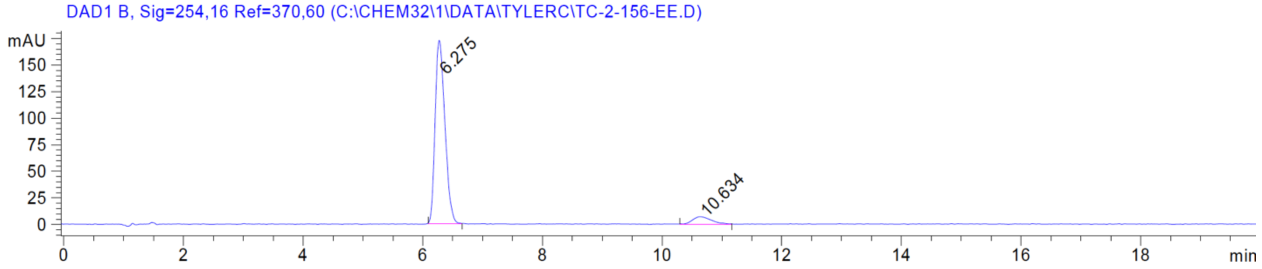

Signal 2: DAD1 B, Sig=254,16 Ref=370,60

Peak #	RetTime [min]	Type	Width [min]	Area [mAU*s]	Height [mAU]	Area %
1	6.275	BB	0.1763	1960.29517	172.92909	93.0373
2	10.634	BB	0.3147	146.70486	6.93692	6.9627

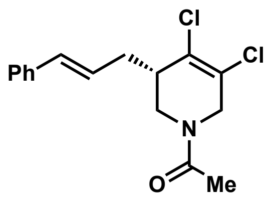

6f

Chemical Formula: $C_{16}H_{17}Cl_2NO$
Exact Mass: 309.0687
Molecular Weight: 310.2180

(S)-1-(4,5-dichloro-3-cinnamyl-3,6-dihydropyridin-1(2H)-yl)ethan-1-one 6f was synthesized following the general procedure B. Therefore, 3,4-dichloropyridine (74.0 mg, 0.5 mmol, 1.0 equiv.) was used. The desired product was isolated as colorless oil (55.0 mg, 0.177 mmol, 35% yield over three steps).

^{1}H NMR (400 MHz, CD_3OD) (as mixture of E/Z amide bond isomers): δ 7.39 – 7.35 (m, 2H), 7.32 – 7.26 (m, 2H), 7.24 – 7.17 (m, 1H), 6.57 – 6.46 (m, 1H), 6.26 – 6.17 (m, 1H), 4.57 – 4.51 (m, 0.5H), 4.42 (dd, *J* = 13.3, 2.0 Hz, 0.5H), 4.32 (dt, *J* = 17.0, 1.2 Hz, 0.5H), 4.16 (dd, *J* = 17.0, 1.9 Hz, 0.5H), 3.92 (dd, *J* = 17.9, 1.5 Hz, 0.5H), 3.82 (dd, *J* = 13.9, 3.2 Hz, 0.5H), 3.58 (dd, *J* = 13.9, 4.1 Hz, 0.5H), 3.18 (dd, *J* = 13.3, 4.2 Hz, 0.5H), 2.81 – 2.68

(m, 1H), 2.66 – 2.56 (m, 1H), 2.39 (dddd, J = 14.6, 9.3, 8.0, 1.1 Hz, 0.5H), 2.31 – 2.21 (m, 0.5H), 2.11 (s, 1.5H), 2.10 (s, 1.5H);

^{13}C NMR (101 MHz, CD_3OD) (as mixture of E/Z amide bond isomers, roughly 1/1 mixture, § = E/Z signal overlapping): δ 172.09, 171.97, 138.73, 138.40, 134.47, 134.46, 132.13, 131.04, 129.62, 129.52, 128.50, 128.27, 127.32, 127.19§, 127.01, 126.21, 125.45, 51.77, 48.17, 47.77, 44.88, 44.82, 42.82, 35.22§, 21.61, 21.16;

HRMS: m/z (ESI) calculated for $(M + H)^+$: 310.0765, found: 310.0756;

R_f (Hexane/EtOAc = 6/4): 0.37;

$[\alpha]_D^{25}$: – 18.23 (c 1.0, $CHCl_3$);

ATR-IR ν (cm^{-1}): 3853, 3734, 3648, 3025, 2919 1716, 1654, 1492, 1420, 1363, 1330, 1276, 1234, 1143, 1032, 985, 884, 825, 749, 695, 682;

Chiral SFC Separation: 10% MeOH, 2.5 mL/min, OD-H column, λ = 254 nm, t_R (min): major = 10.27 (area: 93.86%), minor = 11.57 (area: 6.14%), 87.7% enantiomeric excess.

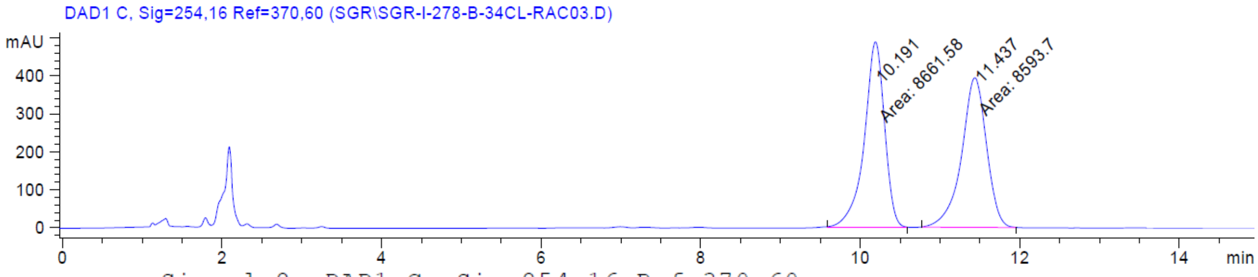

Signal 2: DAD1 C, Sig=254,16 Ref=370,60

Peak #	RetTime [min]	Type	Width [min]	Area [mAU*s]	Height [mAU]	Area %
1	10.191	MM	0.2949	8661.58203	489.44498	50.1967
2	11.437	MM	0.3629	8593.69727	394.65631	49.8033

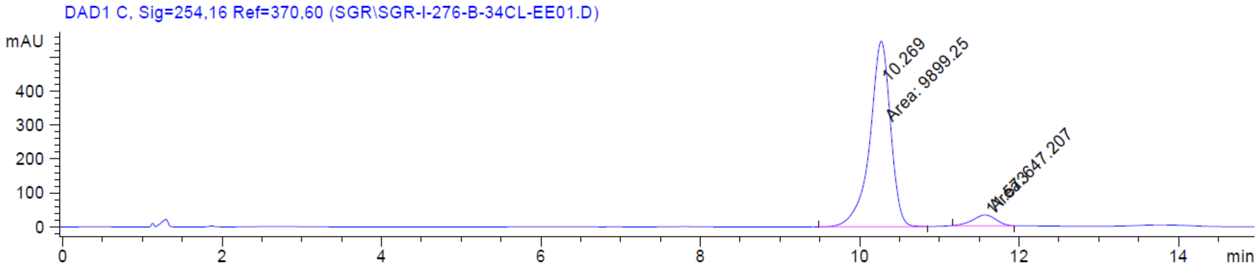

```
Signal 2: DAD1 C, Sig=254,16 Ref=370,60

Peak RetTime Type  Width     Area       Height      Area
  #   [min]        [min]    [mAU*s]     [mAU]        %
----|-------|----|-------|----------|----------|--------|
   1  10.269 MM    0.3012 9899.25098  547.71069  93.8633
   2  11.573 MM    0.3375  647.20721   31.95667   6.1367
```

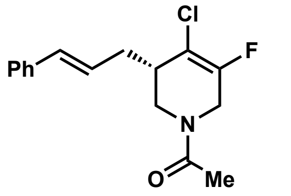

6g

Chemical Formula: $C_{16}H_{17}ClFNO$
Exact Mass: 293.0983
Molecular Weight: 293.7664

(S)-1-(4-chloro-3-cinnamyl-5-fluoro-3,6-dihydropyridin-1(2H)-yl)ethan-1-one 6g was synthesized following the general procedure B. Therefore, 4-chloro-3-fluoropyridine (65.8 mg, 0.5 mmol, 1.0 equiv.) was used. The desired product was isolated as colorless oil (72.2 mg, 0.246 mmol, 49% yield over three steps).

^{1}H NMR (400 MHz, CD_3OD) (as mixture of E/Z amide bond isomers): δ 7.39 – 7.33 (m, 2H), 7.32 – 7.24 (m, 2H), 7.23 – 7.15 (m, 1H), 6.57 – 6.44 (m, 1H), 6.27 – 6.16 (m, 1H), 4.49 – 4.41 (m, 0.6H), 4.34 – 4.24 (m, 0.8H), 4.15 – 4.07 (m, 0.4H), 3.93 – 3.85 (m, 0.6H), 3.74 (dd, *J* = 13.9, 3.3 Hz, 0.6H), 3.54 (dd, *J* = 13.8, 4.1 Hz, 0.6H), 3.19 (dd, *J* = 13.4, 4.1, 0.4H), 2.74 – 2.49 (m, 2H), 2.40 – 2.29 (m, 0.6H), 2.27 – 2.17 (m, 0.4H), 2.13 – 2.06 (m, 3H);

^{13}C NMR (101 MHz, CD_3OD) (as mixture of E/Z amide bond isomers, signals of minor isomer are indicated with *): δ 172.39, 172.35*, 152.61 (d, *J* = 257.2 Hz), 152.00 (d, *J* = 258.0 Hz)*, 138.73*, 138.42, 134.40*, 134.38, 129.62, 129.52*, 128.47, 128.25*, 127.40, 127.18[§], 127.08*, 113.29 (d, *J* = 11.2 Hz)*, 112.32 (d, *J* = 11.8 Hz), 48.03 46.24 (d, *J* = 37.0 Hz)*, 43.11*, 42.67 (d, *J* = 37.5 Hz), 42.04, 41.91*, 34.91, 34.89*, 21.62*, 21.16;

^{19}F NMR (282 MHz, CD_3OD) (as mixture of E/Z amide bond isomers, signals of minor isomer are indicated with *): δ –116.04 (d, *J* = 5.9 Hz), –116.99 (d, *J* = 5.8 Hz)*;

HRMS: m/z (ESI) calculated for $(M + H)^+$: 294.1061, found: 294.1045;

R_f (Hexane/EtOAc = 6/4): 0.21;

ATR-IR ν (cm^{-1}): 3363, 2931, 1716, 1652, 1435, 1372, 1236, 1036, 992, 734, 699;

$[\alpha]_D^{25}$: – 25.73 (c 1.0, $CHCl_3$);

Chiral SFC Separation: 10% iPrOH, 2.5 mL/min, OD-H column, λ = 210 nm, t_R (min): major = 12.46 (area: 98.14%), minor = 14.08 (area: 1.86%), 96.3% enantiomeric excess.

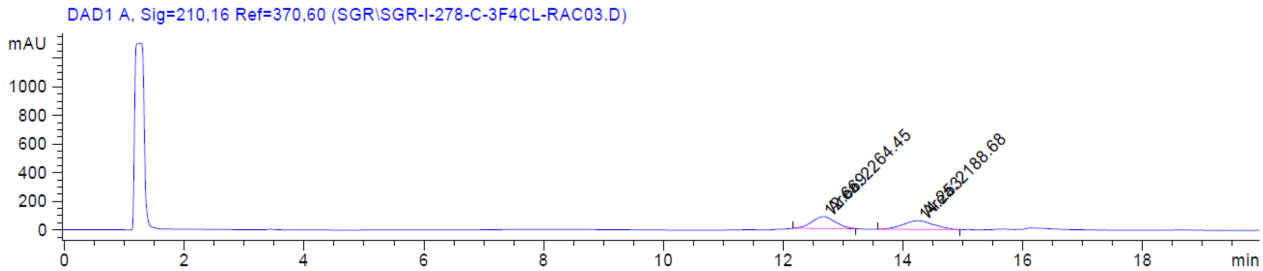

Signal 1: DAD1 A, Sig=210,16 Ref=370,60

Peak #	RetTime [min]	Type	Width [min]	Area [mAU*s]	Height [mAU]	Area %
1	12.669	MM	0.4542	2264.45215	83.09532	50.8508
2	14.253	MM	0.5970	2188.67920	61.09854	49.1492

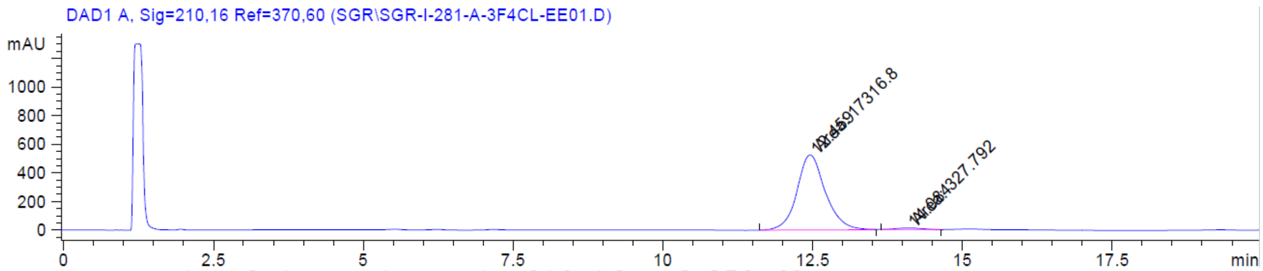

Signal 1: DAD1 A, Sig=210,16 Ref=370,60

Peak #	RetTime [min]	Type	Width [min]	Area [mAU*s]	Height [mAU]	Area %
1	12.459	MM	0.5493	1.73168e4	525.40576	98.1423
2	14.084	MM	0.4721	327.79208	11.57163	1.8577

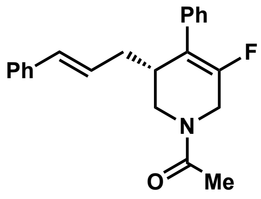

6h

Chemical Formula: $C_{22}H_{22}FNO$
Exact Mass: 335.1685
Molecular Weight: 335.4224

(R)-1-(3-cinnamyl-5-fluoro-4-phenyl-3,6-dihydropyridin-1(2H)-yl)ethan-1-one 6h was synthesized following the general procedure C or D. Therefore, 3-fluoro-4-phenylpyridine (86.6 mg, 0.5 mmol, 1.0 equiv.) was used. The desired product was isolated as colorless oil (34.7 mg, 0.103 mmol, 21% yield over three steps).

^{1}H NMR (400 MHz, CD_3OD) (as mixture of E/Z amide bond isomers): δ 7.43 – 7.35 (m, 4H), 7.32 – 7.21 (m, 5H), 7.19 – 7.12 (m, 1H), 6.42 – 6.29 (m, 1H), 6.16 – 6.07 (m, 1H), 4.62 (d, J = 18.1 Hz, 0.6H), 4.51 (dd, J = 13.2, 2.4 Hz, 0.4H), 4.39 (d, J = 17.0 Hz, 0.4H), 4.10 (dt, J = 16.9, 1.6 Hz, 0.4H), 3.90 (dd, J = 13.7, 2.8 Hz, 0.6H), 3.80 (d, J = 18.0 Hz, 0.6H), 3.55 (dd, J = 13.6, 3.8 Hz, 0.6H), 3.14 (dd, J = 13.2, 3.9 Hz, 0.4H), 3.10 – 3.02 (m, 0.6H), 2.97 – 2.89 (m, 0.4H), 2.37 – 2.29 (m, 0.6H), 2.25 – 2.18 (m, 1H), 2.17 – 2.13 (m, 3H), 2.11 – 2.05 (m, 0.4H);

^{13}C NMR (101 MHz, CD_3OD) (as mixture of E/Z amide bond isomers, signals of minor isomer are indicated with *, § = E/Z signal overlapping): δ 171.23, 171.16*, 150.92 (d, J = 254.8 Hz), 150.40 (d, J = 255.3 Hz)*, 137.52*, 137.18, 134.41*, 134.30, 132.32, 132.21*, 128.20*, 128.17,

128.13, 128.10§, 128.03*, 127.25*, 127.23, 126.89, 126.82*, 126.66§, 125.71*, 125.69, 117.69 (d, *J* = 5.4 Hz)*, 117.05 (d, *J* = 6.0 Hz), 46.55, 44.44 (d, *J* = 41.8 Hz)*, 41.62*, 41.00 (d, *J* = 42.1 Hz), 38.11 (d, *J* = 3.7 Hz), 37.78 (d, *J* = 3.4 Hz)*, 34.80 (d, *J* = 3.0 Hz)§, 20.36*, 19.92;

^{19}F NMR (282 MHz, CD_3OD) (as mixture of E/Z amide bond isomers, signals of minor isomer are indicated with *): δ –119.33 (d, *J* = 5.6 Hz), –120.31 (d, *J* = 5.7 Hz)*;

HRMS: m/z (ESI) calculated for $(M + H)^+$: 336.1764, found: 336.1774;

R_f (Hexane/EtOAc = 6/4): 0.19;

ATR-IR ν (cm^{-1}): 3024, 2930, 1734, 1652, 1496, 1426, 1373, 1242, 1205, 1180, 1051, 1009, 970, 820, 749, 698;

$[\alpha]_D^{25}$: – 67.87 (c 1.0, $CHCl_3$);

Chiral SFC Separation: 30% MeOH, 2.5 mL/min, OJ-H column, λ = 254 nm, t_R (min): major = 4.596 (area: 96.3%), minor = 5.677 (area: 3.7%), 92.6% enantiomeric excess.

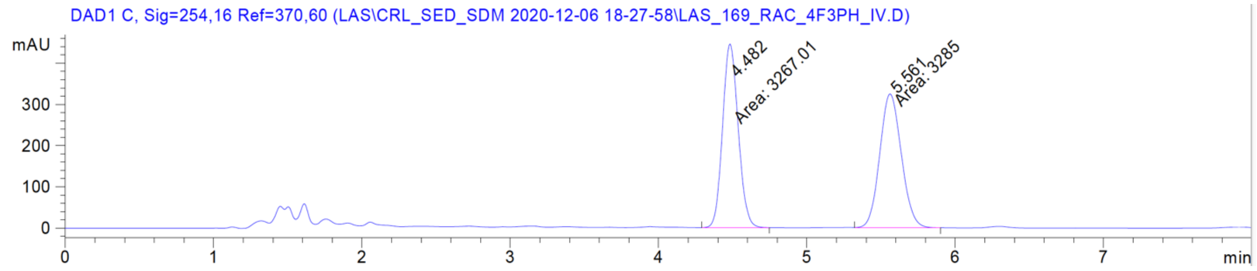

Signal 2: DAD1 C, Sig=254,16 Ref=370,60

Peak #	RetTime [min]	Type	Width [min]	Area [mAU*s]	Height [mAU]	Area %
1	4.482	MM	0.1217	3267.01074	447.36011	49.8627
2	5.561	MM	0.1682	3285.00098	325.46252	50.1373

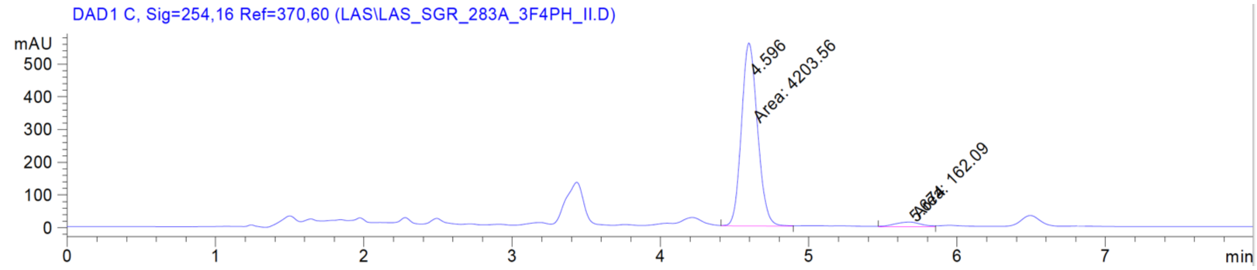

Signal 2: DAD1 C, Sig=254,16 Ref=370,60

Peak #	RetTime [min]	Type	Width [min]	Area [mAU*s]	Height [mAU]	Area %
1	4.596	MM	0.1251	4203.55566	559.99762	96.2872
2	5.674	MM	0.1996	162.08955	13.53266	3.7128

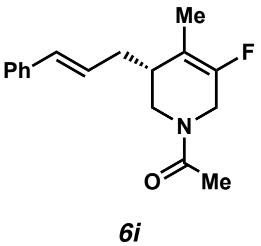

6i

Chemical Formula: $C_{17}H_{20}FNO$
Exact Mass: 273.1529
Molecular Weight: 273.3514

(R)-1-(3-cinnamyl-5-fluoro-4-methyl-3,6-dihydropyridin-1(2H)-yl)ethan-1-one 6i was synthesized following the general procedure D. Therefore, 3-fluoro-4-methylpyridine (52 µL, 0.5 mmol, 1.0 equiv.) was used. The desired product was isolated as pale-yellow oil (60.2 mg, 0.22 mmol, 44% yield over three steps).
Note: Using 2 mol% $[Ir(coe)Cl]_2$ and 10 mol% $Pd(OAc)_2$ delivers product in a higher yield, but lower enantioselectivity (76.6 mg, 0.28 mmol, 56% yield, 77% ee).

^{1}H NMR (400 MHz, $CDCl_3$) (as mixture of E/Z amide bond isomers): δ 7.39 – 7.29 (m, 4H), 7.26 – 7.17 (m, 1H), 6.46 (ddd, *J* = 15.8, 3.8, 2.3 Hz, 1H), 6.28 – 6.07 (m, 1H), 4.54 – 4.38 (m, 0.65H), 4.20 (dd, *J* = 13.2, 3.3 Hz, 0.35H), 4.00 (dt, *J* = 15.9, 2.0 Hz, 0.35H), 3.90 (dtd, *J* = 16.1, 2.0, 1.1 Hz, 0.35H), 3.77 – 3.66 (m, 0.65H), 3.62 (dd, *J* = 13.4, 3.0 Hz, 0.65H), 3.30 (ddd, *J* = 13.3, 3.9, 1.0 Hz, 0.65H), 3.11 (dd, *J* = 13.2, 4.0 Hz, 0.35H), 2.45 – 2.29 (m, 0.7H), 2.23 – 2.14 (overlap, 2.3H), 2.10 (s, 2H), 2.08 (s, 1H), 1.75 (dd, *J* = 4.5, 2.3 Hz, 3H);

^{13}C NMR (101 MHz, $CDCl_3$) (as mixture of E/Z amide bond isomers, signals of minor isomer are indicated with *) δ 170.2, 169.8*, 150.5 (d, *J* = 248.7 Hz), 149.3 (d, *J* = 248.7 Hz)*, 137.6*, 137.0, 132.9, 132.6*, 128.8, 128.6*, 127.8, 127.6, 127.7*, 127.2*, 126.3*, 126.2, 112.7 (d, *J* = 8.4 Hz)*, 111.1 (d, *J* = 9.5 Hz), 46.4, 44.8 (d, *J* = 41.0 Hz)*, 41.9*, 41.8 (d, *J* = 41.8 Hz), 39.5 (d, *J* = 4.8 Hz), 38.8 (d, *J* = 4.4 Hz)*, 34.5 (d, *J* = 2.7 Hz)*, 34.4 (d, *J* = 3.1 Hz), 22.0*, 21.5, 12.4 (dd, *J* = 6.2, 1.8 Hz);

^{19}F NMR (282 MHz, $CDCl_3$) (as mixture of E/Z amide bond isomers, signals of minor isomer are indicated with *): δ -120.71 – -120.73 (m), -122.01 – -122.04 (m)*;

HRMS: m/z (ESI) calculated for $(M + H)^+$: 274.1602, found 274.1605;

R_f (Hexane/EtOAc) = 4/6): 0.65;

$[\alpha]_D^{25}$: – 8.75 (c 1.0, $CHCl_3$);

ATR-IR ν (cm^{-1}): 3022, 2918, 1728, 1648, 1438, 1387, 1369, 1234, 1158, 1070, 969, 918, 750, 724, 693;

Chiral SFC Separation: 25% iPrOH, 2.5 mL/min, AD-H column, λ = 254 nm or 280 nm, t_R (min): major = 2.596 (area: 96.8%), minor = 3.846 (area: 3.2%), 93.6% enantiomeric excess.

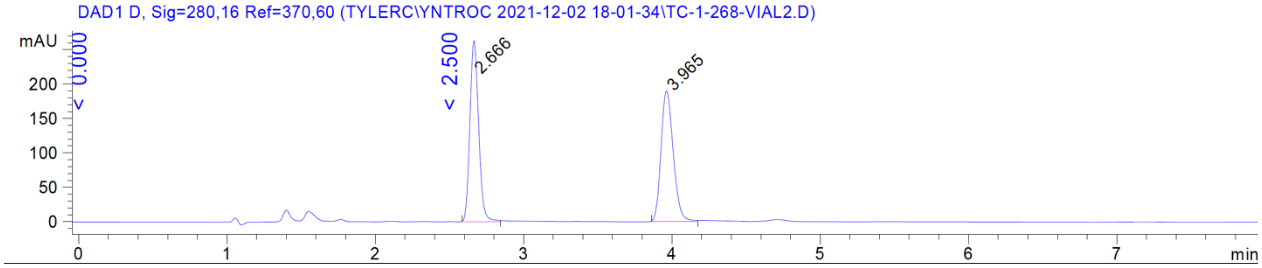

```
Signal 3: DAD1 D, Sig=280,16 Ref=370,60

Peak RetTime Type  Width     Area       Height     Area
  #   [min]        [min]   [mAU*s]      [mAU]        %
----|-------|----|-------|----------|----------|--------|
   1   2.666 BB    0.0644 1081.70117  260.62692  49.7744
   2   3.965 BB    0.0910 1091.50793  190.15250  50.2256
```

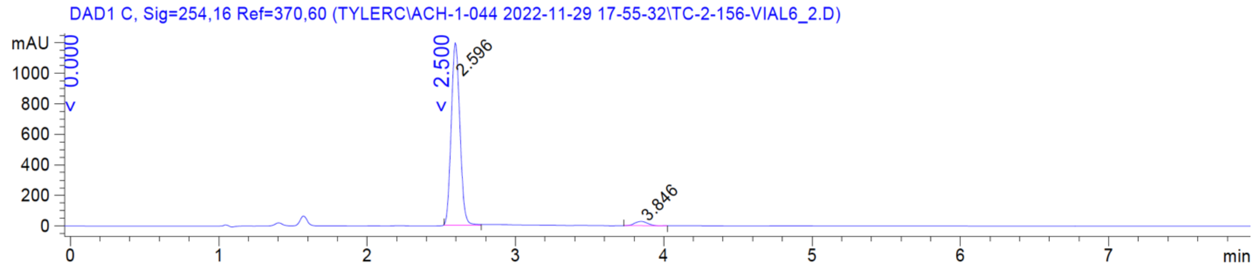


```
Signal 2: DAD1 C, Sig=254,16 Ref=370,60

Peak RetTime Type  Width     Area       Height     Area
  #   [min]        [min]   [mAU*s]      [mAU]        %
----|-------|----|-------|----------|----------|--------|
   1   2.596 BB    0.0678 4862.83008 1186.93994  96.7838
   2   3.846 BB    0.0895  161.59549   28.79920   3.2162
```

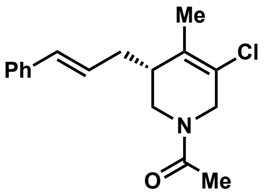

6j

Chemical Formula: $C_{17}H_{20}ClNO$
Exact Mass: 289.1233
Molecular Weight: 289.8030

(R)-1-(5-chloro-3-cinnamyl-4-methyl-3,6-dihydropyridin-1(2H)-yl)ethan-1-one 6j was synthesized following the general procedure D. Therefore, 3-chloro-4-methylpyridine (55 µL, 0.5 mmol, 1.0 equiv.) was used. The desired product was isolated as pale-yellow oil (68.2 mg, 0.235 mmol, 47% yield over three steps).
Note: Using 2 mol% $[Ir(coe)Cl]_2$ and 10 mol% $Pd(OAc)_2$ delivers product in a higher yield, but lower enantioselectivity (108.8 mg, 0.375 mmol, 75% yield, 72% ee).

^{1}H NMR (400 MHz, $CDCl_3$) (as mixture of E/Z amide bond isomers): δ 7.41 – 7.27 (m, 4H), 7.27 – 7.13 (m, 1H), 6.46 (dt, *J* = 15.7, 1.5 Hz, 1H), 6.28 – 6.08 (m, 1H), 4.57 (dt, *J* = 18.0, 1.9 Hz, 0.65H), 4.35 (dd, *J* = 13.2, 2.9 Hz, 0.35H), 4.07 (dt, *J* = 16.6, 1.9 Hz, 0.35H), 3.96 (dt, *J* = 16.6, 2.0 Hz, 0.35H), 3.81 – 3.64 (m, 1.3H), 3.40 – 3.26 (m, 0.65H), 3.04 (dd, *J* = 13.2, 4.0 Hz, 0.35H), 2.56 – 2.47 (m, 0.75H), 2.41 – 2.24 (m, 1.35H), 2.23 – 2.15 (m, 1H), 2.10 (s, 3H), 1.97 – 1.87 (m, 3H);

^{13}C NMR (101 MHz, $CDCl_3$) (as mixture of E/Z amide bond isomers, signals of minor isomer are indicated with *) δ 169.89, 169.43*, 137.51*, 136.96, 133.05, 132.80*, 132.69*, 130.79, 128.79, 128.59*, 127.67, 127.61, 127.24*, 127.16*, 126.26*, 126.13, 123.49, 121.87*, 50.54*, 46.89, 46.01, 42.22, 41.94*, 41.52*, 34.60*, 34.33, 21.94*, 21.43, 18.38*, 18.33;

HRMS: m/z (ESI) calculated for $(M + H)^+$: 290.1306, found 290.1317;

R_f (Hexane/EtOAc = 4/6) : 0.60;

ATR-IR ν (cm^{-1}): 3236, 3027, 2924, 1644, 1434, 1369, 1241, 1014, 972, 894, 749, 694, 655;

$[\alpha]_D^{25}$: – 9.97 (c 1.0, $CHCl_3$);

Chiral SFC Separation: 15% iPrOH, 2.5 mL/min, AD-H column, λ = 254 nm, t_R (min): major = 6.689 (area: 96.5%), minor = 14.926 (area: 3.5%), 93.0% enantiomeric excess.

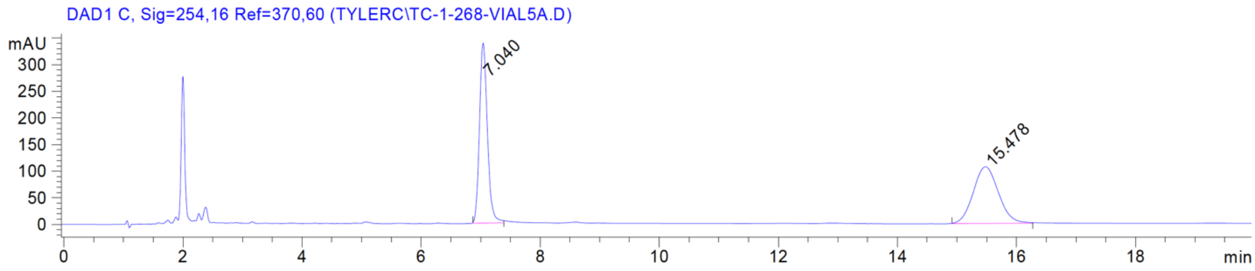

Signal 2: DAD1 C, Sig=254,16 Ref=370,60

Peak #	RetTime [min]	Type	Width [min]	Area [mAU*s]	Height [mAU]	Area %
1	7.040	BB	0.1443	3134.57324	337.63516	49.9836
2	15.478	BB	0.4555	3136.62598	106.54130	50.0164

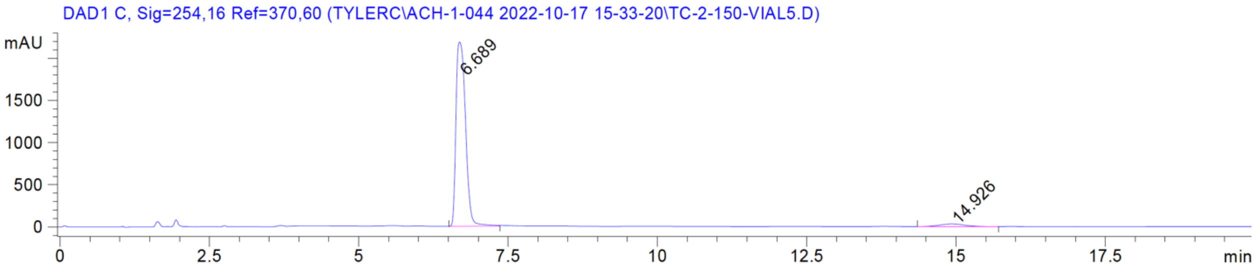

Signal 2: DAD1 C, Sig=254,16 Ref=370,60

Peak #	RetTime [min]	Type	Width [min]	Area [mAU*s]	Height [mAU]	Area %
1	6.689	BB	0.1781	2.43808e4	2186.42725	96.5063
2	14.926	BB	0.4392	882.61859	31.47048	3.4937

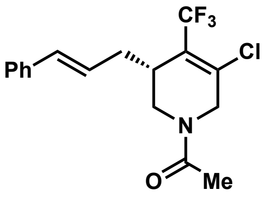

6k

Chemical Formula: $C_{17}H_{17}ClF_3NO$
Exact Mass: 343.0951
Molecular Weight: 343.7742

(R)-1-(5-chloro-3-cinnamyl-4-(trifluoromethyl)-3,6-dihydropyridin-1(2H)-yl)ethan-1-one 6k was synthesized following the general procedure B. Therefore, 3-chloro-4-(trifluoromethyl)pyridine (90.8 mg, 0.5 mmol, 1.0 equiv.) was used. The desired product was isolated as colorless oil (33.9 mg, 0.099 mmol, 20% yield over three steps).

^{1}H NMR (400 MHz, CD_3OD) (as mixture of E/Z amide bond isomers): δ 7.40 – 7.35 (m, 2H), 7.32 – 7.26 (m, 2H), 7.24 – 7.16 (m, 1H), 6.55 – 6.43 (m, 1H), 6.27 – 6.18 (m, 1H), 4.80 – 4.69 (m, 1H), 4.51 – 4.43 (m, 0.5H), 4.25 – 4.16 (m, 0.5H), 4.01 – 3.95 (m, 0.5H), 3.84 – 3.75 (m, 0.5H),

3.37 – 3.32 (m, 0.5H), 2.95 – 2.89 (m, 0.5H), 2.83 – 2.72 (m, 1H), 2.57 – 2.49 (m, 0.5H), 2.44 – 2.36 (m, 0.5H), 2.35 – 2.25 (m, 0.5H), 2.21 – 2.15 (m, 0.5H), 2.13 (s, 1.5H), 2.11 (s, 1.5H);

^{13}C NMR (101 MHz, CD_3OD) (as mixture of E/Z amide bond isomers, roughly 1/1 mixture): δ 172.45, 172.23, 138.71, 138.32, 135.74, (q, *J* = 3.9 Hz), 135.13 (q, *J* = 4.0 Hz), 134.64§, 129.65, 129.53, 129.39, 129.09, 128.58, 128.33, 127.29, 127.22, 127.21, 126.91, 124.04 (q, *J* = 274.1 Hz), 123.97 (q, *J* = 274.0 Hz), 51.72, 48.51, 46.36, 41.26, 38.18, 38.16, 36.19, 36.14, 21.68, 21.24;

^{19}F NMR (282 MHz, CD_3OD) (as mixture of E/Z amide bond isomers, roughly 1/1 mixture): δ -62.48, -62.63;

HRMS: m/z (ESI) calculated for $(M + H)^+$: 344.1029, found: 344.1021;

R_f (Hexane/EtOAc = 6/4): 0.18;

ATR-IR ν (cm^{-1}): 3026, 2927, 1735, 1653, 1493, 1423, 1364, 1287, 1262, 1242, 1210, 1185, 1132, 1052, 985, 887, 772, 744, 694, 649, 610;

$[\alpha]_D^{25}$: – 7.31 (c 1.0, $CHCl_3$);

Chiral SFC Separation: 10% MeOH, 2.5 mL/min, OJ-H column, λ = 254 nm, t_R (min): major = 3.834 (area: 98.0%), minor = 4.990 (area: 2.0%), 96.0% enantiomeric excess.

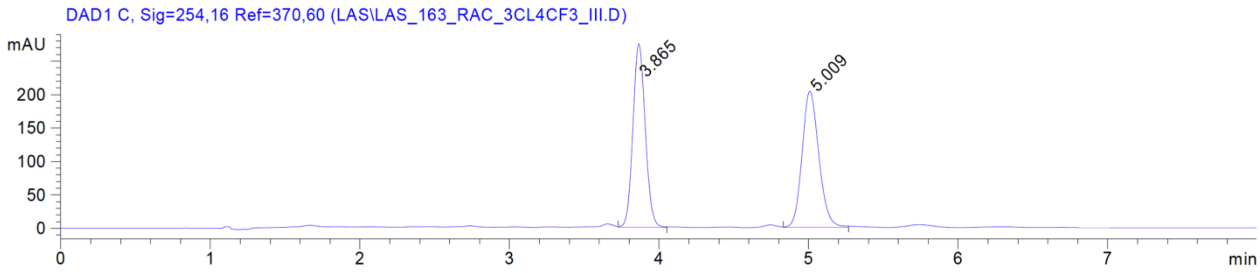

Signal 2: DAD1 C, Sig=254,16 Ref=370,60

Peak #	RetTime [min]	Type	Width [min]	Area [mAU*s]	Height [mAU]	Area %
1	3.865	VB	0.0869	1570.85596	273.97107	49.6514
2	5.009	VB	0.1194	1592.91370	203.42432	50.3486

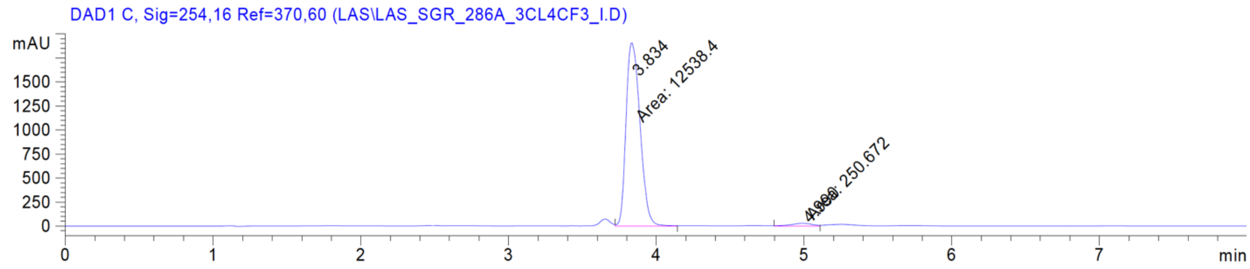

```
Signal 2: DAD1 C, Sig=254,16 Ref=370,60

Peak RetTime Type  Width     Area       Height      Area
  #   [min]        [min]    [mAU*s]     [mAU]        %
----|-------|----|-------|----------|----------|--------|
   1   3.834 MM    0.1089 1.25384e4  1919.23767  98.0400
   2   4.990 MM    0.1466  250.67191   28.49819   1.9600
```

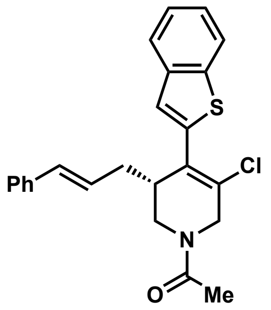

6l
Chemical Formula: $C_{24}H_{22}ClNOS$
Exact Mass: 407.1111
Molecular Weight: 407.9560

(R)-1-(4-(benzo[b]thiophen-2-yl)-5-chloro-3-cinnamyl-3,6-dihydropyridin-1(2H)-yl)ethan-1-one 6l was synthesized following the general procedure C or D. Therefore, 4-(benzo[b]thiophen-2-yl)-3-chloropyridine (122.9 mg, 0.5 mmol, 1.0 equiv.) was used. The desired product was isolated as yellow oil (103.8 mg, 0.255 mmol, 51% yield over three steps).
Note: Using 2 mol% $[Ir(coe)Cl]_2$ and 10 mol% $Pd(OAc)_2$ delivers product in a higher yield, but lower enantioselectivity (113.8 mg, 0.28 mmol, 56% yield, 80% ee).

^{1}H NMR (400 MHz, CD_3OD) (as mixture of E/Z amide bond isomers signals of minor isomer are indicated with *, § = two signals overlapping): δ 7.81 – 7.73 (m, 2H), 7.51 (s, 0.4H)*, 7.50 (s, 0.6H), 7.35 – 7.27 (m, 2H), 7.24 – 7.09 (m, 5H), 6.33 (d, J = 7.6 Hz, 0.4H)*, 6.29 (d, J = 7.6 Hz, 0.6H), 6.16 – 6.04 (m, 1H), 4.74 (d, J = 19.0 Hz, 0.6H), 4.59 (d, J = 11.8 Hz, 0.4H)*, 4.40 (d, J = 17.9 Hz, 0.4H)*, 4.11 (dd, J = 17.9, 1.2 Hz, 0.4H)*, 3.86 – 3.75 (m, 1.2H)§, 3.38 (dd, J = 13.5, 3.4, 0.6H), 3.05 – 2.98 (m, 0.6H), 2.96 – 2.86 (m, 0.8H)*§, 2.40 – 2.12 (m, 2H), 2.08 (s, 1.2H)*, 2.05 (s, 1.8H);

^{13}C NMR (101 MHz, CD_3OD) (as mixture of E/Z amide bond isomers, signals of minor isomer are indicated with *, § = E/Z signal overlapping): δ 172.13, 171.95*, 141.10§, 140.57§, 140.53, 140.49§, 140.34*, 138.76*, 138.37, 133.93, 133.91*, 132.12*, 131.29, 129.54, 129.43*, 128.36, 128.21, 128.11*, 127.84*, 127.15*, 127.12, 126.50§, 126.05§, 125.72*, 125.63, 124.94*, 124.92, 122.95§, 52.00*, 48.71, 46.94, 43.48*, 43.45, 42.06*, 36.32*, 36.27, 21.68*, 21.26;
HRMS: m/z (ESI) calculated for $(M + H)^+$: 408.1189, found: 408.1208;

R_f (Hexane/EtOAc = 1/1): 0.26;

ATR-IR ν (cm^{-1}): 3055, 3025, 2926, 1652, 1495, 1435, 1359, 1305, 1238, 1157, 1129, 1068, 1030, 968, 922, 860, 832, 745, 728, 695, 613;

$[\alpha]_D^{25}$: – 81.02 (c 1.0, $CHCl_3$);

Chiral SFC Separation: 30% MeOH, 2.5 mL/min, IC column, λ = 254 nm, t_R (min): major = 12.233 (area: 97.3%), minor = 11.410 (area: 2.7%), 94.6% enantiomeric excess.

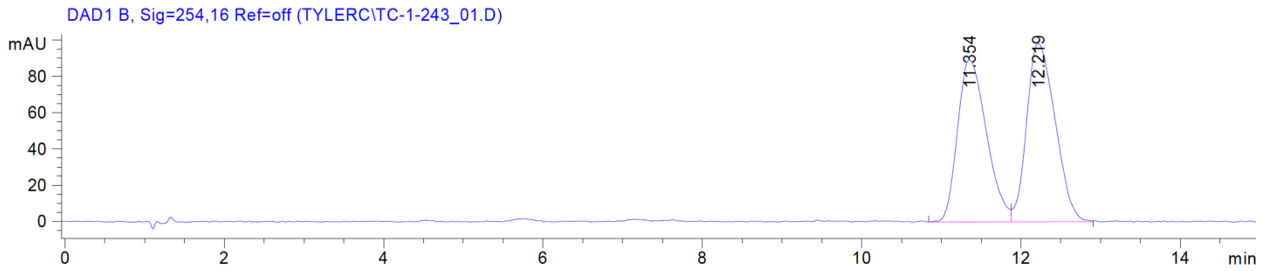

Signal 2: DAD1 B, Sig=254,16 Ref=off

Peak #	RetTime [min]	Type	Width [min]	Area [mAU*s]	Height [mAU]	Area %
1	11.354	VV	0.3963	2260.10962	89.58550	48.0283
2	12.219	VB	0.3680	2445.68018	98.39331	51.9717

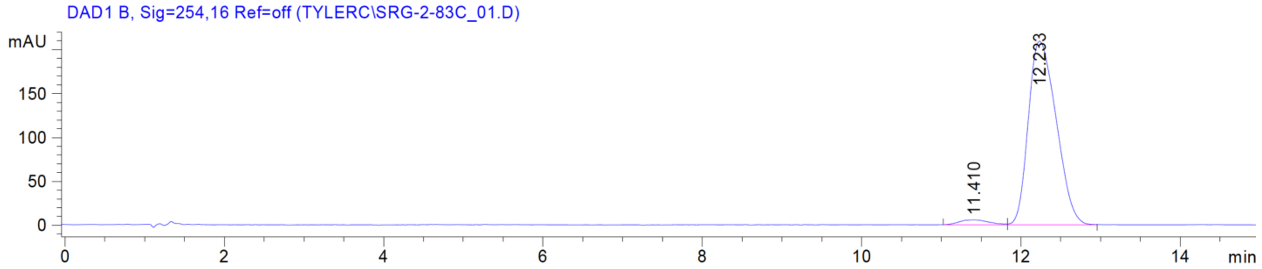

Signal 2: DAD1 B, Sig=254,16 Ref=off

Peak #	RetTime [min]	Type	Width [min]	Area [mAU*s]	Height [mAU]	Area %
1	11.410	BV	0.3061	138.32794	5.48661	2.6563
2	12.233	VB	0.3909	5069.24756	209.07002	97.3437

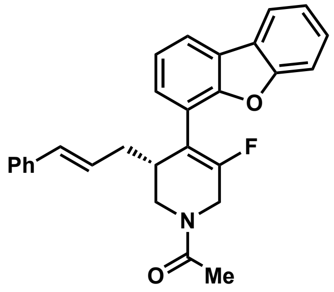

6m

Chemical Formula: $C_{28}H_{24}FNO_2$
Exact Mass: 425.1791
Molecular Weight: 425.5034

(*R*)-1-(3-cinnamyl-4-(dibenzo[*b*,*d*]furan-4-yl)-5-fluoro-3,6-dihydropyridin-1(2*H*)-yl)ethan-1-one 6m was synthesized following the general procedure C or D. Therefore, 4-(dibenzo[b,d]furan-4-yl)-3-fluoropyridine (131.6 mg, 0.5 mmol, 1.0 equiv.) was used. The desired product was isolated as yellow amorphous solid (89.2 mg, 0.21 mmol, 42% yield over three steps). Note: Using 2 mol% $[Ir(coe)Cl]_2$ and 10 mol% $Pd(OAc)_2$ delivers product in a higher yield, but lower enantioselectivity (114.7 mg, 0.27 mmol, 54% yield, 78% ee, 68% yield BRSM).

^{1}H NMR (400 MHz, CD_3OD) (as mixture of E/Z amide bond isomers): δ 8.01 – 7.96 (m, 1H), 7.93 (td, *J* = 5.2, 3.7 Hz, 1H), 7.61 (dt, *J* = 8.2, 0.9 Hz, 1H), 7.49 (ddd, *J* = 8.4, 7.3, 1.4 Hz, 1H), 7.43 – 7.32 (m, 3H), 7.28 – 7.12 (m, 5H), 6.33 (d, *J* = 15.9 Hz, 0.65H), 6.27 (d, *J* = 15.8 Hz, 0.35H), 6.10 (t, *J* = 7.6 Hz, 0.35H), 6.01 (ddd, *J* = 15.8, 8.5, 5.8 Hz, 0.65H), 4.80 (dd, *J* = 18.3, 1.6 Hz, 0.65H), 4.37 – 4.26 (m, 0.70H), 4.14 (dd, *J* = 16.8, 2.0 Hz, 0.35H), 3.99 (dd, *J* = 18.3, 1.8 Hz, 0.65H), 3.84 (dd, *J* = 13.4, 3.4 Hz, 0.65H), 3.69 (dd, *J* = 13.4, 4.0 Hz, 0.65H), 3.58 (dd, *J* = 13.3, 4.3 Hz, 0.35H), 3.32 – 3.20 (m, 1H), 2.42 – 2.32 (m, 0.70H), 2.32 – 2.23 (m, 1.3H), 2.21 (d, *J* = 4.9 Hz, 3H);

^{13}C NMR (101 MHz, CD_3OD) (as mixture of E/Z amide bond isomers, signals of minor isomer are indicated with *) δ 170.18, 169.74 (d, *J* = 1.7 Hz)*, 156.14*, 156.11, 153.62 (overlap)*, 152.52 (d, *J* = 258.3 Hz), 151.19 (d, *J* = 258.2 Hz)*, 137.41*, 136.91, 132.77, 132.51*, 128.59, 128.41*, 128.03, 128.00, 127.72*, 127.69*, 127.52*, 127.43, 127.03*, 126.87*, 126.10*, 126.01, 124.76, 124.19, 122.97 (overlap)*, 122.88 (overlap)*, 120.82, 120.38*, 120.35*, 118.85 (d, *J* = 1.9 Hz), 114.34 (d, *J* = 6.7 Hz)*, 113.02 (d, *J* = 7.7 Hz), 111.85 (overlap)*, 46.76, 45.24 (d, *J* = 39.7 Hz)*, 42.33*, 41.70 (d, *J* = 40.5 Hz), 38.35 (d, *J* = 3.0 Hz), 37.54 (d, *J* = 2.8 Hz)*, 35.26 (d, *J* = 2.8 Hz)*, 35.16 (d, *J* = 3.0 Hz), 22.08*, 21.53;

^{19}F NMR (282 MHz, CD_3OD) (as mixture of E/Z amide bond isomers, signals of minor isomer are indicated with *): δ -112.15 – -112.26 (m), -113.31 (d, *J* = 4.7 Hz)*;

HRMS: m/z (ESI) calculated for $(M + H)^+$: 426.1864, found 426.1872;

R_f (Hexane/acetone = 3/7): 0.40;

ATR-IR ν (cm^{-1}): 3439, 3027, 2930, 1648, 1450, 1414, 1378, 1352, 1257, 1233, 1180, 1046, 1012, 983, 922, 843, 798, 752, 737;

$[\alpha]_D^{25}$: – 17.72 (c 0.304, $CHCl_3$);

Chiral SFC Separation: 30% MeOH, 2.5 mL/min, AD-H column, λ = 254 nm, t_R (min): major = 6.303 (area: 98.5%), minor = 5.431 (area: 1.5%), 97.0% enantiomeric excess.

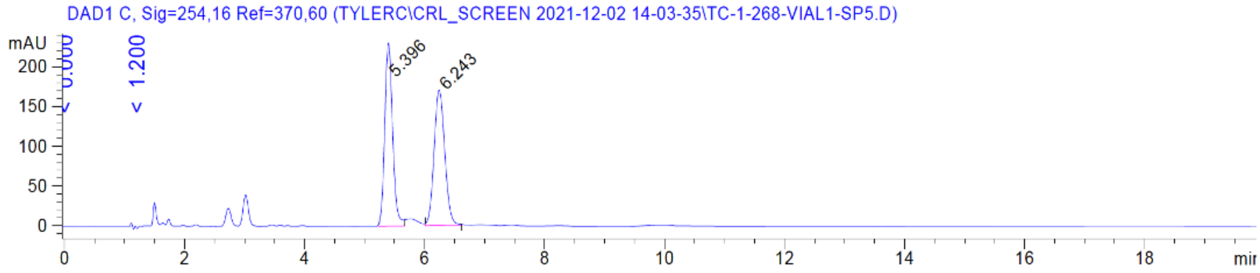

Signal 2: DAD1 C, Sig=254,16 Ref=370,60

Peak #	RetTime [min]	Type	Width [min]	Area [mAU*s]	Height [mAU]	Area %
1	5.396	BB	0.1392	2114.76562	230.07974	50.1048
2	6.243	BB	0.1920	2105.91846	170.63986	49.8952

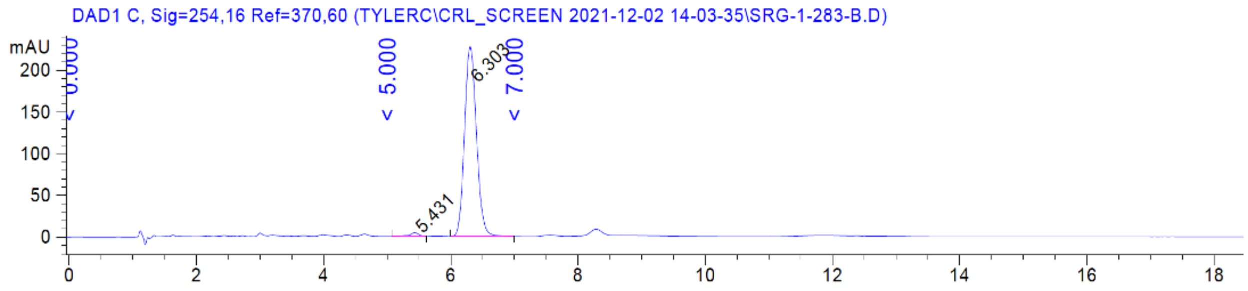


```
Signal 2: DAD1 C, Sig=254,16 Ref=370,60

Peak RetTime Type  Width     Area      Height     Area
  #   [min]        [min]   [mAU*s]     [mAU]       %
----|-------|----|-------|----------|----------|--------|
   1   5.431 BV    0.1582   44.71070    4.19887   1.4915
   2   6.303 VB    0.2013 2953.04980  227.77563  98.5085
```

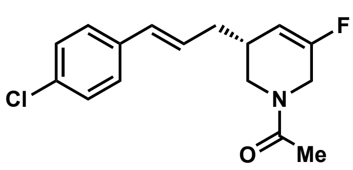

6n

Chemical Formula: $C_{16}H_{17}ClFNO$
Exact Mass: 293.0983
Molecular Weight: 293.7664

(*R*,*E*)-1-(3-(3-(4-chlorophenyl)allyl)-5-fluoro-3,6-dihydropyridin-1(2H)-yl)ethan-1-one 6n was synthesized following the general procedure B using 3-fluoropyridine (43 µL, 0.5 mmol, 1.0 equiv.) as substrate and (*E*)-3-(4-chlorophenyl)allyl methyl carbonate (170 mg, 0.75 mmol, 1.5 equiv.). The desired compound was obtained as yellowish oil (42 mg, 0.143 mmol, 29% yield over three steps).

^{1}H NMR (400 MHz, $CDCl_3$) (as mixture of E/Z amide bond isomers): δ 7.30–7.23 (m, 4H), 6.44–6.34 (m, 1H), 6.18–6.06 (m, 1H), 5.46–5.30 (m, 1H), 4.18–4.04 (m, 1H), 4.02–3.91 (m, 1H), 3.84 (dd, *J* = 13.1, 4.7 Hz, 0.4H), 3.53 (dd, *J* = 13.4, 4.5 Hz, 0.6H), 3.36–3.21 (m, 1H), 2.57–2.42 (m, 1H), 2.32–2.15 (m, 2H), 2.11 (s, 3H);

^{13}C NMR (101 MHz, $CDCl_3$) (as mixture of E/Z amide bond isomers, signals of minor isomer are indicated with *): δ 169.8, 169.7*, 156.4 (d, *J* = 257 Hz), 154.9 (d, *J* = 256 Hz)*, 135.8*, 135.5, 133.2, 132.9*, 131.8, 131.6*, 128.9, 128.7*, 127.4*, 127.4, 127.4, 106.0 (d, *J* = 11.0 Hz)*, 104.3 (d, *J* = 12.5 Hz), 47.7, 44.8 (d, *J* = 39 Hz)*, 42.9*, 41.1 (d, *J* = 40 Hz), 36.8 (d, *J* = 2.2 Hz)*, 36.7 (d, *J* = 2.6 Hz), 34.2 (d, *J* = 6.2 Hz), 33.3 (d, *J* = 5.9 Hz)*, 22.0*, 21.5;

^{19}F NMR (282 MHz, $CDCl_3$) (as mixture of E/Z amide bond isomers, signals of minor isomer are indicated with *): δ –112.2 (dd, *J* = 15.9, 5.0 Hz), –114.2 (dd, *J* = 16.1, 5.2 Hz)*;

HRMS: m/z (ESI) calculated for $(M + H)^+$: 294.1061, found: 294.1080;

R_f (*n*-hexane/EtOAc = 6/4): 0.26;

ATR-IR ν (cm^{-1}): 2913, 1705, 1645, 1490, 1428, 1231, 1091, 1012, 973, 828, 729;

$[\alpha]_D^{25}$: – 33.26 (c 1.0, $CHCl_3$);

Chiral SFC Separation: 30% MeOH, 2.5 mL/min, AD-H column, λ = 254 nm, t_R (min): minor = 7.65 (area: 2.17%), major = 9.41 (area: 97.83%), 95.7% enantiomeric excess.

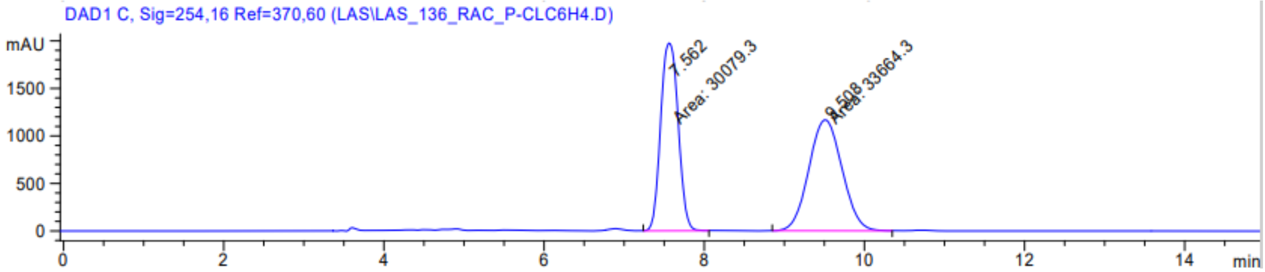

Signal 2: DAD1 C, Sig=254,16 Ref=370,60

Peak #	RetTime [min]	Type	Width [min]	Area [mAU*s]	Height [mAU]	Area %
1	7.562	MM	0.2539	3.00793e4	1974.43616	47.1880
2	9.508	MM	0.4801	3.36643e4	1168.63647	52.8120

Totals : 6.37436e4 3143.07263

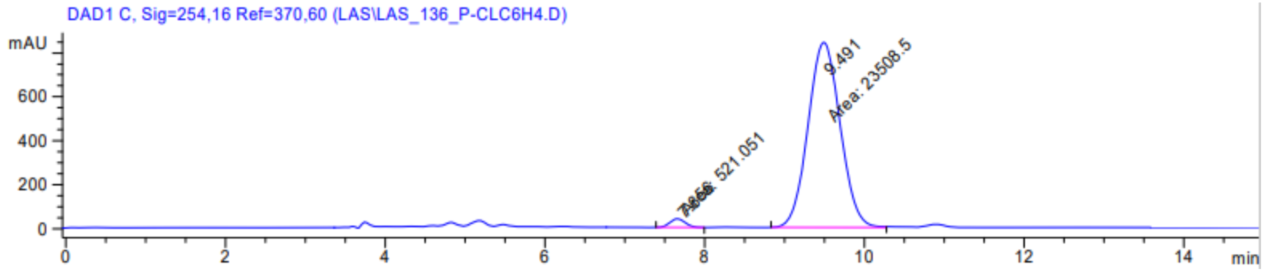

Signal 2: DAD1 C, Sig=254,16 Ref=370,60

Peak #	RetTime [min]	Type	Width [min]	Area [mAU*s]	Height [mAU]	Area %
1	7.656	MM	0.2214	521.05127	39.22103	2.1684
2	9.491	MM	0.4650	2.35085e4	842.62915	97.8316

Totals : 2.40295e4 881.85018

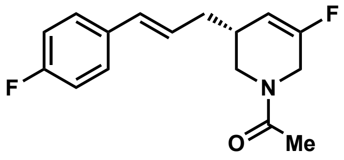

6o
Chemical Formula: $C_{16}H_{17}F_2NO$
Exact Mass: 277.1278
Molecular Weight: 277.3148

(*R*,*E*)-1-(5-fluoro-3-(3-(4-fluorophenyl)allyl)-3,6-dihydropyridin-1(2H)-yl)ethan-1-one 6o was synthesized following the general procedure B using 3-fluoropyridine (43 µL, 0.5 mmol, 1.0 equiv.) as substrate and (*E*)-3-(4-fluorophenyl)allyl methyl carbonate (158 mg, 0.75 mmol, 1.5 equiv.). The desired compound was obtained as yellowish oil (47 mg, 0.169 mmol, 34% yield over three steps).

^{1}H NMR (400 MHz, CDCl$_3$) (as mixture of E/Z amide bond isomers): δ 7.33–7.27 (m, 2H), 7.03–6.94 (m, 2H), 6.40 (dd, *J* = 15.8, 7.2 Hz, 1H), 6.12–6.00 (m, 1H), 5.46–5.30 (m, 1H), 4.18–4.05 (m, 1H), 4.03–3.91 (m, 1H), 3.86 (dd, *J* = 13.1, 4.7 Hz, 0.4H), 3.53 (dd, *J* = 13.6, 4.5 Hz, 0.6H), 3.33–3.22 (m, 1H), 2.50 (br s, 1H), 2.30–2.15 (m, 2H), 2.12 (s, 3H);

^{13}C NMR (101 MHz, $CDCl_3$) (as mixture of E/Z amide bond isomers, signals of minor isomer are indicated with *): δ 169.8, 169.7*, 162.3 (d, J = 248 Hz), 162.2 (d, J = 246 Hz)*, 156.4 (d, J = 257 Hz), 154.9 (d, J = 257 Hz)*, 133.52 (d, J = 3.3 Hz)*, 133.20 (d, J = 3.3 Hz), 131.8, 131.6*, 127,7 (d, J = 8.1 Hz)*, 127.7 (d, J = 8.1 Hz), 126.4 (d, J = 2.6 Hz)*, 126.4 (d, J = 2.2 Hz), 115.6 (d, J = 22.2 Hz), 115.5 (d, J = 21.2 Hz)*, 106.1 (d, J = 11 Hz)*, 104.4 (d, J = 12.5 Hz), 47.7, 44.8 (d, J = 39.2 Hz)*, 42.9*, 41.2 (d, J = 40 Hz), 36.8 (d, J = 2.2 Hz)*, 36.7 (d, J = 2.6 Hz), 34.3 (d, J = 6.2 Hz), 33.4 (d, J = 5.9 Hz)*, 22.0*, 21.5;

^{19}F NMR (282 MHz, $CDCl_3$) (as mixture of E/Z amide bond isomers, signals of minor isomer are indicated with *): δ –112.35 (dd, J = 16.3, 5.0 Hz), –114.30 (dd, J = 16.1, 5.7 Hz)*, –114.55 – (–114.70)(m), –115.05 – (–115.20)(m)*;

HRMS: m/z (ESI) calculated for $(M + H)^+$: 278.1356, found: 278.1364;

R_f (*n*-hexane/EtOAc = 6/4): 0.14;

ATR-IR ν (cm^{-1}): 2922, 1646, 1508, 1428, 1228, 837, 728;

$[\alpha]_D^{25}$: – 43.08 (c 1.0, $CHCl_3$);

Chiral SFC Separation: 10% MeOH, 2.5 mL/min, AD-H column, λ = 254 nm, t_R (min): minor = 15.73 (area: 3.25%), major = 18.08 (area: 96.75%), 93.5% enantiomeric excess;

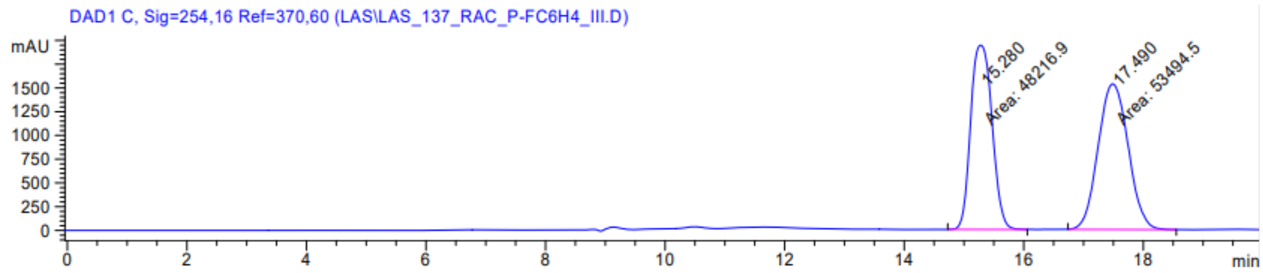

Signal 2: DAD1 C, Sig=254,16 Ref=370,60

Peak #	RetTime [min]	Type	Width [min]	Area [mAU*s]	Height [mAU]	Area %
1	15.280	MM	0.4145	4.82169e4	1938.92371	47.4056
2	17.490	MM	0.5819	5.34945e4	1532.15027	52.5944
Totals :				1.01711e5	3471.07397	

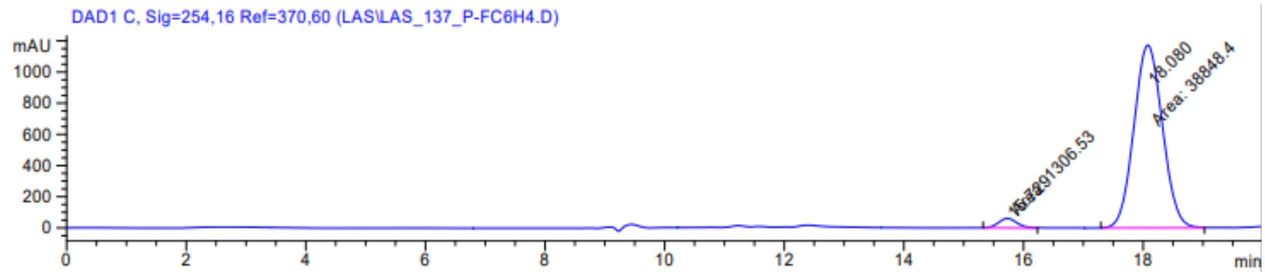

```
Signal 2: DAD1 C, Sig=254,16 Ref=370,60

Peak RetTime Type  Width     Area       Height      Area
  #   [min]        [min]   [mAU*s]      [mAU]        %
----|-------|----|-------|----------|----------|--------|
   1  15.729 MM    0.3570 1306.52649   60.98846   3.2537
   2  18.080 MM    0.5522 3.88484e4  1172.47803  96.7463

Totals :                  4.01549e4  1233.46649
```

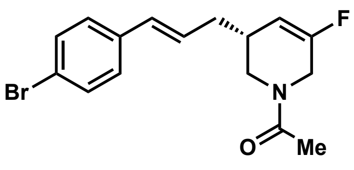

6p

Chemical Formula: $C_{16}H_{17}BrFNO$
Exact Mass: 337.0478
Molecular Weight: 338.2204

(*R*,*E*)-1-(3-(3-(4-bromophenyl)allyl)-5-fluoro-3,6-dihydropyridin-1(2H)-yl)ethan-1-one 6p was synthesized following the general procedure B using 3-fluoropyridine (43 µL, 0.5 mmol, 1.0 equiv.) as substrate and (*E*)-3-(4-bromophenyl)allyl methyl carbonate (203 mg, 0.75 mmol, 1.5 equiv.). The desired compound was obtained as yellowish oil (45 mg, 0.133 mmol, 27% yield over three steps).

^{1}H NMR (400 MHz, CDCl$_3$) (as mixture of E/Z amide bond isomers): δ 7.45–7.38 (m, 2H), 7.20 (d, *J* = 7.6 Hz, 2H), 6.37 (dd, *J* = 15.9, 6.7 Hz, 1H), 6.20–6.08 (m, 1H), 5.45–5.30 (m, 1H), 4.18–4.04 (m, 1H), 4.03–3.90 (m, 1H), 3.83 (dd, *J* = 13.1, 4.7 Hz, 0.4H), 3.53 (dd, *J* = 13.4, 4.5 Hz, 0.6H), 3.34–3.20 (m, 1H), 2.48 (br s, 1H), 2.32–2.16 (m, 2H), 2.11 (s, 3H);

^{13}C NMR (101 MHz, CDCl$_3$) (as mixture of E/Z amide bond isomers, signals of minor isomer are indicated with *): δ 169.8*, 169.7, 156.4 (d, *J* = 257 Hz), 154.9 (d, *J* = 257 Hz)*, 136.3*, 135.9, 131.9*, 131.8, 131.7, 131.6*, 127.8*, 127.7, 127.6*, 127.5, 121.3, 121.0*, 106.1 (d, *J* = 11.4 Hz)*, 104.3 (d, *J* = 12.5 Hz), 47.7, 44.8 (d, *J* = 39.2 Hz)*, 42.9*, 41.1 (d, *J* = 39.6 Hz), 36.8 (d, *J* = 2.2 Hz)*, 36.8 (d, *J* = 2.6 Hz), 34.2 (d, *J* = 6.6 Hz), 33.3 (d, *J* = 5.9 Hz)*, 22.0*, 21.5;

^{19}F NMR (282 MHz, CDCl$_3$) (as mixture of E/Z amide bond isomers, signals of minor isomer are indicated with *): δ –112.22 (dd, *J* = 16.1, 4.7 Hz), –114.16 (dd, *J* = 16.3, 5.4 Hz);

HRMS: m/z (ESI) calculated for $(M+H)^+$: 338.0556, found: 338.0554;

R$_f$ (*n*-hexane/EtOAc = 1/1): 0.23;

ATR-IR ν (cm^{-1}): 2917, 1706, 1648, 1486, 1426, 1379, 1229, 1164, 1071, 1007, 969, 838;

$[\alpha]_D^{25}$: – 36.48 (c 1.0, CHCl$_3$);

Chiral SFC Separation: 30% MeOH, 2.5 mL/min, AD-H column, λ = 254 nm, t_R (min): minor = 6.15 (area: 3.17%), major = 8.83 (area: 96.83%), 93.6% enantiomeric excess.

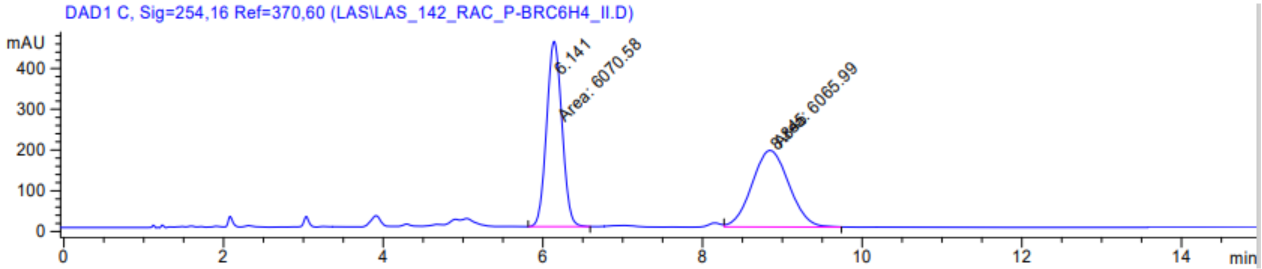

Signal 2: DAD1 C, Sig=254,16 Ref=370,60

Peak #	RetTime [min]	Type	Width [min]	Area [mAU*s]	Height [mAU]	Area %
1	6.141	MM	0.2225	6070.58252	454.82550	50.0189
2	8.845	MM	0.5376	6065.99072	188.07510	49.9811

Totals : 1.21366e4 642.90060

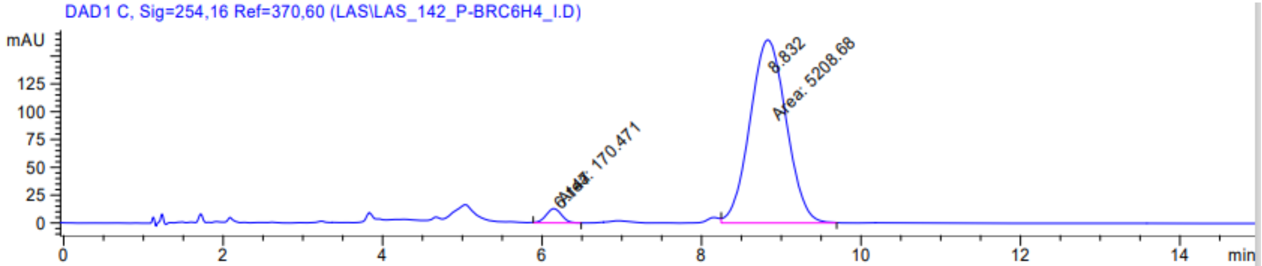

Signal 2: DAD1 C, Sig=254,16 Ref=370,60

Peak #	RetTime [min]	Type	Width [min]	Area [mAU*s]	Height [mAU]	Area %
1	6.147	MM	0.2215	170.47104	12.82446	3.1691
2	8.832	FM	0.5278	5208.68311	164.49005	96.8309

Totals : 5379.15414 177.31451

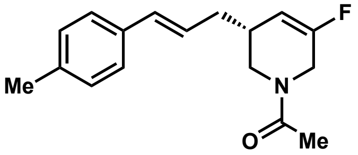

6q
Chemical Formula: $C_{17}H_{20}FNO$
Exact Mass: 273.1529
Molecular Weight: 273.3514

(*R*,*E*)-1-(5-fluoro-3-(3-(p-tolyl)allyl)-3,6-dihydropyridin-1(2H)-yl)ethan-1-one 6q was synthesized following the general procedure B using 3-fluoropyridine (43 µL, 0.5 mmol, 1.0 equiv.) as substrate and (*E*)-methyl (3-(*p*-tolyl)allyl) carbonate (174 mg, 0.75 mmol, 1.5 equiv.). The desired compound was obtained as colourless oil (61 mg, 0.223 mmol, 45% yield over three steps).

^{1}H NMR (400 MHz, CDCl$_3$) (as mixture of E/Z amide bond isomers): δ 7.27–7.21 (m, 2H), 7.16–7.08 (m, 2H), 6.40 (dd, *J* = 15.8, 7.6 Hz, 1H), 6.15–6.03 (m, 1H), 5.48–5.30 (m, 1H), 4.19–4.03 (m, 1H), 4.01–3.94 (m, 1H), 3.91 (dd, *J* = 13.1, 4.7 Hz, 0.4H), 3.52 (dd, *J* = 13.5, 4.5 Hz, 0.6H), 3.24 (td, *J* = 13.4, 6.5 Hz, 1H), 2.49 (br s, 1H), 2.35–2.30 (m, 3H), 2.30–2.16 (m, 2H), 2.11 (s, 3H);

^{13}C NMR (101 MHz, $CDCl_3$) (as mixture of E/Z amide bond isomers, signals of minor isomer are indicated with *): δ 169.8, 169.6*, 156.24 (d, *J* = 255 Hz), 154.7 (d, *J* = 256 Hz)*, 137.4, 137.1*, 134.5*, 134.2, 132.8, 132.5*, 129.4, 129.3*, 126.1*, 126.0, 125.6, 125.6*, 106.1 (d, *J* = 11.0 Hz)*, 104.5 (d, *J* = 12.1 Hz), 47.0, 44.8 (d, *J* = 39 Hz)*, 43.0*, 41.1 (d, *J* = 39 Hz), 36.8 (d, *J* = 2.2 Hz)*, 36.7 (d, *J* = 2.6 Hz), 34.3 (d, *J* = 6.2 Hz), 33.4 (d, *J* = 5.9 Hz)*, 22.0*, 21.5, 21.5*, 21.2;

^{19}F NMR (282 MHz, $CDCl_3$): δ–112.55 (dd, *J* = 15.9, 5.0 Hz), –114.44 (dd, *J* = 16.3, 5.4 Hz);

HRMS: m/z (ESI) calculated for $(M + H)^+$: 274.1607, found: 274.1610;

R_f (*n*-hexane/EtOAc = 6/4): 0.21;

ATR-IR ν (cm^{-1}): 3022, 2919, 1708, 1652, 1512, 1427, 1380, 1274, 1230, 1161, 1109, 1035, 970, 825, 788;

$[\alpha]_D^{25}$: – 41.25 (c 1.0, $CHCl_3$);

Chiral SFC Separation: 30% MeOH, 2.5 mL/min, AD-H column, λ = 254 nm, t_R (min): minor = 4.34 (area: 3.57%), major = 6.00 (area: 96.43%), 92.86% enantiomeric excess.

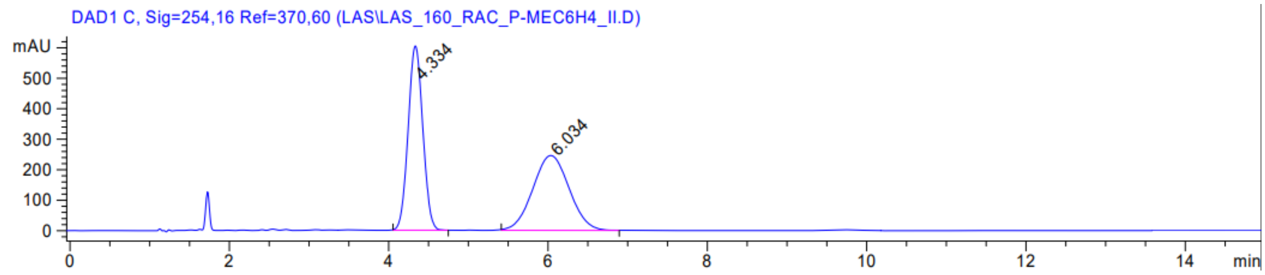

Signal 2: DAD1 C, Sig=254,16 Ref=370,60

Peak #	RetTime [min]	Type	Width [min]	Area [mAU*s]	Height [mAU]	Area %
1	4.334	BB	0.2012	7725.56982	604.15234	49.8360
2	6.034	BB	0.4972	7776.40967	245.71536	50.1640

Totals : 1.55020e4 849.86771

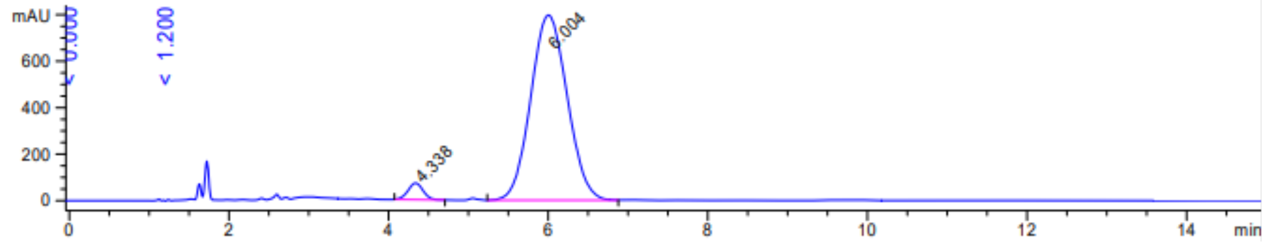

```
Signal 2: DAD1 C, Sig=254,16 Ref=370,60

Peak RetTime Type  Width     Area       Height      Area
  #   [min]        [min]   [mAU*s]      [mAU]        %
----|-------|----|-------|----------|----------|--------|
   1   4.338 BB    0.2011  939.37775   71.56826   3.5722
   2   6.004 VB    0.4994 2.53577e4   796.44708  96.4278

Totals :                  2.62971e4   868.01534
```

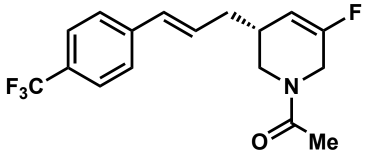

6r
Chemical Formula: $C_{17}H_{17}F_4NO$
Exact Mass: 327.1246
Molecular Weight: 327.3226

(*R*,*E*)-1-(5-fluoro-3-(3-(4-(trifluoromethyl)phenyl)allyl)-3,6-dihydropyridin-1(2H)-yl)ethan-1-one 6r was synthesized following the general procedure B using 3-fluoropyridine (43 µL, 0.5 mmol, 1.0 equiv.) as substrate and (*E*)-methyl (3-(4-(trifluoromethyl)phenyl)allyl) carbonate (195 mg, 0.75 mmol, 1.5 equiv.). The desired compound was obtained as colourless oil (50 mg, 0.153 mmol, 31% yield over three steps).

^{1}H NMR (400 MHz, CDCl$_3$) (as mixture of E/Z amide bond isomers): δ 7.59–7.51 (m, 2H), 7.47–7.40 (m, 2H), 6.56–6.43 (m, 1H), 6.33–6.20 (m, 1H), 5.48–5.30 (m, 1H), 4.19–4.06 (m, 1H), 4.04–3.93 (m, 1H), 3.85–3.78 (m, 0.4H), 3.59–3.50 (m, 0.6H), 3.40–3.22 (m, 1H), 2.60–2.45 (br s, 1H), 2.38–2.19 (m, 2H), 2.12 (s, 3H);

^{13}C NMR (101 MHz, CDCl$_3$) (as mixture of E/Z amide bond isomers, signals of minor isomer are indicated with *): δ 169.8, 169.7*, 156.5 (d, *J* = 256 Hz), 155.0 (d, *J* = 256 Hz)*, 140.8*, 140.5, 131.8, 131.6*, 129.6*, 129.5, 129.3, 129.0*, 126.4*, 126.4, 125.8 (q, *J* = 3.7 Hz), 125.6 (q, *J* = 4.0 Hz)*, 106.0 (d, *J* = 11.4 Hz)*, 104.3 (d, *J* = 12.5 Hz), 47.8, 44.9 (d, *J* = 39 Hz), 42.8*, 41.2 (d, *J* = 40 Hz), 36.9, 36.8*, 34.2 (d, *J* = 6.2 Hz), 33.3 (d, *J* = 6.2 Hz)*, 22.0*, 21.6; The carbon atom for the CF_3-group was not detected.

^{19}F NMR (282 MHz, CDCl$_3$) (as mixture of E/Z amide bond isomers, signals of minor isomer are indicated with *): δ –62.44* (s), –62.50 (s), –112.06 (dd, *J* = 15.9, 5.0 Hz), –114.02 (dd, *J* = 16.3, 5.0 Hz)*;

HRMS: m/z (ESI) calculated for $(M + H)^+$: 328.1325, found: 328.1338;

R_f (*n*-hexane/EtOAc = 6/4): 0.18;

ATR-IR ν (cm^{-1}): 2921, 1651, 1434, 1326, 1233, 1163, 1121, 1067, 1016, 839;

$[\alpha]_D^{25}$: – 26.31 (c 1.0, CHCl$_3$);

Chiral SFC Separation: 7% *i*PrOH, 2.5 mL/min, AD-H column, λ = 254 nm, t_R (min): major = 11.26 (area: 97.72%), minor = 12.22 (area: 2.28%), 95.4% enantiomeric excess.

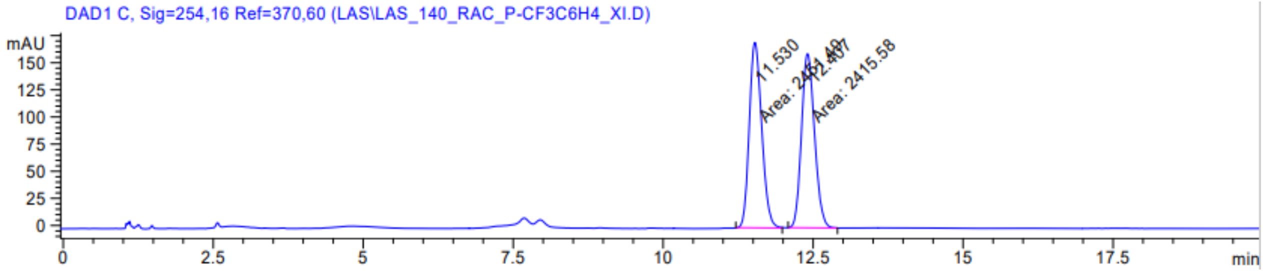

Signal 2: DAD1 C, Sig=254,16 Ref=370,60

Peak #	RetTime [min]	Type	Width [min]	Area [mAU*s]	Height [mAU]	Area %
1	11.530	MM	0.2391	2451.48804	170.85316	50.3689
2	12.407	MM	0.2509	2415.58301	160.48860	49.6311
Totals :				4867.07104	331.34177	

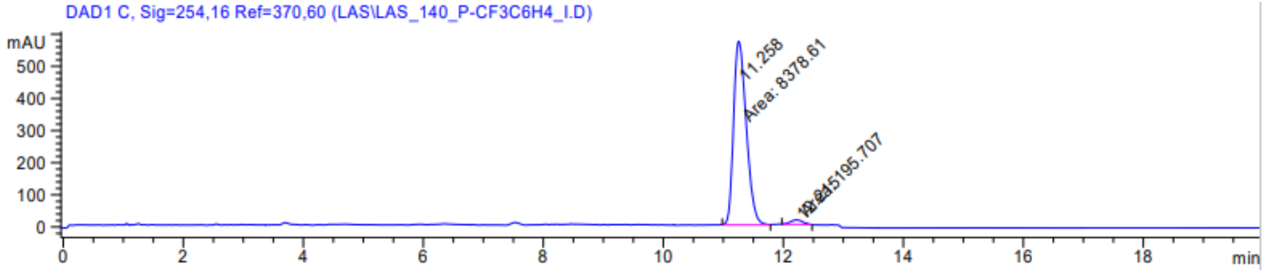

Signal 2: DAD1 C, Sig=254,16 Ref=370,60

Peak #	RetTime [min]	Type	Width [min]	Area [mAU*s]	Height [mAU]	Area %
1	11.258	MM	0.2440	8378.60938	572.39178	97.7175
2	12.215	MM	0.2372	195.70685	13.75326	2.2825
Totals :				8574.31622	586.14504	

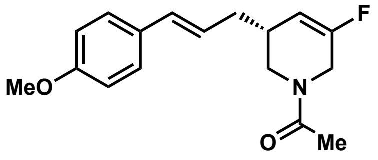

6s
Chemical Formula: $C_{17}H_{20}FNO_2$
Exact Mass: 289.1478
Molecular Weight: 289.3504

(*R*,*E*)-1-(5-fluoro-3-(3-(4-methoxyphenyl)allyl)-3,6-dihydropyridin-1(2H)-yl)ethan-1-one 6s was synthesized following the general procedure B using 3-fluoropyridine (43 µL, 0.5 mmol, 1.0 equiv.) as substrate and (*E*)-3-(4-methoxyphenyl)allyl methyl carbonate (168 mg, 0.75 mmol, 1.5 equiv.). The desired compound was obtained as colourless oil (49 mg, 0.169 mmol, 34% yield over three steps).

^{1}H NMR (400 MHz, CDCl$_3$) (as mixture of E/Z amide bond isomers): δ 7.31–7.23 (m, 2H), 6.88–6.80 (m, 2H), 6.44–6.32 (m, 1H), 6.07–5.93 (m, 1H), 5.48–5.30 (m, 1H), 4.18–4.05 (m, 1H), 4.02–3.93 (s, 1H), 3.93–3.87 (m, 0.4H), 3.81–3.79 (m, 3H), 3.52 (dd, *J* = 13.6, 4.6 Hz, 0.6H), 3.33–3.17 (m, 1H), 2.49 (br s, 1H), 2.39–2.13 (m, 2H), 2.11 (s, 3H);

^{13}C NMR (101 MHz, CDCl$_3$) (as mixture of E/Z amide bond isomers, signals of minor isomer are indicated with *): δ 169.8, 169.6*, 159.2, 159.0*, 156.3 (d, *J* = 256 Hz), 154.8 (d, *J* = 256

Hz)*, 132.4, 132.1*, 130.2*, 129.9, 127.4*, 127.3, 124.4*, 124.4, 114.1, 114.0*, 106.2 (d, J = 11.0 Hz)*, 104.6 (d, J = 12.5 Hz), 55.4, 55.4*, 47.6, 44.8 (d, J = 39 Hz)*, 43.0*, 41.2 (d, J = 40 Hz), 36.8 (d, J = 2.2 Hz)*, 36.8 (d, J = 2.6 Hz), 34.4 (d, J = 6.2 Hz), 33.5 (d, J = 5.9 Hz)*, 22.0*, 21.5;

^{19}F NMR (282 MHz, $CDCl_3$) (as mixture of E/Z amide bond isomers, signals of minor isomer are indicated with *): δ–112.61 (dd, J = 15.9, 5.0 Hz), –114.52 (dd, J = 16.8, 5.4 Hz)*;

HRMS: m/z (ESI) calculated for $(M + H)^+$: 290.1556, found: 290.1563;

R_f (n-hexane/EtOAc = 6/4): 0.24;

ATR-IR ν (cm^{-1}): 2915, 1647, 1511, 1456, 1248, 1173, 1159, 1032;

$[\alpha]_D^{25}$: – 35.38 (c 1.0, $CHCl_3$);

Chiral SFC Separation: 35% MeOH, 2.5 mL/min, AD-H column, λ = 254 nm, t_R (min): minor = 4.84 (area: 3.52%), major = 7.46 (area: 96.48%), 93.0% enantiomeric excess.

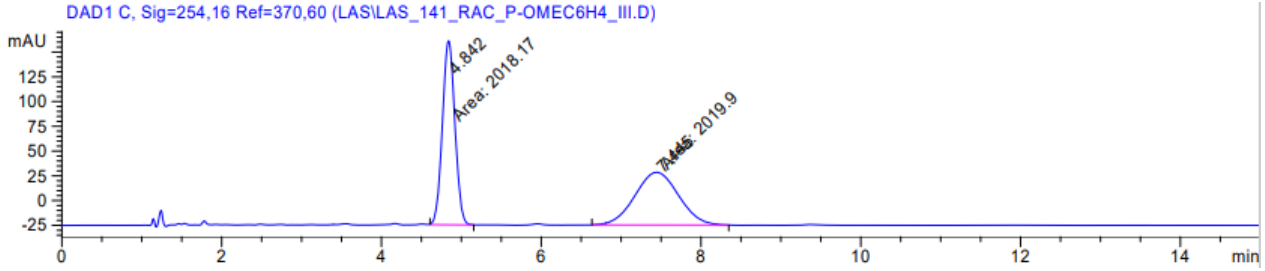

Signal 2: DAD1 C, Sig=254,16 Ref=370,60

Peak #	RetTime [min]	Type	Width [min]	Area [mAU*s]	Height [mAU]	Area %
1	4.842	MM	0.1808	2018.17383	186.01950	49.9786
2	7.445	MM	0.6333	2019.90381	53.15785	50.0214

Totals : 4038.07764 239.17735

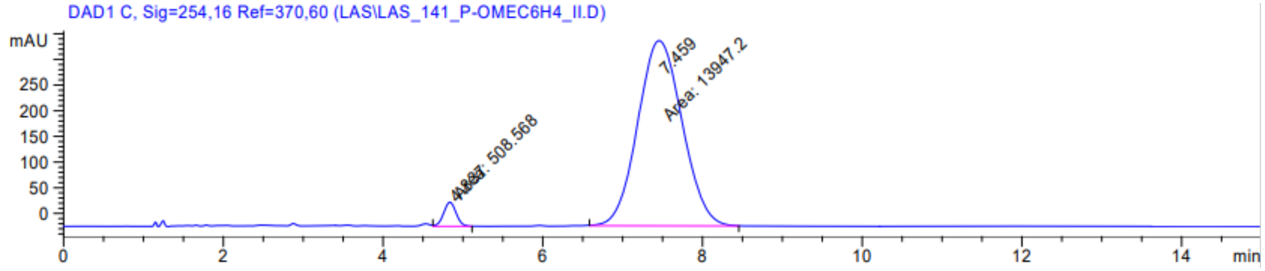

```
Signal 2: DAD1 C, Sig=254,16 Ref=370,60

Peak RetTime Type  Width     Area      Height     Area
  #   [min]        [min]   [mAU*s]     [mAU]        %
----|-------|----|-------|----------|----------|--------|
   1   4.837 MM    0.1804  508.56772   46.98798   3.5181
   2   7.459 MM    0.6439 1.39472e4   360.99673  96.4819

Totals :                  1.44558e4   407.98472
```

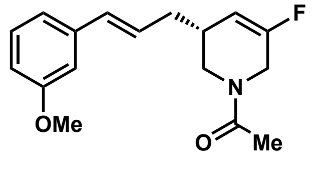

6t
Chemical Formula: $C_{17}H_{20}FNO_2$
Exact Mass: 289.1478
Molecular Weight: 289.3504

(*R*,*E*)-1-(5-fluoro-3-(3-(3-methoxyphenyl)allyl)-3,6-dihydropyridin-1(2H)-yl)ethan-1-one 6t was synthesized following the general procedure B using 3-fluoropyridine (43 µL, 0.5 mmol, 1.0 equiv.) as substrate and (*E*)-3-(3-methoxyphenyl)allyl methyl carbonate (167 mg, 0.75 mmol, 1.5 equiv.). The desired compound was obtained as colourless oil (52 mg, 0.180 mmol, 36% yield over three steps).

^{1}H NMR (400 MHz, CD_2Cl_2) (as mixture of E/Z amide bond isomers): δ 7.26–7.18 (m, 1H), 6.98–6.93 (m, 1H), 6.91–6.87 (m, 1H), 6.81–6.74 (m, 1H), 6.42 (dd, *J* = 15.8, 9.7 Hz, 1H), 6.25–6.14 (m, 1H), 5.48–5.34 (m, 1H), 4.15–3.92 (m, 2H), 3.86–3.77 (m, 3.4H), 3.54 (dd, *J* = 13.2, 4.9 Hz, 0.6H), 3.30–3.20 (m, 1H), 2.58–2.42 (m, 1H), 2.32–2.15 (m, 2H), 2.10–2.06 (m, 3H);

^{13}C NMR (101 MHz, CD_2Cl_2) (as mixture of E/Z amide bond isomers, signals of minor isomer are indicated with *): δ 169.7, 169.6*, 160.3, 160.3*, 156.8 (d, J = 256 Hz), 155.5 (d, *J* = 256 Hz)*, 139.2*, 139.0, 132.8, 132.6*, 129.9, 129.8*, 127.7*, 127.7, 119.0, 119.0*, 113.2, 113.1*, 111.8, 111.7*, 106.2 (d, *J* = 11.0 Hz)*, 104.9 (d, *J* = 12.1 Hz), 55.5, 55.5*, 47.9, 45.0 (d, *J* = 39 Hz)*, 43.0*, 41.2 (d, *J* = 40 Hz), 37.1*, 36.9, 34.6 (d, *J* = 6.2 Hz), 33.7 (d, *J* = 5.9 Hz)*, 22.1*, 21.6;

^{19}F NMR (282 MHz, CD_2Cl_2) (as mixture of E/Z amide bond isomers, signals of minor isomer are indicated with *): δ–113.52 (dd, *J* = 16.6, 4.7 Hz), –115.27 (dd, *J* = 16.8, 4.5 Hz);

HRMS: m/z (ESI) calculated for $(M + H)^+$: 290.1556, found: 290.1560;

R_f (*n*-hexane/EtOAc = 6/4): 0.17;

ATR-IR ν (cm^{-1}): 2922, 1706, 1651, 1598, 1578, 1431, 1380, 1264, 1233, 1158, 1041, 972, 834, 776, 689;

$[\alpha]_D^{25}$: – 40.94 (c 1.0, $CHCl_3$);

Chiral SFC Separation: 30% MeOH, 2.5 mL/min, AD-H column, λ = 254 nm, t_R (min): minor = 5.22 (area: 2.90%), major = 12.67 (area: 97.10%), 94.2% enantiomeric excess.

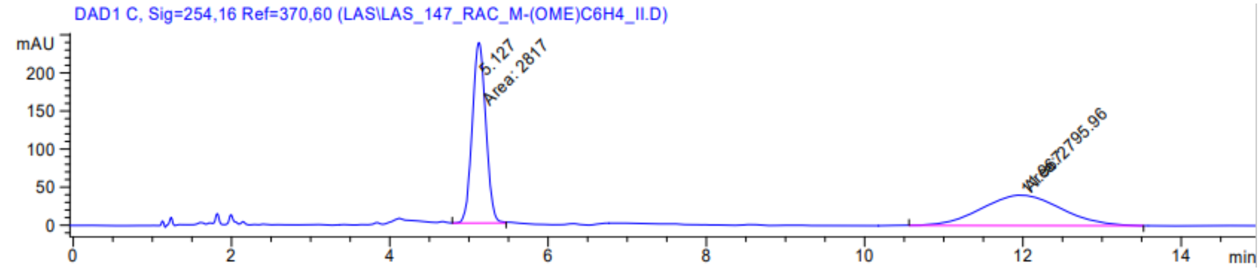

Signal 2: DAD1 C, Sig=254,16 Ref=370,60

Peak #	RetTime [min]	Type	Width [min]	Area [mAU*s]	Height [mAU]	Area %
1	5.127	MM	0.1978	2816.99536	237.34180	50.1874
2	11.967	MM	1.1664	2795.95776	39.95090	49.8126
Totals :				5612.95313	277.29269	

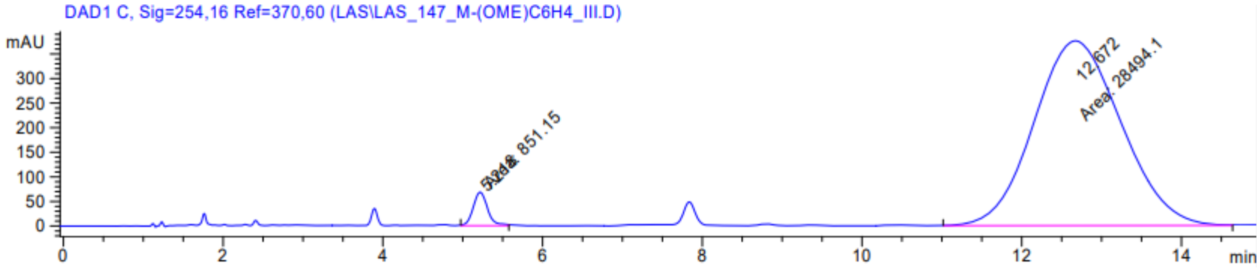

Signal 2: DAD1 C, Sig=254,16 Ref=370,60

Peak #	RetTime [min]	Type	Width [min]	Area [mAU*s]	Height [mAU]	Area %
1	5.218	MM	0.2081	851.15015	68.15288	2.9005
2	12.672	MM	1.2638	2.84941e4	375.75864	97.0995
Totals :				2.93453e4	443.91151	

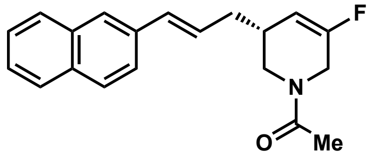

6u
Chemical Formula: $C_{20}H_{20}FNO$
Exact Mass: 309.1529
Molecular Weight: 309.3844

(*R*,*E*)-1-(5-fluoro-3-(3-(naphthalen-2-yl)allyl)-3,6-dihydropyridin-1(2H)-yl)ethan-1-one 6u was synthesized following the general procedure B using 3-fluoropyridine (43 µL, 0.5 mmol, 1.0 equiv.) as substrate and (*E*)-methyl (3-(naphthalen-2-yl)allyl) carbonate (181 mg, 0.75 mmol, 1.5 equiv.). The desired compound was obtained as colourless oil (39 mg, 0.126 mmol, 25% yield over three steps).

^{1}H NMR (400 MHz, CD_2Cl_2) (as mixture of E/Z amide bond isomers): δ 7.85–7.77 (m, 3H), 7.72 (s, 1H), 7.64–7.58 (m, 1H), 7.52–7.40 (m, 2H), 6.67–6.58 (m, 1H), 6.39–6.28 (m, 1H), 5.53–5.37 (m, 1H), 4.18–3.95 (m, 2H), 3.84 (dd, *J* = 13.0, 4.7 Hz, 0.4H), 3.56 (dd, *J* = 13.6, 4.5 Hz, 0.6H), 3.37–3.23 (m, 1H), 2.60–2.46 (m, 1H), 2.40–2.22 (m, 2H), 2.11–2.07 (m, 3H);

^{13}C NMR (101 MHz, CD_2Cl_2) (as mixture of E/Z amide bond isomers, signals of minor isomer are indicated with *, one signal is overlapping): δ169.7, 169.6*, 156.8 (d, *J* = 256 Hz), 155.5 (d,

J = 256 Hz)*, 135.3*, 135.0, 134.1*, 134.0, 133.3, 133.2*, 133.0, 132.8*, 128.5, 128.4*, 128.2, 128.0, 128.0*, 127.9*, 126.7, 126.6*, 126.2, 126.1, 126.0*, 126.0*, 123.9*, 123.8, 106.3 (d, *J* = 11.0 Hz)*, 104.9 (d, *J* = 12.1 Hz), 47.9, 45.0 (d, *J* = 39 Hz)*, 43.6*, 41.2 (d, *J* = 39 Hz), 37.2 (d, *J* = 2.2 Hz)*, 37.1 (d, *J* = 2.6 Hz), 34.6 (d, *J* = 6.2 Hz), 33.8 (d, *J* = 5.9 Hz)*, 22.1*, 21.6;

^{19}F NMR (282 MHz, CD_2Cl_2) (as mixture of E/Z amide bond isomers, signals of minor isomer are indicated with *): δ–113.47 (dd, *J* = 16.8, 5.0 Hz), –115.23 (dd, *J* = 16.6, 5.2 Hz);

HRMS: m/z (ESI) calculated for $(M + H)^+$: 310.1602, found: 310.1289;

R_f (*n*-hexane/EtOAc = 6/4): 0.21;

ATR-IR ν (cm^{-1}): 2918, 1737, 1712, 1651, 1506, 1428, 1383, 1271, 1229, 1165, 967, 897, 860, 825, 748, 617;

$[\alpha]_D^{25}$: – 47.73 (c 1.0, $CHCl_3$);

Chiral SFC Separation: 45% MeOH, 2.5 mL/min, AD-H column, λ = 254 nm, t_R (min): minor = 6.13 (area: 2.20%), major = 12.21 (area: 97.80%), 95.6% enantiomeric excess.

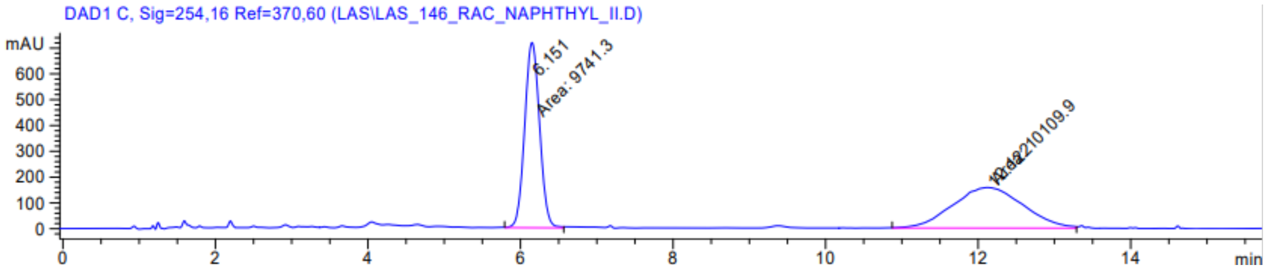

Signal 2: DAD1 C, Sig=254,16 Ref=370,60

Peak #	RetTime [min]	Type	Width [min]	Area [mAU*s]	Height [mAU]	Area %
1	6.151	MM	0.2260	9741.30273	718.51941	49.0717
2	12.122	MM	1.0702	1.01099e4	157.44484	50.9283
Totals :				1.98512e4	875.96425	

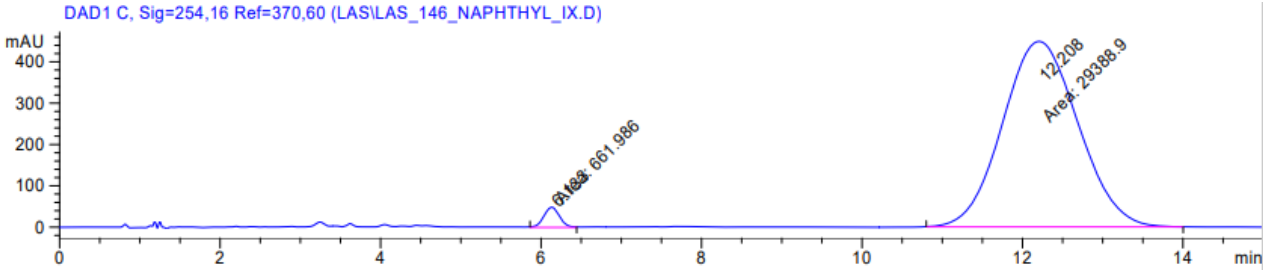

```
Signal 2: DAD1 C, Sig=254,16 Ref=370,60

Peak RetTime Type  Width     Area       Height      Area
  #   [min]        [min]    [mAU*s]     [mAU]        %
----|-------|----|-------|----------|----------|--------|
   1   6.133 MM    0.2259  661.98602   48.84750   2.2029
   2  12.208 MM    1.0918 2.93889e4   448.63095  97.7971

Totals :                  3.00509e4   497.47845
```

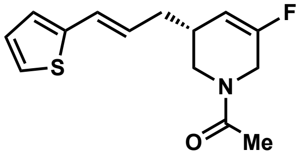

6v
Chemical Formula: $C_{14}H_{16}FNOS$
Exact Mass: 265.0937
Molecular Weight: 265.3464

(*R*,*E*)-1-(5-fluoro-3-(3-(thiophen-2-yl)allyl)-3,6-dihydropyridin-1(2H)-yl)ethan-1-one 6v was synthesized following the general procedure B using 3-fluoropyridine (43 µL, 0.5 mmol, 1.0 equiv.) as substrate and (*E*)-methyl (3-(thiophen-2-yl)allyl) carbonate (149 mg, 0.75 mmol, 1.5 equiv.). The desired compound was obtained as colorless oil (34 mg, 0.128 mmol, 26% yield over three steps).

^{1}H NMR (400 MHz, CDCl$_3$) (as mixture of E/Z amide bond isomers): δ 7.12 (dd, *J* = 11.0, 5.1 Hz, 1H), 6.98–6.92 (m, 1H), 6.92–6.88 (m, 1H), 6.61–6.52 (m, 1H), 6.04–5.92 (m, 1H), 5.47–5.30 (m, 1H), 4.18–3.92 (m, 2H), 3.84 (dd, *J* = 13.1, 4.6 Hz, 0.4H), 3.53 (dd, *J* = 13.4, 4.5 Hz, 0.6H), 3.33–3.22 (m, 1H), 2.55–2.40 (s, 1H), 2.30–2.14 (m, 2H), 2.12 (s, 3H);

^{13}C NMR (101 MHz, CDCl$_3$) (as mixture of E/Z amide bond isomers, signals of minor isomer are indicated with *): δ 169.8, 169.7*, 156.4 (d, *J* = 256 Hz), 154.9 (d, *J* = 256 Hz)*, 142.5*, 142.1, 127.5, 127.4*, 126.5*, 126.4, 126.2, 125.9*, 125.3, 125.1*, 124.0, 123.7*, 106.0 (d, *J* = 11.0 Hz)*, 104.4 (d, *J* = 12.5 Hz), 47.7, 44.9 (d, *J* = 39 Hz)*, 42.9*, 41.2 (d, *J* = 40 Hz), 36.7 (d, *J* = 2.2 Hz)*, 36.63 (d, *J* = 2.6 Hz), 34.3 (d, *J* = 6.2 Hz), 33.3 (d, *J* = 5.9 Hz)*, 22.0*, 21.6;

^{19}F NMR (282 MHz, CDCl$_3$) (as mixture of E/Z amide bond isomers, signals of minor isomer are indicated with *): δ –112.35 (dd, *J* = 16.3, 5.0 Hz), –114.23 (dd, *J* = 16.3, 5.0 Hz)*;

HRMS: m/z (ESI) calculated for $(M + H)^+$: 266.1009, found: 266.1006;

R$_f$ (*n*-hexane/EtOAc = 6/4): 0.18;

ATR-IR ν (cm^{-1}): 2921, 1732, 1651, 1427, 1378, 1236, 1159, 1039, 697;

$[\alpha]_D^{25}$: – 35.56 (c 1.0, CHCl$_3$);

Chiral SFC Separation: 30% MeOH, 2.5 mL/min, AD-H column, λ = 254 nm, t_R (min): minor = 4.22 (area: 2.98%), major = 5.28 (area: 97.02%), 94.0% enantiomeric excess.

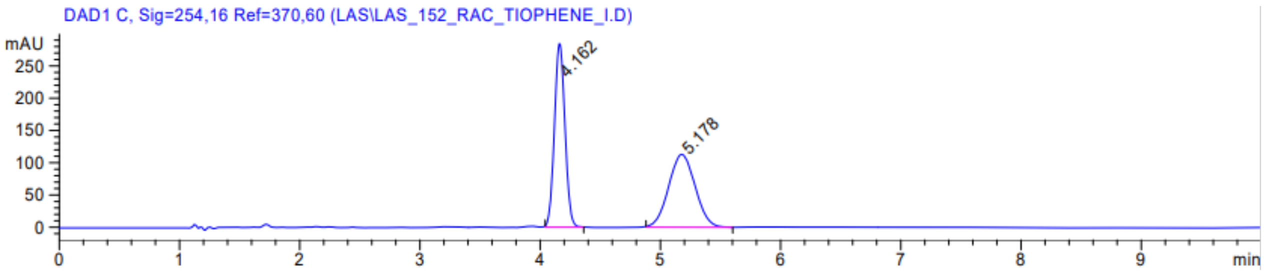

Signal 2: DAD1 C, Sig=254,16 Ref=370,60

Peak #	RetTime [min]	Type	Width [min]	Area [mAU*s]	Height [mAU]	Area %
1	4.162	BB	0.0991	1731.62109	283.99393	50.0738
2	5.178	BB	0.2385	1726.51636	112.82227	49.9262
Totals :				3458.13745	396.81620	

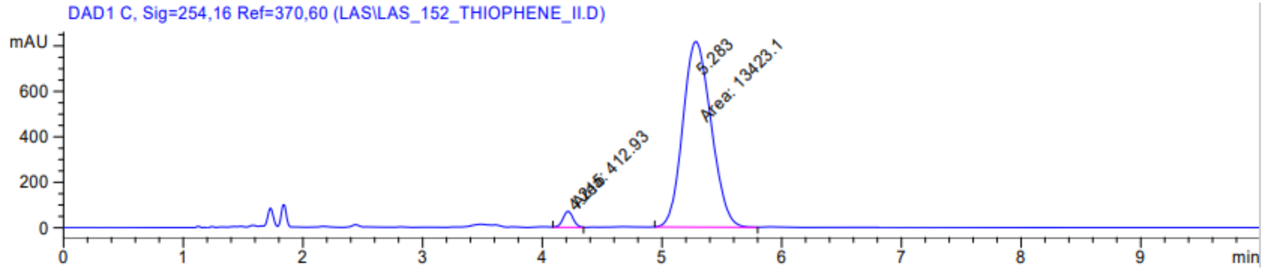

Signal 2: DAD1 C, Sig=254,16 Ref=370,60

Peak #	RetTime [min]	Type	Width [min]	Area [mAU*s]	Height [mAU]	Area %
1	4.215	MM	0.0971	412.92996	70.84860	2.9845
2	5.283	MM	0.2736	1.34231e4	817.65662	97.0155
Totals :				1.38360e4	888.50522	

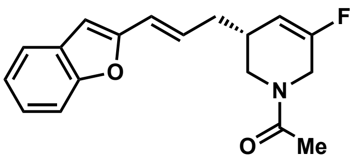

6w

Chemical Formula: $C_{18}H_{18}FNO_2$
Exact Mass: 299.1322
Molecular Weight: 299.3454

(*R*,*E*)-1-(3-(3-(benzofuran-2-yl)allyl)-5-fluoro-3,6-dihydropyridin-1(2H)-yl)ethan-1-one 6w was synthesized following the general procedure B using 3-fluoropyridine (43 µL, 0.5 mmol, 1.0 equiv.) as substrate and (*E*)-3-(benzofuran-2-yl)allyl methyl carbonate (174 mg, 0.75 mmol, 1.5 equiv.). The desired compound was obtained as colourless oil (58 mg, 0.194 mmol, 39% yield over three steps).

^{1}H NMR (400 MHz, $CDCl_3$) (as mixture of E/Z amide bond isomers): δ 7.47–7.38 (m, 1H), 7.38–7.32 (m, 1H), 7.22–7.06 (m, 2H), 6.46–6.40 (m, 1H), 6.35–6.27 (m, 2H), 5.42–5.24 (m, 1H), 4.12–3.84 (m, 2H), 3.75 (dd, *J* = 12.7, 4.6 Hz, 0.4H), 3.47 (dd, *J* = 12.5, 4.5 Hz, 0.6H), 3.28 (dd, *J* = 13.1, 6.5 Hz, 0.4H), 3.19 (dd, *J* = 13.6, 6.1 Hz, 0.6H), 2.52–2.37 (s, 1H), 2.30–2.11 (m, 2H), 2.07–2.00 (m, 3H);

^{13}C NMR (101 MHz, CDCl$_3$) (as mixture of E/Z amide bond isomers, signals of minor isomer are indicated with *): δ 169.8, 169.7*, 156.4 (d, J = 257 Hz), 155.0 (d, J = 257 Hz)*, 154.8, 154.7*, 154.5*, 154.2, 129.2, 129.1*, 129.0, 128.9*, 124.6, 124.4*, 123.0, 122.8*, 121.5, 121.3*, 121.0, 120.9*, 110.9, 110.9*, 105.9 (d, J = 11.0 Hz)*, 104.4 (d, J = 12.5 Hz), 104.1, 103.8*, 47.6, 44.8 (d, J = 39 Hz)*, 42.8*, 41.1 (d, J = 40 Hz), 36.8 (d, J = 2.6 Hz)*, 36.7 (d, J = 2.6 Hz), 34.2 (d, J = 6.2 Hz), 33.2 (d, J = 5.9 Hz)*, 22.0*, 21.5;

^{19}F NMR (282 MHz, CDCl$_3$) (as mixture of E/Z amide bond isomers, signals of minor isomer are indicated with *): δ–112.16 (dd, J = 16.1, 4.7 Hz), –114.03 (dd, J = 16.1, 5.2 Hz)*;

HRMS: m/z (ESI) calculated for $(M + H)^+$: 300.1400, found: 300.1385;

R$_f$ (*n*-hexane/EtOAc = 6/4): 0.14;

ATR-IR ν (cm^{-1}): 2917, 1711, 1650, 1452, 1380, 1254, 1230, 1163, 1105, 965, 882, 826, 751;

$[\alpha]_D^{25}$: – 34.73 (c 1.0, $CHCl_3$);

Chiral SFC Separation: 30% MeOH, 2.5 mL/min, AD-H column, λ = 254 nm, t_R (min): minor = 6.83 (area: 2.62%), major = 10.22 (area: 97.38%), 94.8% enantiomeric excess.

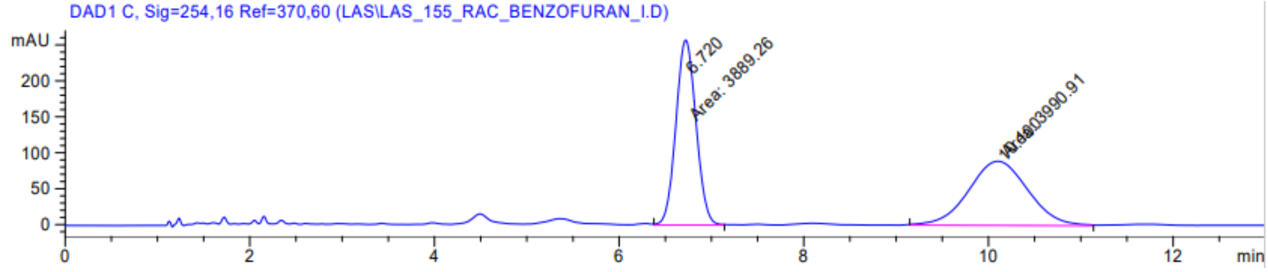

Signal 2: DAD1 C, Sig=254,16 Ref=370,60

Peak #	RetTime [min]	Type	Width [min]	Area [mAU*s]	Height [mAU]	Area %
1	6.720	MM	0.2516	3889.25513	257.60300	49.3550
2	10.100	MM	0.7447	3990.90601	89.32148	50.6450
Totals :				7880.16113	346.92448	

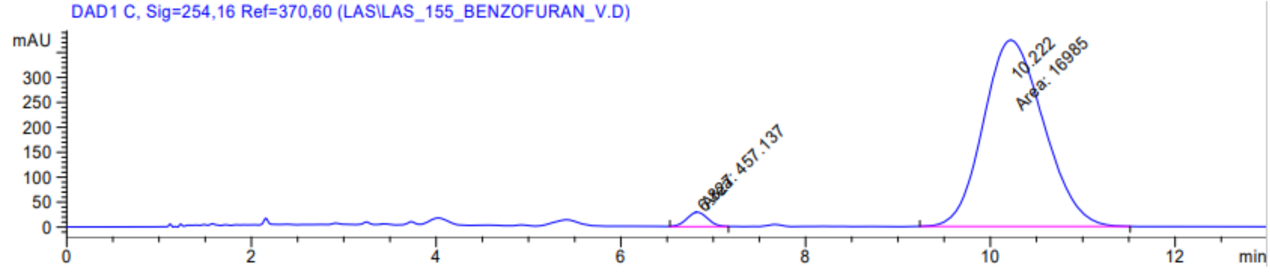

Signal 2: DAD1 C, Sig=254,16 Ref=370,60

Peak #	RetTime [min]	Type	Width [min]	Area [mAU*s]	Height [mAU]	Area %
1	6.827	MM	0.2607	457.13745	29.22491	2.6209
2	10.222	MM	0.7577	1.69850e4	373.63022	97.3791

Totals : 1.74421e4 402.85512

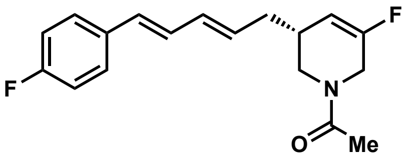

6x

Chemical Formula: $C_{18}H_{19}F_2NO$
Exact Mass: 303.1435
Molecular Weight: 303.3528

1-((*R*)-5-fluoro-3-((2*E*,4*E*)-5-(4-fluorophenyl)penta-2,4-dien-1-yl)-3,6-dihydropyridin-1(2H)-yl)ethan-1-one 6x was synthesized following the general procedure B using 3-fluoropyridine (43 µL, 0.5 mmol, 1.0 equiv.) as substrate and (2*E*,4*E*)-5-(4-fluorophenyl)penta-2,4-dien-1-yl methyl carbonate (236 mg, 0.75 mmol, 1.5 equiv.). The desired compound was obtained as colourless oil (31 mg, 0.102 mmol, 20% yield over three steps).

^{1}H NMR (400 MHz, CD_2Cl_2) (as mixture of E/Z amide bond isomers): δ 7.34–7.21 (m, 2H), 6.99–6.85 (m, 2H), 6.69–6.56 (m, 1H), 6.44–6.30 (m, 1H), 6.24–6.09 (m, 1H), 5.71 (ddd, *J* = 14.9, 7.5, 7.5 Hz, 1H), 5.37–5.24 (m, 1H), 4.08–3.83 (m, 2H), 3.72 (dd, *J* = 13.1, 4.7 Hz, 0.4H), 3.44 (dd, *J* = 13.5, 4.6 Hz, 0.6H), 3.20–3.07 (m, 1H), 2.47–2.29 (m, 1H), 2.19–2.05 (m, 2H), 2.04–1.96 (s, 3H);

^{13}C NMR (101 MHz, CD_2Cl_2) (as mixture of E/Z amide bond isomers, signals of minor isomer are indicated with *): δ 169.8, 169.7*, 162.6 (d, *J* = 247 Hz), 162.5 (d, *J* = 246 Hz)*, 156.8 (d, *J* = 255 Hz), 155 (d, *J* = 256 Hz)*, 134.2 (d, *J* = 2.9 Hz)*, 134.0 (d, *J* = 3.3 Hz), 133.4, 133.2*, 132.1*, 131.8, 130.3, 130.0*, 129.1 (d, *J* = 2.6 Hz)*, 128.9 (d, *J* = 2.2 Hz), 128.1 (d, *J* = 7.7 Hz), 128.1 (d, *J* = 8.1 Hz)*, 115.9 (d, *J* = 21.6 Hz), 115.8 (d, *J* = 22.0 Hz)*, 106.3 (d, *J* = 11 Hz)*, 104.9 (d, *J* = 12.1 Hz), 47.9, 45.0 (d, *J* = 39 Hz)*, 42.9*, 41.2 (d, *J* = 40 Hz), 36.9 (d, *J* = 2.2 Hz)*, 36.8 (d, *J* = 2.2 Hz), 34.6 (d, *J* = 6.2 Hz), 33.7 (d, *J* = 5.9 Hz)*, 22.1*, 21.7;

^{19}F NMR (282 MHz, CD_2Cl_2) (as mixture of E/Z amide bond isomers, signals of minor isomer are indicated with *): δ –113.6 (dd, *J* = 16.1, 4.7 Hz, C_{Alkyl}F), –115.24 – (–115.43 (m, C_{Alkyl}F*, C_{Aryl}F), –115.48 – (–115.60 (m, C_{Aryl}F*));

HRMS: m/z (ESI) calculated for $(M + H)^+$: 304.1513, found: 304.1500;

R_f (*n*-hexane/EtOAc = 6/4): 0.21;

ATR-IR ν (cm^{-1}): 2916, 1707, 1650, 1599, 1507, 1428, 1379, 1275, 1228, 1159, 1095, 1060, 1034, 989, 833;

$[\alpha]_D^{25}$: – 33.48 (c 1.0, $CHCl_3$);

Chiral SFC Separation: 40% MeOH, 2.5 mL/min, AD-H column, λ = 280 nm, t_R (min): minor = 6.35 (area: 3.43%), major = 10.95 (area: 96.57%), 93.1% enantiomeric excess.

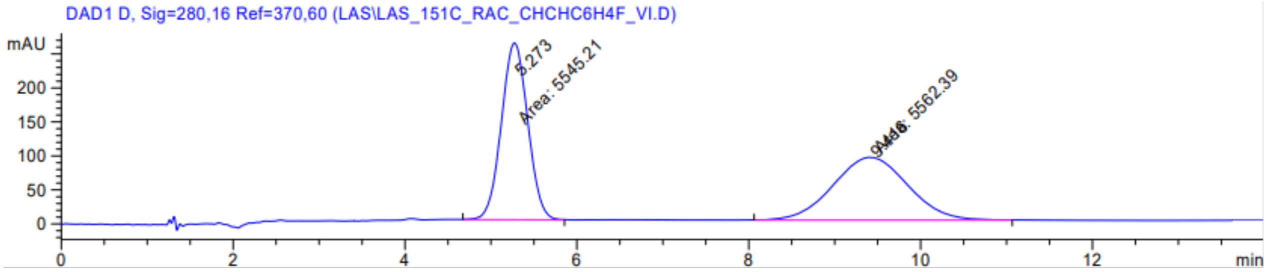

Signal 3: DAD1 D, Sig=280,16 Ref=370,60

Peak #	RetTime [min]	Type	Width [min]	Area [mAU*s]	Height [mAU]	Area %
1	5.273	MM	0.3555	5545.20654	259.98407	49.9226
2	9.416	MM	1.0051	5562.39063	92.23834	50.0774
Totals :				1.11076e4	352.22241	

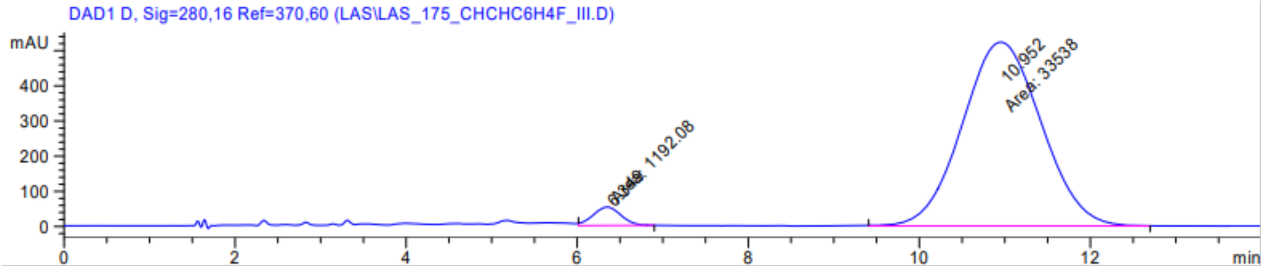

Signal 3: DAD1 D, Sig=280,16 Ref=370,60

Peak #	RetTime [min]	Type	Width [min]	Area [mAU*s]	Height [mAU]	Area %
1	6.349	MM	0.3749	1192.08069	52.99109	3.4324
2	10.952	MM	1.0693	3.35380e4	522.74005	96.5676
Totals :				3.47301e4	575.73114	

Product Characterization: Rearomatized product

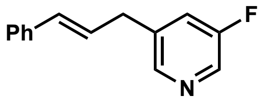

4a
Chemical Formula: $C_{14}H_{12}FN$
Exact Mass: 213.0954
Molecular Weight: 213.2554

3-cinnamyl-5-fluoropyridine 4a was synthesized as followed. In a 2 mL screw vial equipped with a magnetic stir bar was then transferred to an argon filled glovebox. Chlor-bis-(cycloocten)-iridium(I) dimer ($[Ir(coe)_2Cl]_2$, 2.3 mg, 0.0025 mmol, 0.5 mol%) was added to the vial, closed with a septum screw cap and transferred out of the glovebox. Diethyl silane (Et_2SiH_2, 97 µL, 0.75 mmol, 1.5 equiv.) was added and the resulting mixture was stirred at room temperature for 4 minutes. 3-Fluoro pyridine (43 µL, 0.5 mmol, 1.0 equiv.) was added and the resulting reaction mixture was stirred at 50 °C for 5 h. A separate 10 mL screw vial equipped with a magnetic stir bar was transferred to the glovebox. Ligand **L3** (24.2 mg, 0.035 mmol, 7 mol%) and palladium(II) acetate ($Pd(OAc)_2$, 5.6 mg, 0.025 mmol, 5 mol%) were added. The vial was closed with a septum screw cap and transferred out of the glovebox. Dichloromethane (CH_2Cl_2, 2.5 mL) was added at room temperature under nitrogen. The mixture was stirred for 5 minutes Cinnamyl methyl carbonate (192.2 mg, 1.0 mmol, 2.0 equiv.) was added, followed by the reaction mixture from the 2 mL vial. The resulting mixture was then stirred at 40 °C for 16 h. Afterwards, the reaction mixture was cooled to room temperature and the solvent was evaporated. The residue was submitted to flash column chromatography over silica gel using a solvent mixture of hexane/ethyl acetate = 8/2 as the eluent. The desired product **4a** was obtained as colorless oil (30.1 mg, 0.14 mmol, 28% yield).

^{1}H NMR (400 MHz, $CDCl_3$): δ 8.36 – 8.33 (m, 2H), 7.38 – 7.34 (m, 2H), 7.34 – 7.27 (m, 3H), 7.26 – 7. 21 (m, 1H), 6.49 (d, *J* = 15.8 Hz, 1H), 6.29 (dt, *J* = 15.8, 6.9 Hz, 1H), 3.58 (d, 6.7 Hz, 2H);

^{13}C NMR (101 MHz, $CDCl_3$): δ 159.72 (d, *J* = 256.8 Hz), 146.00 (d, *J* = 3.7 Hz), 137.54 (d, *J* = 3.2 Hz), 136.91, 136.22 (d, *J* = 23.1 Hz), 132.78, 128.76, 127.74, 126.86, 126.34, 123.01 (d, *J* = 17.8 Hz), 36.00 (d, *J* = 1.4 Hz);

^{19}F NMR (282 MHz, $CDCl_3$): δ -127.24 (dd, *J* = 9.3, 1.7 Hz);

HRMS: m/z (ESI) calculated for $(M + H)^+$: 214.1032, found: 214.1005;

R_f (Hexane/EtOAc = 8/2): 0.47;

ATR-IR ν (cm^{-1}): 3734, 3029, 2922, 1599, 1576, 1496, 1448, 1431, 1259, 1144, 1026, 965, 879, 808, 780, 754, 697, 676.

Synthesis of Free NH Product 3

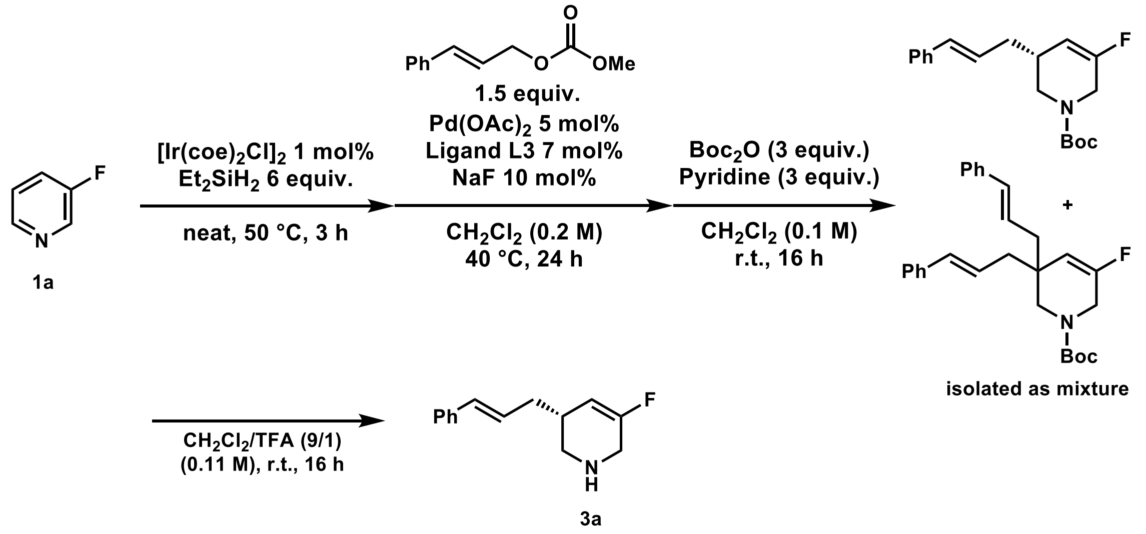

*Scheme 1. Synthetic route to free N-H product **3a**.*

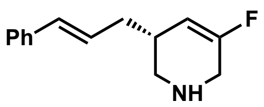

3a
Chemical Formula: $C_{14}H_{16}FN$
Exact Mass: 217.1267
Molecular Weight: 217.2874

(R)-3-cinnamyl-5-fluoro-1,2,3,6-tetrahydropyridine 3a was synthesized in a following the synthetic sequence in Scheme S6. A 10 mL screw vial was transferred to an argon filled glovebox. Chlor-bis-(cycloocten)-iridium(I) dimer ($[Ir(coe)_2Cl]_2$, 22.4 mg, 0.025 mmol, 1 mol%) was added to the vial. The vial was closed with a septum screw cap. The vial was transferred out of the glovebox. Diethyl silane (Et_2SiH_2, 1.94 mL, 15.0 mmol, 6.0 equiv.) was added and the resulting mixture was stirred at room temperature for 4 minutes. 3-Fluoropyridine (215 µL, 2.5 mmol, 1.0 equiv.) was added and the resulting reaction mixture was stirred at 50 °C for 3 hours. A separate 25 mL screw vial equipped with a magnetic stir bar was transferred to the glovebox. Ligand **L3** (120.8 mg, 0.175 mmol, 7 mol%), NaF (10.5 mg, 0.25 mmol, 10 mol%) and palladium(II) acetate ($Pd(OAc)_2$, 28.1 mg, 0.125 mmol, 5 mol%) were added. The vial was closed with a septum screw cap and transferred out of the glovebox. Dichloromethane (CH_2Cl_2, 12.5 mL) was added at room temperature under nitrogen. The mixture was stirred for 5 minutes. Cinnamyl methyl carbonate (720.8 mg, 3.75 mmol, 1.5 equiv.) was added, followed by the reaction mixture from the 10 mL vial. The resulting mixture was then stirred at 40 °C for 24 h. The mixture was then cooled to room temperature and diluted with additional dichloromethane (10 mL). Pyridine (604 µL, 7.5 mmol, 3.0 equiv.) was added as a base, followed by di-tert-butyl dicarbonate (1.72 mL, 7.5 mmol, 3.0 equiv.). The mixture was stirred at room temperature for 16 h and then transferred to a 50 mL flask and diluted with MeOH (20 mL). Imidazole (510 mg, 7.5 mmol, 3.0 equiv.) were added and the mixture was stirred for 2 hours to decompose residual Boc_2O. The solvent was evaporated and the crude subjected to flash column chromatography over silica gel using a solvent mixture of hexane/ethyl acetate (20/1) as the eluent. An inseparable mixture of the mono-alkylated and bisalkylated N-Boc compounds were isolated as colorless oil and directly used for the deprotection step. The mixture was redissolved in CH_2Cl_2 (20 mL) at room temperature. TFA (2mL) was added dropwise and the resulting solution was stirred at room temperature for 16 hours. An aqueous workup with 4M NaOH was performed to neutralize the

TFA. The aqueous phase was extracted with EtOAc three times and the combined organic phases were dried over magnesium sulfate and filtered. The mono-alkylated NH tetrahydropyridine **3a** was purified by flash column chromatography over silica gel using a solvent mixture of hexane/acetone (7/3 to 1/1) as the eluent. The desired compound was isolated as colorless oil (122 mg, 0.56 mmol, 22% yield over 4 steps). The absolute configuration was adopted from product **6a**. The enantioselectivity was not again measured for the free N-H product **3a**.

^{1}H NMR (400 MHz, $CDCl_3$): δ 7.37 – 7.28 (m, 4H), 7.24 – 7.19 (m, 1H), 6.46 – 6.40 (m, 1H), 6.17 (dt, *J* = 15.8, 7.3 Hz, 1H), 5.29 (ddt, *J* = 17.7, 3.1, 1.5 Hz, 1H), 3.42 – 3.30 (m, 2H), 3.03 (ddd, *J* = 12.9, 4.9, 2.1 Hz, 1H), 2.56 (ddd, *J* = 12.9, 7.1, 1.0 Hz, 1H), 2.43 (tqt, *J* = 7.4, 5.1, 2.5 Hz, 1H), 2.32 – 2.19 (m, 2H), 2.11 (bs, 1H);

^{13}C NMR (101 MHz, $CDCl_3$): δ 159.12 (d, *J* = 261.9 Hz), 137.45, 132.20, 128.67, 127.63, 127.30, 126.16, 104.91 (d, *J* = 11.0 Hz), 48.13 (d, *J* = 1.7 Hz), 44.20 (d, *J* = 30.1 Hz), 37.54 (d, *J* = 2.2 Hz), 35.10 (d, *J* = 4.8 Hz);

^{19}F NMR (282 MHz, $CDCl_3$): δ -110.91 (dd, *J* = 17.5, 5.3 Hz);

HRMS: m/z (ESI) calculated for $(M + H)^+$: 218.1345, found: 218.1343;

R_f (Hexane/acetone = 7/3): 0.23;

ATR-IR ν (cm^{-1}): 3056, 3025, 2914, 2844, 1700, 1598, 1494, 1448, 1367, 1279, 1156, 1108, 1070, 1047, 1029, 966, 910, 841, 744, 694;

$[\alpha]_D^{25}$: – 8.24 (c 0.9, $CHCl_3$).

General Procedure: Alternative route to free NH product 3a

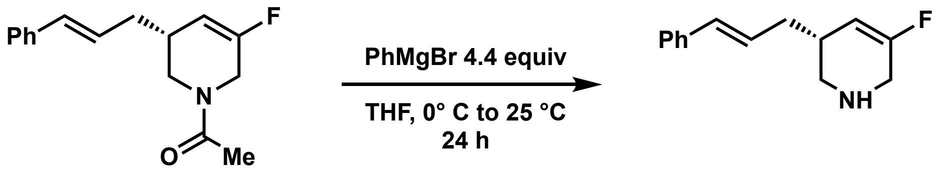

6a ***3a***

*Scheme 2. Alternative route to **3a**.*

(R)-3-cinnamyl-5-fluoro-1,2,3,6-tetrahydropyridine 3a was synthesized following the synthetic sequence in Scheme S7. (R)-1-(3-cinnamyl-5-fluoro-3,6-dihydropyridin-1(2H)-yl)ethan-1-one (26 mg, 0.1 mmol, 1.0 equiv.) was dissolved in tetrahydrofuran (THF, 180 µL) in a 4 mL screw cap vial equipped with a septum screw cap. The reaction mixture was cooled to 0 °C and a solution of phenyl magnesium bromide (PhMgBr 1 M in THF, 440 µL, 0.44 mmol, 4.4 equiv.) was added slowly at 0 °C. The reaction was allowed to warm to ambient temperature and stirred for 24 hours. The reaction mixture was then cooled to 0 °C and an aqueous workup with 2 M HCl (2.0 mL) was added dropwise. The acidic aqueous layer was extracted with EtOAc (3 x 5 mL) to remove any organic impurities. The acidic aqueous phase was then basified with

$NaHCO_3$ and the resulting aqueous phase was then extracted with EtOAc (3 x 5 mL). The resulting organic layer was dried with Na_2SO_4 and filtered and the solvent was evaporated and the mono-alkylated NH tetrahydropyridine **3a** was purified by flash column chromatography over silica gel using a solvent mixture of hexane/acetone (7/3 to 1/1) as the eluent. The desired compound was isolated as colorless oil (17.4 mg, 0.08 mmol, 80% yield). The enantioselectivity was not again measured for the free N-H product **3a**. The spectra match the spectra of free amine **3a** using the method described in scheme 6. *Note: some product was observed in the organic layer prior to basification, product can be obtained by flash column chromatography over silica gel using a solvent mixture of hexane/acetone (7/3 to 1/1) as the eluent.*

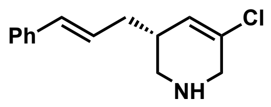

3b
Chemical Formula: $C_{14}H_{16}ClN$
Exact Mass: 233.0971
Molecular Weight: 233.7390

(R)-5-chloro-3-cinnamyl-1,2,3,6-tetrahydropyridine 3b was synthesized following the procedure reported in Scheme S7 (R)-1-(3-cinnamyl-5-chloro-3,6-dihydropyridin-1(2H)-yl)ethan-1-one (27.5 mg, 0.1 mmol, 1.0 equiv.). The desired compound was obtained as colourless oil (20.5 mg, 0.088 mmol, 88% yield). The absolute configuration was adopted from product **6b**. The enantioselectivity was not again measured for the free N-H product **3b**.

^{1}H NMR (400 MHz, $CDCl_3$): δ 7.38 – 7.28 (m, 4H), 7.25 – 7.20 (m, 1H), 6.44 (d, J = 16.0 Hz, 1H), 6.22 – 6.10 (m, 1H), 5.90 (dt, J = 3.5, 1.7 Hz, 1H), 3.50 – 3.34 (m, 2H), 3.09 (dd, J = 13.1, 5.1 Hz, 1H), 2.61 (dd, J = 13.1, 7.4 Hz, 1H), 2.43 (ddq, J = 10.1, 5.0, 2.7 Hz, 1H), 2.26 (tdd, J = 7.4, 4.0, 1.3 Hz, 1H);

^{13}C NMR (101 MHz, $CDCl_3$) 137.36, 132.48, 131.88, 128.71, 127.41, 127.22, 127.05, 126.21, 50.14, 47.43, 37.54, 37.08;

HRMS: m/z (ESI) calculated for $(M + H)^+$: 234.1044, found 234.1048;

R_f (*n*-hexane/acetone = 1/1): 0.35;

ATR-IR ν (cm^{-1}): 3026, 2919, 2851, 1654, 1449, 969, 749, 697;

$[\alpha]_D^{25}$: – 13.39 (c 0.93, $CHCl_3$);

Follow up chemistry and product derivatization

Cross coupling reactions of 6b

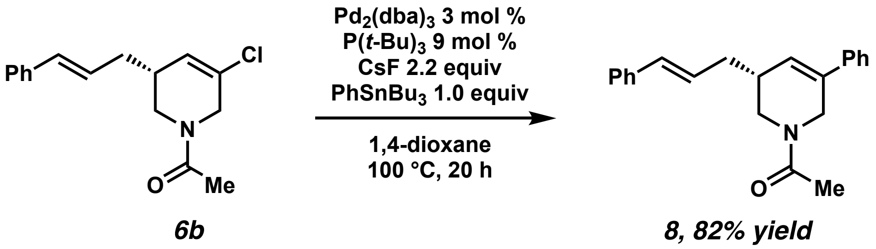

Scheme S8. Stille Cross Coupling of vinyl chloride ***6b****.*

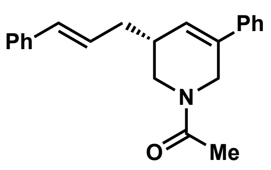

8

Chemical Formula: $C_{22}H_{23}NO$
Exact Mass: 317.1780
Molecular Weight: 317.4320

(*R*)-1-(3-cinnamyl-5-phenyl-3,6-dihydropyridin-1(2*H*)-yl)ethan-1-one 8 was synthesized following the synthetic sequence in Scheme S8. A 4 mL screw cap vial was transferred to an argon filled glovebox. Bis(dibenzylideneacetone)palladium(0) (Pd_2dba_3, 3 mg, 0.003 mmol, 3 mol%) was added to a vial with tri-*tert*-butylphosphine (P(*t*-Bu)$_3$, 2 mg, 0.009 mmol, 9 mol%) and cesium fluoride (CsF, 33 mg, 0.22 mmol, 2.2 equiv.). Dioxane (200 μL) was added to mixture and stirred at ambient temperature for 5 minutes. (R)-1-(3-cinnamyl-5-chloro-3,6-dihydropyridin-1(2H)-yl)ethan-1-one (27.5 mg, 0.1 mmol, 1.0 equiv.) was dissolved in dioxane (200 μL) and added to the reaction mixture followed by tributylphenylstannane ($PhSnBu_3$, 34 μL, 0.1 mmol, 1.0 equiv.). The reaction mixture was sealed with a septum screw cap and heated to 100 °C for 20 hours. The reaction mixture was cooled to room temperature, filtered through a plug of silica and washed with copious amounts of ethyl acetate. The solvent was evaporated and the crude reaction mixture was purified by flash chromatography over silica gel using a solvent mixture of hexane/EtOAc (7/3 to 2/8) as the eluent. The desired compound was isolated as a pale-yellow oil (26.1 mg, 0.082 mmol, 82% yield). The absolute configuration was adopted from product **6b**. The enantioselectivity was not again measured for the cross-coupled product **8**.

^{1}H NMR (400 MHz, CDCl$_3$) (as mixture of E/Z amide bond isomers): δ 7.48 – 7.28 (m, 9H), 7.26 – 7.19 (m, 1H), 6.55 – 6.44 (m, 1H), 6.32 – 6.23 (m, 1H), 6.23 – 6.14 (m, 1H), 4.54 (dt, *J* = 18.0, 2.2 Hz, 0.65H), 4.39 (dt, *J* = 18.1, 2.3 Hz, 0.65H), 4.30 (dt, *J* = 4.8, 2.4 Hz, 0.7H), 4.14 (dd, *J* = 12.8, 5.0 Hz, 0.35H), 3.67 (dd, *J* = 13.2, 4.7 Hz, 0.65H), 3.40 – 3.30 (m, 0.65H), 3.22 (dd, *J* = 12.8, 7.5 Hz, 0.35H), 2.68 – 2.55 (m, 1H), 2.46 – 2.29 (m, 2H), 2.18 (s, 1H), 2.18 (s, 1.1H), 2.17 (s, 1.9H);

^{13}C NMR (101 MHz, CDCl$_3$) (as mixture of E/Z amide bond isomers, signals of minor isomer are indicated with *)**:** δ 169.9, 169.7*, 138.82*, 138.7, 137.5, 137.2*, 135.2, 133.8*, 132.8, 132.5*, 128.8*, 128.8, 128.7 (overlap)*, 128.1*, 128.0*, 127.9, 127.6, 127.3*, 127.3 (overlap)*, 127.3, 126.3*, 126.2, 125.8*, 125.5 (overlap)*, 125.2, 47.6, 47.6*, 43.5, 43.0*, 37.0*, 36.8, 36.4*, 35.5, **29.8**, 22.2*, 21.8; (bold is impurity)

HRMS: m/z (ESI) calculated for $(M + H)^+$: 318.1852, found 318.1867;

R$_f$ (Hexane/acetone = 3/7): 0.4;

ATR-IR ν (cm^{-1}): 3027, 2924, 2853, 1625, 1447, 1317, 1269, 1033, 970, 751, 696, 689, 665;

$[\alpha]_D^{25}$: – 5.14 (c 1.0, $CHCl_3$).

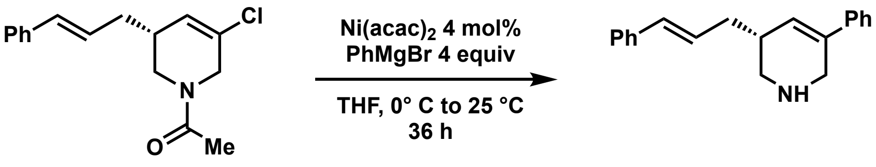

*Scheme 9. Kumada-type Cross Coupling of vinyl chloride **6b**.*

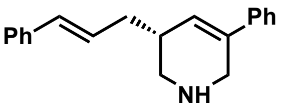

9
Chemical Formula: $C_{20}H_{21}N$
Exact Mass: 275.1674
Molecular Weight: 275.3950

(*R*)-3-cinnamyl-5-phenyl-1,2,3,6-tetrahydropyridine 9 was synthesized following the synthetic sequence in Scheme S9. A 4 mL screw cap vial was charged with nickel(II) acetylacetonate (Ni(acac)$_2$, 1.0 mg, 0.004 mmol, 4 mol%) and dissolved in tetrahydrofuran (THF, 100 µL). The reaction mixture was cooled to 0 °C, and phenyl magnesium bromide (PhMgBr 1 M in THF, 400 µL, 0.4 mmol, 4.0 equiv.) was added to the reaction mixture dropwise. (R)-1-(3-cinnamyl-5-chloro-3,6-dihydropyridin-1(2H)-yl)ethan-1-one (27.5 mg, 0.1 mmol, 1.0 equiv.) was dissolved in tetrahydrofuran (THF, 200 µL) and added to the reaction mixture at 0 °C and allowed to warm to ambient temperature overnight. After 36 hours, the reaction was cooled to 0 °C and an aqueous workup was performed with saturated NH_4Cl (1 mL). The aqueous phase was extracted with EtOAc (3 x 10 mL) and the organic layer was dried over Na_2SO_4 and filtered. The solvent was evaporated and the crude reaction mixture was purified by flash chromatography over silica gel using a solvent mixture of methanol/ethyl acetate (1/9) with 1% triethyl amine as the eluent. The desired compound was isolated as a pale-yellow amorphous solid (22.8 mg, 0.083 mmol, 83% yield). The absolute configuration was adopted from product **6b**. The enantioselectivity was not again measured for the cross-coupled product **9**.

^{1}H NMR (400 MHz, CDCl$_3$) ^{1}H NMR (400 MHz, CDCl$_3$) δ 7.39 – 7.27 (m, 8H), 7.22 (d, *J* = 7.0 Hz, 2H), 6.51 (d, *J* = 15.7 Hz, 1H), 6.21 (s, 1H), 6.15 (dd, *J* = 15.5, 7.5 Hz, 1H), 4.07 (d, *J* = 16.4 Hz, 1H), 3.98 (d, *J* = 16.5 Hz, 1H), 3.56 (dd, *J* = 12.3, 5.4 Hz, 1H), 3.06 (s, 1H), 2.88 (t, *J* = 10.9 Hz, 1H), 2.44 (t, *J* = 6.9 Hz, 2H).
Et_3N•HCl present ^{1}H NMR (400 MHz, CDCl$_3$) δ 3.13 (q, *J* = 7.3 Hz, 2H) 1.44 – 1.34 (m, 3H).
*Note: Titration of amine **9** with Et_3N resulted in better resolution in peaks due to solubility issues of **9** in CDCl$_3$*

^{1}H NMR (400 MHz, CDCl$_3$) δ 7.39 – 7.28 (m, 8H), 7.25 – 7.19 (m, 2H), 6.48 (d, *J* = 15.8 Hz, 1H), 6.22 (dt, *J* = 15.8, 7.2 Hz, 1H), 6.16 (s, 1H), 3.92 – 3.75 (m, 2H), 3.36 – 3.26 (m, 1H), 2.79 – 2.63 (m, 2H), 2.41 – 2.31 (m, 2H). without Et_3N trituration.

^{13}C NMR (101 MHz, CDCl$_3$) ^{13}C NMR (101 MHz, CDCl$_3$) δ 137.11, 136.92, 133.75, 130.70, 128.93, 128.75, 128.60, 127.69, 126.48, 126.36, 125.44, 125.30, 44.90, 43.25, 36.66, 32.63,
Et_3N•HCl present ^{13}C NMR (101 MHz, CDCl$_3$) δ 46.07, 8.78
*Note: Titration of amine **9** with Et_3N resulted in better resolution in peaks due to solubility issues of **9** in CDCl$_3$*
^{13}C NMR (101 MHz, CDCl$_3$) δ 138.93, 137.36, 134.68,p 132.64, 128.70, 128.69, 127.83, 127.41, 127.16, 126.84, 126.25, 125.17, 46.98, 45.58, 37.32, 34.65. without Et_3N trituration.

HRMS: m/z (ESI) calculated for $(M + H)^+$: 276.1747, found 276.1755;

R$_f$ (methanol/ethyl acetate = 1/9): 0.4;

ATR-IR ν (cm^{-1}): 2924, 1634, 1447, 969, 738, 751, 694;

$[\alpha]_D^{25}$: – 6.80 (c 0.833, $CHCl_3$);

Ring-closing metathesis

Synthesis of 10a

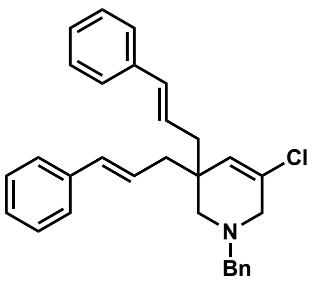

10a
Chemical Formula: $C_{30}H_{30}ClN$
Exact Mass: 439.2067
Molecular Weight: 440.0270

1-Benzyl-5-chloro-3,3-dicinnamyl-1,2,3,6-tetrahydropyridine 10a Benzyl bromide (247 µL, 2.07 mmol, 3.0 equiv.) and triethyl amine (96 µL, 0.69 mmol, 1.0 equiv.) were added to 5-chloro-3,3-bis((*E*)-3-(phenyl)allyl)-1,2,3,6-tetrahydropyridine (**5b**, 242 mg, 0.69 mmol, 1.0 equiv.) in dichloromethane (4 mL). The resulting reaction mixture was stirred at room temperature for 19 h. The reaction was quenched with a saturated aqueous ammonium chloride solution (2 mL). The phases were separated and the aqueous phase was extracted with dichloromethane (3 x 2 mL). The combined organic phases were washed with brine (1 x 2 mL), dried over sodium sulphate, and concentrated under reduced pressure. Purification of the residue by flash chromatography on silica gel using *n*-hexane/ethyl acetate gave the title compound **10a** (260 mg, 0.59 mmol, 86%) as colourless oil.

^{1}H NMR (400 MHz, $CDCl_3$): δ 7.38–7.27 (m, 13H), 7.25–7.19 (m, 2H), 6.36 (d, *J* = 15.8 Hz, 2H), 6.13 (dt, *J* = 15.4, 7.4 Hz, 2H), 5.79 (s, 1H), 3.57 (s, 2H), 3.10 (s, 2H), 2.43–2.25 (m, 6H);

^{13}C NMR (101 MHz, $CDCl_3$) *(one carbon signal is overlapping)*: δ 138.3, 137.6, 133.2, 130.2, 129.2, 129.1, 128.6, 128.5, 127.4, 127.3, 126.2, 126.0, 62.6, 58.8, 58.0, 42.8, 41.2;

HRMS: m/z (ESI) calculated for $(M + H)^+$: 440.2145, found: 440.2150;

R$_f$ (*n*-hexane/EtOAc = 10/1): 0.58;

ATR-IR ν (cm^{-1}): 3028, 2922, 2853, 1681, 1495, 1452, 1265, 1073, 1028, 970, 826, 738, 699, 676;

Ring-closing metathesis of 10a

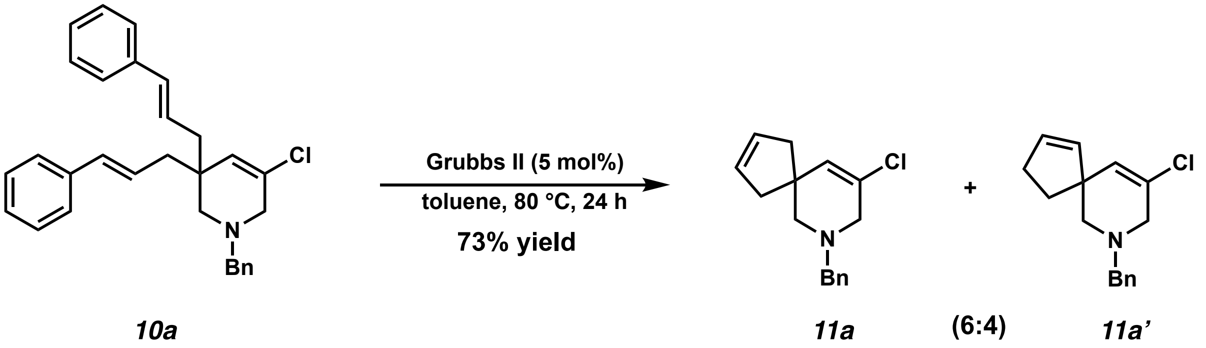

Scheme 10. Ring closing metathesis of 10a

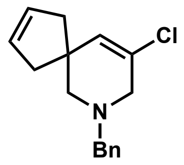

11a

Chemical Formula: $C_{16}H_{18}ClN$
Exact Mass: 259.1128
Molecular Weight: 259.7770

1-Benzyl-5-chloro-3,3-dicinnamyl-1,2,3,6-tetrahydropyridine 11a. A solution of 1-benzyl-5-chloro-3,3-dicinnamyl-1,2,3,6-tetrahydropyridine **10a** (50 mg, 0.11 mmol, 1.0 equiv.) in toluene (1 mL) was added to a 10-mL screw vial charged with dichloro[1,3-bis(2,4,6-trimethylphenyl)-2-imidazolidinylidene](benzylidene)-(tricyclohexylphosphine)ruthenium(II) (Grubbs II, 4.8 mg, 5.7 µmol, 5 mol%) under a nitrogen atmosphere. The resulting mixture was stirred at 80°C for 24 hours. The reaction mixture was cooled to room temperature and filtered through a small plug silica gel. The volatiles were remover under reduced pressure. Purification of the residue by flash chromatography on silica gel using *n*-hexane/ethyl acetate (100:0 to 20:1) gave the title compound **11a** in mixture with the isomer **11a'** (21 mg, 0.081 mmol, 73%, ratio 6:4 for **11a**:**11a'**) as colourless oil.

^{1}H NMR (400 MHz, $CDCl_3$): δ 7.31 (dd, J = 13.6, 6.6 Hz, 4H), 7.27 – 7.21 (m, 1H), 5.81 (s, 0.4H), 5.74 (d, J = 5.9 Hz, 0.4H), 5.65 (s, 0.4H), 5.59 – 5.54 (m, 1.2H), 5.31 – 5.30 (m, 1H), 3.57 (s, 2H), 3.04 (s, 2H), 2.34 (d, J = 45.6 Hz, 5H), 1.85 – 1.68 (m, 1H);

^{13}C NMR (101 MHz, $CDCl_3$): δ 138.7, 136.5, 132.6, 131.8, 130.6, 129.3, 129.2, 128.6, 128.1, 128.0, 127.5, 127.5, 62.3, 62.2, 61.3, 59.4, 58.2, 57.9, 53.1, 45.9, 45.0, 35.5, 31.3;

HRMS: m/z (ESI) calculated for $(M + H)^+$: 260.1206, found: 260.1226;

R_f (*n*-hexane/EtOAc = 20/1): 0.07;

ATR-IR ν (cm^{-1}): 3028, 1917, 2840, 2798, 1654, 1494, 1457, 1312, 1148, 1064, 967, 848, 741, 698, 680.

X ray crystallographic analysis

Synthesis of 7a

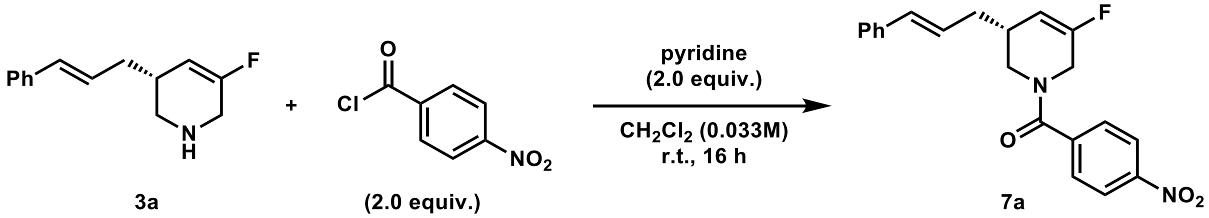

Scheme 11. Derivatization for crystallization.

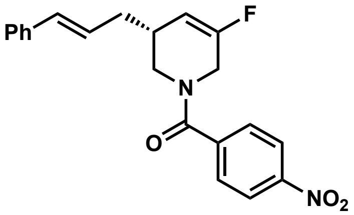

7a

Chemical Formula: $C_{21}H_{19}FN_2O_3$
Exact Mass: 366.1380
Molecular Weight: 366.3924

(R)-(3-cinnamyl-5-fluoro-3,6-dihydropyridin-1(2H)-yl)(4-nitrophenyl)methanone 7a was synthesized following the reaction in Scheme 9. Therefore, (R)-3-cinnamyl-5-fluoro-1,2,3,6-tetrahydropyridine (35.9 mg, 0.165 mmol, 1.0 equiv.) was dissolved in CH_2Cl_2 (5 mL) under air at room temperature. Pyridine (26.6 µL, 0.33 mmol, 2.0 equiv.) was added, followed by 4-nitrobenzoyl chloride (61.2 mg, 0.33 mmol, 2.0 equiv.). The resulting mixture was stirred at room temperature for 16 hours and then quenched with brine. The aqueous phase was extracted with EtOAc. The combined organic phases were dried over magnesium sulfate and filtered. The crude was subjected to flash column chromatography over silica gel using a solvent mixture of hexane/EtOAc (7/3) as the eluent. The desired compound was obtained as white solids (60.0 mg, 0.164 mmol, 99%).

^{1}H NMR (400 MHz, CD_3OD): (as mixture of E/Z amide bond isomers) δ 8.33 – 8.19 (m, 2H), 7.71 – 7.60 (m, 2H), 7.42 – 7.12 (m, 5H), 6.53 (d, J = 15.8 Hz, 0.3H), 6.36 – 6.25 (m, 0.3H), 6.20 (d, J = 15.9 Hz, 0.7H), 6.01 – 5.90 (dt, J = 15.2 Hz, 0.7H), 5.48 (d, J = 16.2 Hz, 1H), 4.38 – 4.24 (m, 1.4H), 4.00 – 3.92 (m, 0.6H), 3.90 – 3.68 (m, 0.7H), 3.52 (dd, J = 13.6, 4.6 Hz, 0.7H), 3.27 (dd, J = 13.7, 6.1 Hz, 0.6H), 2.68 – 2.48 (m, 1H), 2.39 –2.21 (m, 1.3H), 2.13 – 2.03 (m, 0.7H);

^{13}C NMR (101 MHz, CD_3OD): (as mixture of E/Z amide bond isomers, signals of minor isomer are indicated with *) δ 170.77, 170.49*, 156.51 (d, J = 253.8 Hz), 156.07 (d, J = 252.8 Hz)*, 150.05*, 149.83, 142.70, 142.61*, 138.80*, 138.29, 133.89*, 133.67, 129.55*, 129.47, 129.46, 129.31*, 128.32, 127.77*, 127.61, 127.21*, 126.91, 124.91, 106.76 (d, J = 10.1 Hz)*, 106.16 (d, J = 12.1 Hz), 47.15 (d, J = 40.3 Hz)*, 44.67, 42.16 (d, J = 40.8 Hz), 37.98*, 36.95, 35.34 (d, J = 6.3 Hz), 34.65*;

^{19}F NMR (282 MHz, CD_3OD): (as mixture of E/Z amide bond isomers, signal of minor isomer is indicated with *) δ -114.94 (dd, J = 16.2, 4.9 Hz), -115.88 – -116.01 (m)*;

HRMS: m/z (ESI) calculated for $(M + H)^+$: 367.1458, found: 367.1483;

R_f (Hexane/EtOAc = 7/3): 0.35;

ATR-IR ν (cm^{-1}): 3027, 2913, 2856, 1709, 1643, 1601, 1521, 1495, 1434, 1380, 1347, 1380, 1347, 1314, 1286, 1263, 1179, 1132, 1107, 1030, 1012, 969, 863, 853, 772, 742, 696.

Crystallization procedure

Crystals suitable for X-ray crystallographic analysis were obtained by dissolving the white solids (60.0 mg, 0.164 mmol) in hot Methanol (~4 mL). The solvent was allowed to slowly evaporate over 48 hours to obtain white crystals.
CCDC#2234165.

The crystal structure of **7a** was registered in the Cambridge crystallographic data center and can be found as CCDC#2234165.

Experimental details

Low-temperature diffraction data (ϕ-and ω-scans) were collected on a Bruker AXS D8 VENTURE KAPPA diffractometer coupled to a PHOTON II CPAD detector with Cu K_α radiation (λ = 1.54178 Å) from an IμS micro-source for the structure of compound V20222. The structure was solved by direct methods using SHELXS (Sheldrick, G. M. *Acta Cryst.* **1990**, A46, 467-473.) and refined against F^2 on all data by full-matrix least squares with SHELXL-2017 (Sheldrick, G. M. *Acta Cryst.* **2015**, C71, 3-8.) using established refinement techniques (Müller, P. *Crystallography Reviews* **2009**, *15*, 57-83). All non-hydrogen atoms were refined anisotropically. All hydrogen atoms were included into the model at geometrically calculated positions and refined using a riding model. The isotropic displacement parameters of all hydrogen atoms were fixed to 1.2 times the *U* value of the atoms they are linked to (1.5 times for methyl groups).

Compound **7a** (internal compound number **V20222**) crystallizes in the triclinic space group *P*1 with two molecules in the asymmetric unit.

Table 1. Crystal data and structure refinement for V20222.

Identification code	V20222	
Empirical formula	C42 H38 F2 N4 O6	
Formula weight	732.76	
Temperature	106(2) K	
Wavelength	1.54178 Å	
Crystal system	Triclinic	
Space group	P1	
Unit cell dimensions	a = 7.5776(5) Å	α= 71.507(6)°.
	b = 9.8771(9) Å	β= 76.607(4)°.
	c = 13.5619(9) Å	γ = 70.196(8)°.
Volume	896.88(13) $Å^3$	
Z	1	
Density (calculated)	1.357 Mg/m^3	

Absorption coefficient	0.815 mm^{-1}
F(000)	384
Crystal size	0.250 x 0.150 x 0.100 mm^3
Theta range for data collection	3.470 to 74.499°.
Index ranges	-9<=h<=9, -12<=k<=12, -16<=l<=16
Reflections collected	29009
Independent reflections	7038 [R(int) = 0.0265]
Completeness to theta = 67.679°	99.7 %
Absorption correction	Semi-empirical from equivalents
Max. and min. transmission	0.7538 and 0.6794
Refinement method	Full-matrix least-squares on F^2
Data / restraints / parameters	7038 / 3 / 487
Goodness-of-fit on F^2	1.039
Final R indices [I>2sigma(I)]	R1 = 0.0251, wR2 = 0.0666
R indices (all data)	R1 = 0.0252, wR2 = 0.0666
Absolute structure parameter	0.05(2)
Extinction coefficient	n/a
Largest diff. peak and hole	0.162 and -0.158 e.$Å^{-3}$

Table 2. Atomic coordinates (x 10^4) and equivalent isotropic displacement parameters ($Å^2$x 10^3) for V20222. U(eq) is defined as one third of the trace of the orthogonalized U^{ij} tensor.

	x	y	z	U(eq)
N(1)	3744(2)	7545(2)	8229(1)	20(1)
C(6)	2563(2)	6705(2)	8395(1)	21(1)
C(11)	3068(2)	5579(2)	7770(1)	20(1)
C(12)	3195(2)	4099(2)	8322(1)	23(1)
C(13)	3628(3)	3023(2)	7785(1)	25(1)
C(14)	3923(2)	3451(2)	6699(1)	23(1)
N(2)	4425(2)	2302(2)	6126(1)	28(1)
O(2)	4342(3)	1053(2)	6619(1)	44(1)
O(3)	4889(2)	2662(2)	5171(1)	39(1)
C(15)	3771(2)	4915(2)	6128(1)	23(1)
C(16)	3314(2)	5986(2)	6673(1)	23(1)
O(1)	1111(2)	6825(2)	9034(1)	29(1)
C(1)	3228(2)	8677(2)	8808(1)	22(1)
C(2)	4929(3)	8576(2)	9236(1)	23(1)
F(1)	4465(2)	9437(1)	9925(1)	31(1)
C(3)	6683(3)	7789(2)	9022(1)	25(1)
C(4)	7208(2)	6925(2)	8205(1)	22(1)
C(7)	7881(3)	5229(2)	8673(1)	25(1)
C(8)	8587(2)	4410(2)	7829(1)	24(1)
C(9)	8227(3)	3169(2)	7871(1)	25(1)
C(21)	8824(2)	2377(2)	7039(1)	25(1)
C(22)	8998(3)	857(2)	7296(2)	36(1)
C(23)	9488(3)	111(2)	6514(2)	44(1)
C(24)	9787(3)	851(3)	5475(2)	39(1)
C(25)	9631(3)	2360(3)	5206(2)	39(1)
C(26)	9158(3)	3116(2)	5983(2)	31(1)
C(5)	5584(2)	7403(2)	7559(1)	20(1)
N(101)	6170(2)	2464(2)	1720(1)	21(1)
C(106)	4459(2)	3365(2)	1472(1)	20(1)
C(111)	3397(2)	4513(2)	2074(1)	19(1)
C(112)	2685(2)	5983(2)	1504(1)	23(1)

C(113)	1732(2)	7092(2)	2015(1)	25(1)
C(114)	1482(2)	6691(2)	3104(1)	22(1)
N(102)	589(2)	7862(2)	3668(1)	27(1)
O(102)	-41(2)	9137(2)	3153(1)	37(1)
O(103)	538(2)	7498(2)	4625(1)	36(1)
C(115)	2064(2)	5231(2)	3691(1)	21(1)
C(116)	3030(2)	4138(2)	3167(1)	19(1)
O(101)	3728(2)	3288(2)	776(1)	31(1)
C(101)	7173(3)	1327(2)	1147(2)	26(1)
C(102)	9157(2)	1389(2)	772(1)	24(1)
F(101)	10032(2)	555(1)	69(1)	31(1)
C(103)	10047(2)	2107(2)	1049(1)	25(1)
C(104)	9074(2)	2969(2)	1864(1)	22(1)
C(107)	8688(2)	4663(2)	1375(1)	23(1)
C(108)	8056(2)	5529(2)	2185(1)	24(1)
C(109)	6543(2)	6705(2)	2183(1)	23(1)
C(121)	5883(2)	7606(2)	2952(1)	23(1)
C(122)	4483(3)	8971(2)	2726(2)	27(1)
C(123)	3865(3)	9880(2)	3413(2)	33(1)
C(124)	4645(3)	9453(2)	4329(2)	35(1)
C(125)	6019(3)	8099(3)	4568(2)	34(1)
C(126)	6613(3)	7175(2)	3896(1)	28(1)
C(105)	7264(2)	2550(2)	2451(1)	21(1)

Table 3. Bond lengths [Å] and angles [°] for V20222.

N(1)-C(6)	1.353(2)
N(1)-C(1)	1.4621(19)
N(1)-C(5)	1.467(2)
C(6)-O(1)	1.229(2)
C(6)-C(11)	1.505(2)
C(11)-C(12)	1.396(2)
C(11)-C(16)	1.397(2)
C(12)-C(13)	1.382(2)
C(12)-H(12)	0.9500
C(13)-C(14)	1.383(2)
C(13)-H(13)	0.9500
C(14)-C(15)	1.386(2)
C(14)-N(2)	1.472(2)
N(2)-O(2)	1.218(2)
N(2)-O(3)	1.228(2)
C(15)-C(16)	1.384(2)
C(15)-H(15)	0.9500
C(16)-H(16)	0.9500
C(1)-C(2)	1.494(2)
C(1)-H(1A)	0.9900
C(1)-H(1B)	0.9900
C(2)-C(3)	1.311(3)
C(2)-F(1)	1.3670(19)
C(3)-C(4)	1.510(2)
C(3)-H(3)	0.9500
C(4)-C(5)	1.532(2)
C(4)-C(7)	1.538(2)
C(4)-H(4)	1.0000
C(7)-C(8)	1.503(2)
C(7)-H(7A)	0.9900
C(7)-H(7B)	0.9900
C(8)-C(9)	1.325(3)
C(8)-H(8)	0.9500
C(9)-C(21)	1.475(2)

C(9)-H(9)	0.9500
C(21)-C(26)	1.394(3)
C(21)-C(22)	1.395(3)
C(22)-C(23)	1.391(3)
C(22)-H(22)	0.9500
C(23)-C(24)	1.372(3)
C(23)-H(23)	0.9500
C(24)-C(25)	1.387(3)
C(24)-H(24)	0.9500
C(25)-C(26)	1.392(3)
C(25)-H(25)	0.9500
C(26)-H(26)	0.9500
C(5)-H(5A)	0.9900
C(5)-H(5B)	0.9900
N(101)-C(106)	1.348(2)
N(101)-C(101)	1.464(2)
N(101)-C(105)	1.470(2)
C(106)-O(101)	1.232(2)
C(106)-C(111)	1.505(2)
C(111)-C(116)	1.394(2)
C(111)-C(112)	1.395(2)
C(112)-C(113)	1.385(3)
C(112)-H(112)	0.9500
C(113)-C(114)	1.387(3)
C(113)-H(113)	0.9500
C(114)-C(115)	1.383(2)
C(114)-N(102)	1.474(2)
N(102)-O(102)	1.221(2)
N(102)-O(103)	1.227(2)
C(115)-C(116)	1.385(2)
C(115)-H(115)	0.9500
C(116)-H(116)	0.9500
C(101)-C(102)	1.487(2)
C(101)-H(10A)	0.9900
C(101)-H(10B)	0.9900
C(102)-C(103)	1.307(3)

C(102)-F(101)	1.3653(18)
C(103)-C(104)	1.512(2)
C(103)-H(103)	0.9500
C(104)-C(105)	1.535(2)
C(104)-C(107)	1.542(2)
C(104)-H(104)	1.0000
C(107)-C(108)	1.497(2)
C(107)-H(10C)	0.9900
C(107)-H(10D)	0.9900
C(108)-C(109)	1.327(3)
C(108)-H(108)	0.9500
C(109)-C(121)	1.476(2)
C(109)-H(109)	0.9500
C(121)-C(126)	1.395(3)
C(121)-C(122)	1.399(3)
C(122)-C(123)	1.392(2)
C(122)-H(122)	0.9500
C(123)-C(124)	1.382(3)
C(123)-H(123)	0.9500
C(124)-C(125)	1.384(3)
C(124)-H(124)	0.9500
C(125)-C(126)	1.387(3)
C(125)-H(125)	0.9500
C(126)-H(126)	0.9500
C(105)-H(10E)	0.9900
C(105)-H(10F)	0.9900

C(6)-N(1)-C(1)	118.35(13)
C(6)-N(1)-C(5)	127.02(13)
C(1)-N(1)-C(5)	114.55(13)
O(1)-C(6)-N(1)	122.72(15)
O(1)-C(6)-C(11)	119.18(15)
N(1)-C(6)-C(11)	118.10(14)
C(12)-C(11)-C(16)	120.15(15)
C(12)-C(11)-C(6)	117.74(14)
C(16)-C(11)-C(6)	122.03(14)

C(13)-C(12)-C(11)	120.09(15)
C(13)-C(12)-H(12)	120.0
C(11)-C(12)-H(12)	120.0
C(12)-C(13)-C(14)	118.47(16)
C(12)-C(13)-H(13)	120.8
C(14)-C(13)-H(13)	120.8
C(13)-C(14)-C(15)	122.82(16)
C(13)-C(14)-N(2)	118.58(15)
C(15)-C(14)-N(2)	118.60(15)
O(2)-N(2)-O(3)	123.48(16)
O(2)-N(2)-C(14)	118.51(15)
O(3)-N(2)-C(14)	118.01(15)
C(16)-C(15)-C(14)	118.25(16)
C(16)-C(15)-H(15)	120.9
C(14)-C(15)-H(15)	120.9
C(15)-C(16)-C(11)	120.16(15)
C(15)-C(16)-H(16)	119.9
C(11)-C(16)-H(16)	119.9
N(1)-C(1)-C(2)	108.81(13)
N(1)-C(1)-H(1A)	109.9
C(2)-C(1)-H(1A)	109.9
N(1)-C(1)-H(1B)	109.9
C(2)-C(1)-H(1B)	109.9
H(1A)-C(1)-H(1B)	108.3
C(3)-C(2)-F(1)	120.93(16)
C(3)-C(2)-C(1)	127.66(16)
F(1)-C(2)-C(1)	111.40(14)
C(2)-C(3)-C(4)	120.60(16)
C(2)-C(3)-H(3)	119.7
C(4)-C(3)-H(3)	119.7
C(3)-C(4)-C(5)	110.36(14)
C(3)-C(4)-C(7)	112.79(14)
C(5)-C(4)-C(7)	112.91(13)
C(3)-C(4)-H(4)	106.8
C(5)-C(4)-H(4)	106.8
C(7)-C(4)-H(4)	106.8

C(8)-C(7)-C(4)	111.51(14)
C(8)-C(7)-H(7A)	109.3
C(4)-C(7)-H(7A)	109.3
C(8)-C(7)-H(7B)	109.3
C(4)-C(7)-H(7B)	109.3
H(7A)-C(7)-H(7B)	108.0
C(9)-C(8)-C(7)	125.00(16)
C(9)-C(8)-H(8)	117.5
C(7)-C(8)-H(8)	117.5
C(8)-C(9)-C(21)	126.33(16)
C(8)-C(9)-H(9)	116.8
C(21)-C(9)-H(9)	116.8
C(26)-C(21)-C(22)	118.15(17)
C(26)-C(21)-C(9)	121.81(16)
C(22)-C(21)-C(9)	119.99(17)
C(23)-C(22)-C(21)	120.51(19)
C(23)-C(22)-H(22)	119.7
C(21)-C(22)-H(22)	119.7
C(24)-C(23)-C(22)	120.96(19)
C(24)-C(23)-H(23)	119.5
C(22)-C(23)-H(23)	119.5
C(23)-C(24)-C(25)	119.29(19)
C(23)-C(24)-H(24)	120.4
C(25)-C(24)-H(24)	120.4
C(24)-C(25)-C(26)	120.3(2)
C(24)-C(25)-H(25)	119.9
C(26)-C(25)-H(25)	119.9
C(25)-C(26)-C(21)	120.82(18)
C(25)-C(26)-H(26)	119.6
C(21)-C(26)-H(26)	119.6
N(1)-C(5)-C(4)	111.53(13)
N(1)-C(5)-H(5A)	109.3
C(4)-C(5)-H(5A)	109.3
N(1)-C(5)-H(5B)	109.3
C(4)-C(5)-H(5B)	109.3
H(5A)-C(5)-H(5B)	108.0

C(106)-N(101)-C(101)	118.01(14)
C(106)-N(101)-C(105)	127.73(13)
C(101)-N(101)-C(105)	114.06(13)
O(101)-C(106)-N(101)	122.89(15)
O(101)-C(106)-C(111)	118.73(15)
N(101)-C(106)-C(111)	118.38(14)
C(116)-C(111)-C(112)	119.72(15)
C(116)-C(111)-C(106)	122.18(14)
C(112)-C(111)-C(106)	118.02(14)
C(113)-C(112)-C(111)	120.59(15)
C(113)-C(112)-H(112)	119.7
C(111)-C(112)-H(112)	119.7
C(112)-C(113)-C(114)	118.00(15)
C(112)-C(113)-H(113)	121.0
C(114)-C(113)-H(113)	121.0
C(115)-C(114)-C(113)	122.74(15)
C(115)-C(114)-N(102)	118.05(15)
C(113)-C(114)-N(102)	119.20(15)
O(102)-N(102)-O(103)	124.06(16)
O(102)-N(102)-C(114)	117.96(15)
O(103)-N(102)-C(114)	117.98(14)
C(114)-C(115)-C(116)	118.36(15)
C(114)-C(115)-H(115)	120.8
C(116)-C(115)-H(115)	120.8
C(115)-C(116)-C(111)	120.36(15)
C(115)-C(116)-H(116)	119.8
C(111)-C(116)-H(116)	119.8
N(101)-C(101)-C(102)	109.27(14)
N(101)-C(101)-H(10A)	109.8
C(102)-C(101)-H(10A)	109.8
N(101)-C(101)-H(10B)	109.8
C(102)-C(101)-H(10B)	109.8
H(10A)-C(101)-H(10B)	108.3
C(103)-C(102)-F(101)	121.33(15)
C(103)-C(102)-C(101)	127.62(15)
F(101)-C(102)-C(101)	111.05(14)

C(102)-C(103)-C(104)	120.31(16)
C(102)-C(103)-H(103)	119.8
C(104)-C(103)-H(103)	119.8
C(103)-C(104)-C(105)	110.90(14)
C(103)-C(104)-C(107)	111.40(14)
C(105)-C(104)-C(107)	112.08(13)
C(103)-C(104)-H(104)	107.4
C(105)-C(104)-H(104)	107.4
C(107)-C(104)-H(104)	107.4
C(108)-C(107)-C(104)	112.63(14)
C(108)-C(107)-H(10C)	109.1
C(104)-C(107)-H(10C)	109.1
C(108)-C(107)-H(10D)	109.1
C(104)-C(107)-H(10D)	109.1
H(10C)-C(107)-H(10D)	107.8
C(109)-C(108)-C(107)	124.73(16)
C(109)-C(108)-H(108)	117.6
C(107)-C(108)-H(108)	117.6
C(108)-C(109)-C(121)	126.67(16)
C(108)-C(109)-H(109)	116.7
C(121)-C(109)-H(109)	116.7
C(126)-C(121)-C(122)	117.92(16)
C(126)-C(121)-C(109)	123.09(16)
C(122)-C(121)-C(109)	118.98(16)
C(123)-C(122)-C(121)	120.86(17)
C(123)-C(122)-H(122)	119.6
C(121)-C(122)-H(122)	119.6
C(124)-C(123)-C(122)	120.34(19)
C(124)-C(123)-H(123)	119.8
C(122)-C(123)-H(123)	119.8
C(123)-C(124)-C(125)	119.35(18)
C(123)-C(124)-H(124)	120.3
C(125)-C(124)-H(124)	120.3
C(124)-C(125)-C(126)	120.56(18)
C(124)-C(125)-H(125)	119.7
C(126)-C(125)-H(125)	119.7

C(125)-C(126)-C(121)	120.91(18)
C(125)-C(126)-H(126)	119.5
C(121)-C(126)-H(126)	119.5
N(101)-C(105)-C(104)	111.07(13)
N(101)-C(105)-H(10E)	109.4
C(104)-C(105)-H(10E)	109.4
N(101)-C(105)-H(10F)	109.4
C(104)-C(105)-H(10F)	109.4
H(10E)-C(105)-H(10F)	108.0

Symmetry transformations used to generate equivalent atoms:

Table 4. Anisotropic displacement parameters ($Å^2$x 10^3) for V20222. The anisotropic displacement factor exponent takes the form: $-2\pi^2[h^2 a^{*2}U^{11} + ... + 2 h k a^* b^* U^{12}]$

	U^{11}	U^{22}	U^{33}	U^{23}	U^{13}	U^{12}
N(1)	19(1)	20(1)	21(1)	-10(1)	-1(1)	-4(1)
C(6)	20(1)	22(1)	21(1)	-7(1)	-5(1)	-3(1)
C(11)	17(1)	22(1)	23(1)	-9(1)	-4(1)	-4(1)
C(12)	26(1)	26(1)	19(1)	-5(1)	-4(1)	-9(1)
C(13)	31(1)	18(1)	26(1)	-4(1)	-8(1)	-7(1)
C(14)	24(1)	21(1)	25(1)	-9(1)	-8(1)	-2(1)
N(2)	35(1)	21(1)	28(1)	-10(1)	-11(1)	-1(1)
O(2)	72(1)	20(1)	40(1)	-11(1)	-5(1)	-11(1)
O(3)	62(1)	29(1)	24(1)	-12(1)	-13(1)	-3(1)
C(15)	28(1)	24(1)	20(1)	-6(1)	-7(1)	-6(1)
C(16)	26(1)	19(1)	24(1)	-5(1)	-7(1)	-5(1)
O(1)	22(1)	35(1)	34(1)	-19(1)	4(1)	-10(1)
C(1)	23(1)	20(1)	24(1)	-10(1)	-1(1)	-4(1)
C(2)	29(1)	21(1)	22(1)	-10(1)	-2(1)	-9(1)
F(1)	34(1)	30(1)	34(1)	-21(1)	-5(1)	-5(1)
C(3)	25(1)	26(1)	28(1)	-11(1)	-4(1)	-10(1)
C(4)	19(1)	26(1)	24(1)	-11(1)	2(1)	-8(1)
C(7)	23(1)	25(1)	24(1)	-10(1)	-3(1)	-3(1)
C(8)	20(1)	25(1)	25(1)	-8(1)	-1(1)	-2(1)
C(9)	24(1)	24(1)	24(1)	-5(1)	-3(1)	-4(1)
C(21)	21(1)	22(1)	31(1)	-9(1)	0(1)	-4(1)
C(22)	41(1)	24(1)	40(1)	-6(1)	3(1)	-11(1)
C(23)	46(1)	25(1)	60(1)	-19(1)	9(1)	-14(1)
C(24)	37(1)	40(1)	48(1)	-29(1)	13(1)	-16(1)
C(25)	50(1)	39(1)	32(1)	-16(1)	8(1)	-18(1)
C(26)	38(1)	23(1)	31(1)	-10(1)	3(1)	-10(1)
C(5)	21(1)	22(1)	19(1)	-8(1)	1(1)	-6(1)
N(101)	20(1)	20(1)	23(1)	-11(1)	-2(1)	-2(1)
C(106)	20(1)	22(1)	18(1)	-6(1)	-1(1)	-6(1)
C(111)	16(1)	21(1)	23(1)	-8(1)	-4(1)	-4(1)
C(112)	22(1)	24(1)	21(1)	-3(1)	-5(1)	-4(1)

C(113)	23(1)	18(1)	30(1)	-2(1)	-7(1)	-3(1)
C(114)	18(1)	19(1)	30(1)	-11(1)	-4(1)	-2(1)
N(102)	22(1)	21(1)	38(1)	-14(1)	-4(1)	-1(1)
O(102)	37(1)	18(1)	48(1)	-9(1)	-2(1)	0(1)
O(103)	43(1)	30(1)	36(1)	-20(1)	-12(1)	3(1)
C(115)	19(1)	22(1)	22(1)	-7(1)	-3(1)	-5(1)
C(116)	19(1)	16(1)	22(1)	-4(1)	-5(1)	-3(1)
O(101)	28(1)	38(1)	29(1)	-17(1)	-9(1)	-3(1)
C(101)	27(1)	25(1)	31(1)	-17(1)	0(1)	-5(1)
C(102)	25(1)	18(1)	24(1)	-9(1)	-1(1)	2(1)
F(101)	30(1)	28(1)	34(1)	-20(1)	4(1)	-1(1)
C(103)	18(1)	25(1)	28(1)	-8(1)	-1(1)	0(1)
C(104)	18(1)	23(1)	25(1)	-11(1)	-6(1)	0(1)
C(107)	21(1)	26(1)	25(1)	-10(1)	-3(1)	-5(1)
C(108)	22(1)	26(1)	26(1)	-11(1)	-5(1)	-7(1)
C(109)	23(1)	25(1)	24(1)	-9(1)	-3(1)	-8(1)
C(121)	21(1)	24(1)	26(1)	-9(1)	2(1)	-11(1)
C(122)	29(1)	24(1)	30(1)	-9(1)	-2(1)	-9(1)
C(123)	33(1)	24(1)	42(1)	-15(1)	4(1)	-10(1)
C(124)	36(1)	39(1)	37(1)	-24(1)	11(1)	-18(1)
C(125)	35(1)	50(1)	23(1)	-16(1)	4(1)	-18(1)
C(126)	26(1)	32(1)	25(1)	-10(1)	2(1)	-9(1)
C(105)	19(1)	22(1)	21(1)	-8(1)	-5(1)	-1(1)

Table 5. Hydrogen coordinates (x 10^4) and isotropic displacement parameters ($Å^2$x 10^3) for V20222.

	x	y	z	U(eq)
H(12)	2983	3831	9068	28
H(13)	3721	2013	8153	30
H(15)	3976	5175	5383	28
H(16)	3167	7002	6300	27
H(1A)	2196	8511	9390	26
H(1B)	2774	9681	8336	26
H(3)	7633	7761	9385	30
H(4)	8303	7213	7710	27
H(7A)	8909	4989	9091	29
H(7B)	6818	4893	9149	29
H(8)	9351	4812	7219	29
H(9)	7514	2749	8499	31
H(22)	8781	328	8011	43
H(23)	9617	-927	6702	52
H(24)	10097	336	4946	47
H(25)	9849	2879	4489	47
H(26)	9062	4148	5790	37
H(5A)	5579	8371	7041	25
H(5B)	5790	6657	7167	25
H(112)	2856	6224	759	28
H(113)	1264	8098	1631	30
H(115)	1806	4984	4436	25
H(116)	3445	3128	3554	23
H(10A)	6528	1507	541	32
H(10B)	7167	328	1613	32
H(103)	11311	2087	736	30
H(104)	9959	2673	2390	27
H(10C)	9858	4866	936	28
H(10D)	7700	5008	912	28
H(108)	8792	5214	2740	28

H(109)	5810	6996	1630	27
H(122)	3946	9282	2095	33
H(123)	2904	10799	3251	39
H(124)	4241	10083	4791	42
H(125)	6560	7800	5196	41
H(126)	7530	6236	4081	33
H(10E)	7606	1574	2973	25
H(10F)	6476	3304	2832	25

Table 6. Torsion angles [°] for V20222.

C(1)-N(1)-C(6)-O(1)	-2.3(2)
C(5)-N(1)-C(6)-O(1)	174.18(16)
C(1)-N(1)-C(6)-C(11)	177.95(14)
C(5)-N(1)-C(6)-C(11)	-5.6(2)
O(1)-C(6)-C(11)-C(12)	-54.8(2)
N(1)-C(6)-C(11)-C(12)	124.94(17)
O(1)-C(6)-C(11)-C(16)	122.02(18)
N(1)-C(6)-C(11)-C(16)	-58.2(2)
C(16)-C(11)-C(12)-C(13)	2.2(3)
C(6)-C(11)-C(12)-C(13)	179.10(15)
C(11)-C(12)-C(13)-C(14)	-0.2(3)
C(12)-C(13)-C(14)-C(15)	-1.1(3)
C(12)-C(13)-C(14)-N(2)	178.69(16)
C(13)-C(14)-N(2)-O(2)	8.7(2)
C(15)-C(14)-N(2)-O(2)	-171.54(18)
C(13)-C(14)-N(2)-O(3)	-172.07(17)
C(15)-C(14)-N(2)-O(3)	7.7(2)
C(13)-C(14)-C(15)-C(16)	0.3(3)
N(2)-C(14)-C(15)-C(16)	-179.48(15)
C(14)-C(15)-C(16)-C(11)	1.7(2)
C(12)-C(11)-C(16)-C(15)	-3.0(2)
C(6)-C(11)-C(16)-C(15)	-179.77(15)
C(6)-N(1)-C(1)-C(2)	134.31(15)
C(5)-N(1)-C(1)-C(2)	-42.60(17)
N(1)-C(1)-C(2)-C(3)	9.7(2)
N(1)-C(1)-C(2)-F(1)	-170.11(13)
F(1)-C(2)-C(3)-C(4)	-175.73(14)
C(1)-C(2)-C(3)-C(4)	4.5(3)
C(2)-C(3)-C(4)-C(5)	12.6(2)
C(2)-C(3)-C(4)-C(7)	-114.69(19)
C(3)-C(4)-C(7)-C(8)	-173.93(14)
C(5)-C(4)-C(7)-C(8)	60.10(18)
C(4)-C(7)-C(8)-C(9)	-138.02(18)
C(7)-C(8)-C(9)-C(21)	177.01(16)

C(8)-C(9)-C(21)-C(26)	-26.6(3)
C(8)-C(9)-C(21)-C(22)	155.9(2)
C(26)-C(21)-C(22)-C(23)	-0.2(3)
C(9)-C(21)-C(22)-C(23)	177.45(19)
C(21)-C(22)-C(23)-C(24)	-0.8(3)
C(22)-C(23)-C(24)-C(25)	1.2(4)
C(23)-C(24)-C(25)-C(26)	-0.6(4)
C(24)-C(25)-C(26)-C(21)	-0.3(3)
C(22)-C(21)-C(26)-C(25)	0.7(3)
C(9)-C(21)-C(26)-C(25)	-176.85(19)
C(6)-N(1)-C(5)-C(4)	-114.51(17)
C(1)-N(1)-C(5)-C(4)	62.08(17)
C(3)-C(4)-C(5)-N(1)	-43.69(18)
C(7)-C(4)-C(5)-N(1)	83.57(16)
C(101)-N(101)-C(106)-O(101)	-1.3(2)
C(105)-N(101)-C(106)-O(101)	173.15(16)
C(101)-N(101)-C(106)-C(111)	178.58(14)
C(105)-N(101)-C(106)-C(111)	-6.9(2)
O(101)-C(106)-C(111)-C(116)	126.28(18)
N(101)-C(106)-C(111)-C(116)	-53.6(2)
O(101)-C(106)-C(111)-C(112)	-50.4(2)
N(101)-C(106)-C(111)-C(112)	129.66(16)
C(116)-C(111)-C(112)-C(113)	4.6(2)
C(106)-C(111)-C(112)-C(113)	-178.61(15)
C(111)-C(112)-C(113)-C(114)	-1.1(2)
C(112)-C(113)-C(114)-C(115)	-3.1(3)
C(112)-C(113)-C(114)-N(102)	175.88(15)
C(115)-C(114)-N(102)-O(102)	-175.07(16)
C(113)-C(114)-N(102)-O(102)	5.9(2)
C(115)-C(114)-N(102)-O(103)	5.5(2)
C(113)-C(114)-N(102)-O(103)	-173.51(16)
C(113)-C(114)-C(115)-C(116)	3.7(2)
N(102)-C(114)-C(115)-C(116)	-175.26(14)
C(114)-C(115)-C(116)-C(111)	-0.1(2)
C(112)-C(111)-C(116)-C(115)	-4.0(2)
C(106)-C(111)-C(116)-C(115)	179.39(15)

C(106)-N(101)-C(101)-C(102)	131.28(16)
C(105)-N(101)-C(101)-C(102)	-43.96(19)
N(101)-C(101)-C(102)-C(103)	12.3(3)
N(101)-C(101)-C(102)-F(101)	-168.29(13)
F(101)-C(102)-C(103)-C(104)	-177.36(14)
C(101)-C(102)-C(103)-C(104)	2.0(3)
C(102)-C(103)-C(104)-C(105)	13.8(2)
C(102)-C(103)-C(104)-C(107)	-111.81(19)
C(103)-C(104)-C(107)-C(108)	-169.49(14)
C(105)-C(104)-C(107)-C(108)	65.58(18)
C(104)-C(107)-C(108)-C(109)	-129.96(18)
C(107)-C(108)-C(109)-C(121)	-178.94(16)
C(108)-C(109)-C(121)-C(126)	-11.1(3)
C(108)-C(109)-C(121)-C(122)	167.76(18)
C(126)-C(121)-C(122)-C(123)	1.2(2)
C(109)-C(121)-C(122)-C(123)	-177.79(16)
C(121)-C(122)-C(123)-C(124)	0.7(3)
C(122)-C(123)-C(124)-C(125)	-1.3(3)
C(123)-C(124)-C(125)-C(126)	0.0(3)
C(124)-C(125)-C(126)-C(121)	1.9(3)
C(122)-C(121)-C(126)-C(125)	-2.4(3)
C(109)-C(121)-C(126)-C(125)	176.48(16)
C(106)-N(101)-C(105)-C(104)	-112.83(18)
C(101)-N(101)-C(105)-C(104)	61.85(18)
C(103)-C(104)-C(105)-N(101)	-43.68(18)
C(107)-C(104)-C(105)-N(101)	81.51(17)

Symmetry transformations used to generate equivalent atoms:

Abbreviations

DMAP	4-Dimethylaminopyridine
EDC*HCl	1-Ethyl-3-(3-dimethylaminopropyl)carbodiimide hydrochloride salt
EtOAc	Ethyl acetate
Tol	Toluene
KOtBu	Potassium *tert*-butoxide
SPhos	Dicyclohexyl(2′,6′-dimethoxy[1,1′-biphenyl]-2-yl)phosphane
DIBAL	Diisobutylaluminium hydride
coe	cyclooctene

NMR and IR Spectra of New Compounds

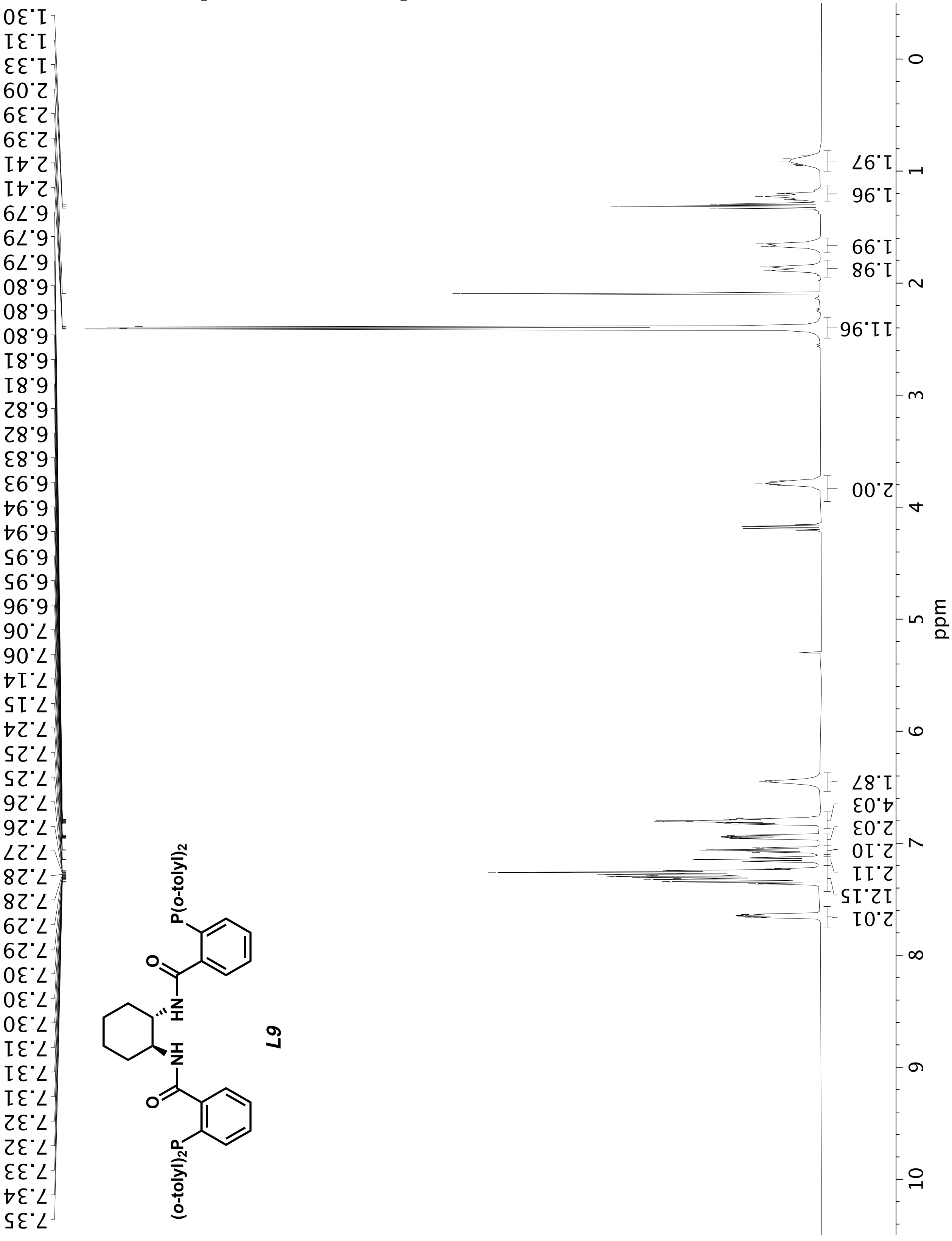

^{1}H NMR (400 MHz, $CDCl_3$) of compound **L9**.

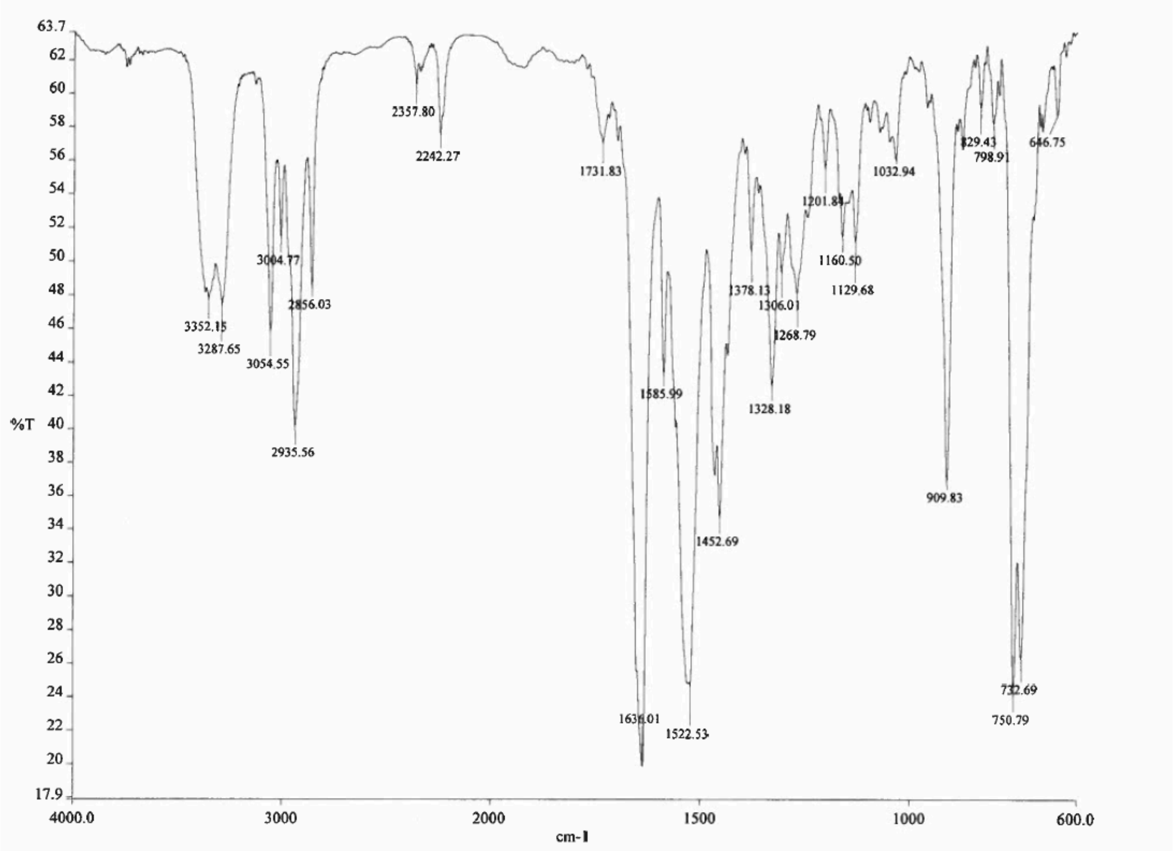

Infrared spectrum (Thin Film, NaCl) of compound **L9**.

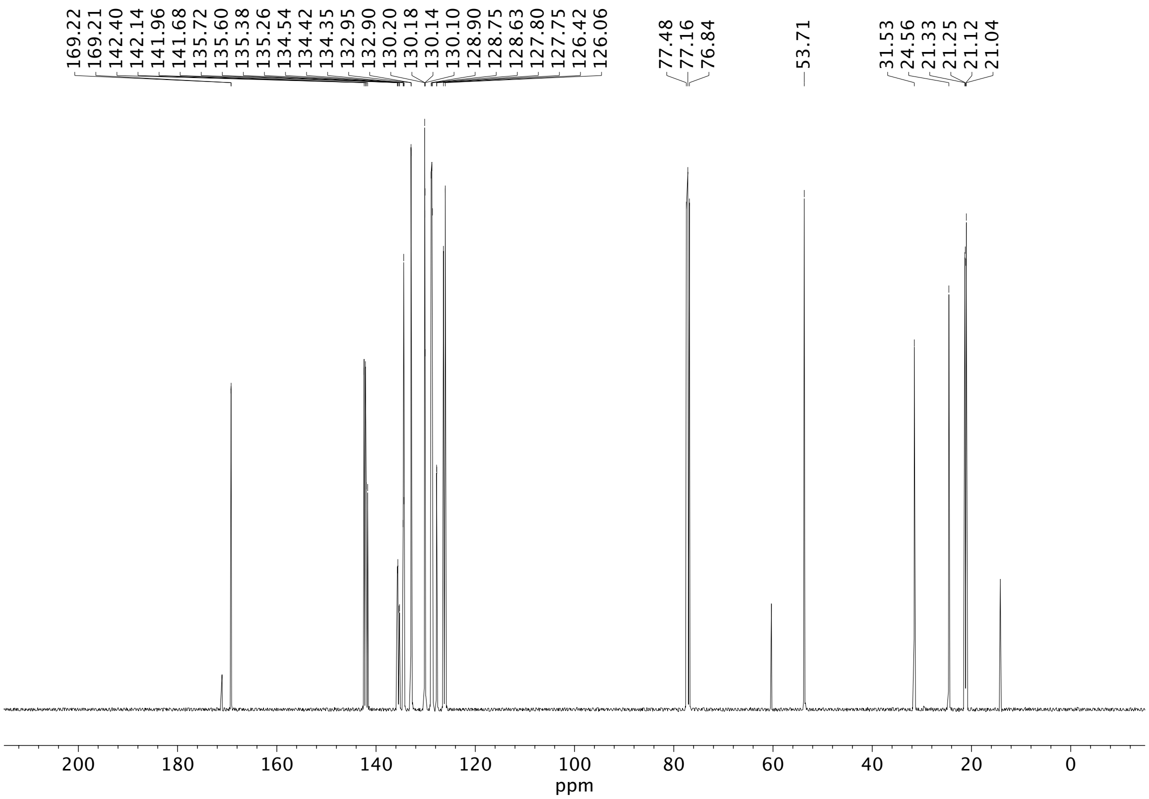

^{13}C NMR (100 MHz, $CDCl_3$) of compound **L9**.

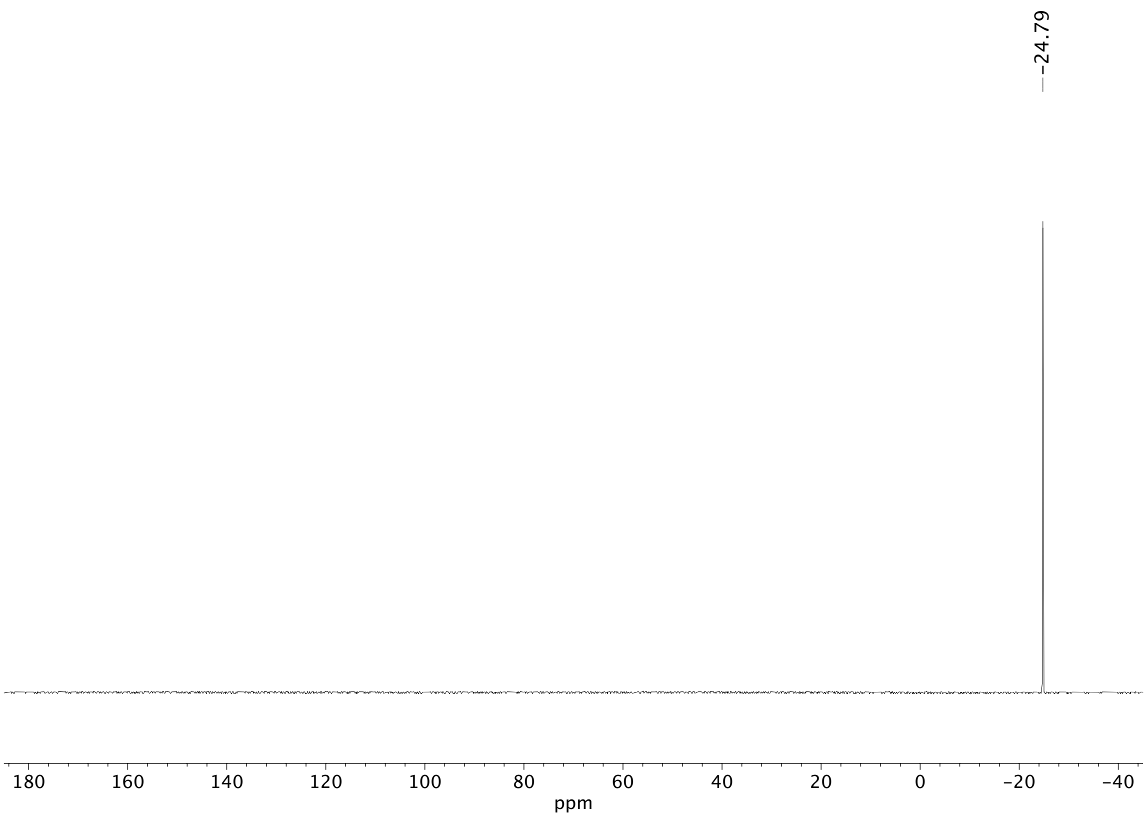

^{31}P NMR (121 MHz, $CDCl_3$) of compound **L9**.

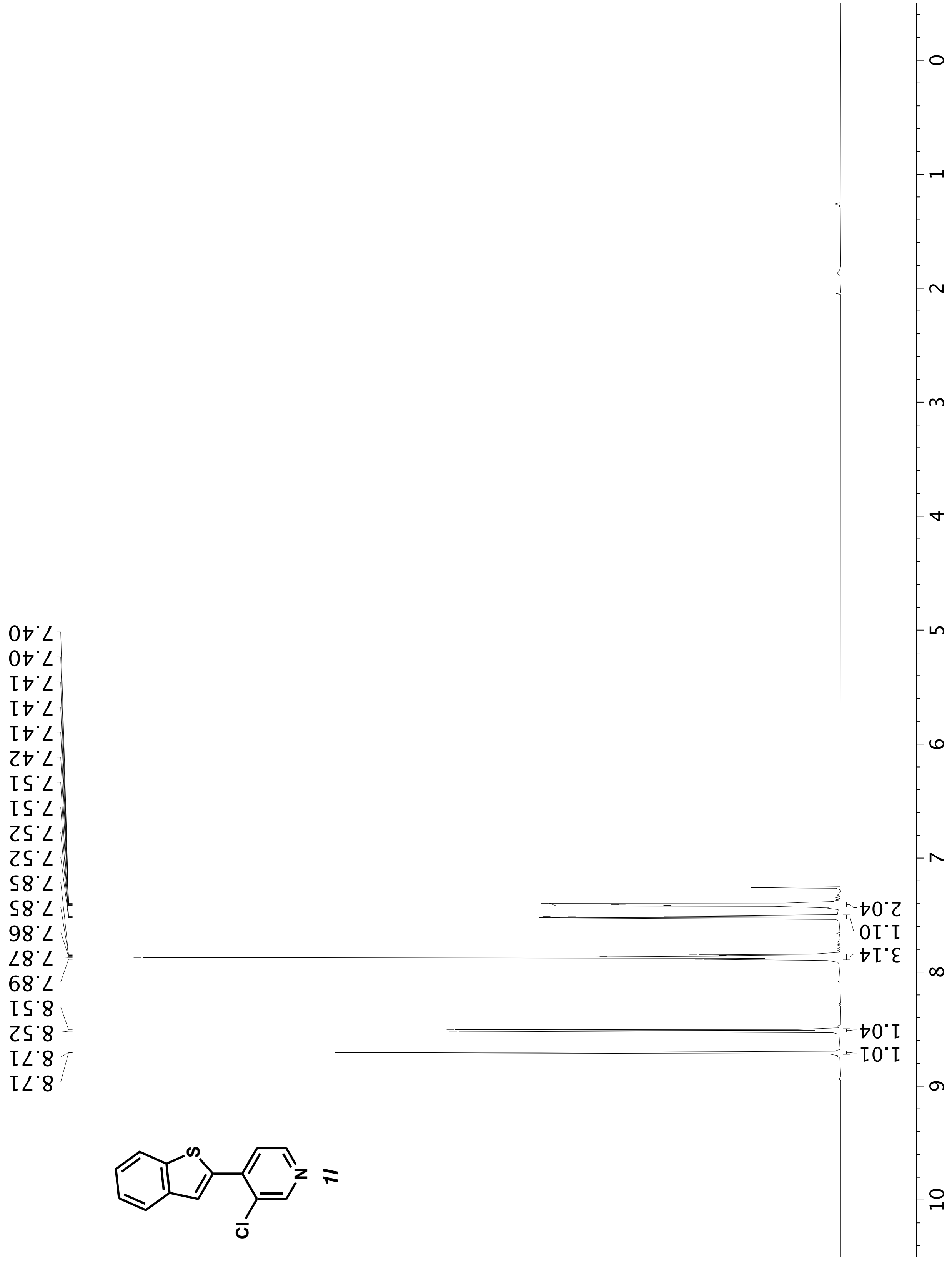

^{1}H NMR (400 MHz, CDCl$_3$) of compound **1l**.

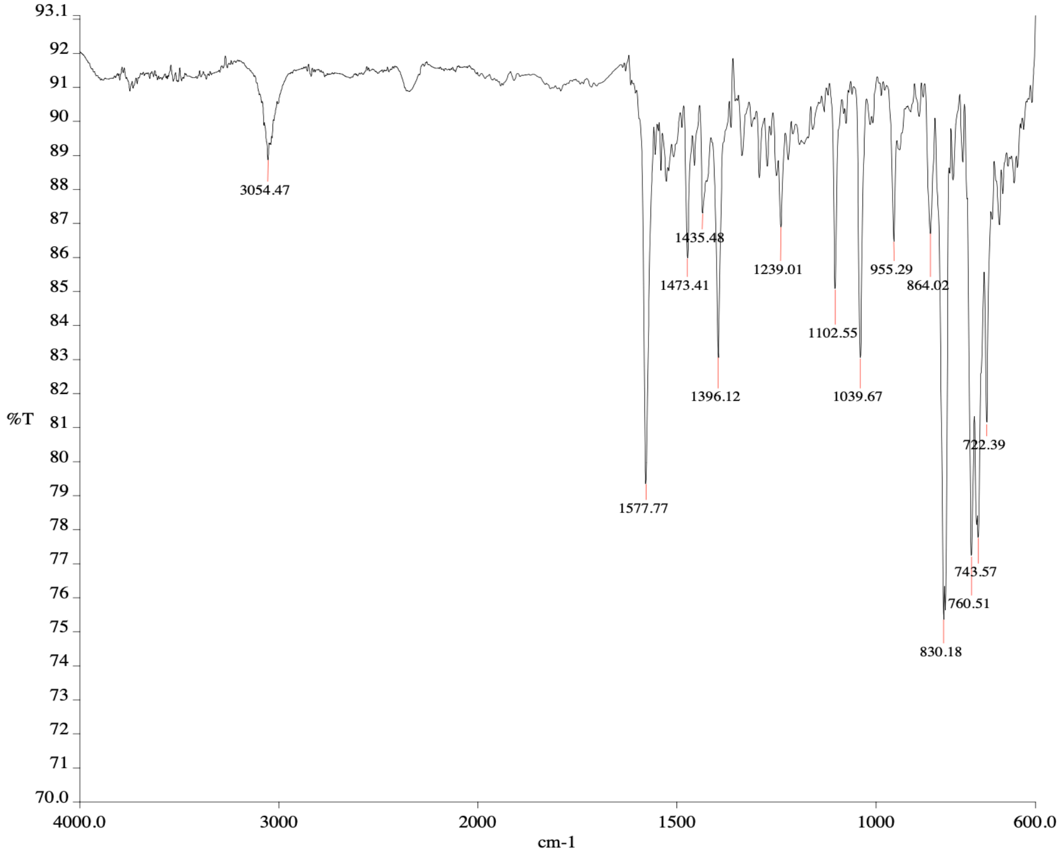

Infrared spectrum (Thin Film, NaCl) of compound **1l**.

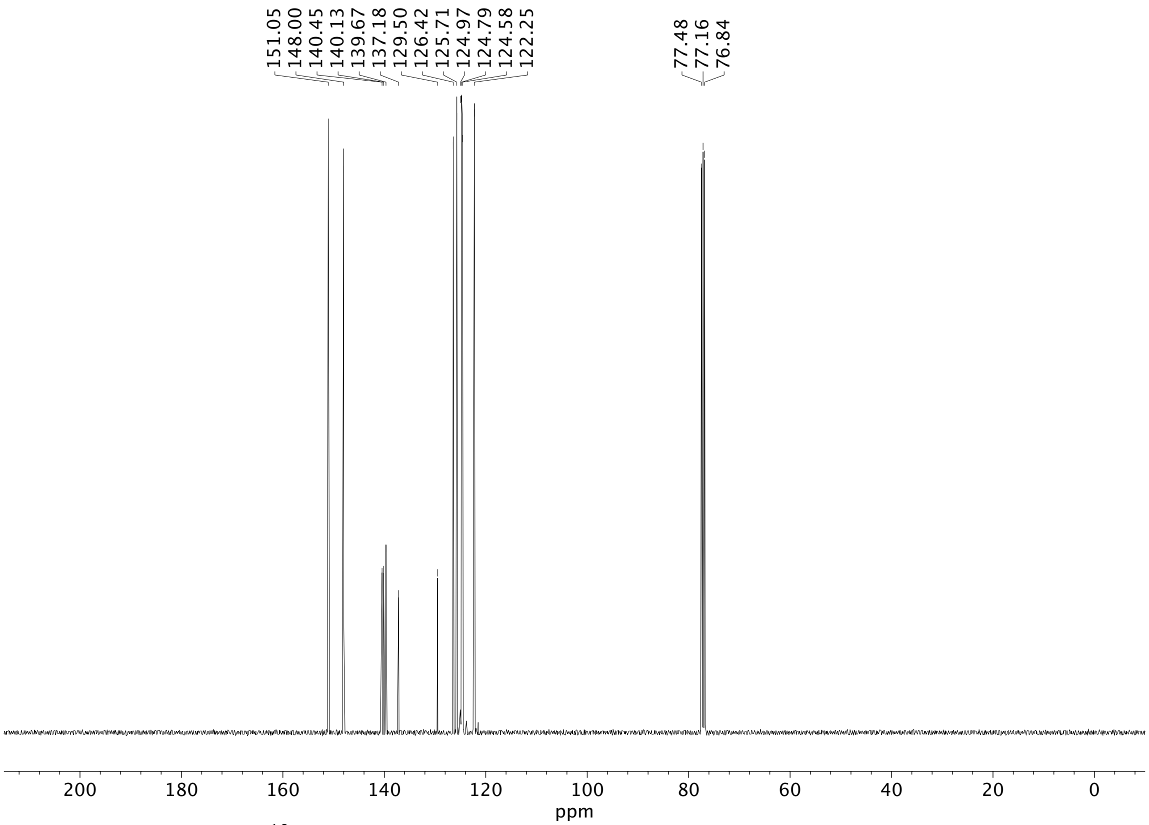

^{13}C NMR (100 MHz, $CDCl_3$) of compound **1l**.

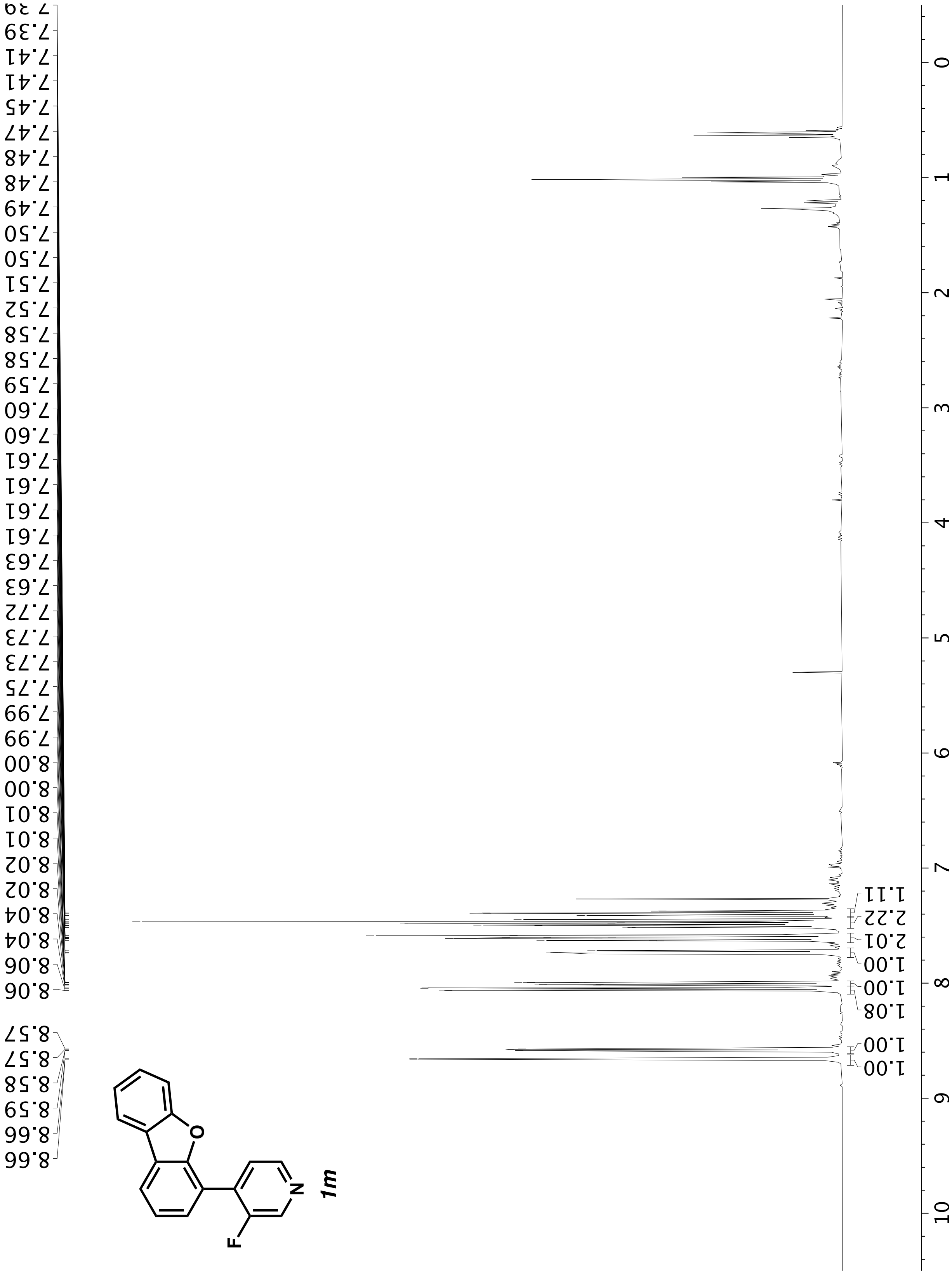

^{1}H NMR (400 MHz, $CDCl_3$) of compound **1m**.

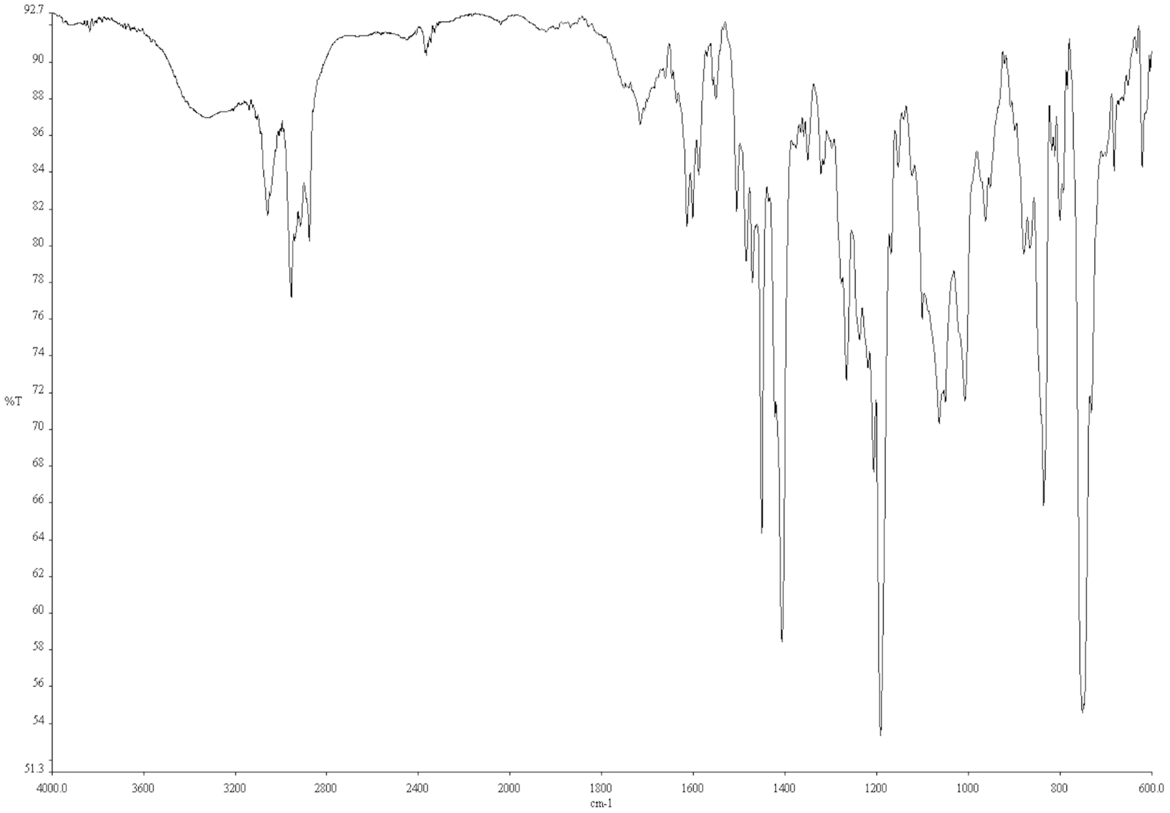

Infrared spectrum (Thin Film, NaCl) of compound **1m**.

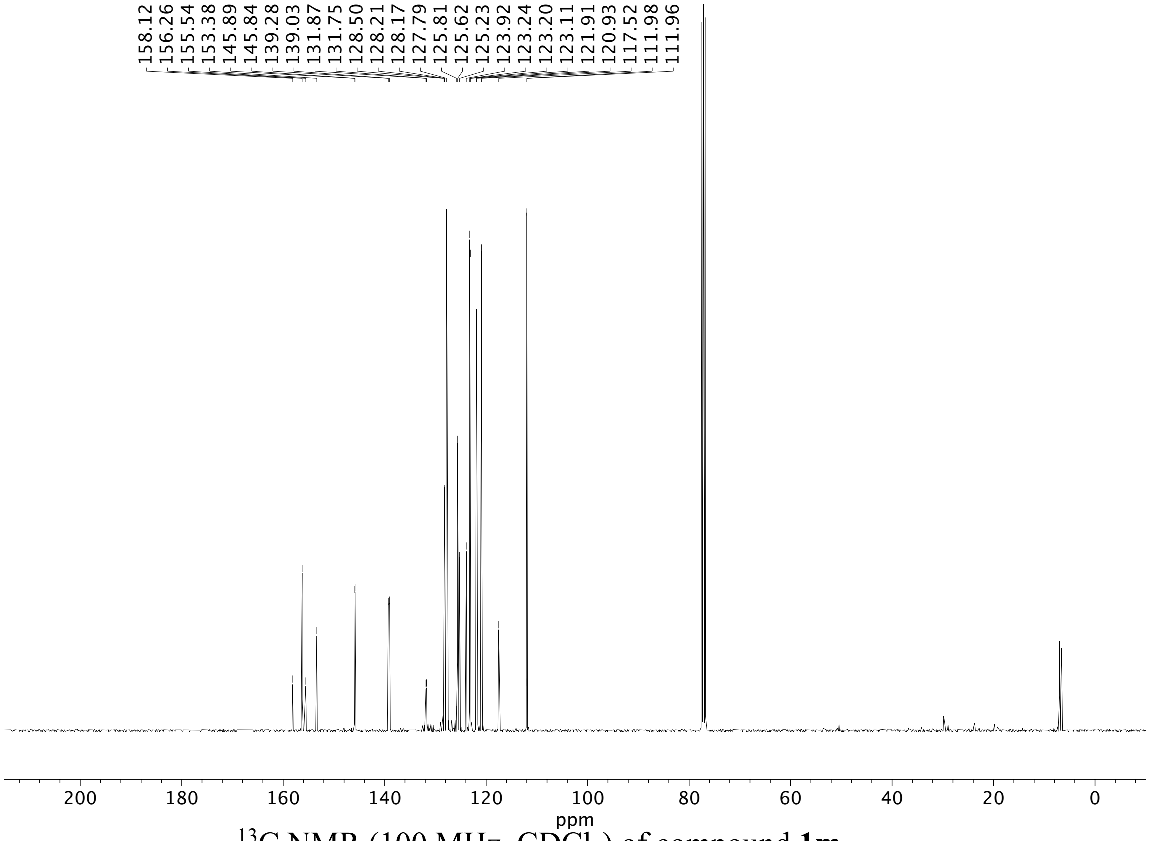

^{13}C NMR (100 MHz, $CDCl_3$) of compound **1m**.

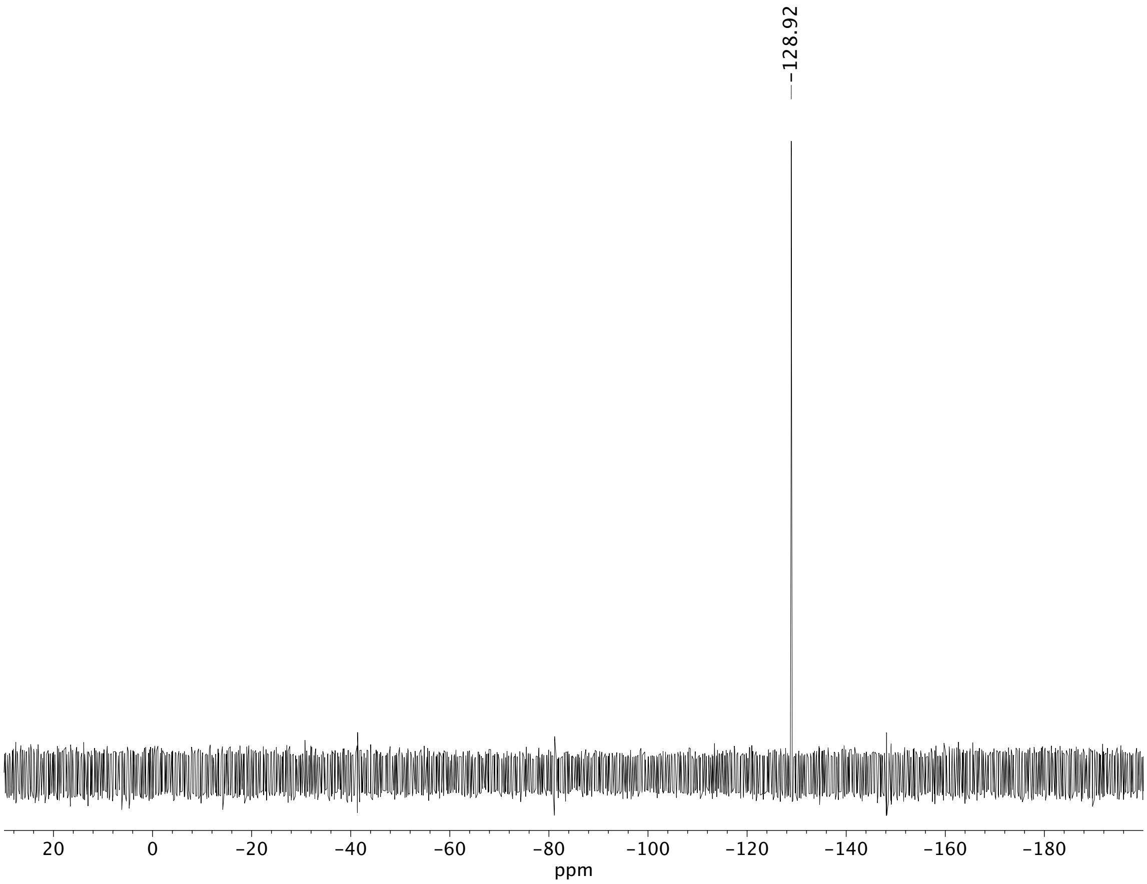

^{19}F NMR (282 MHz, $CDCl_3$) of compound **1m**.

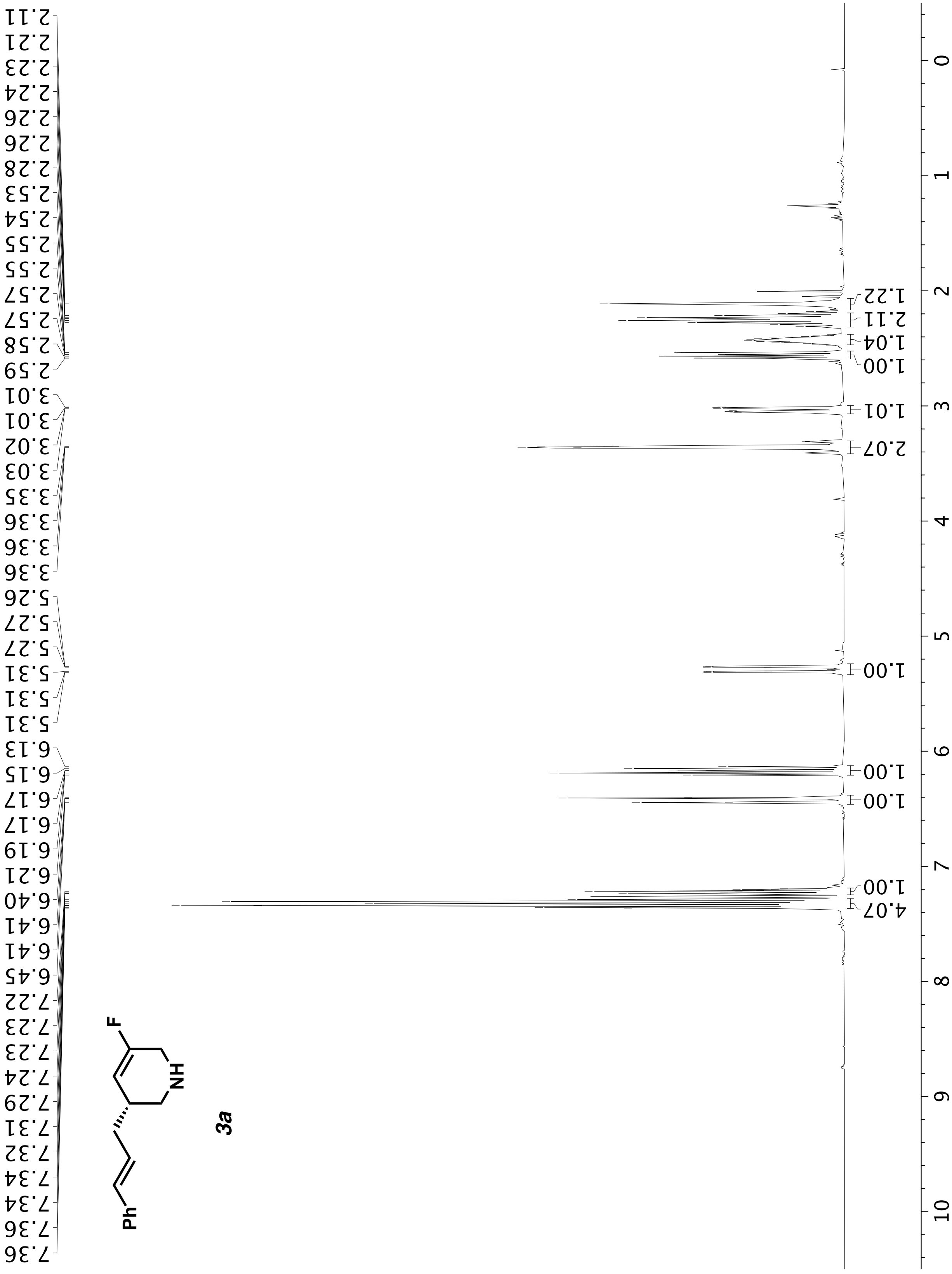

^{1}H NMR (400 MHz, $CDCl_3$) of compound **3a**.

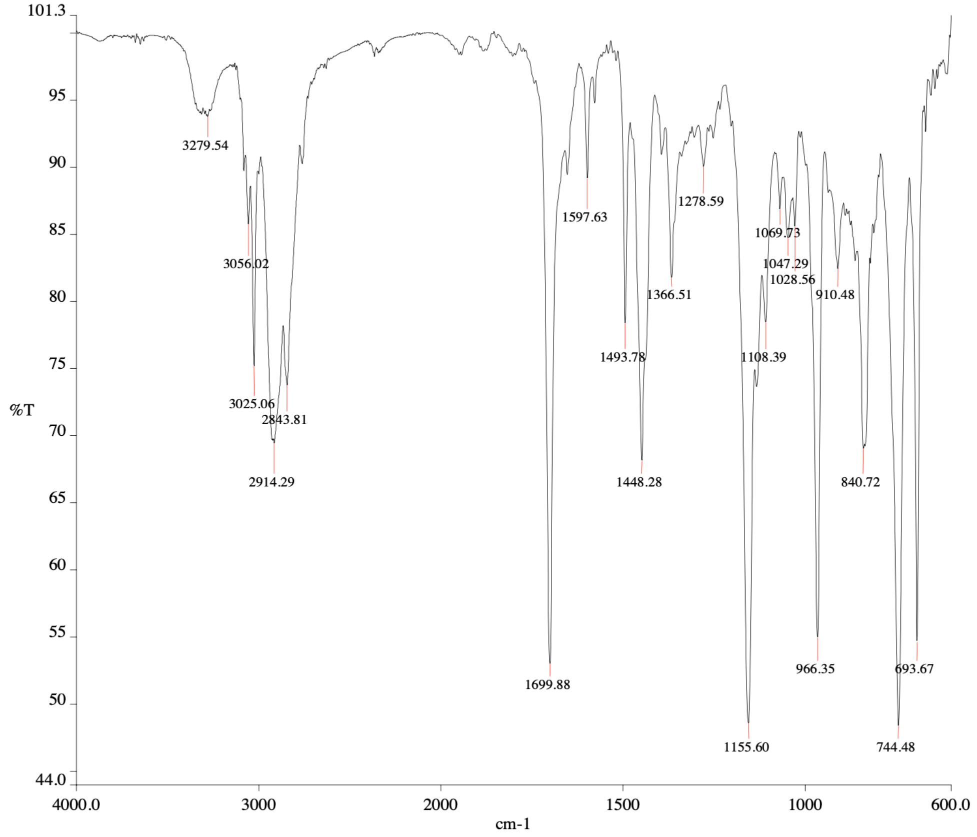

Infrared spectrum (Thin Film, NaCl) of compound **3a**.

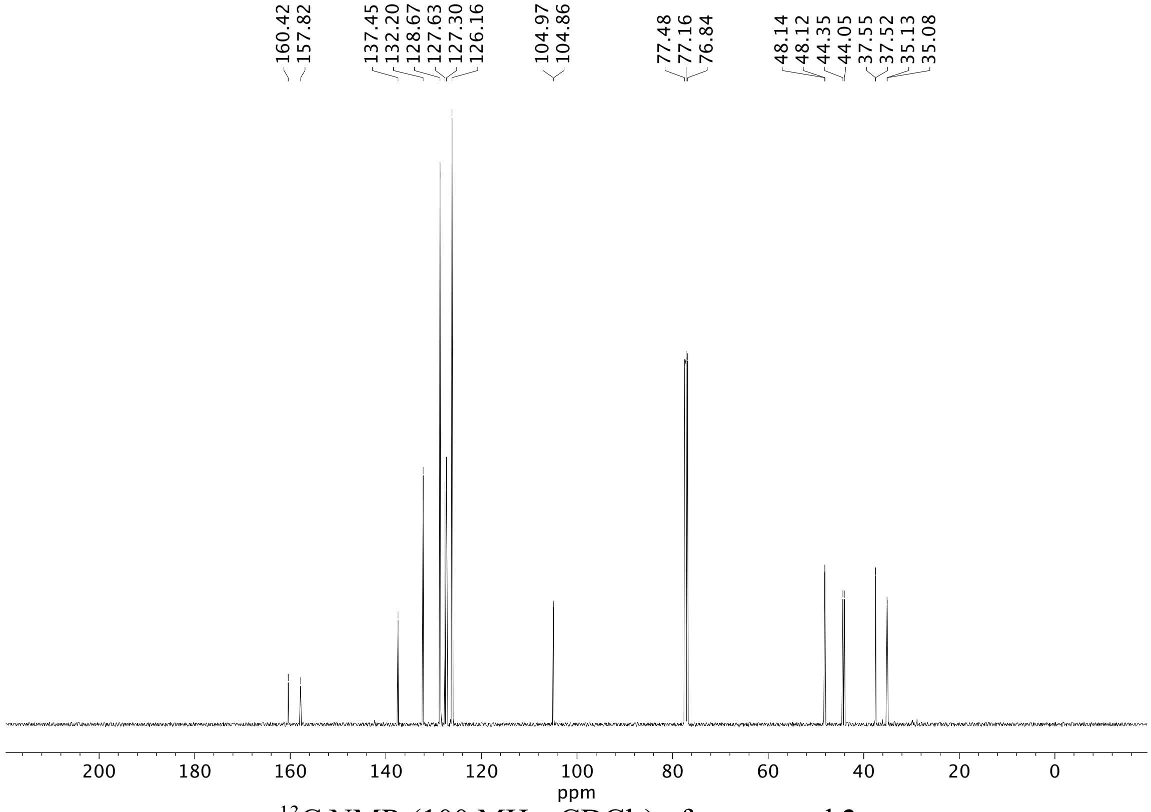

^{13}C NMR (100 MHz, $CDCl_3$) of compound **3a**.

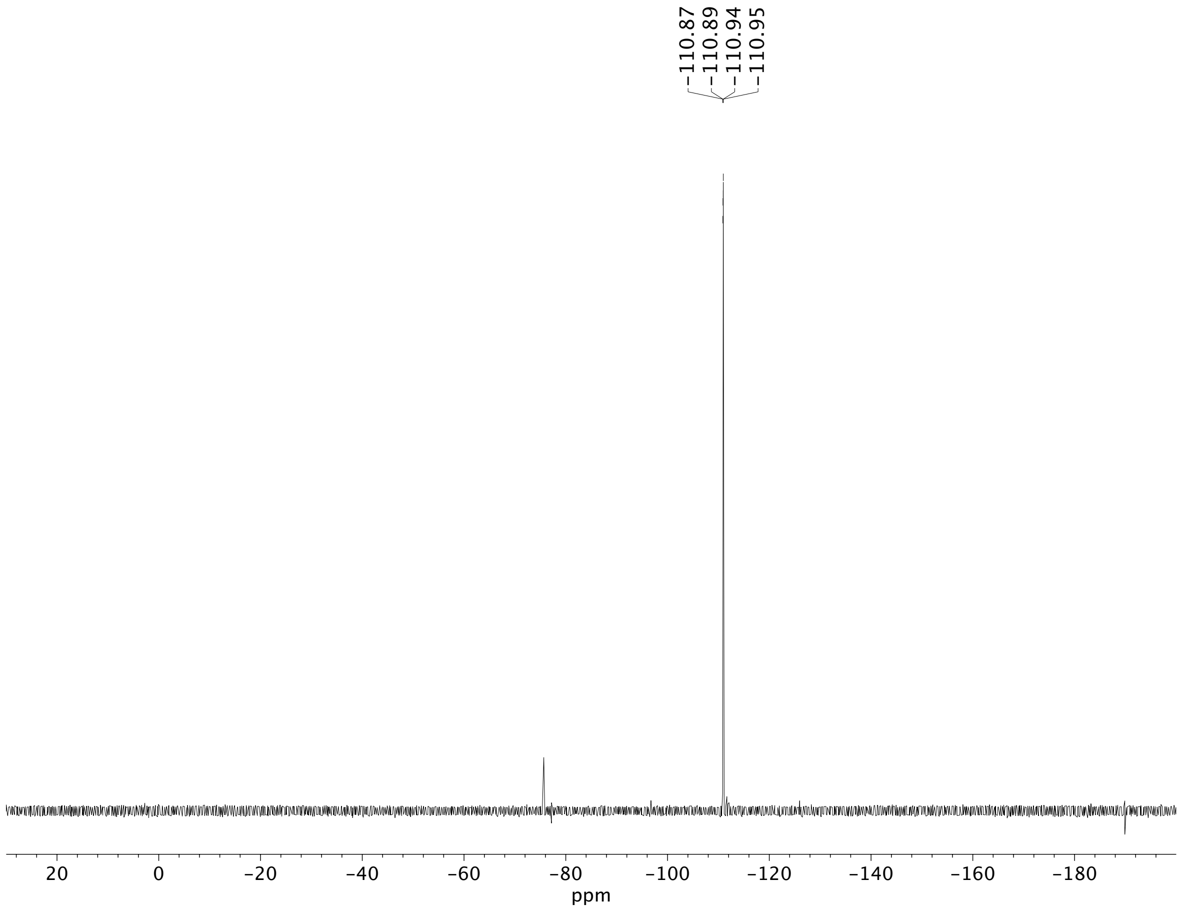

^{19}F NMR (282 MHz, $CDCl_3$) of compound **3a.**

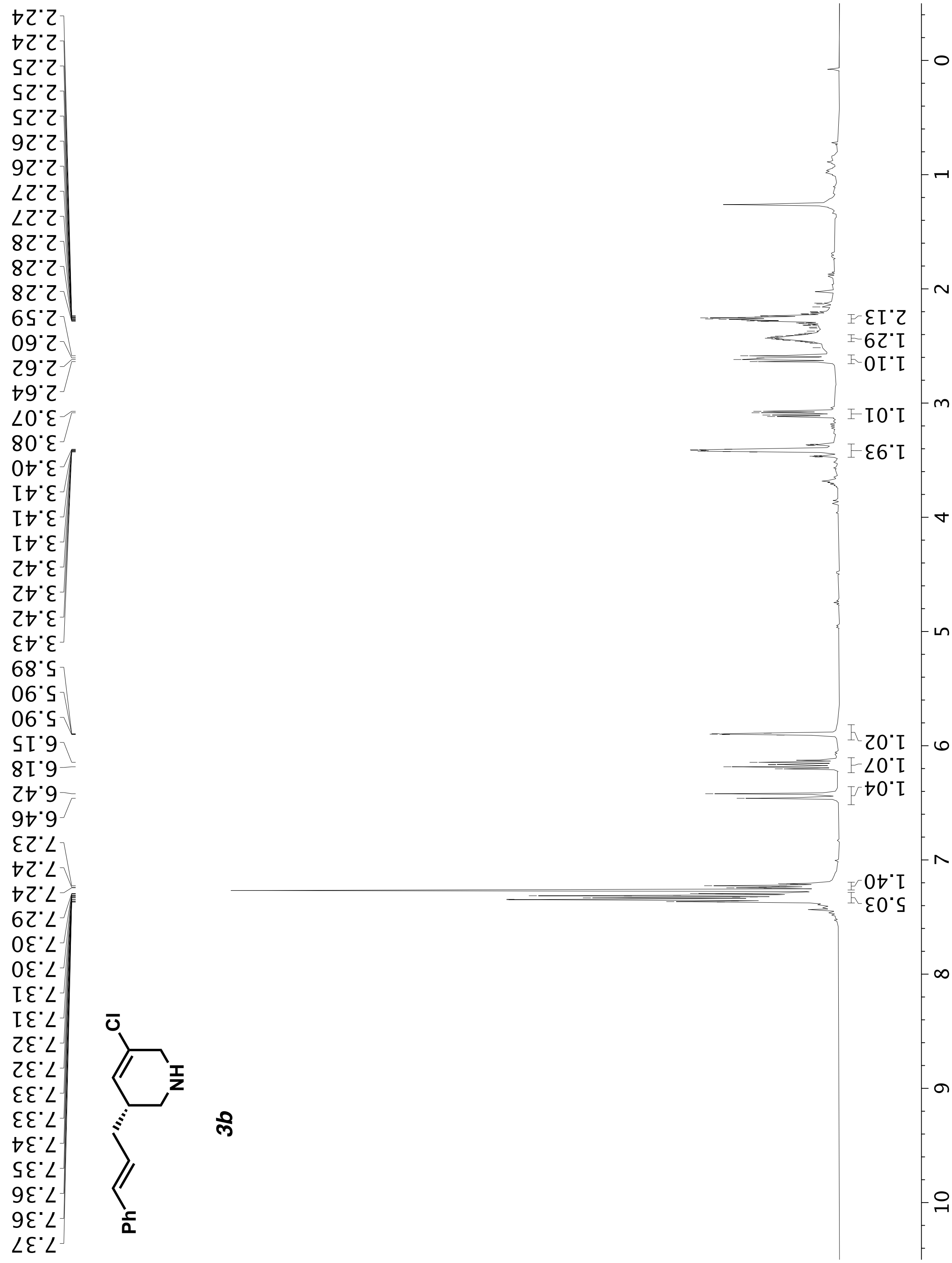

^{1}H NMR (400 MHz, $CDCl_3$) of compound **3b**.

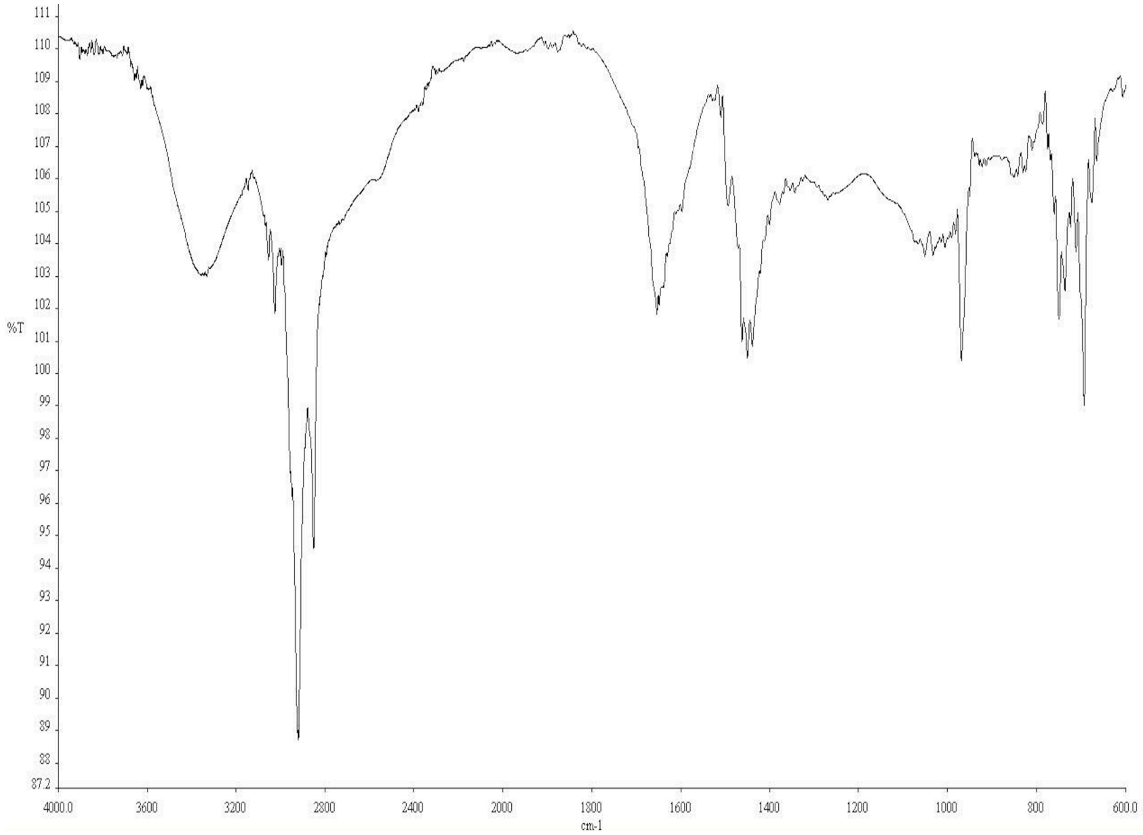

Infrared spectrum (Thin Film, NaCl) of compound **3b**.

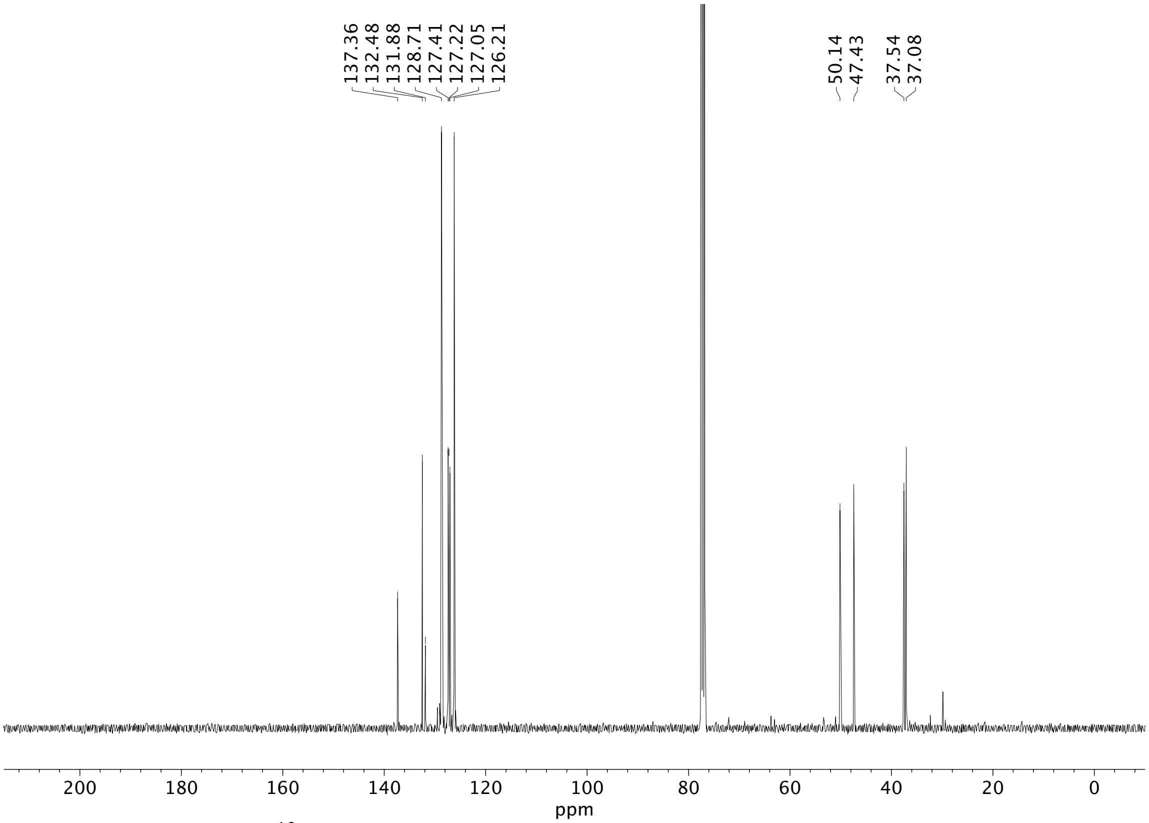

^{13}C NMR (100 MHz, $CDCl_3$) of compound **3b**.

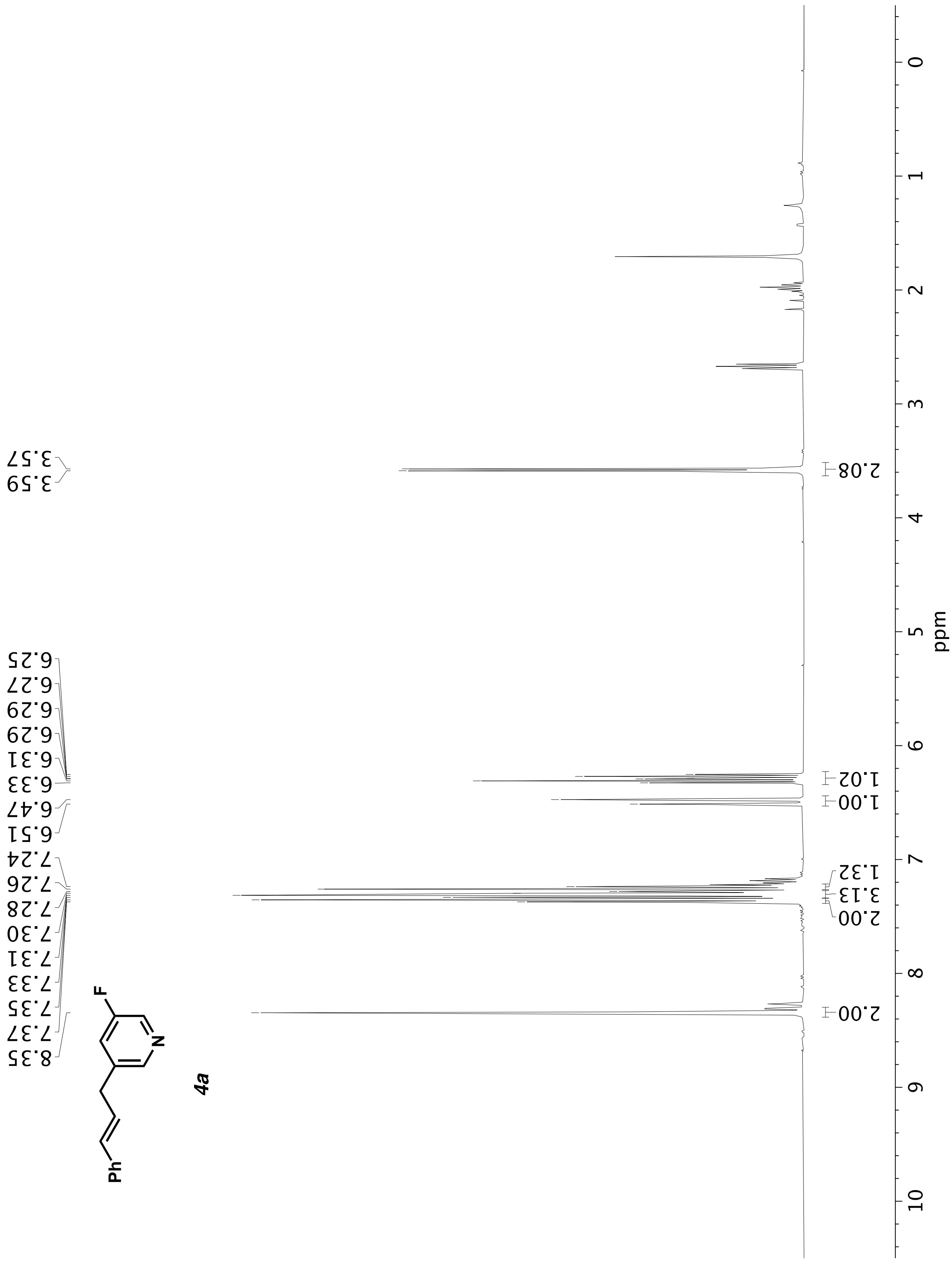

^{1}H NMR (400 MHz, $CDCl_3$) of compound **4a**

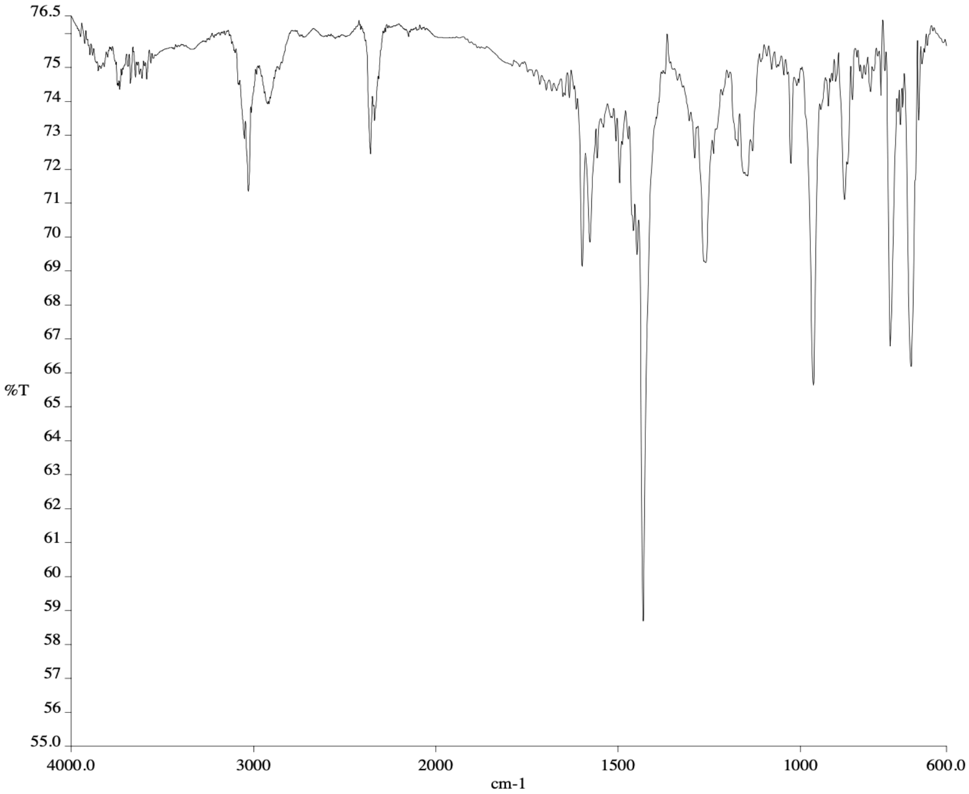

Infrared spectrum (Thin Film, NaCl) of compound **4a**.

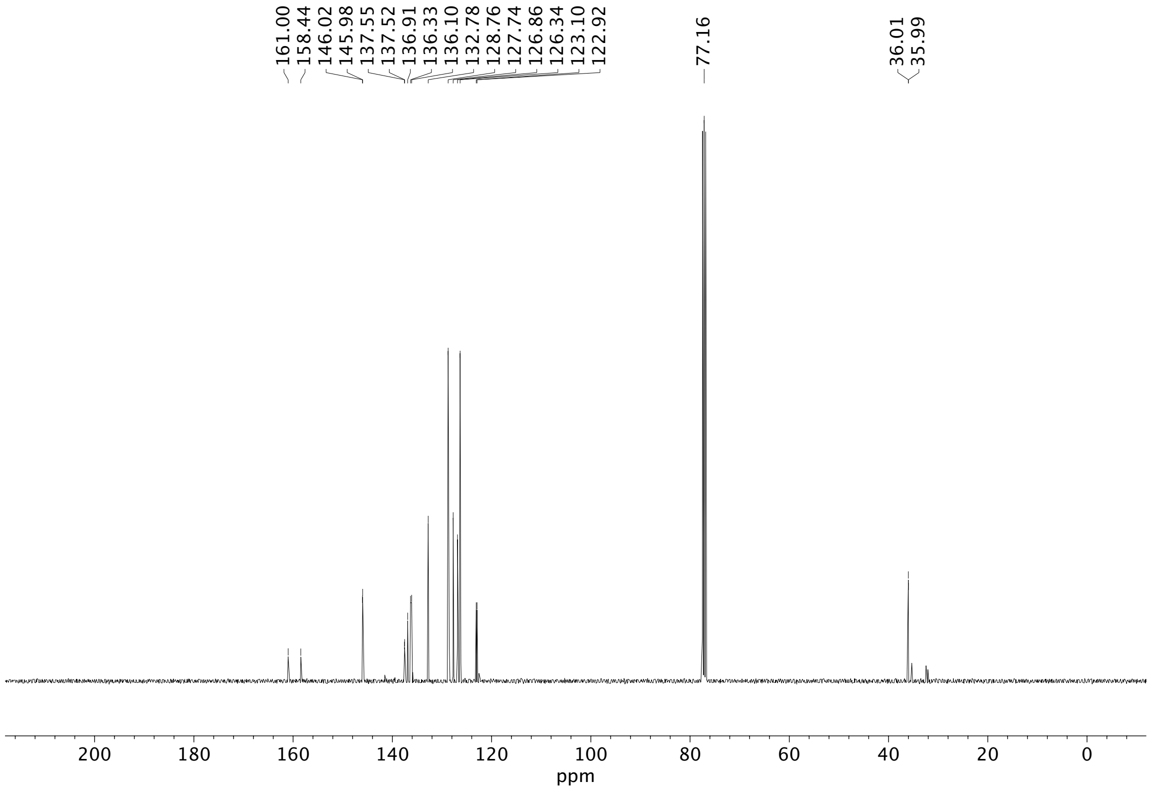

^{13}C NMR (100 MHz, $CDCl_3$) of compound **4a**.

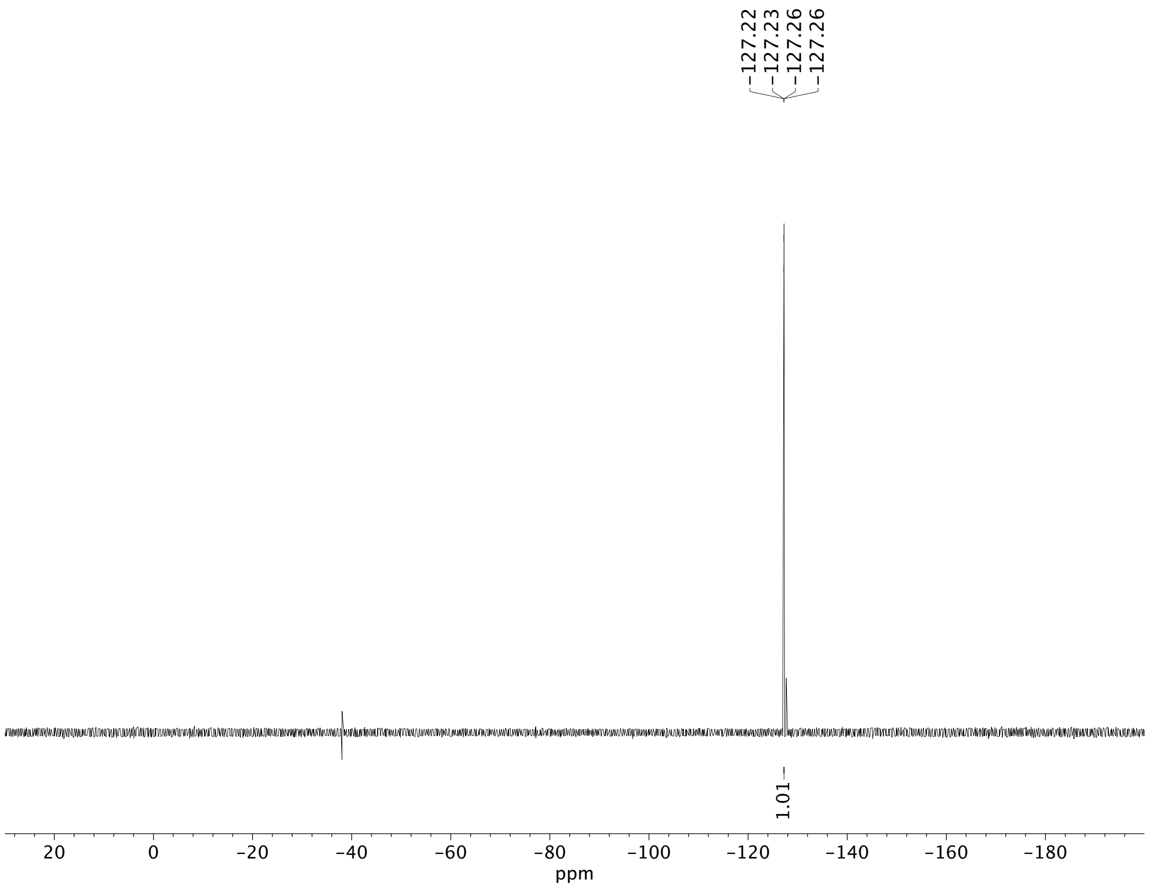

^{19}F NMR (282 MHz, $CDCl_3$) of compound **4a**.

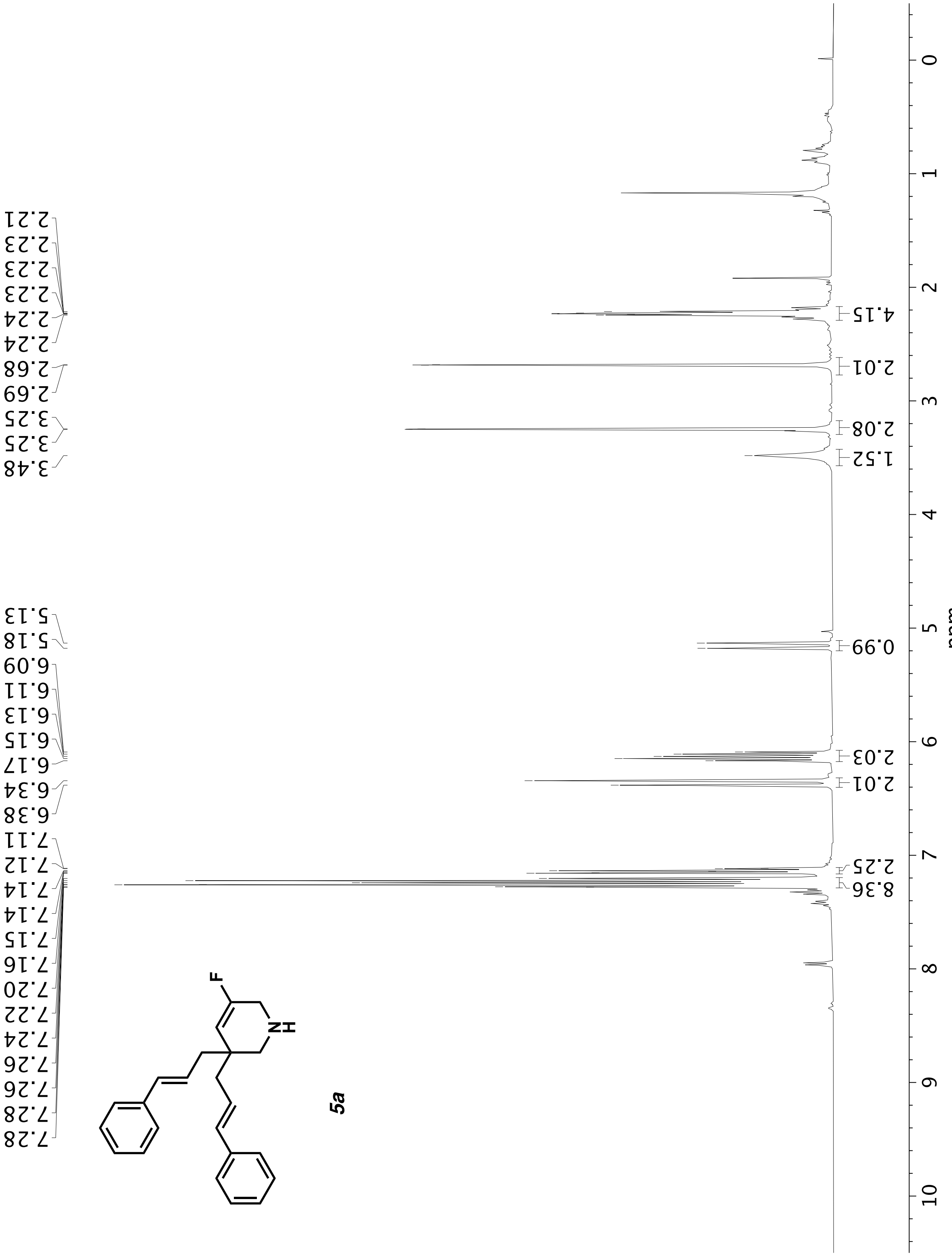

^{1}H NMR (400 MHz, $CDCl_3$) of compound **5a**.

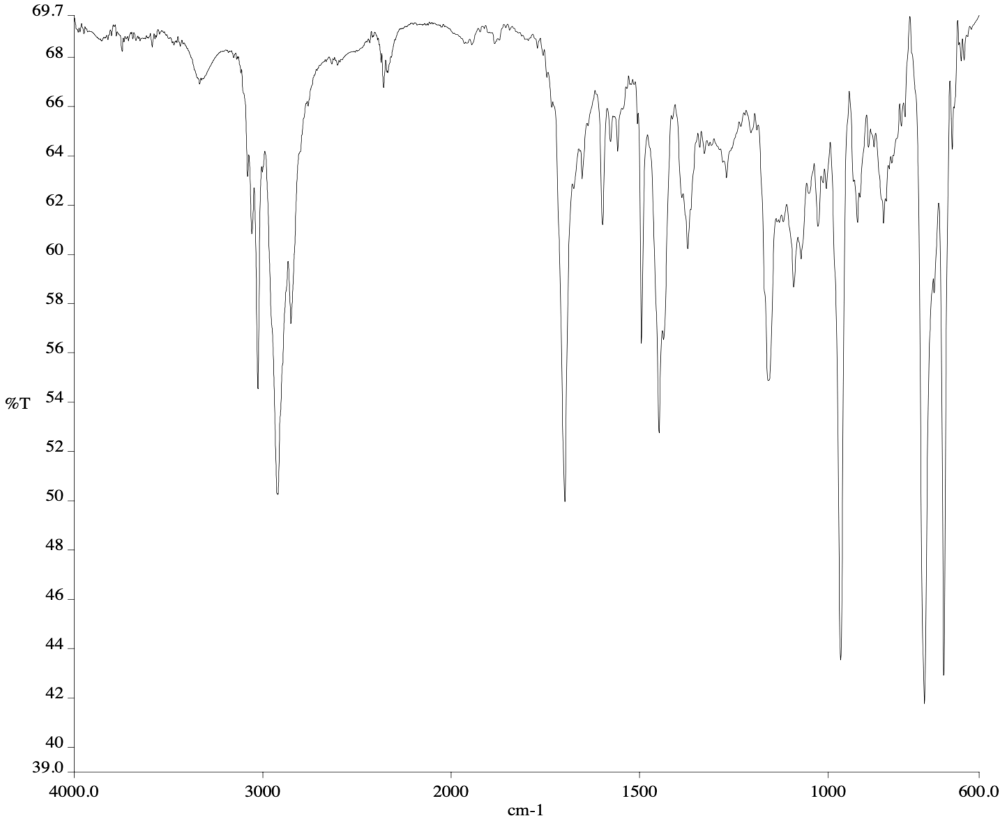

Infrared spectrum (Thin Film, NaCl) of compound **5a**.

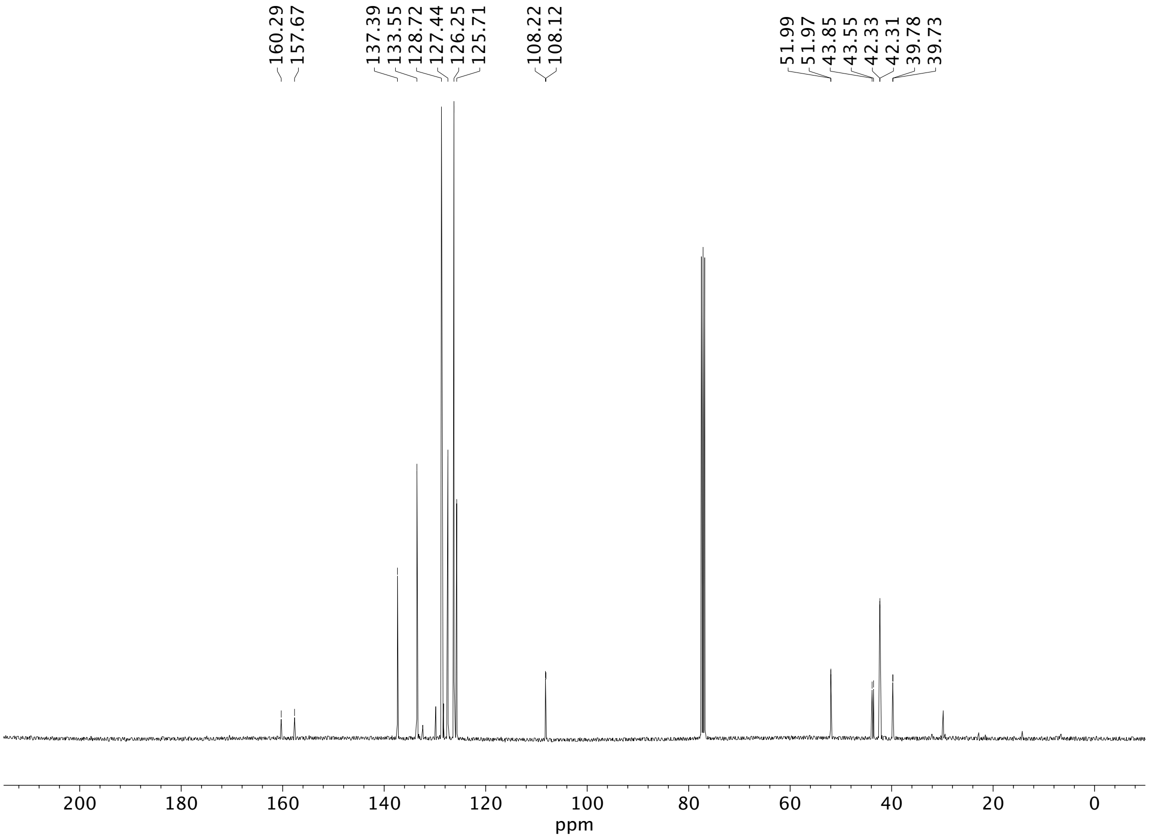

^{13}C NMR (100 MHz, $CDCl_3$) of compound **5a**.

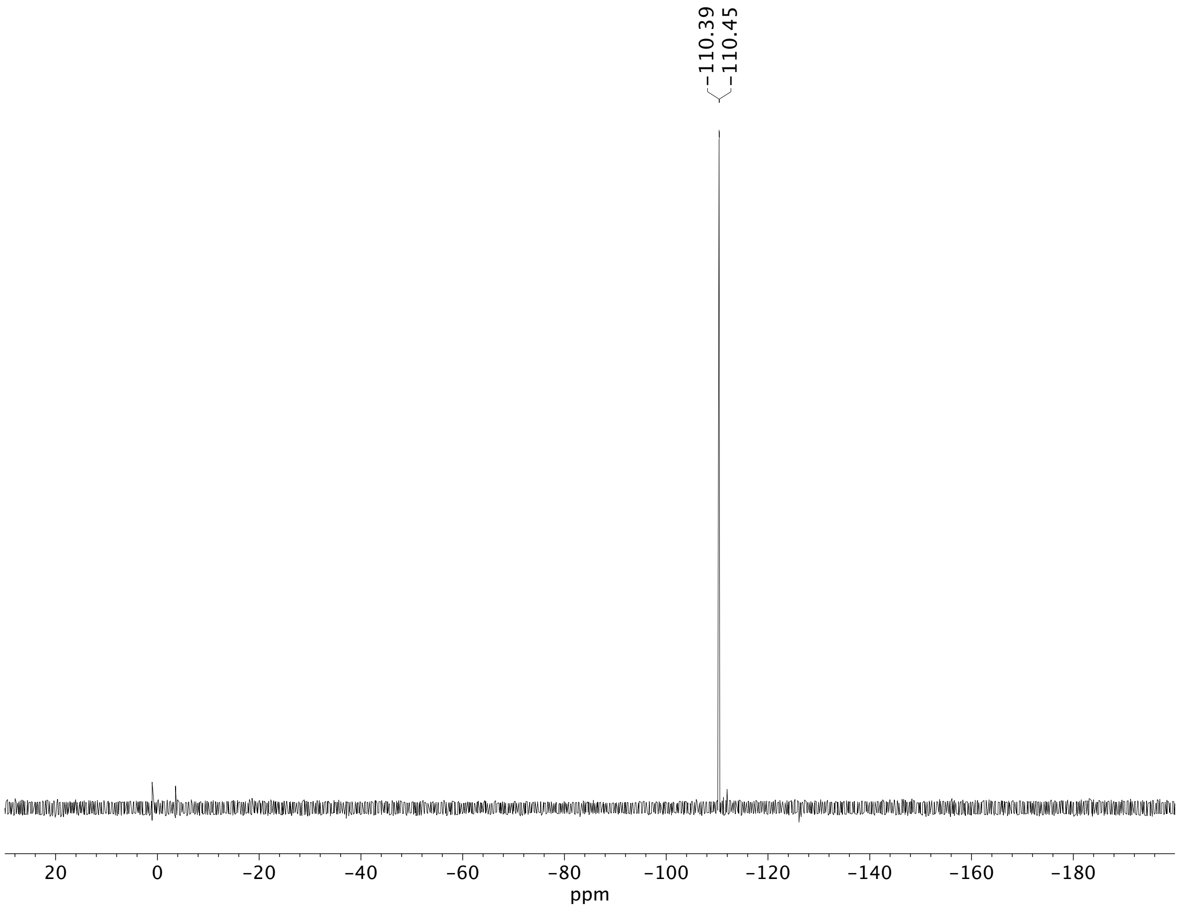

^{19}F NMR (282 MHz, $CDCl_3$) of compound **5a.**

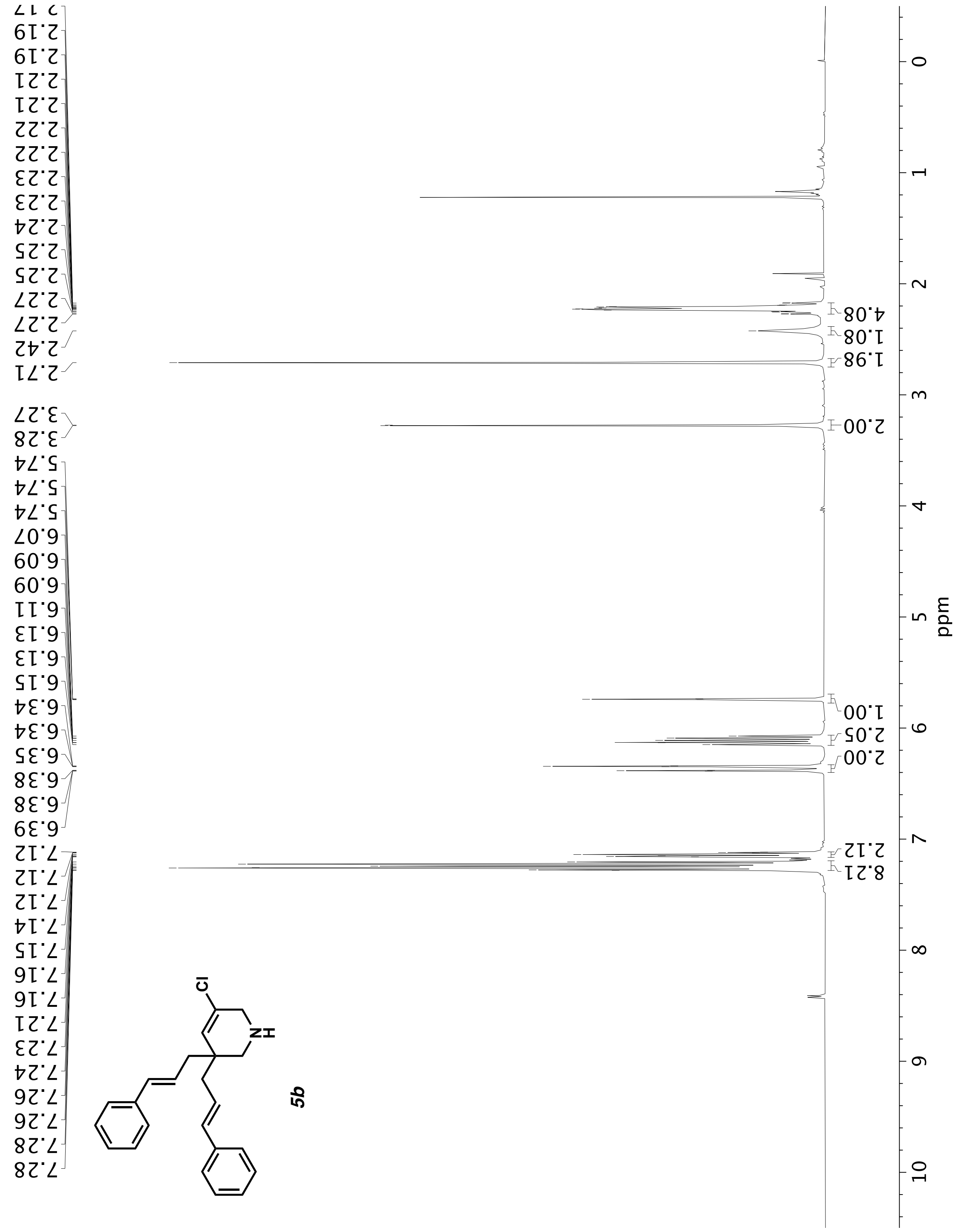

^{1}H NMR (400 MHz, $CDCl_3$) of compound **5b**.

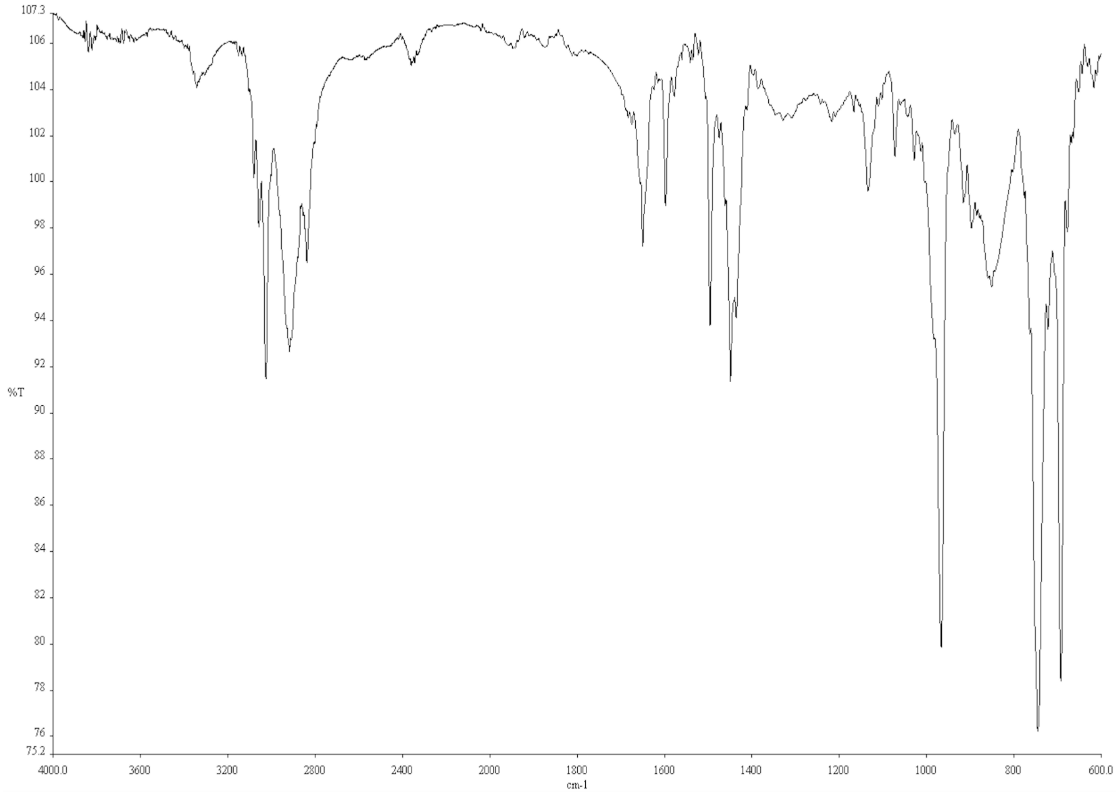

Infrared spectrum (Thin Film, NaCl) of compound **5b**.

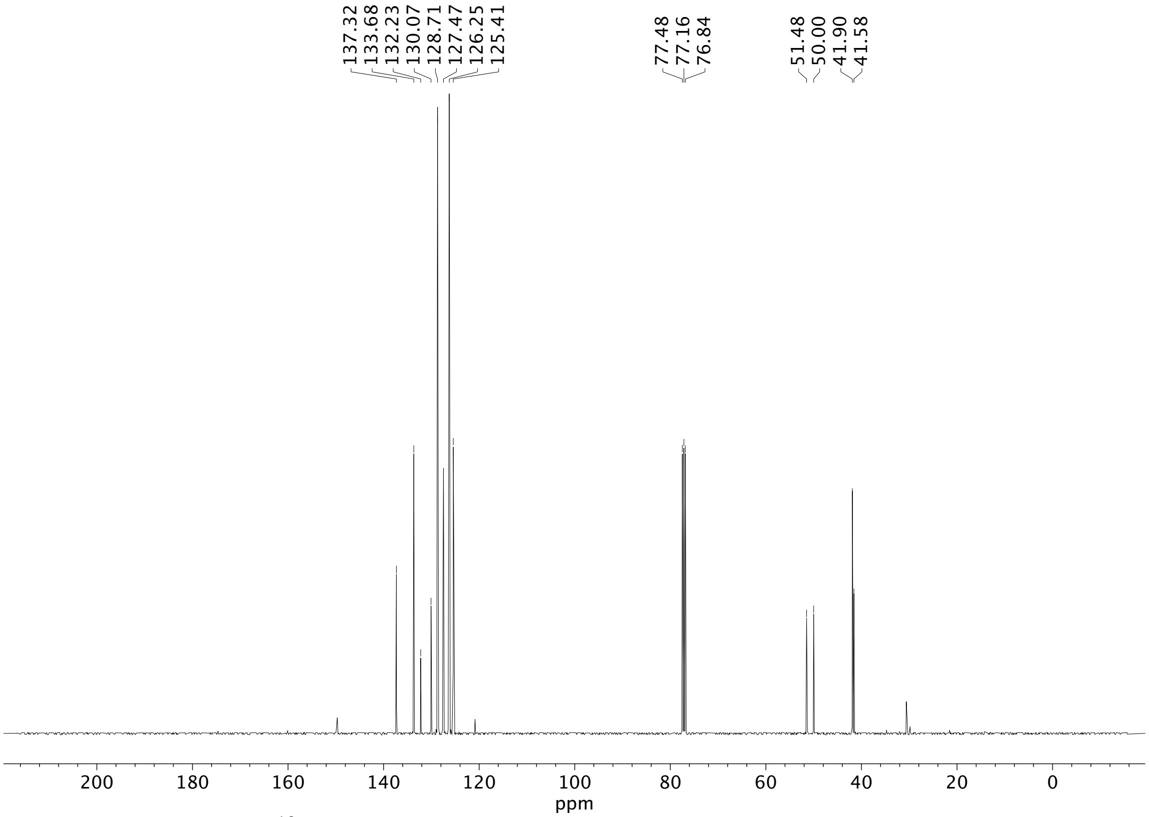

^{13}C NMR (100 MHz, $CDCl_3$) of compound **5b**.

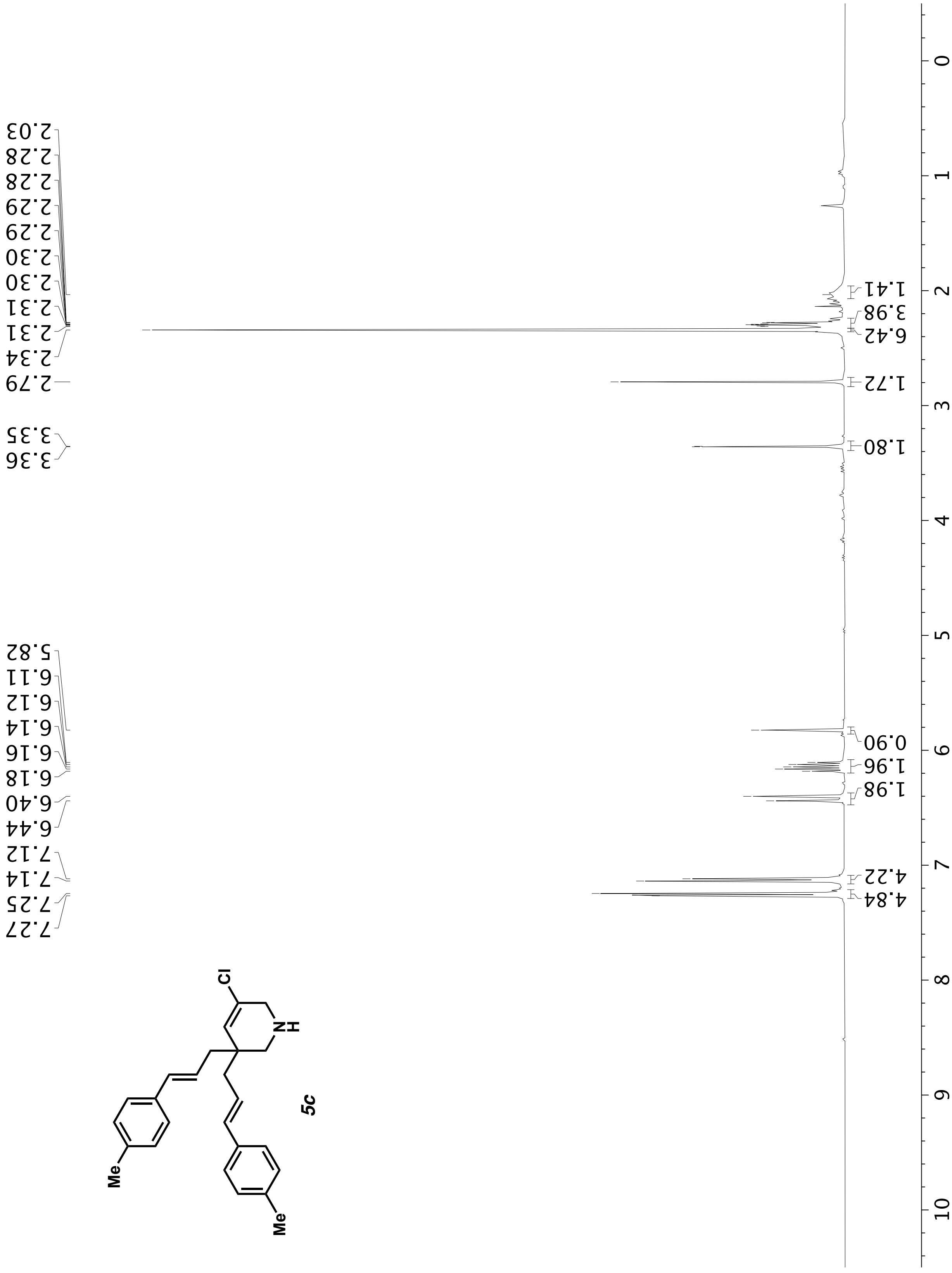

^{1}H NMR (400 MHz, $CDCl_3$) of compound **5c**.

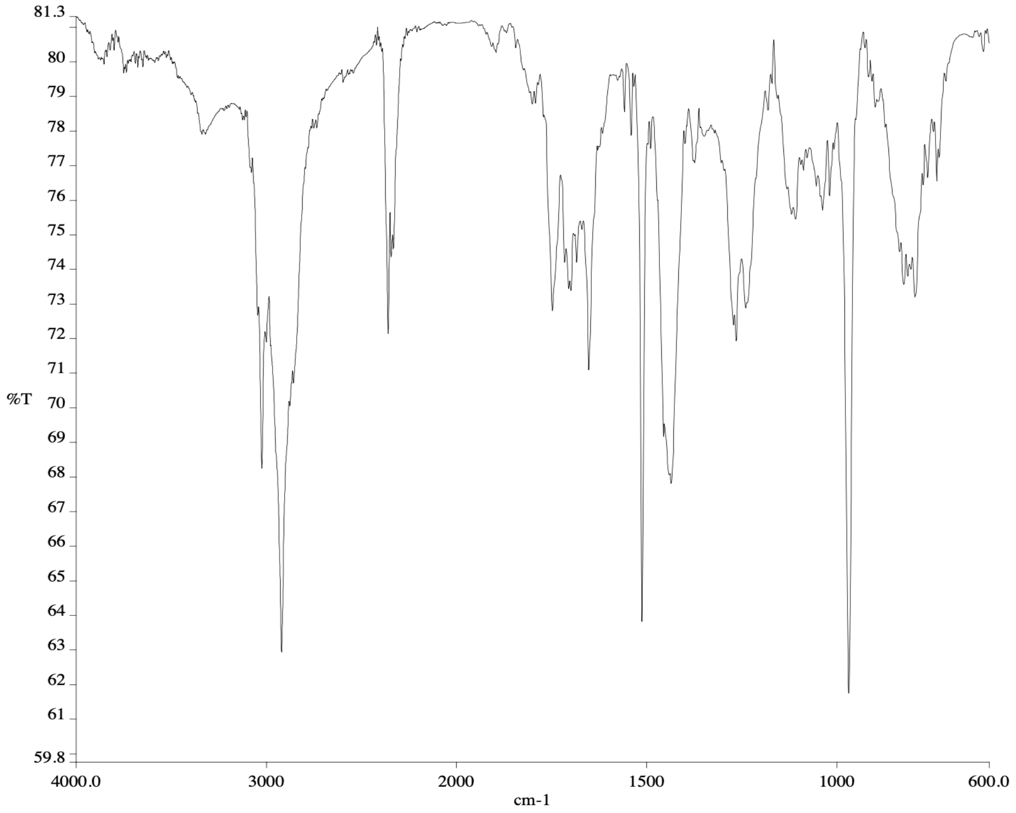

Infrared spectrum (Thin Film, NaCl) of compound **5c**.

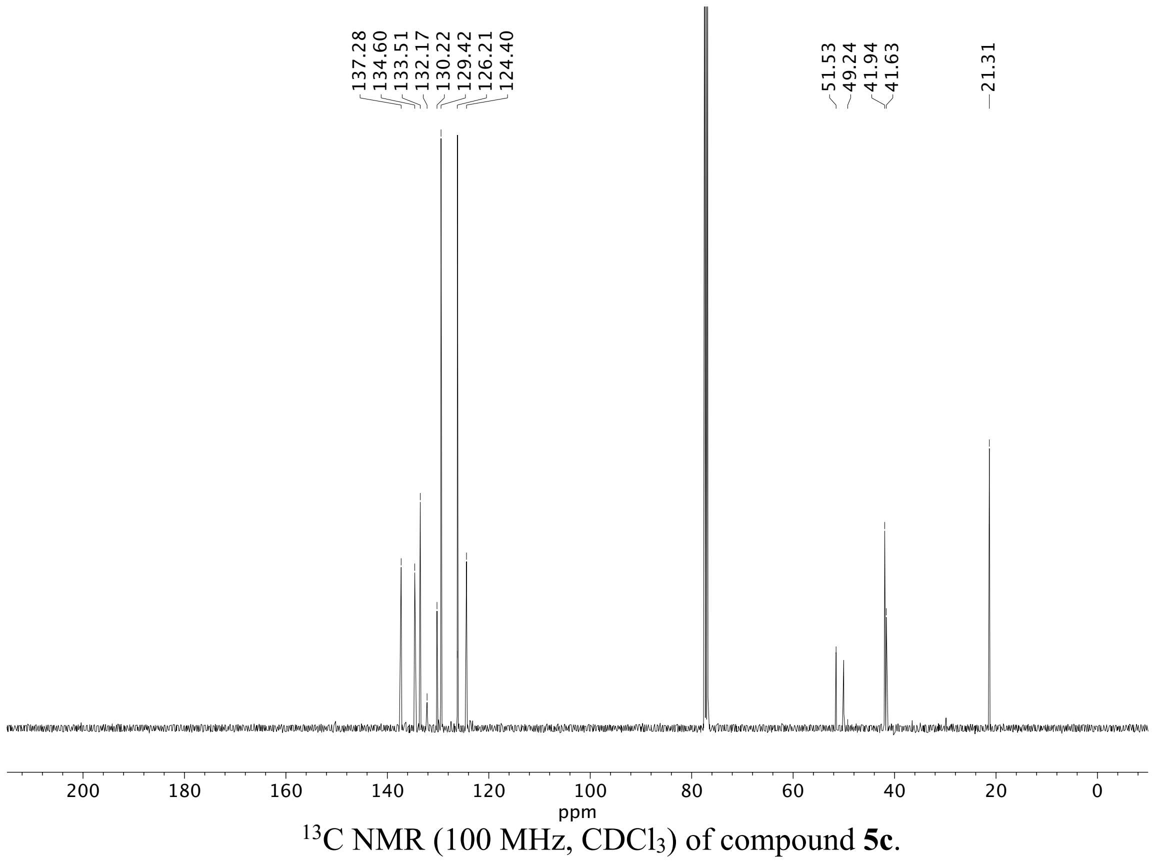

^{13}C NMR (100 MHz, $CDCl_3$) of compound **5c**.

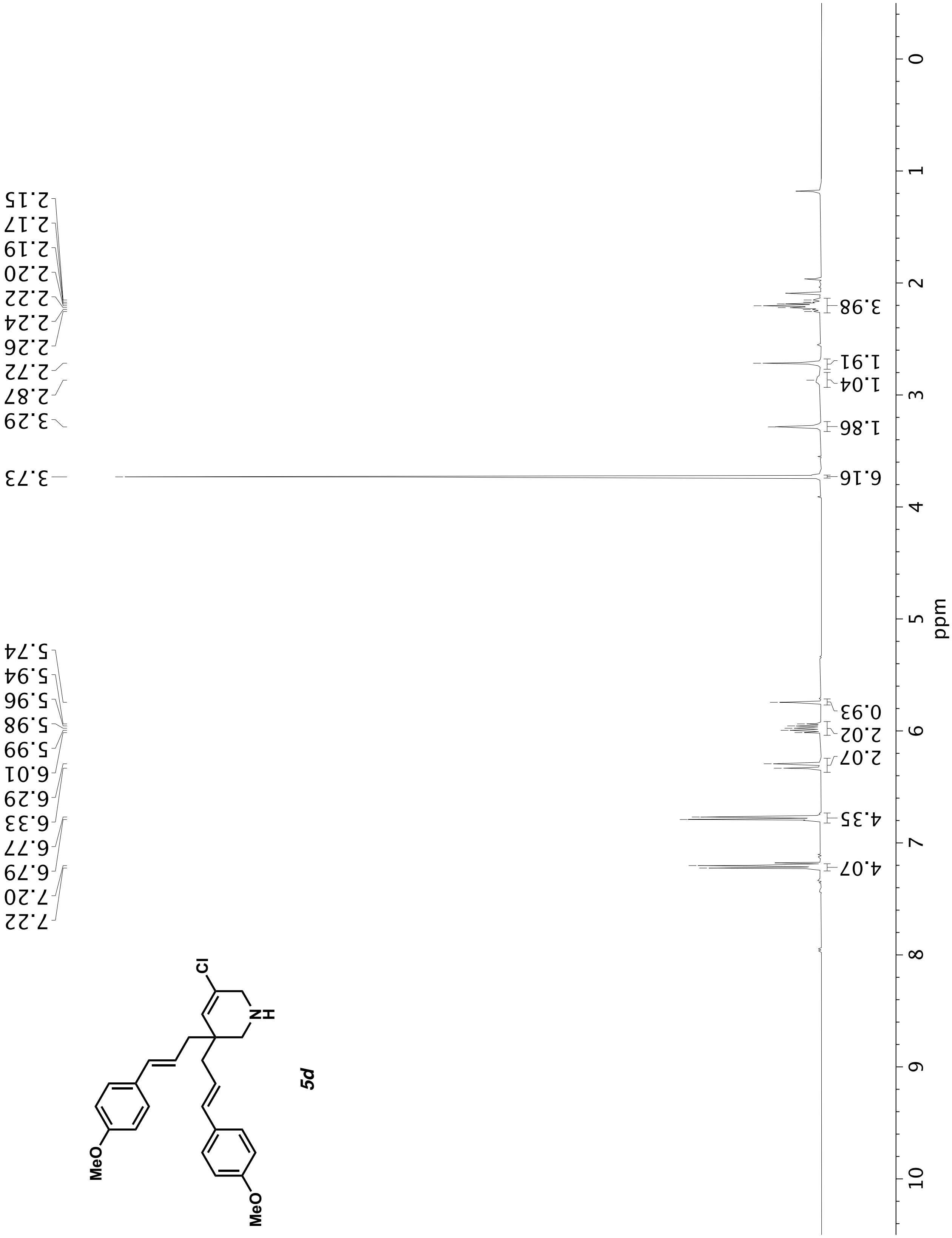

^{1}H NMR (400 MHz, $CDCl_3$) of compound **5d**.

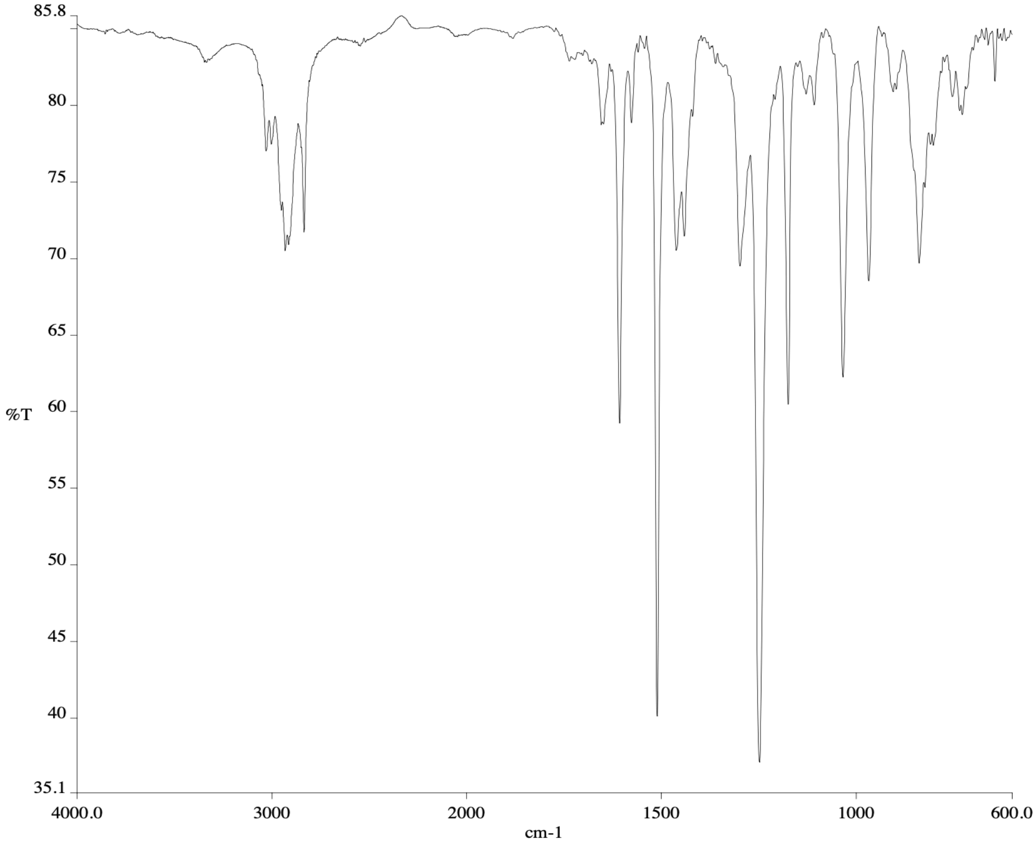

Infrared spectrum (Thin Film, NaCl) of compound **5d**.

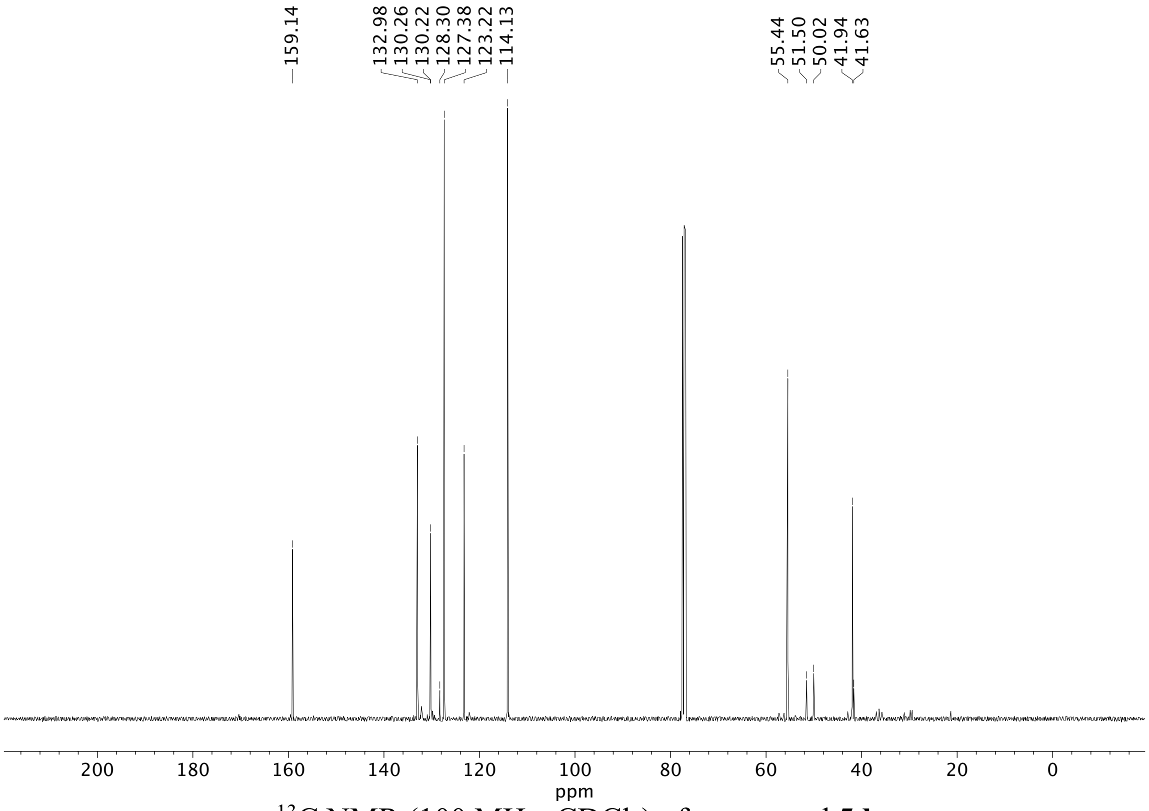

^{13}C NMR (100 MHz, $CDCl_3$) of compound **5d**.

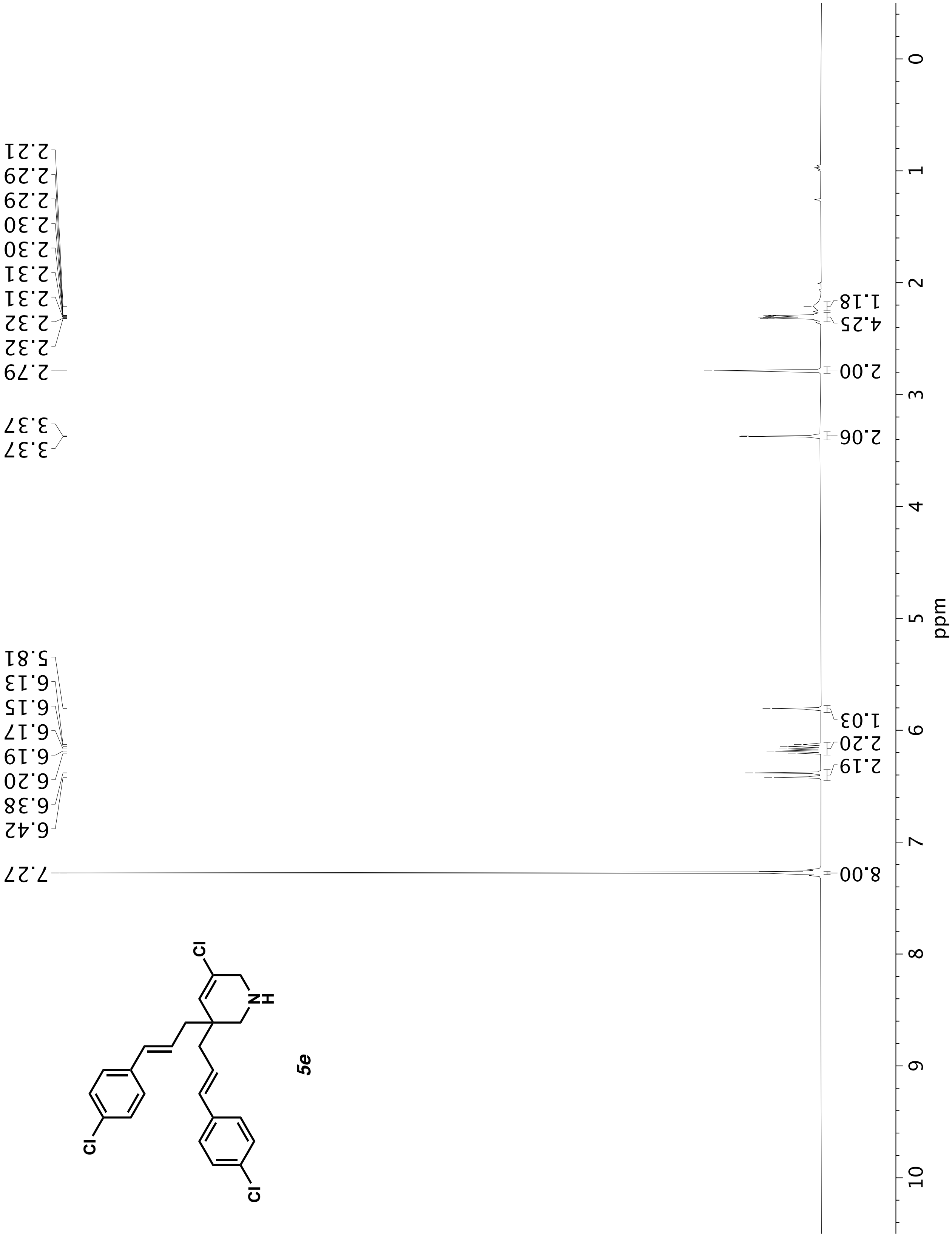

^{1}H NMR (400 MHz, $CDCl_3$) of compound **5e**.

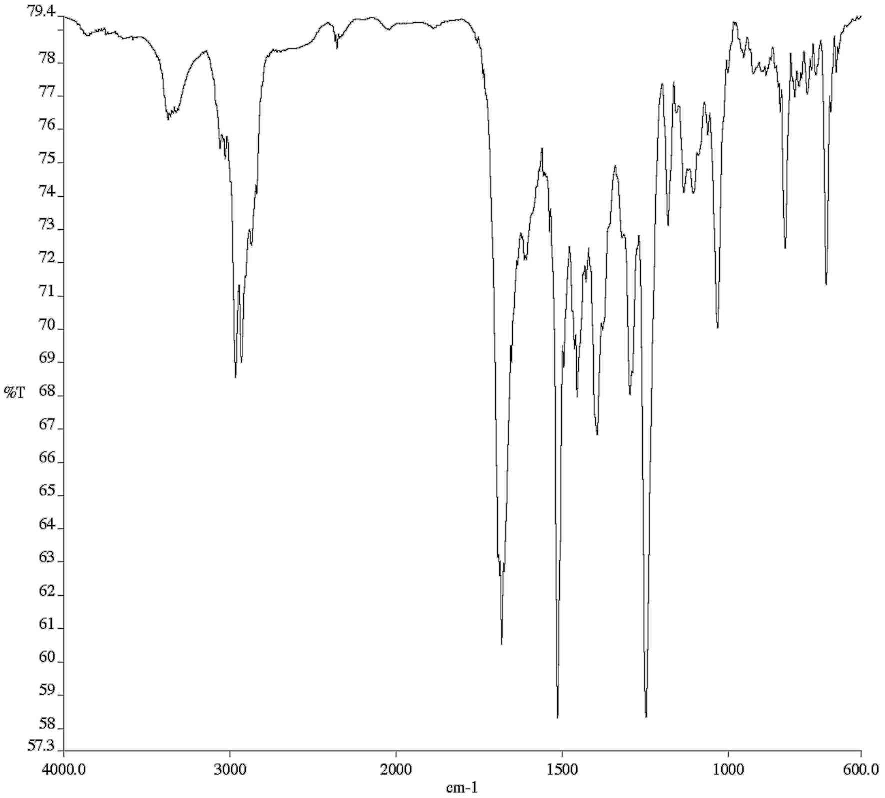

Infrared spectrum (Thin Film, NaCl) of compound **5e**.

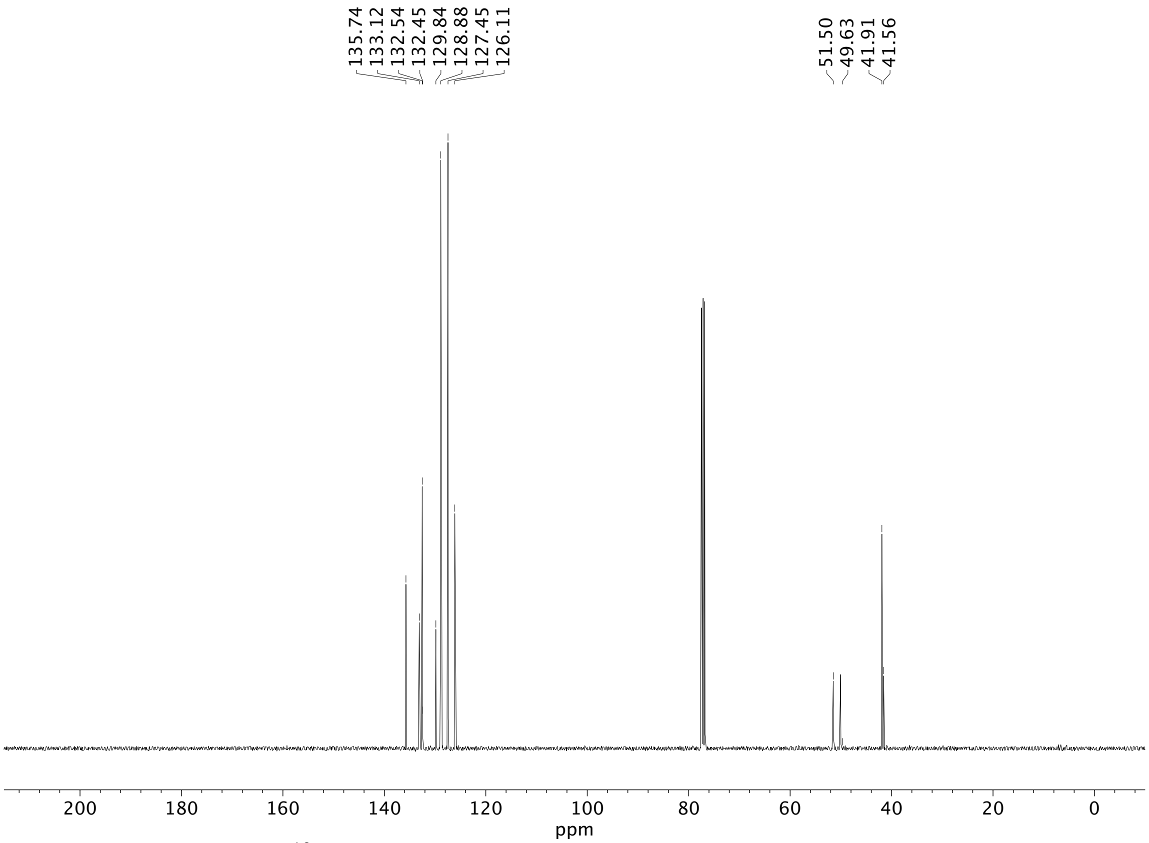

^{13}C NMR (100 MHz, $CDCl_3$) of compound **5e**.

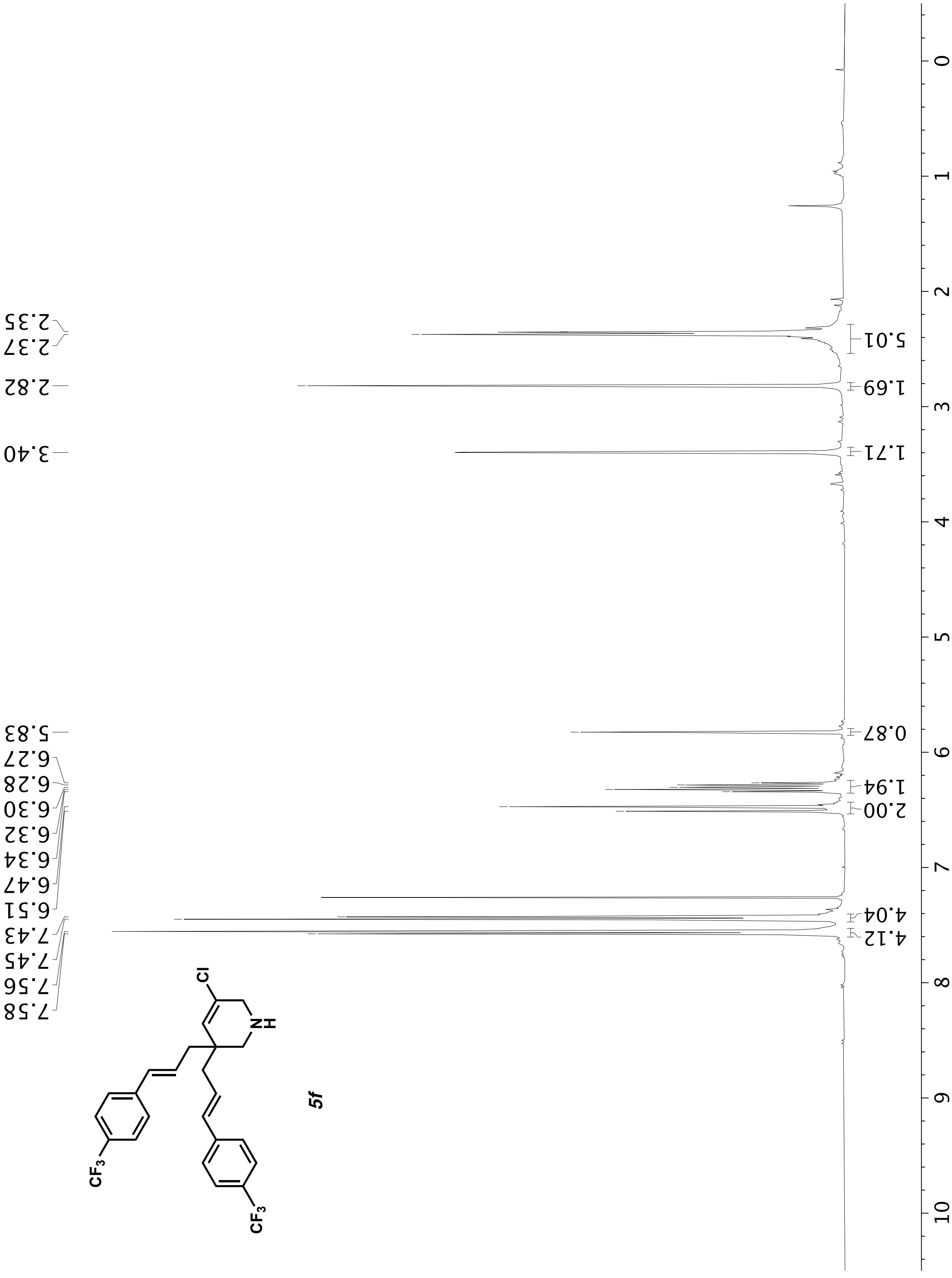

^{1}H NMR (400 MHz, $CDCl_3$) of compound **5f**.

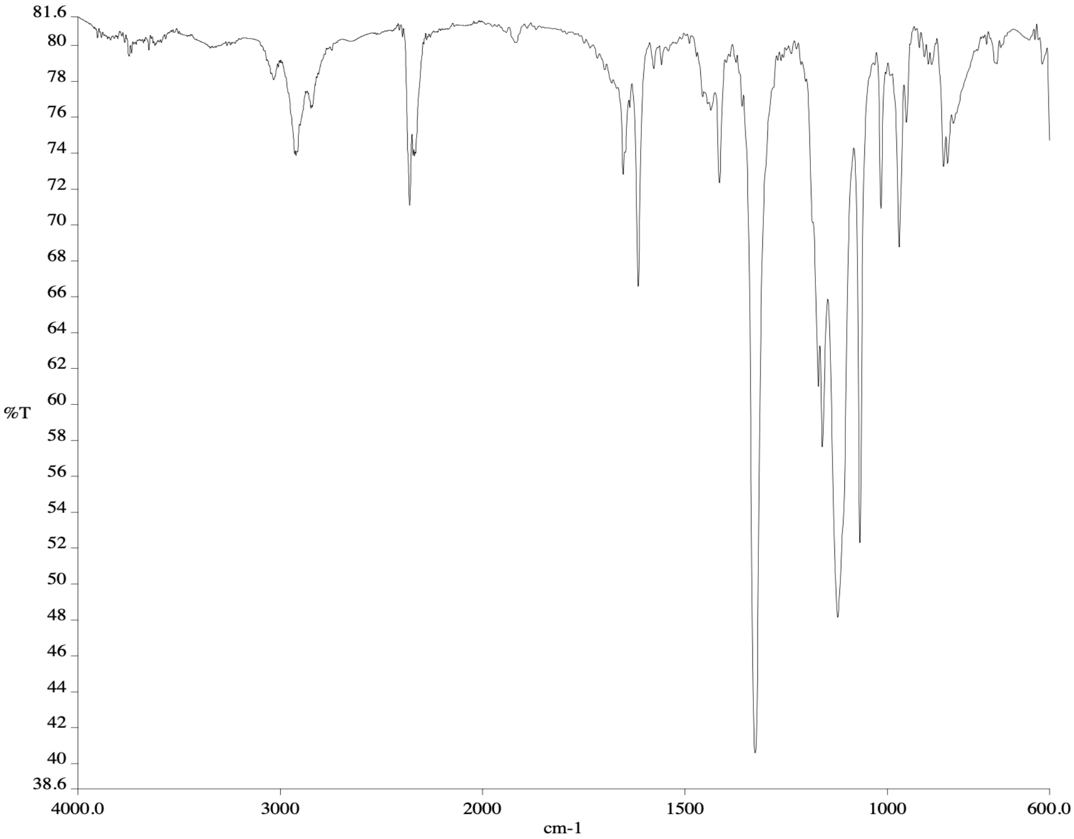

Infrared spectrum (Thin Film, NaCl) of compound **5f**.

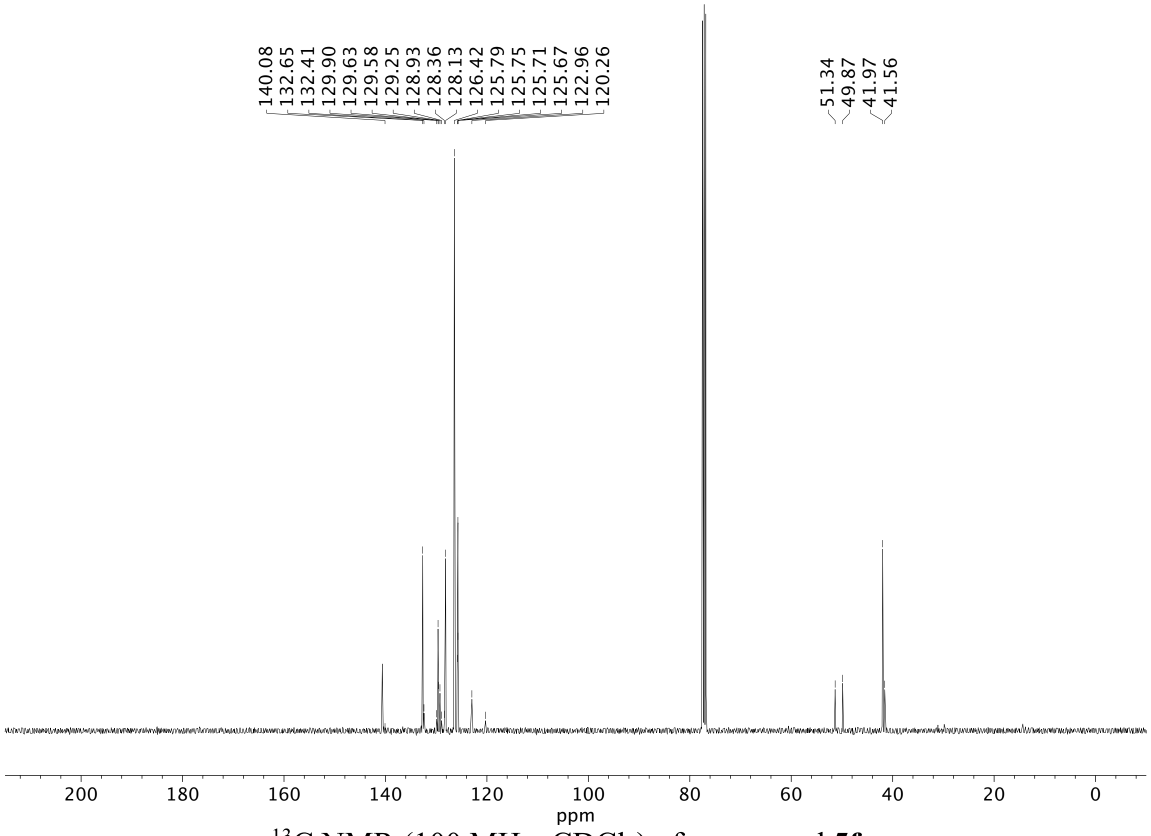

^{13}C NMR (100 MHz, $CDCl_3$) of compound **5f**.

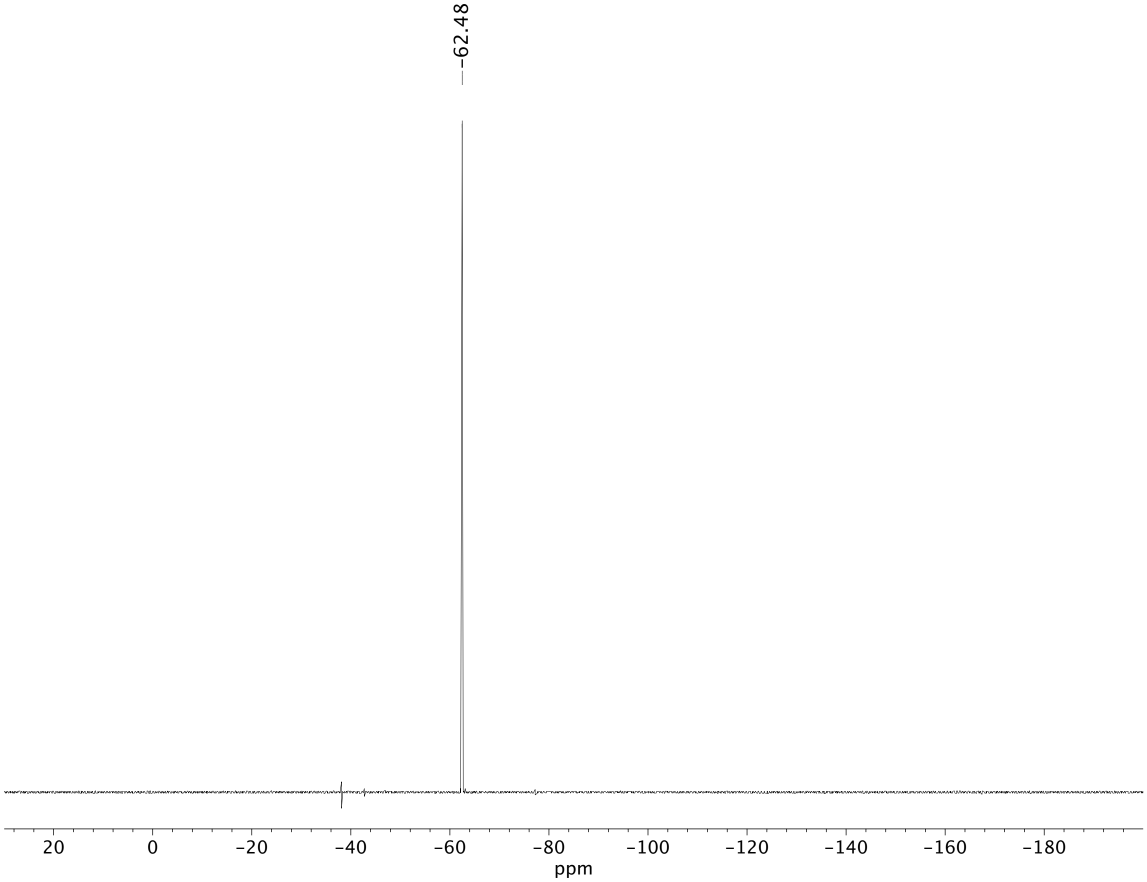

^{19}F NMR (282 MHz, $CDCl_3$) of compound **5f**.

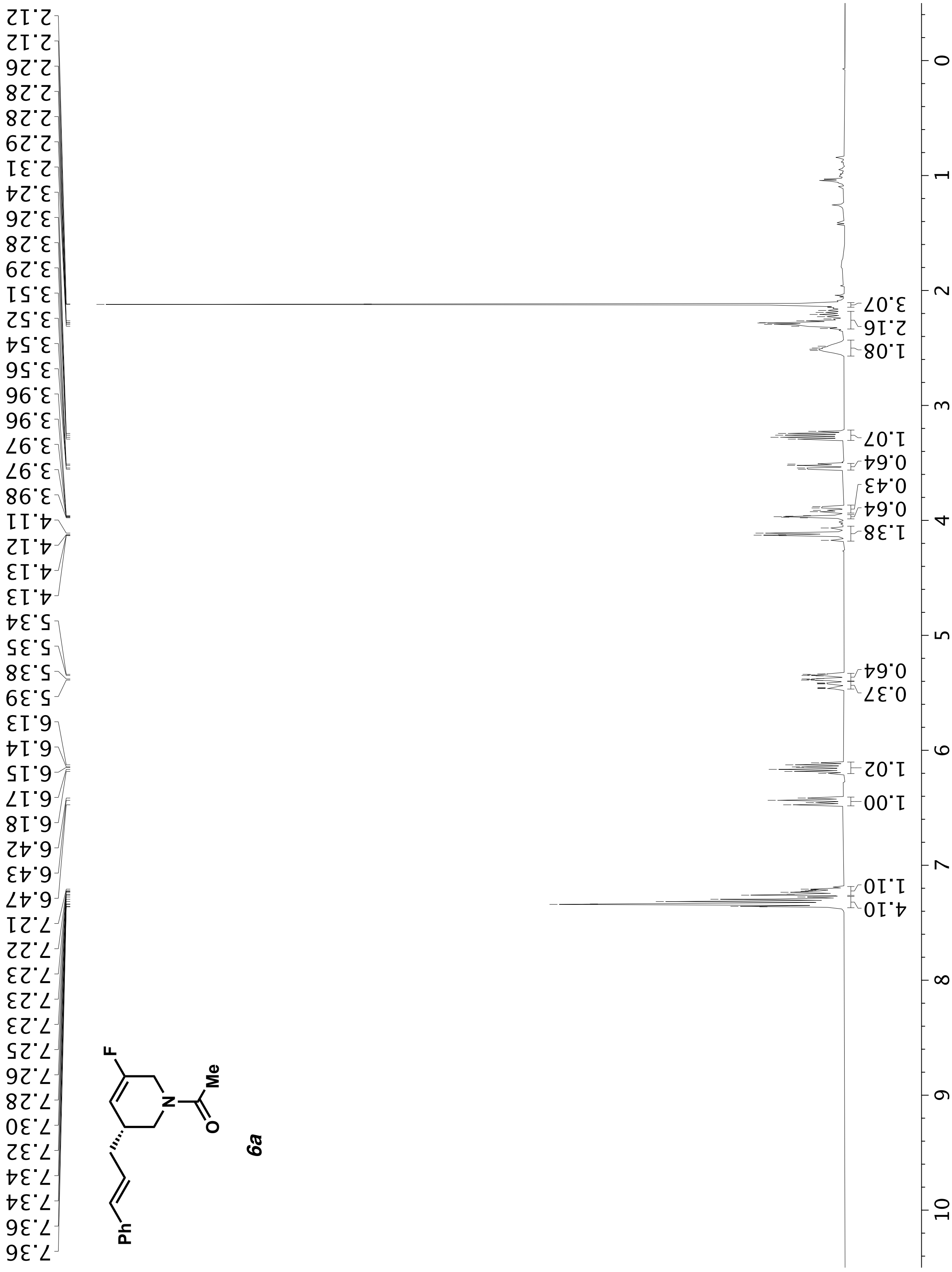

^{1}H NMR (400 MHz, $CDCl_3$) of compound **6a**.

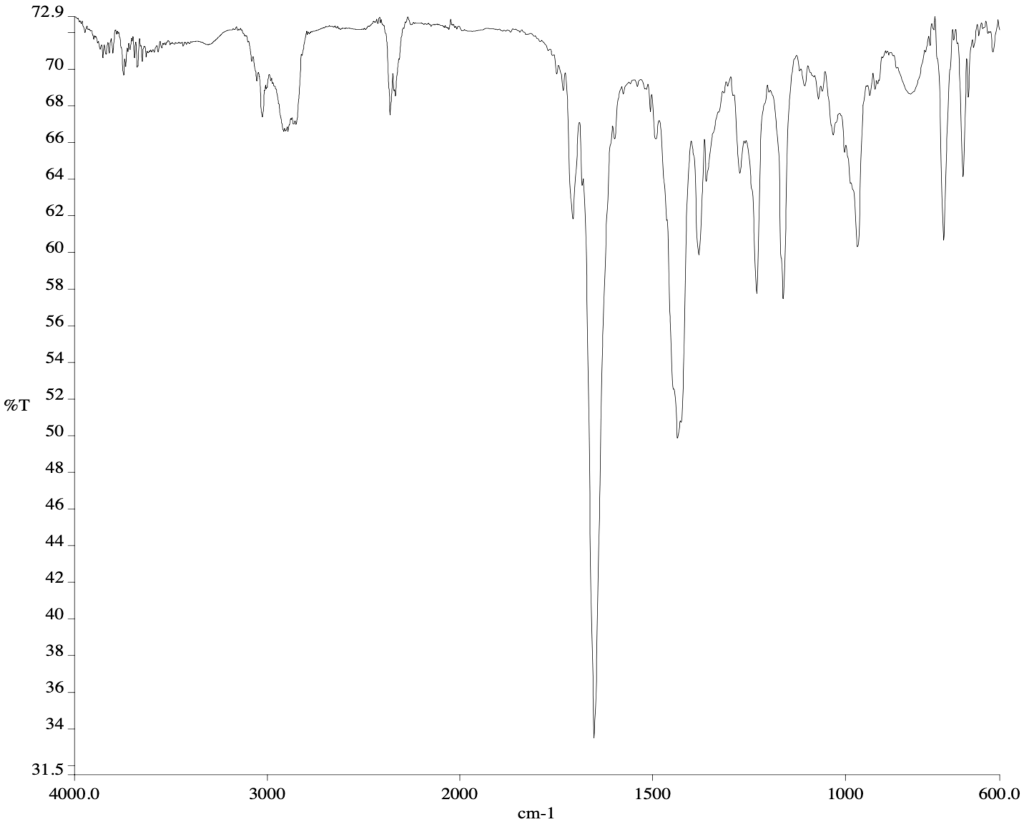

Infrared spectrum (Thin Film, NaCl) of compound **6a**.

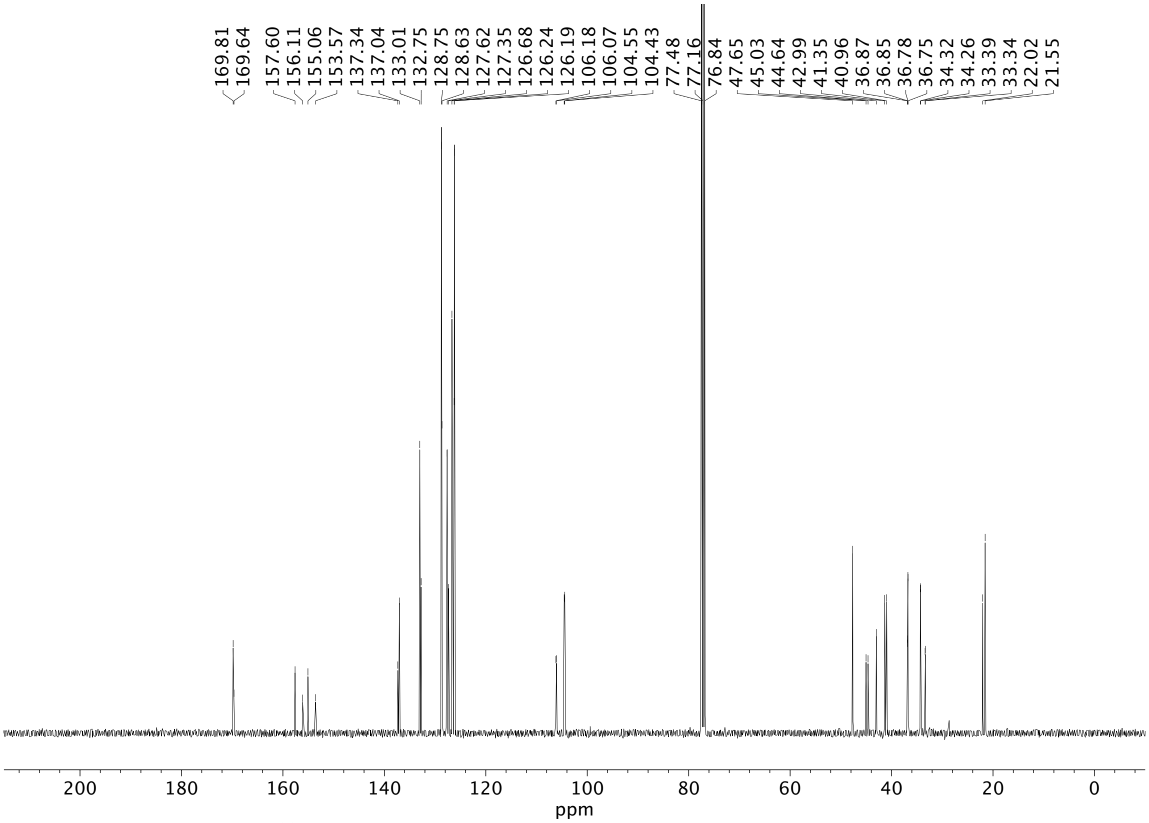

^{13}C NMR (100 MHz, $CDCl_3$) of compound **6a**.

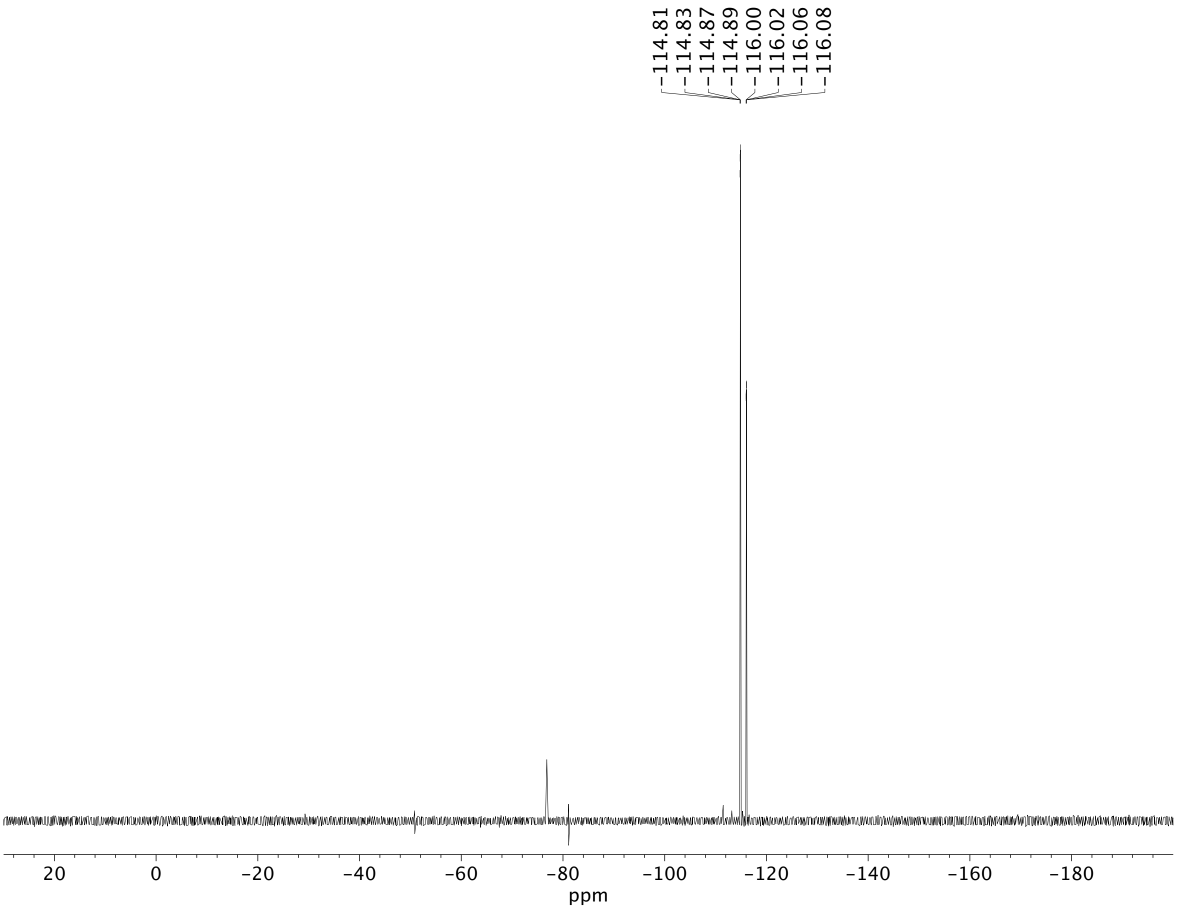

^{19}F NMR (282 MHz, CD_3OD) of compound **6a.**

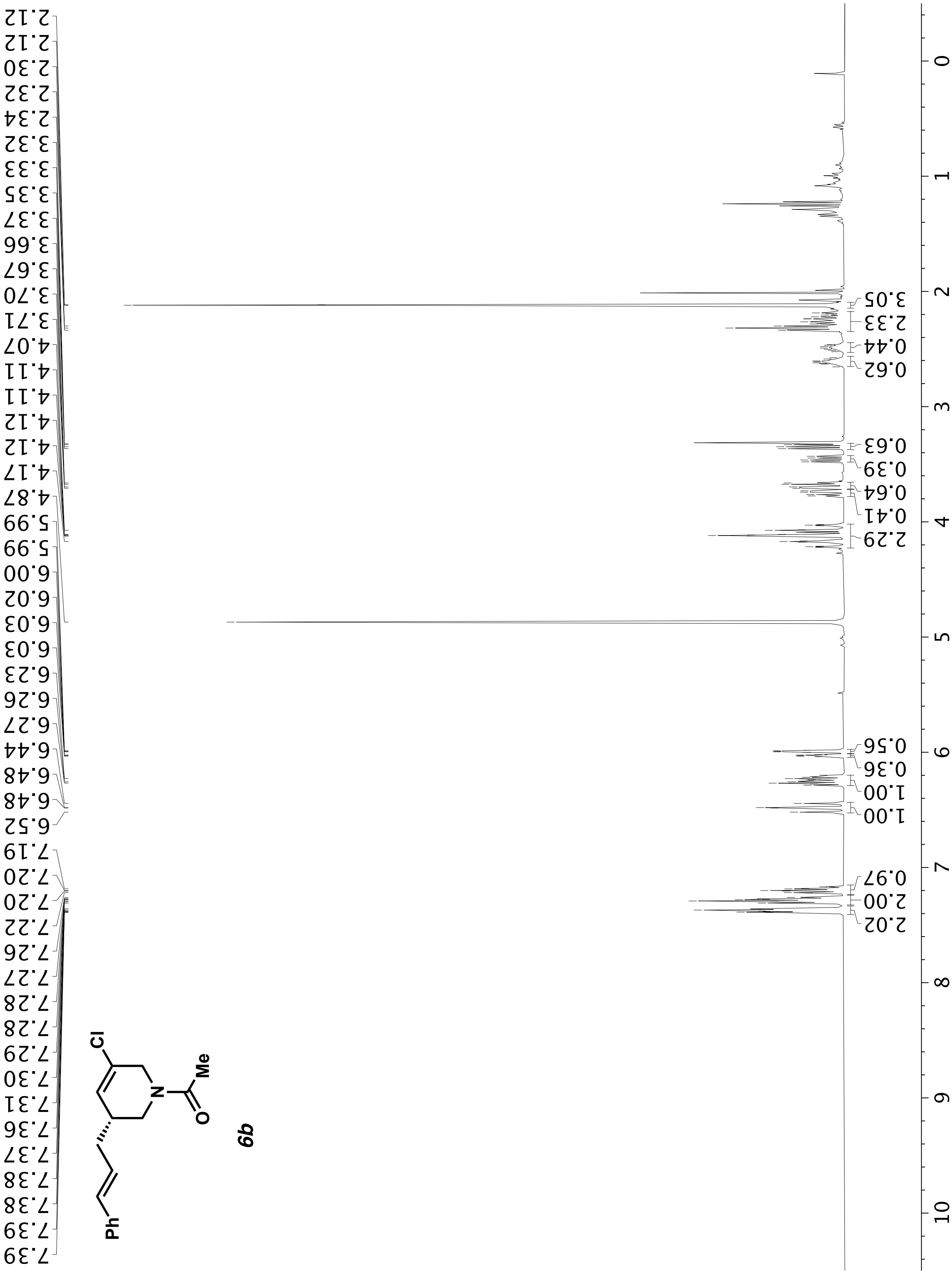

^{1}H NMR (400 MHz, CD_3OD) of compound **6b**

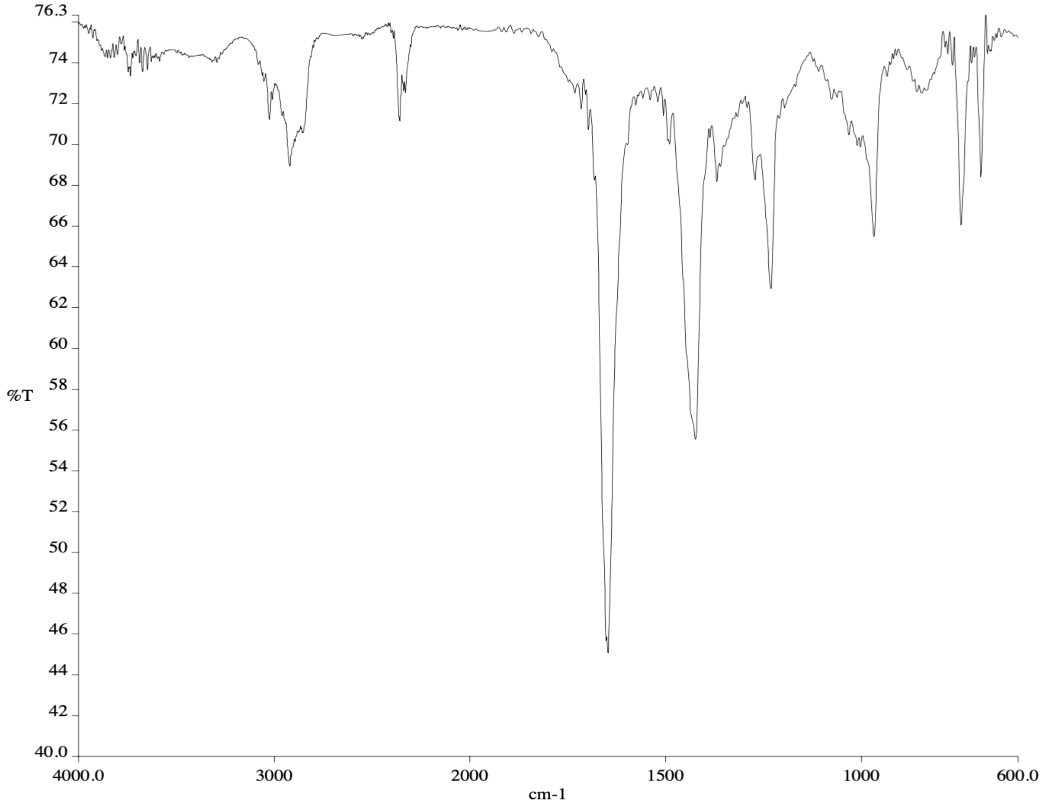

Infrared spectrum (Thin Film, NaCl) of compound **6b**.

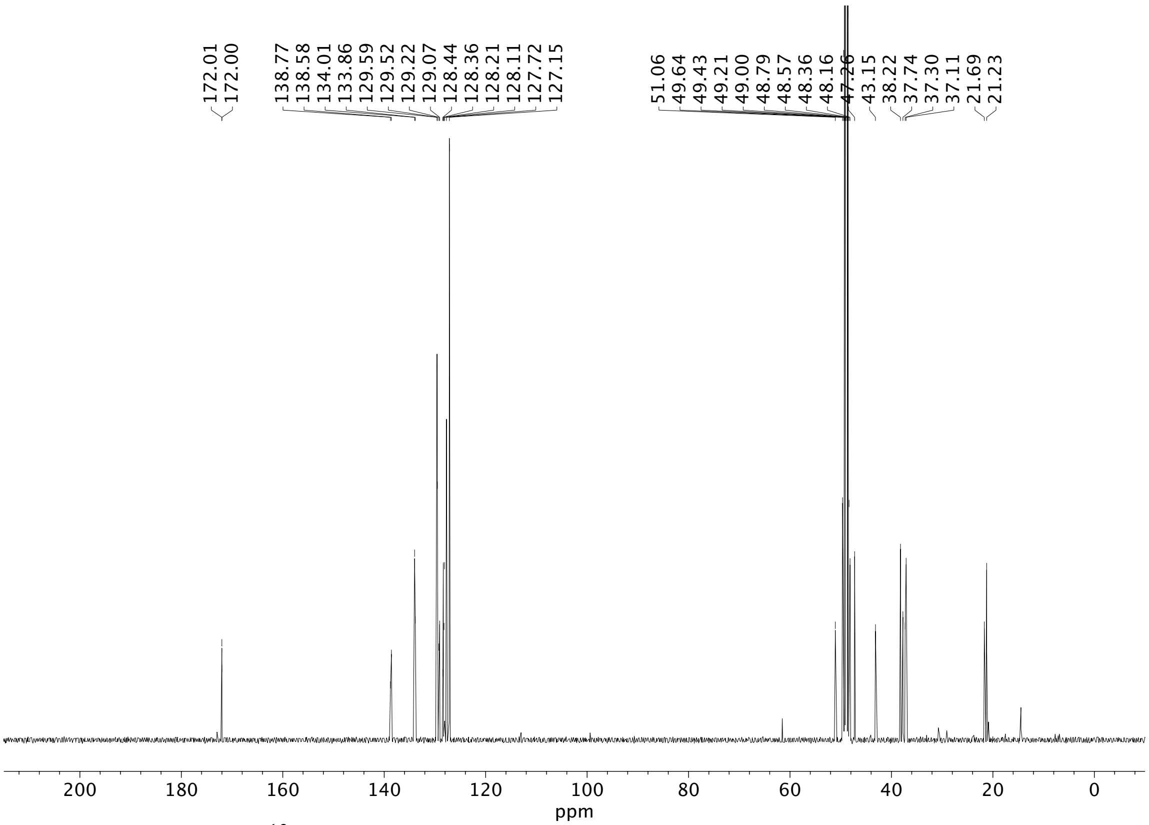

^{13}C NMR (100 MHz, CD_3OD) of compound **6b**.

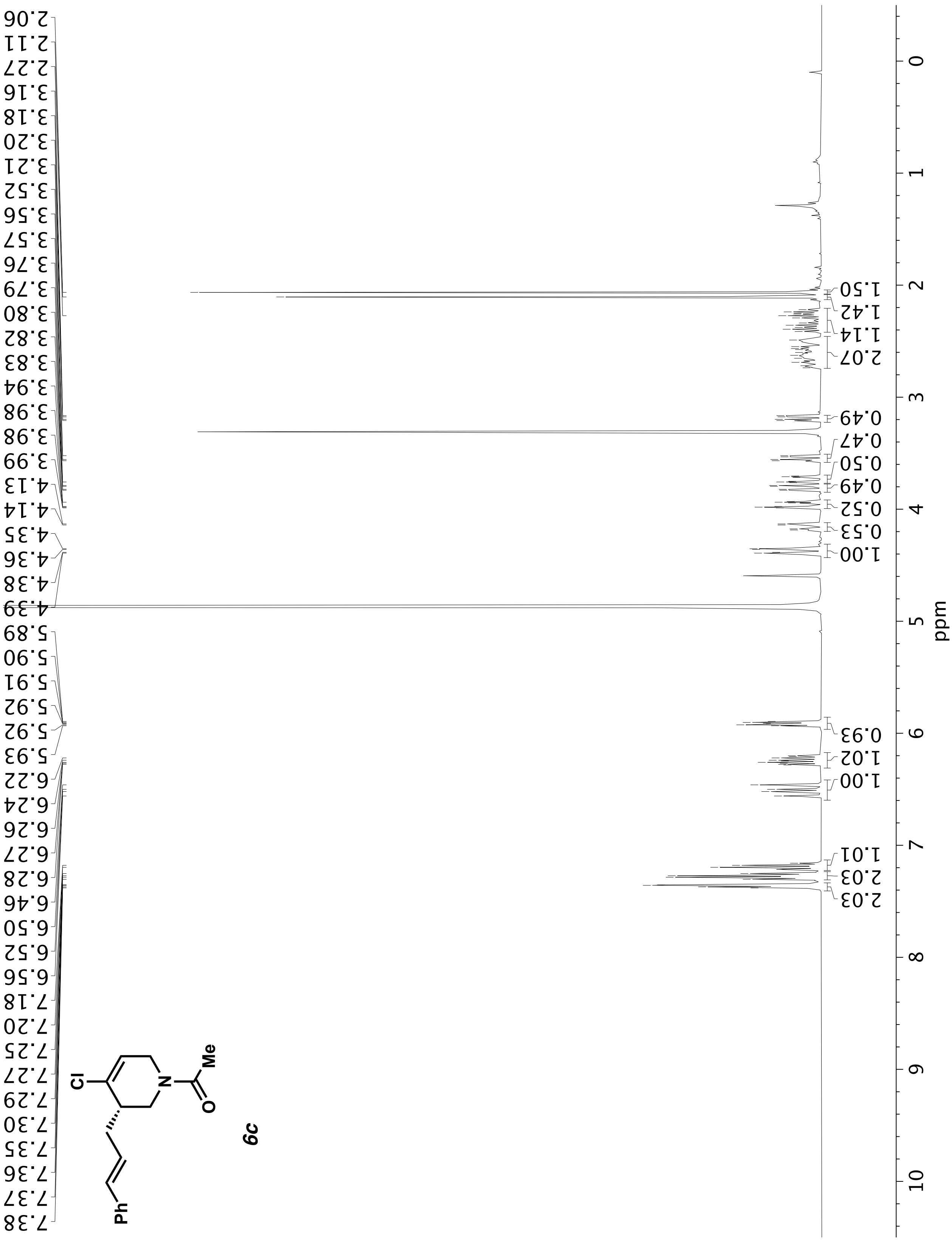

^{1}H NMR (500 MHz, CD_3OD) of compound **6c**.

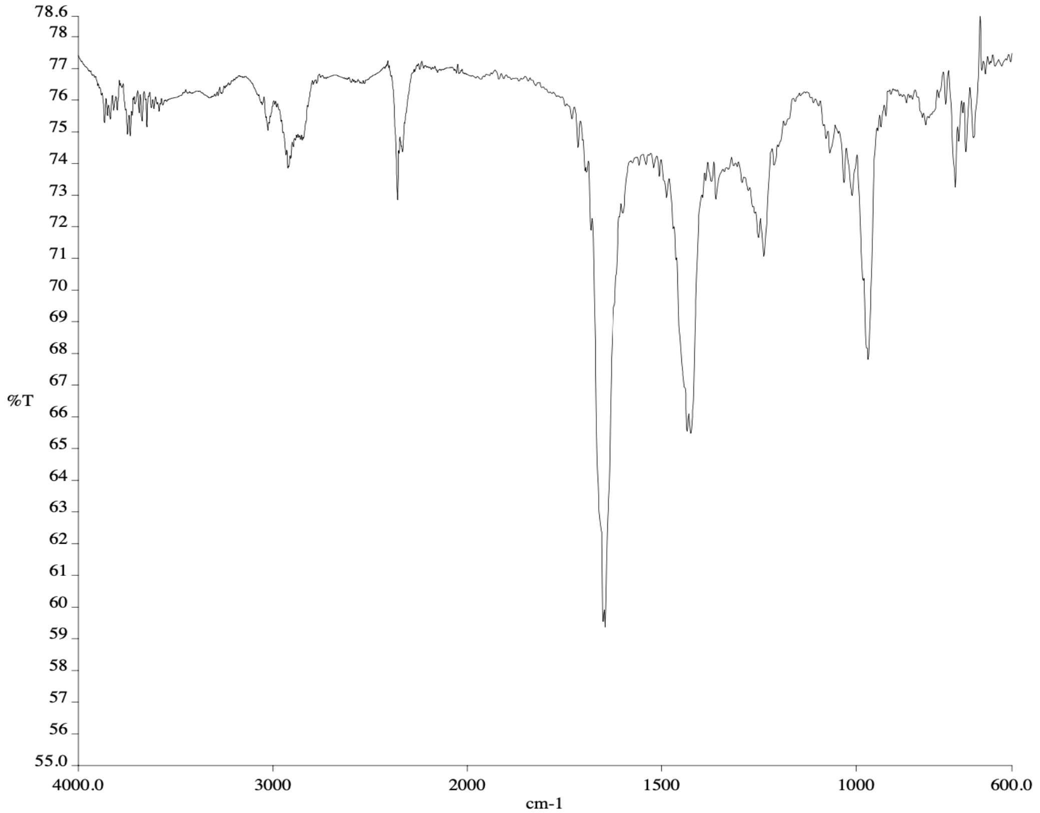

Infrared spectrum (Thin Film, NaCl) of compound **6c**.

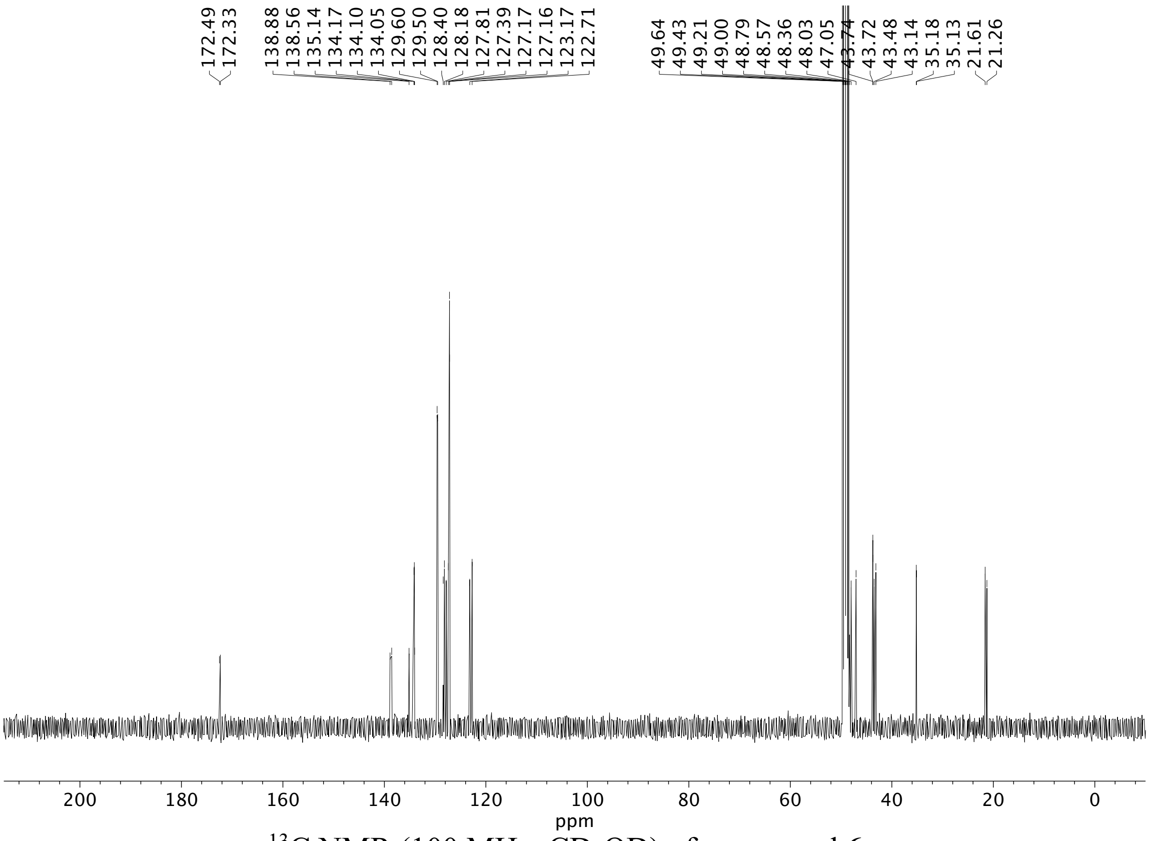

^{13}C NMR (100 MHz, CD_3OD) of compound **6c**.

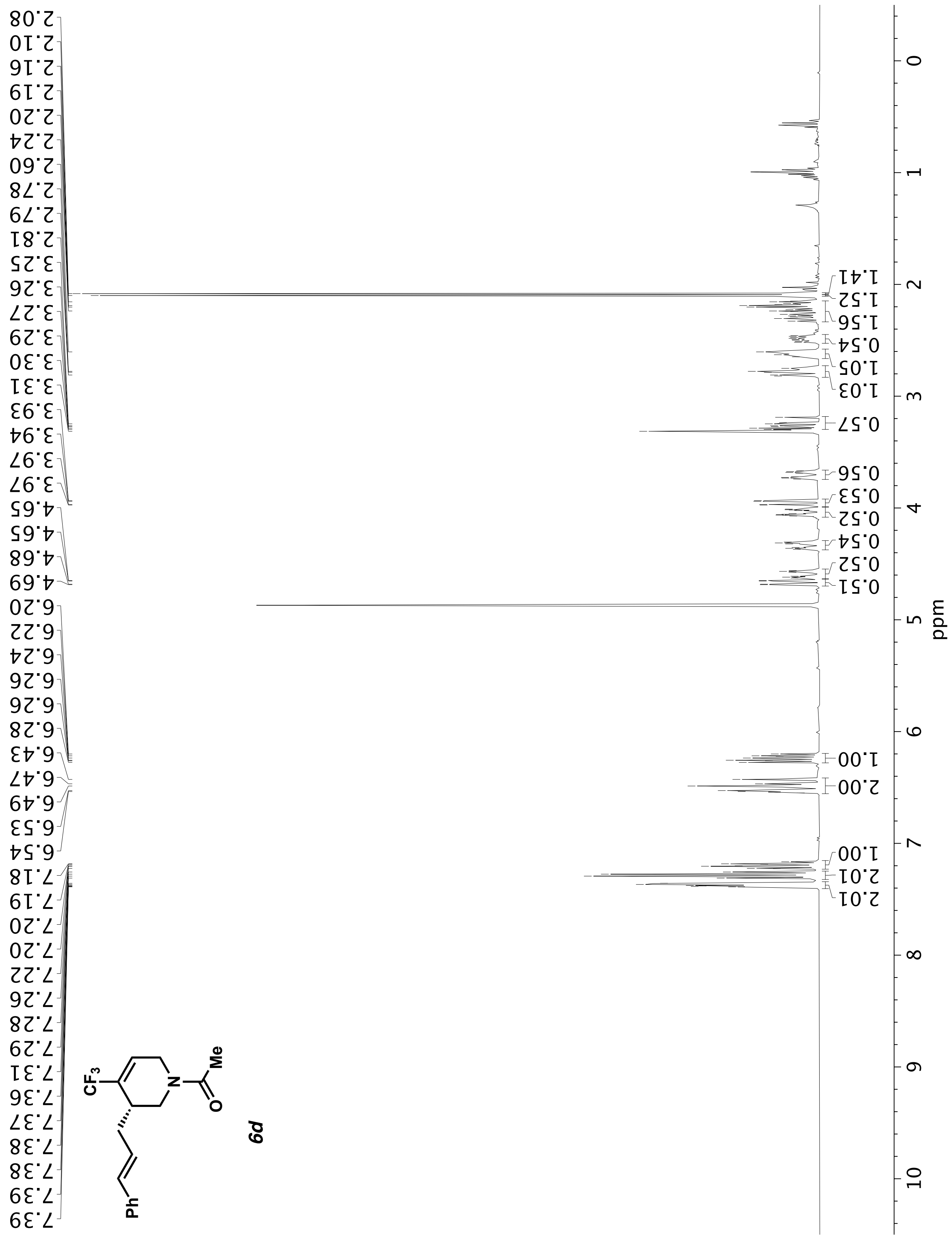

^{1}H NMR (400 MHz, CD_3OD) of compound **6d**.

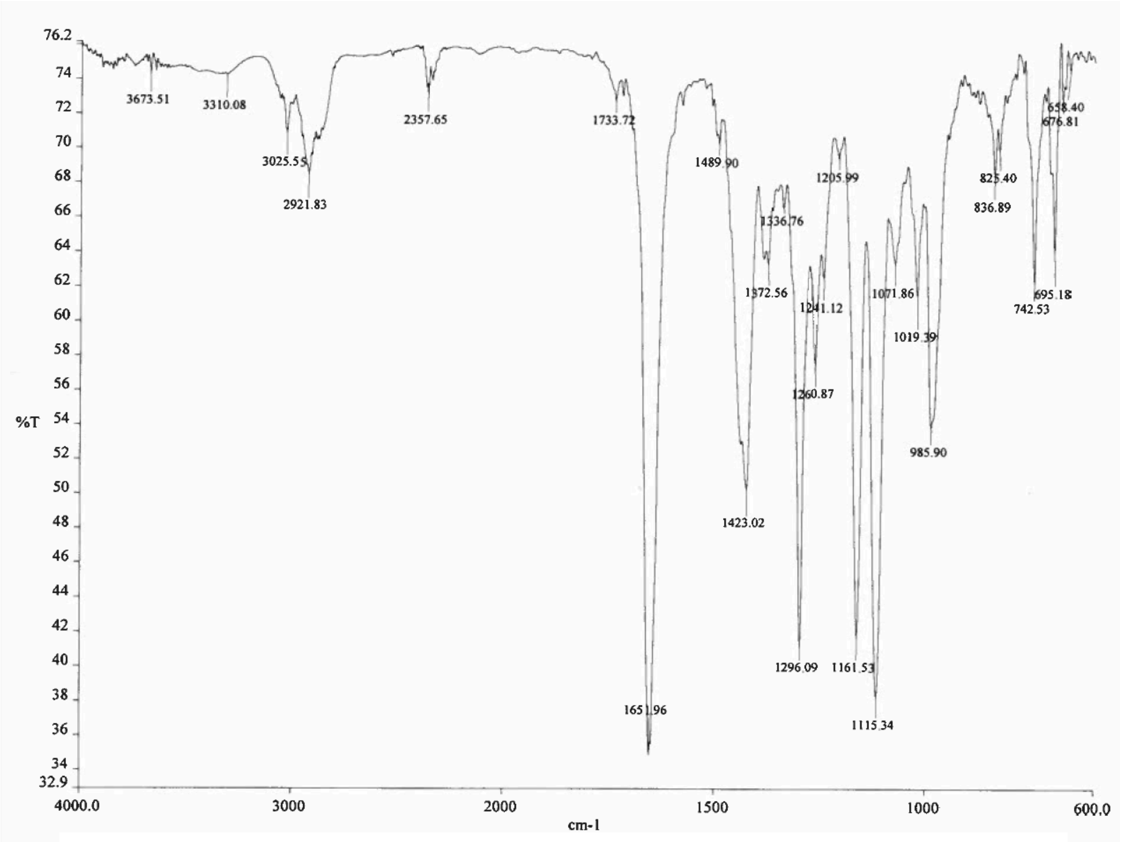

Infrared spectrum (Thin Film, NaCl) of compound **6d**.

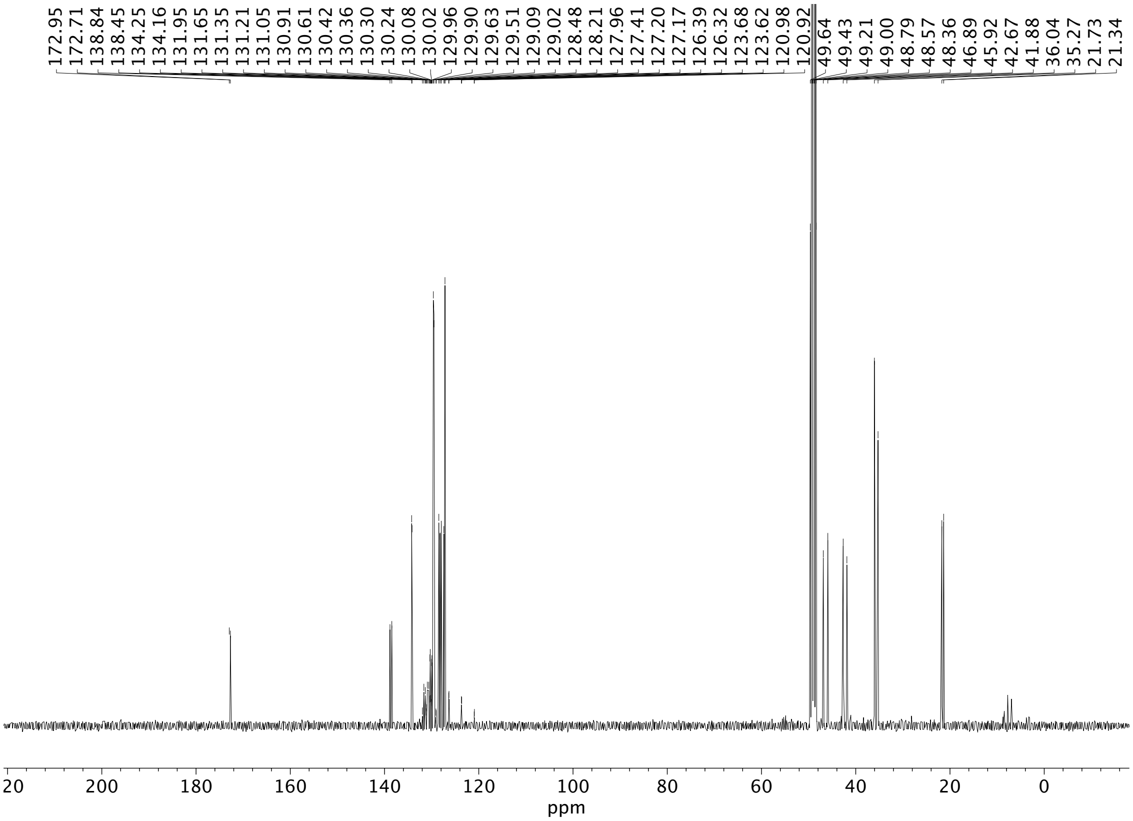

^{13}C NMR (100 MHz, CD_3OD) of compound **6d**.

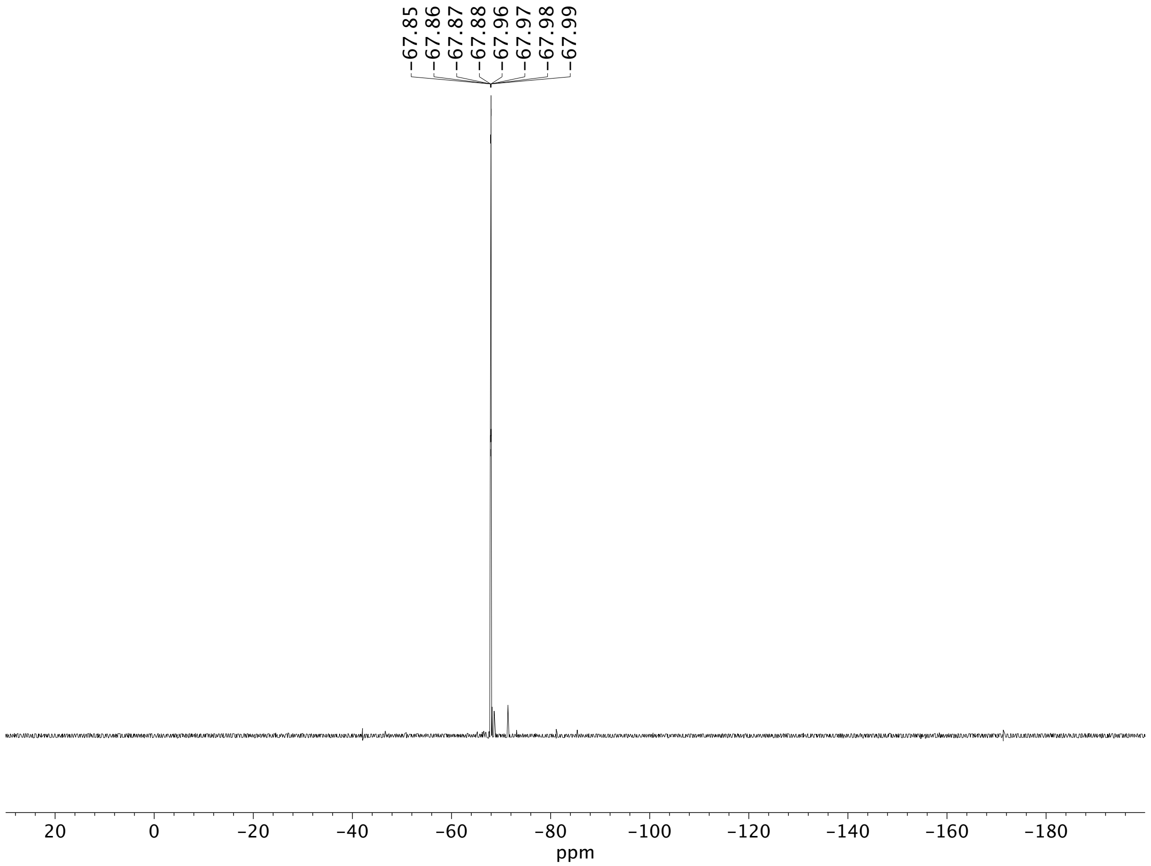

^{19}F NMR (282 MHz, CD_3OD) of compound **6d**.

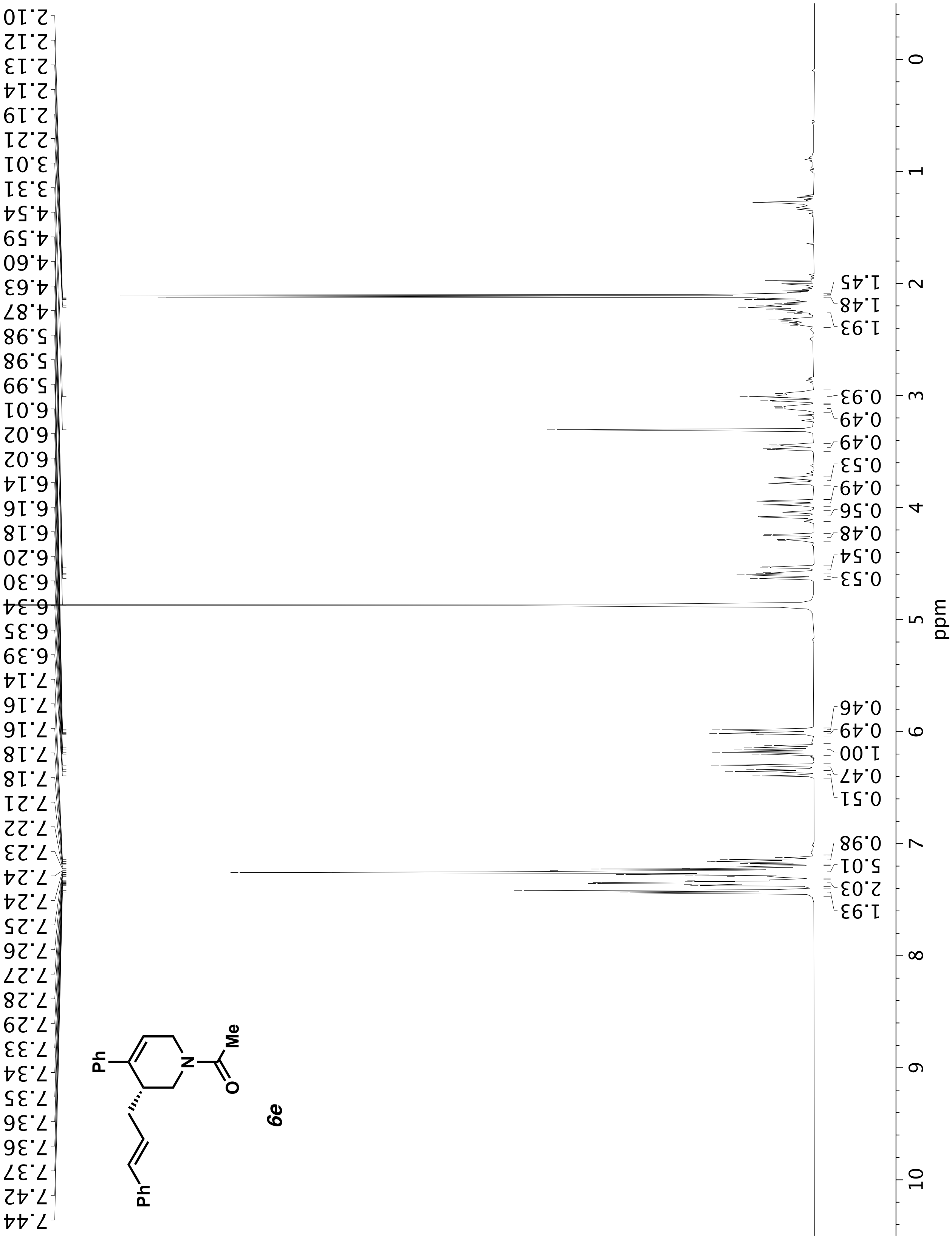

^{1}H NMR (400 MHz, CD_3OD) of compound **6e**.

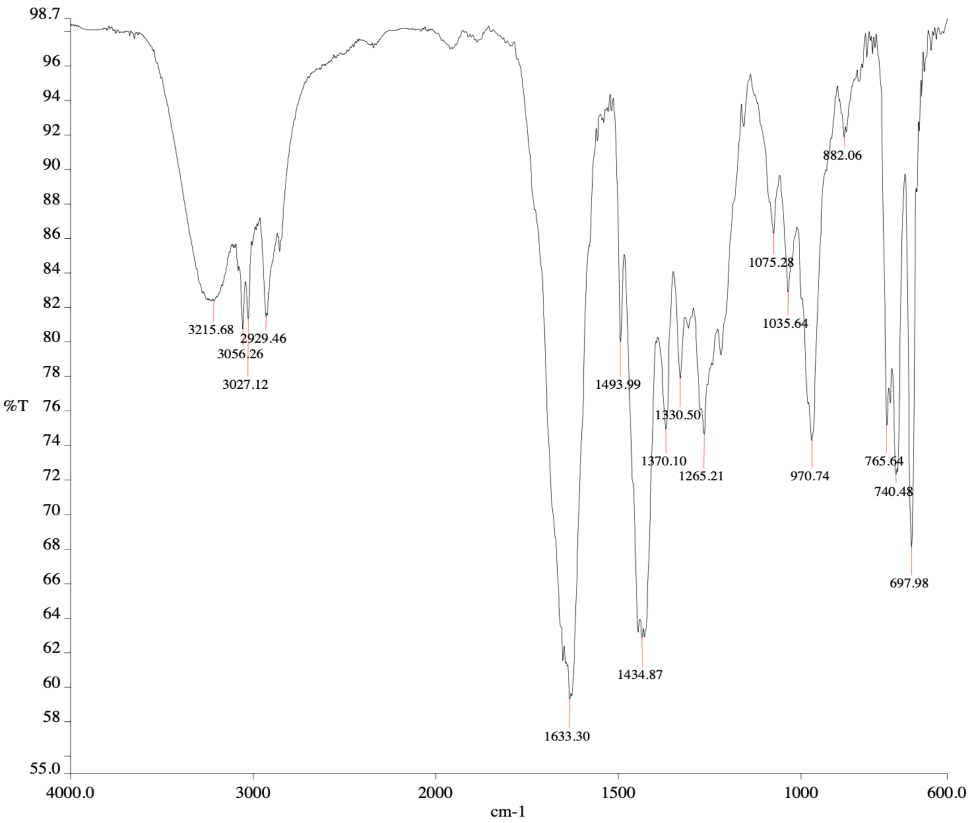

Infrared spectrum (Thin Film, NaCl) of compound **6e**.

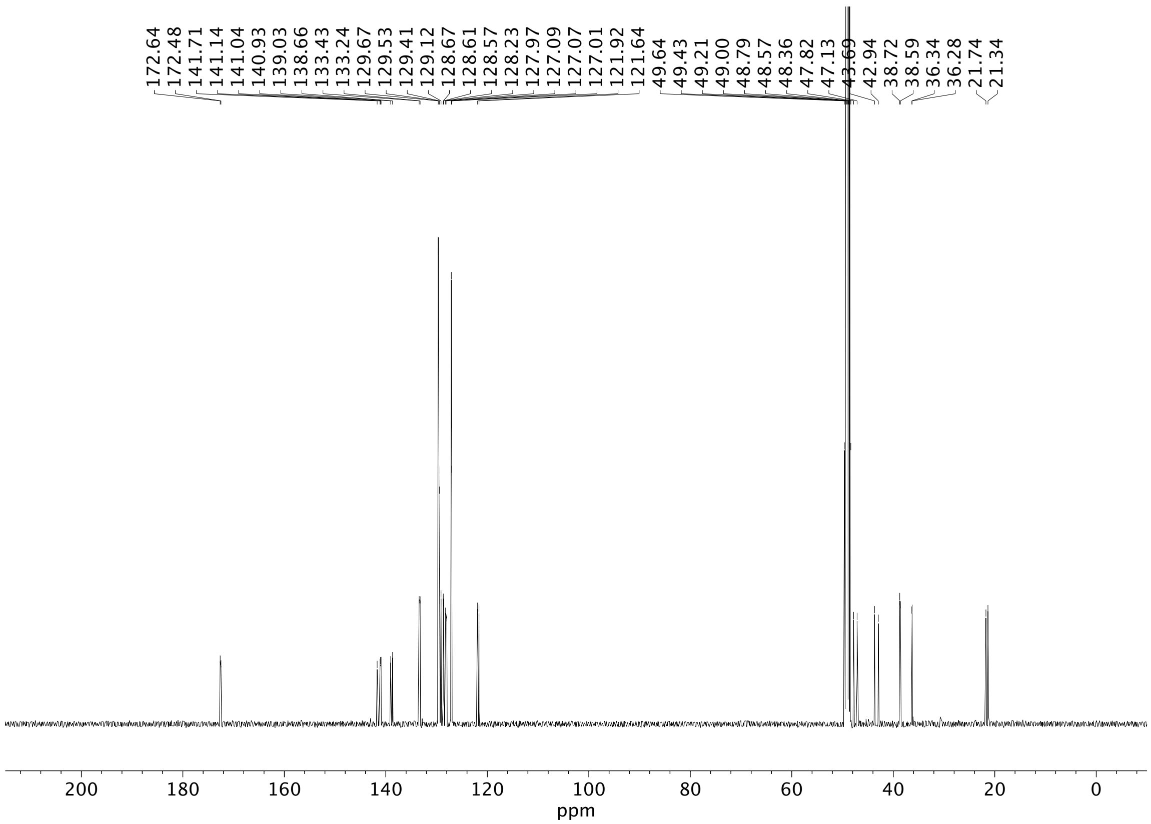

^{13}C NMR (100 MHz, CD_3OD) of compound **6e**.

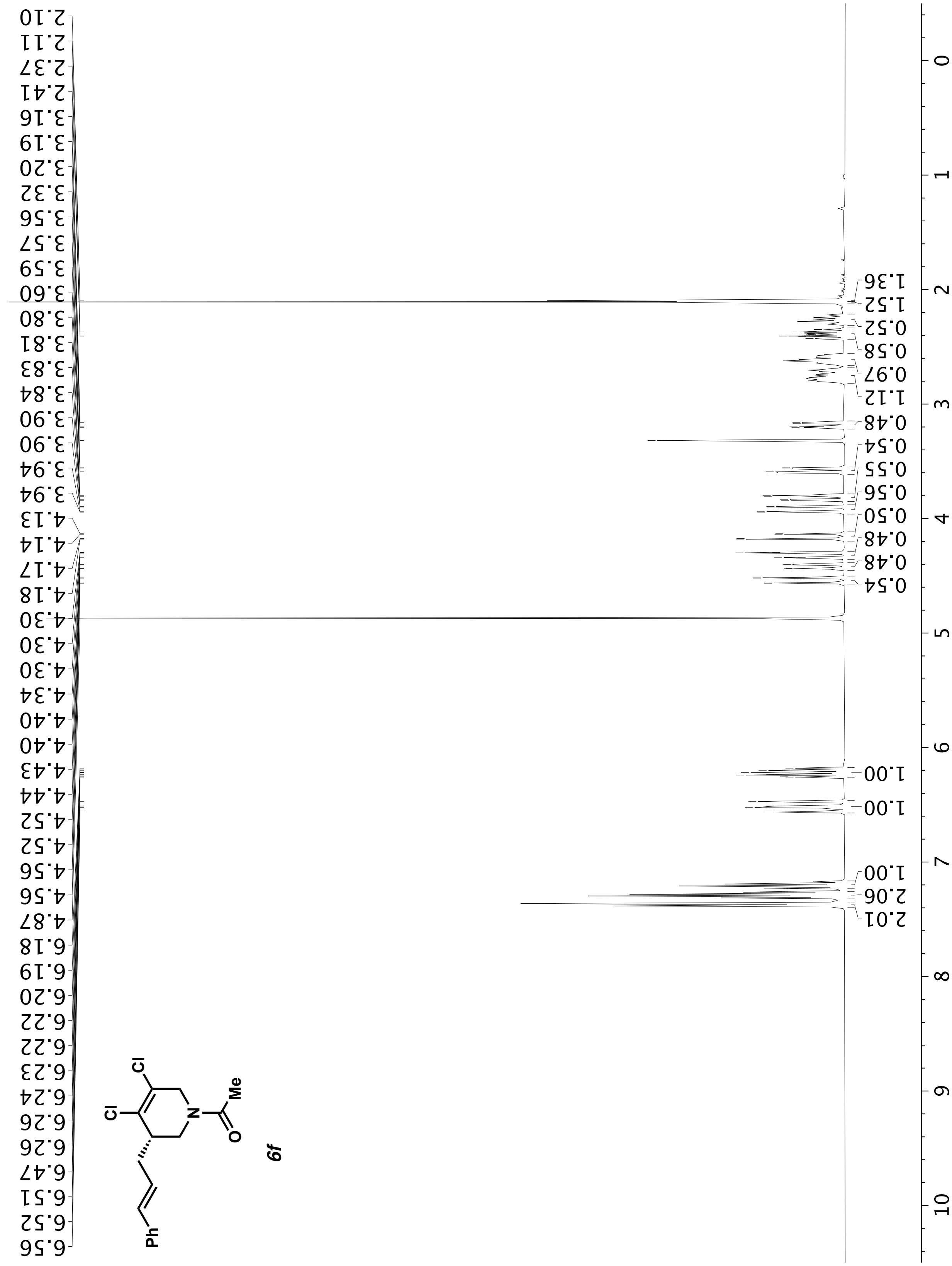

¹H NMR (400 MHz, CD_3OD) of compound **6f**.

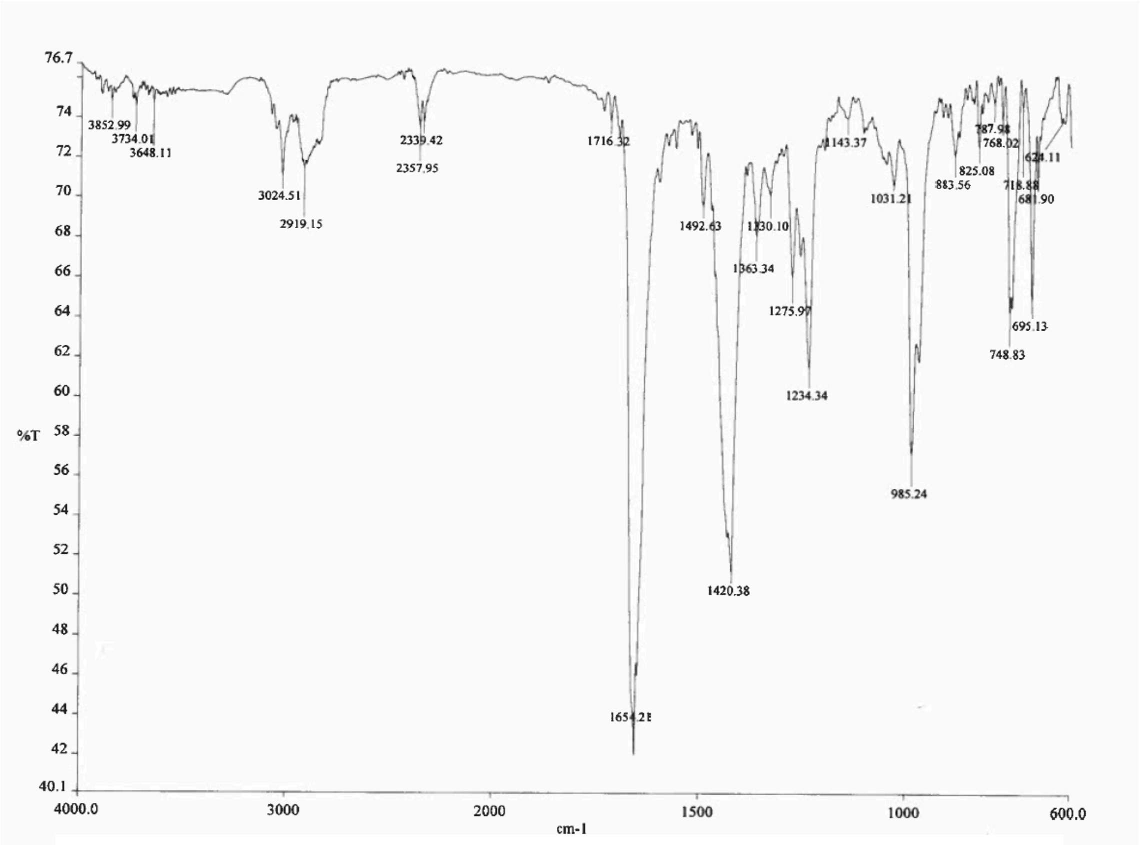

Infrared spectrum (Thin Film, NaCl) of compound **6f**.

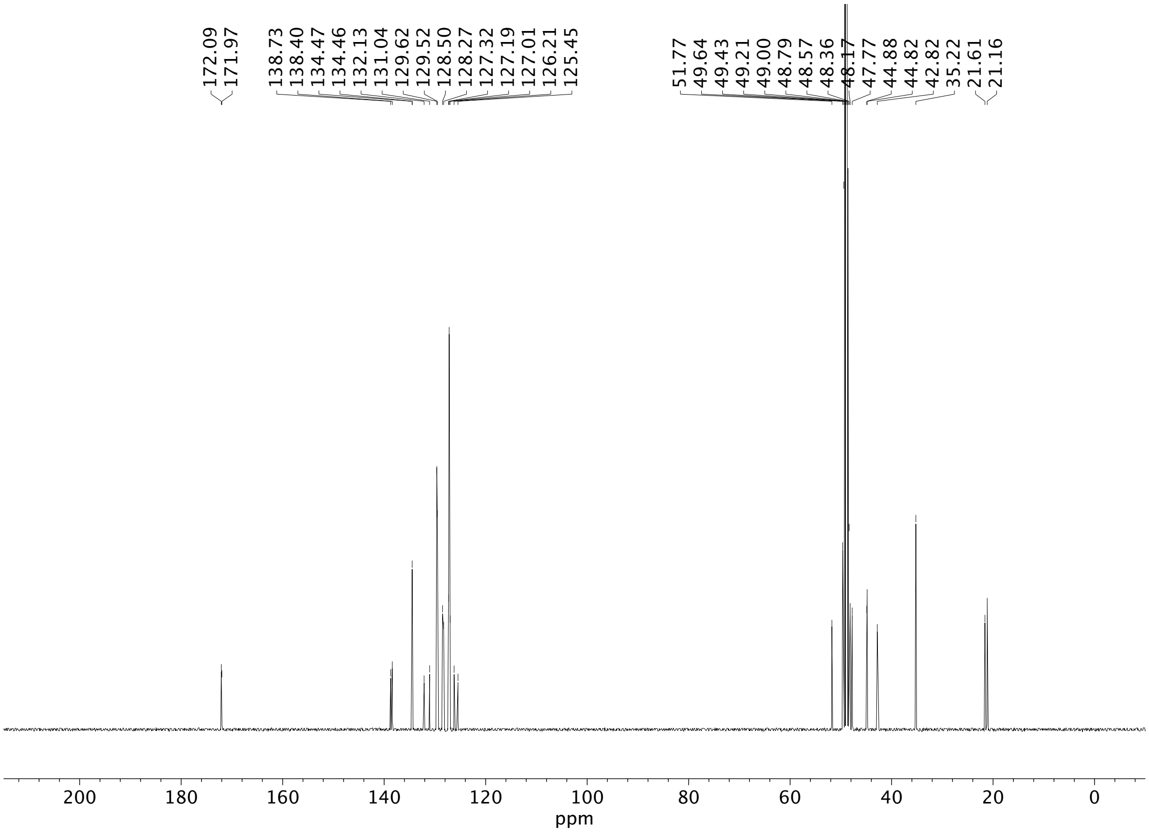

^{13}C NMR (100 MHz, CD_3OD) of compound **6f**.

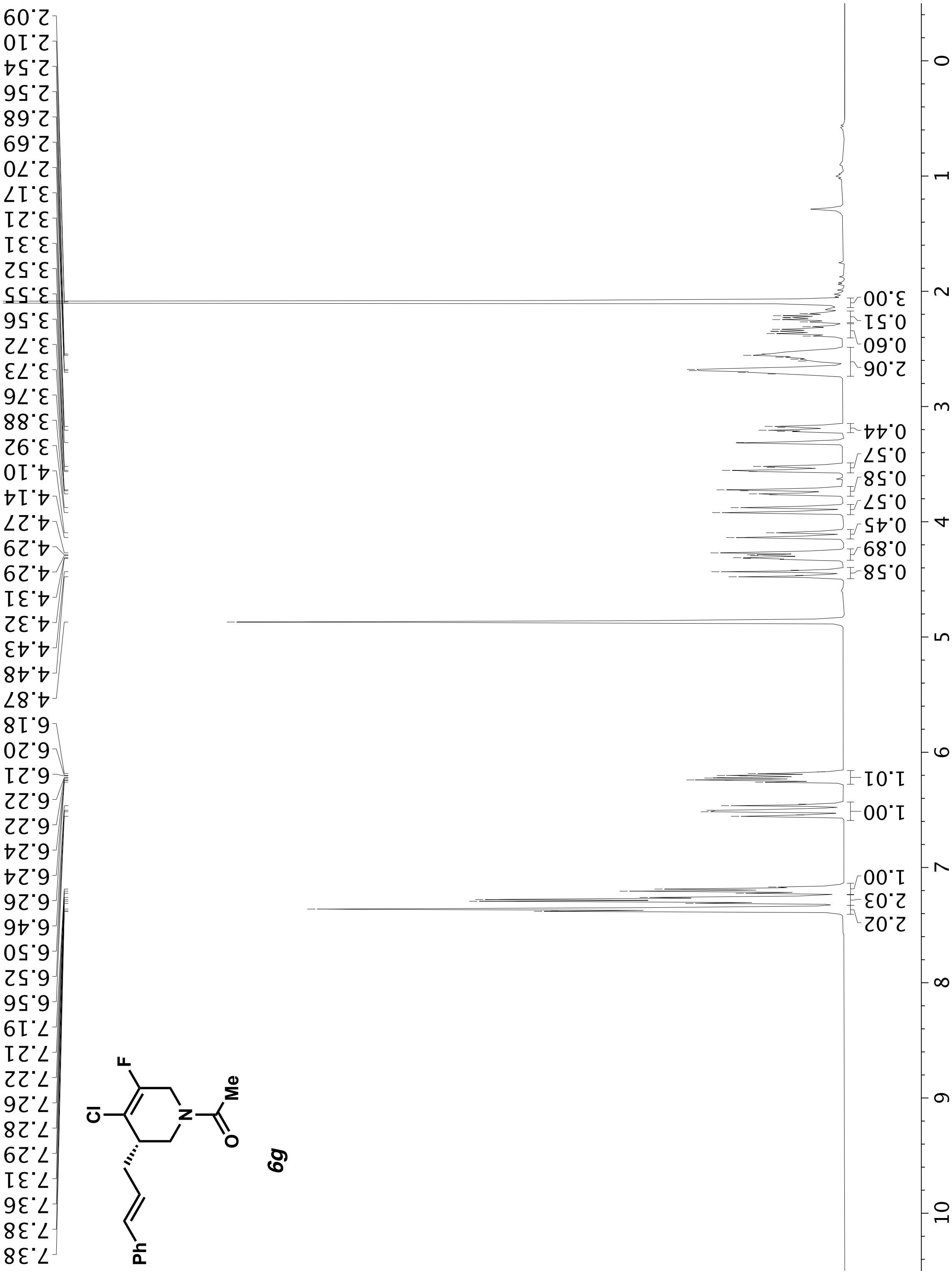

^{1}H NMR (400 MHz, CD_3OD) of compound **6g**.

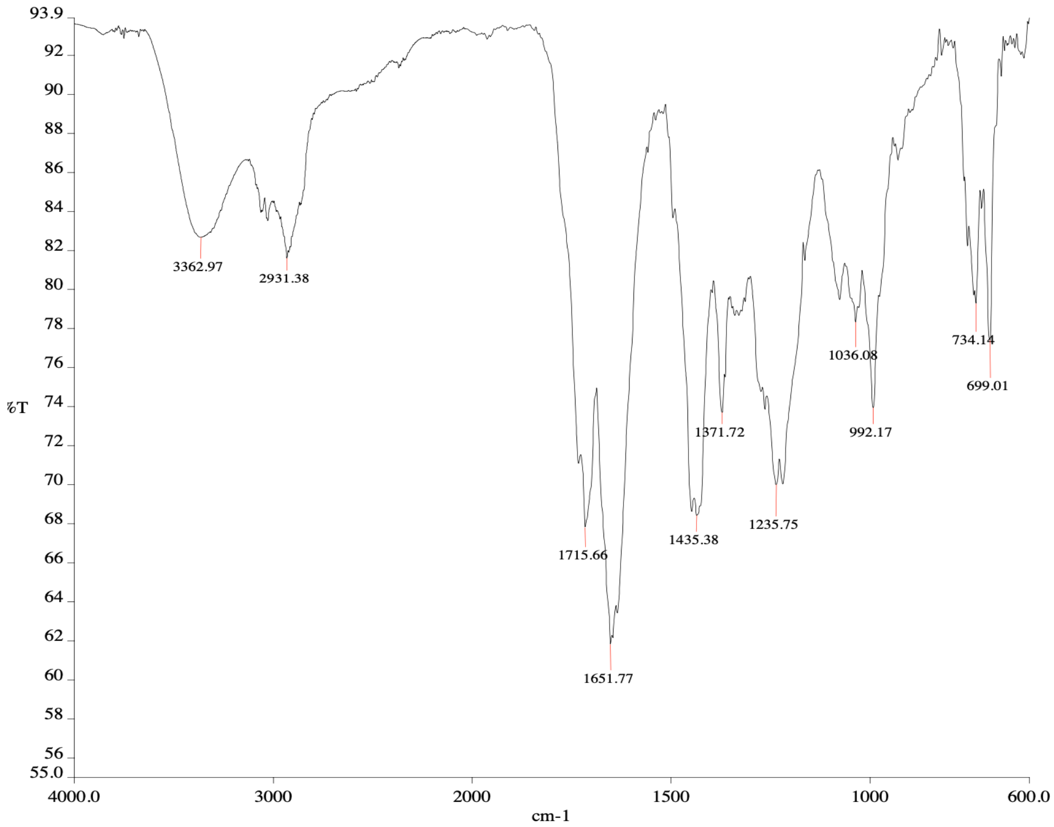

Infrared spectrum (Thin Film, NaCl) of compound **6g**.

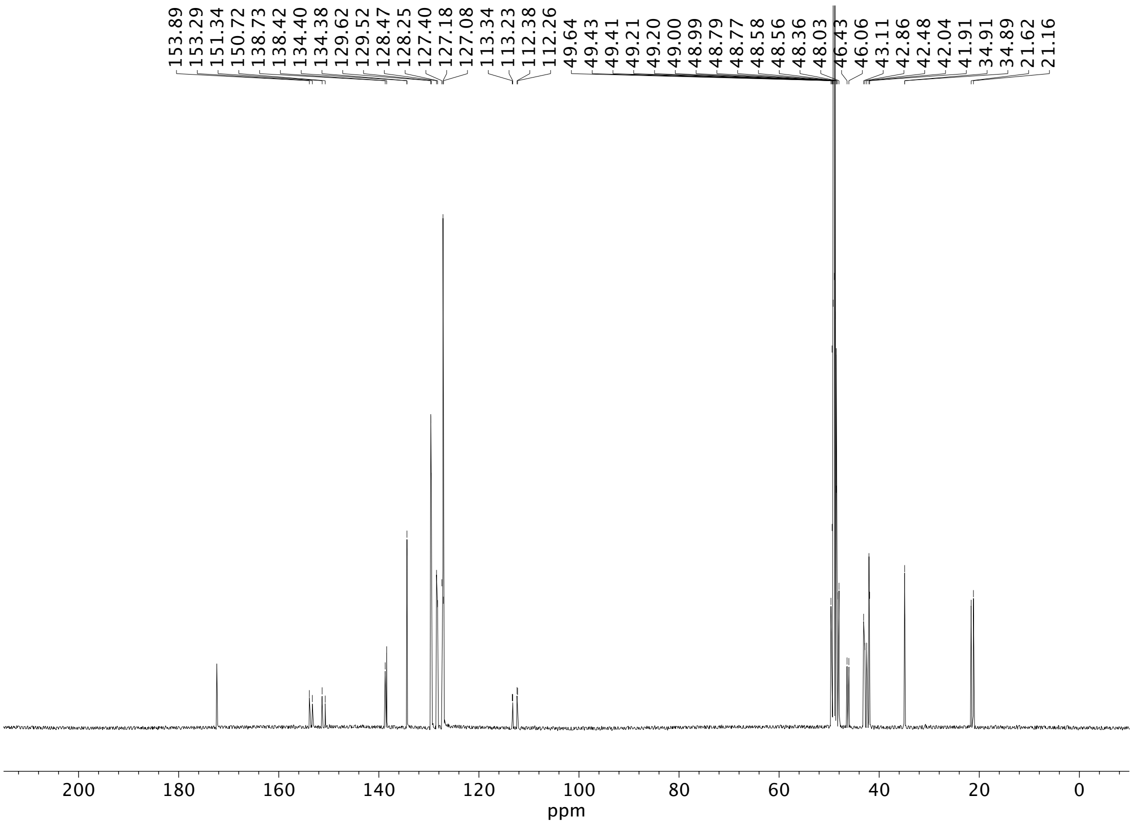

^{13}C NMR (100 MHz, CD_3OD) of compound **6g**.

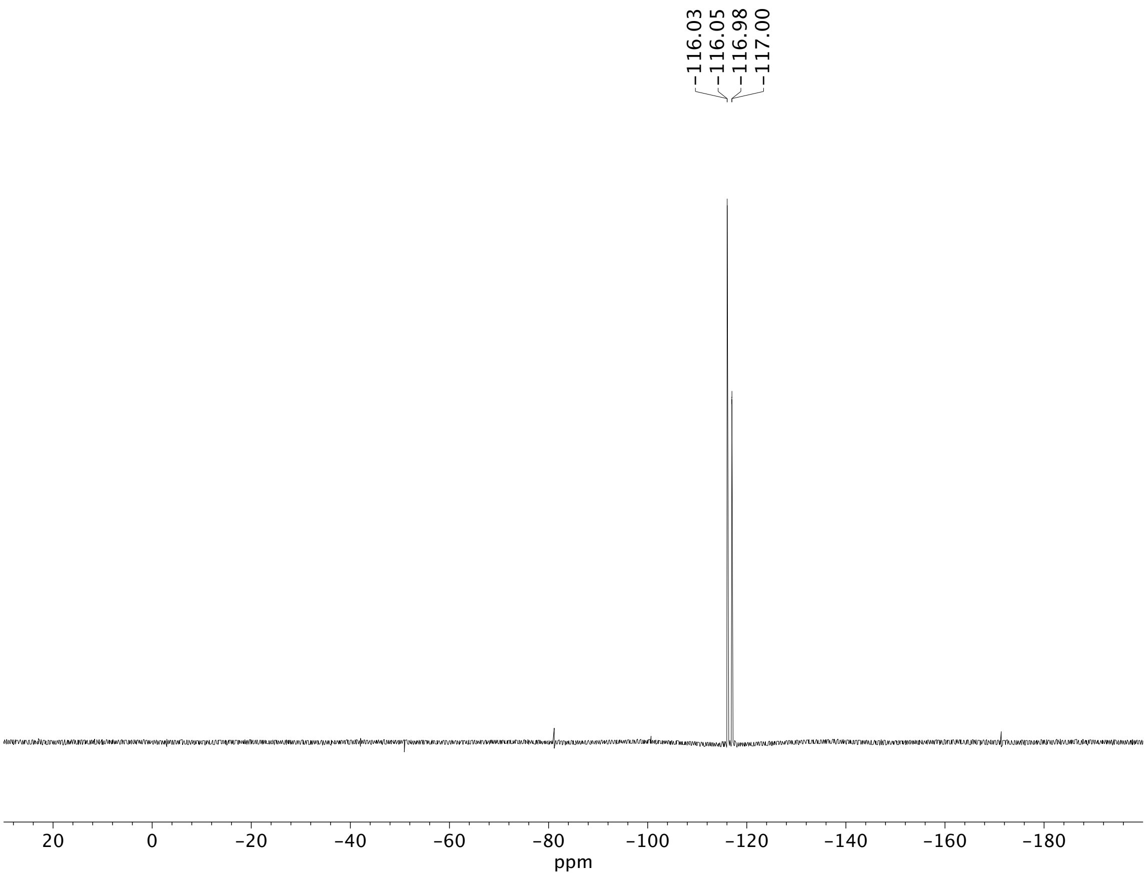

^{19}F NMR (282 MHz, CD_3OD) of compound **6g**.

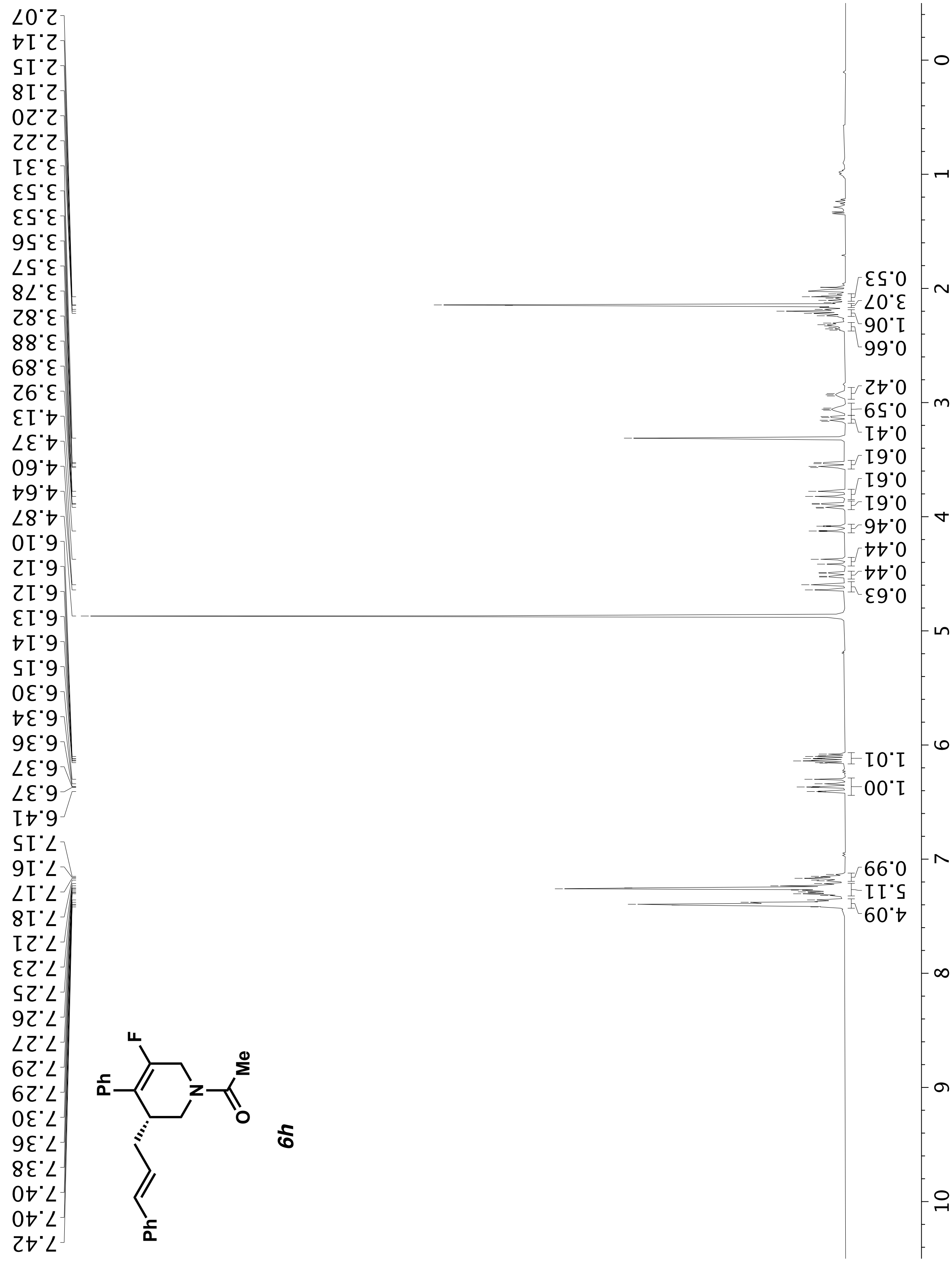

^{1}H NMR (400 MHz, CD_3OD) of compound **6h**.

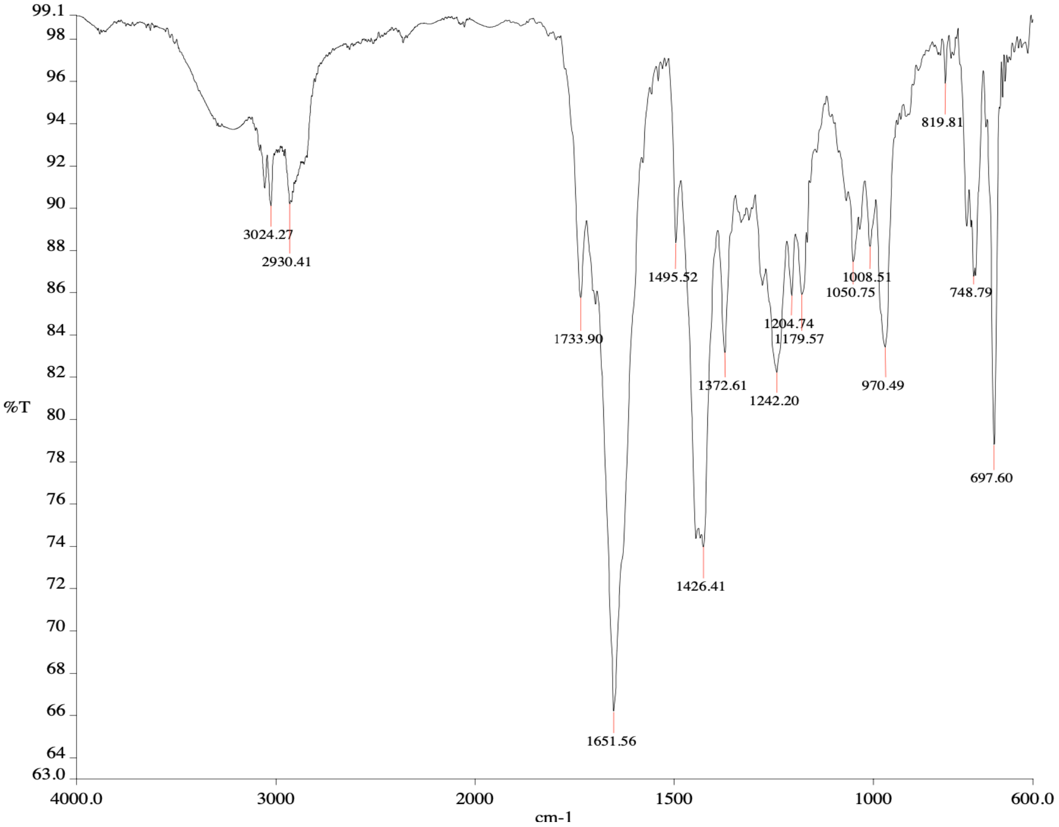

Infrared spectrum (Thin Film, NaCl) of compound **6h**.

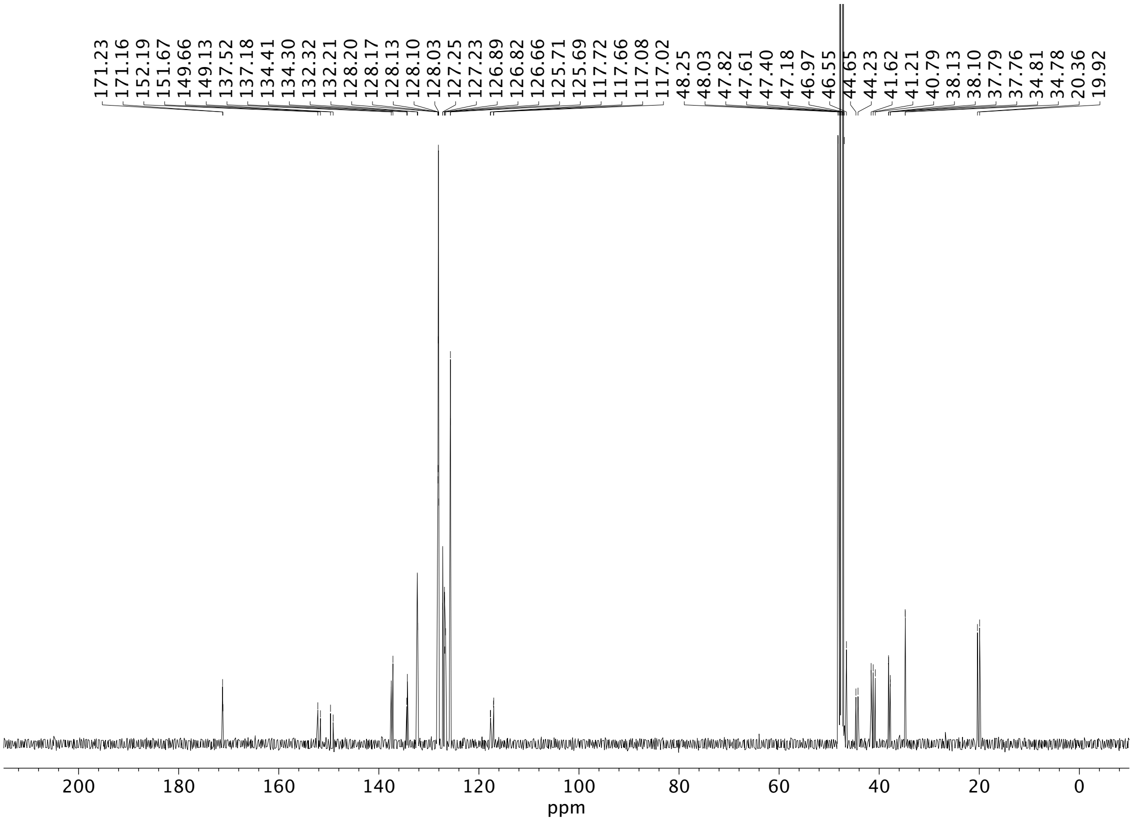

^{13}C NMR (100 MHz, CD_3OD) of compound **6h**.

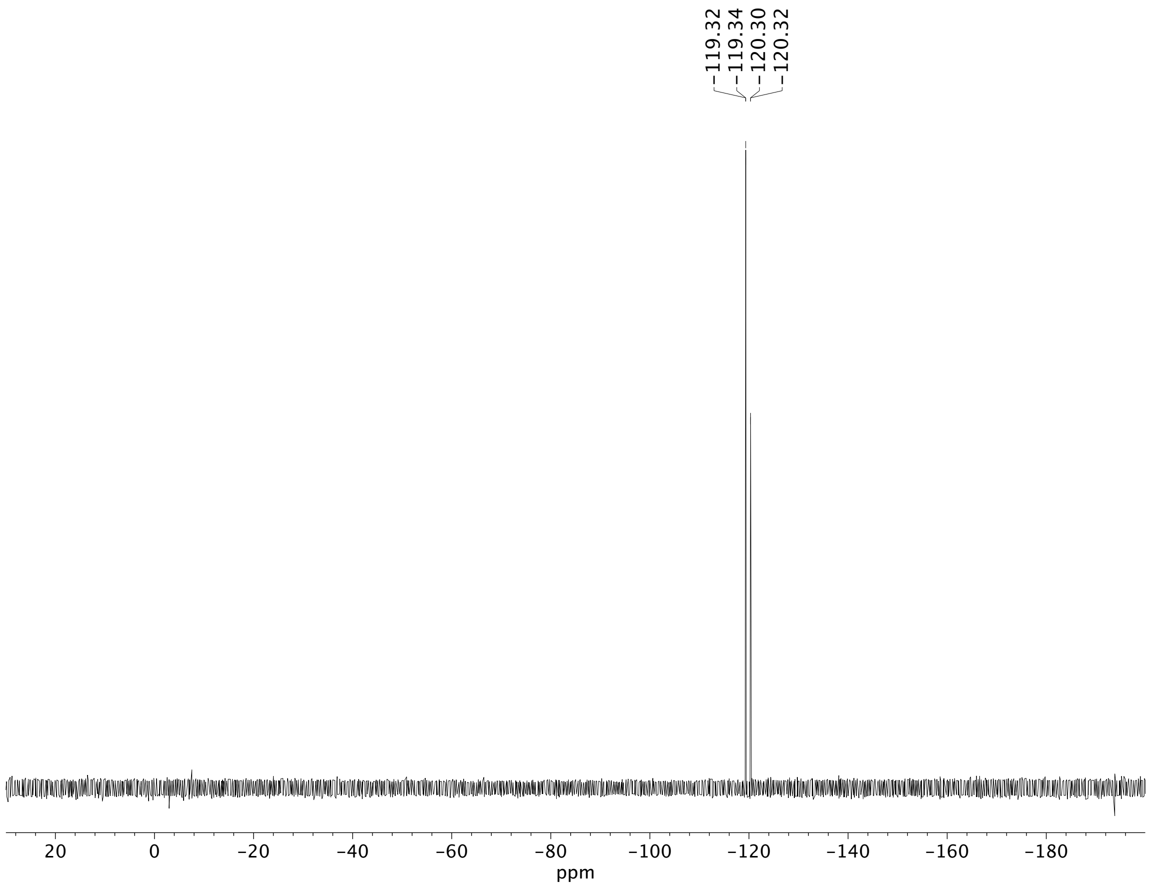

^{19}F NMR (282 MHz, CD_3OD) of compound **6h**.

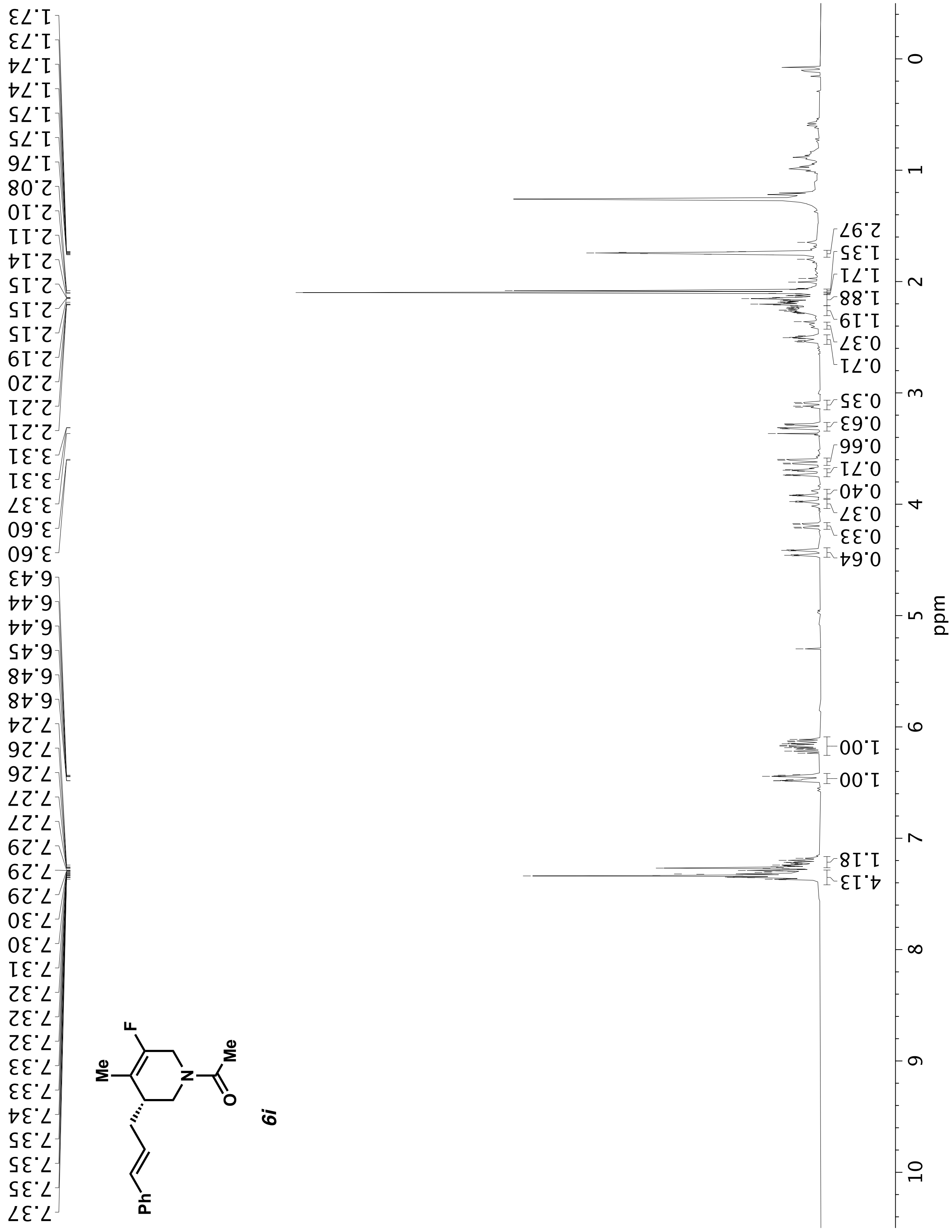

^{1}H NMR (400 MHz, $CDCl_3$) of compound **6i**.

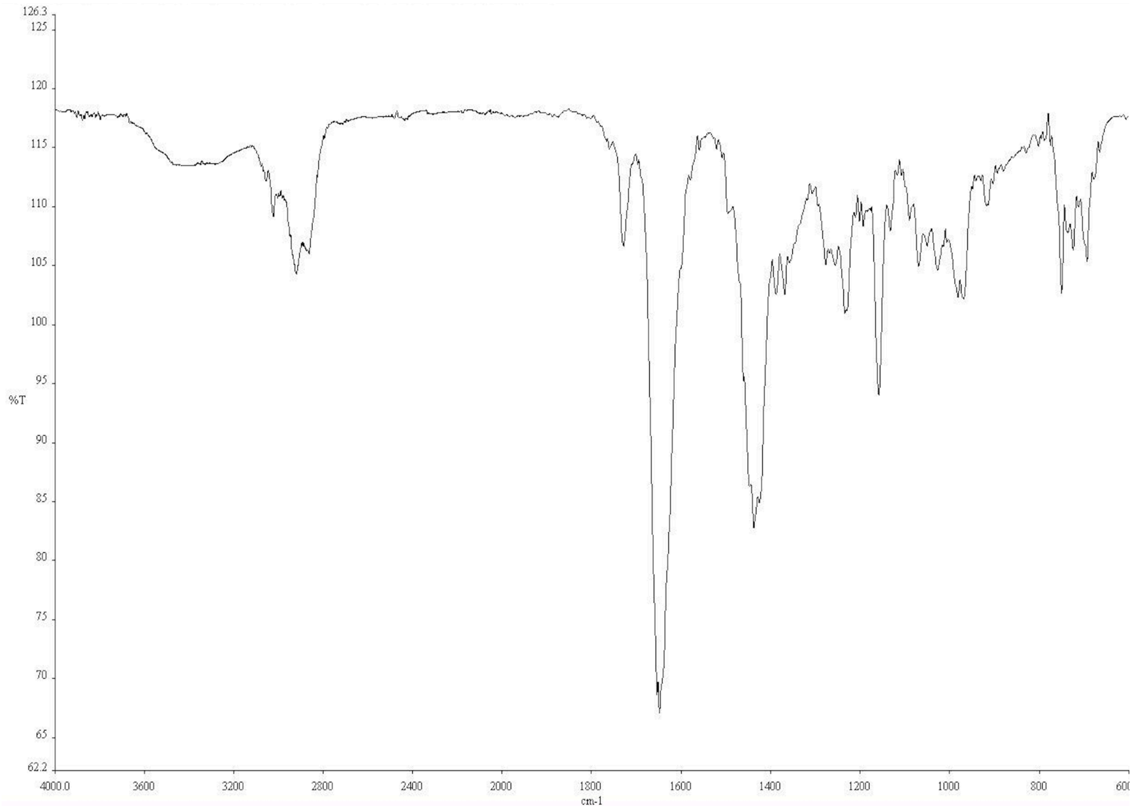

Infrared spectrum (Thin Film, NaCl) of compound **6i**.

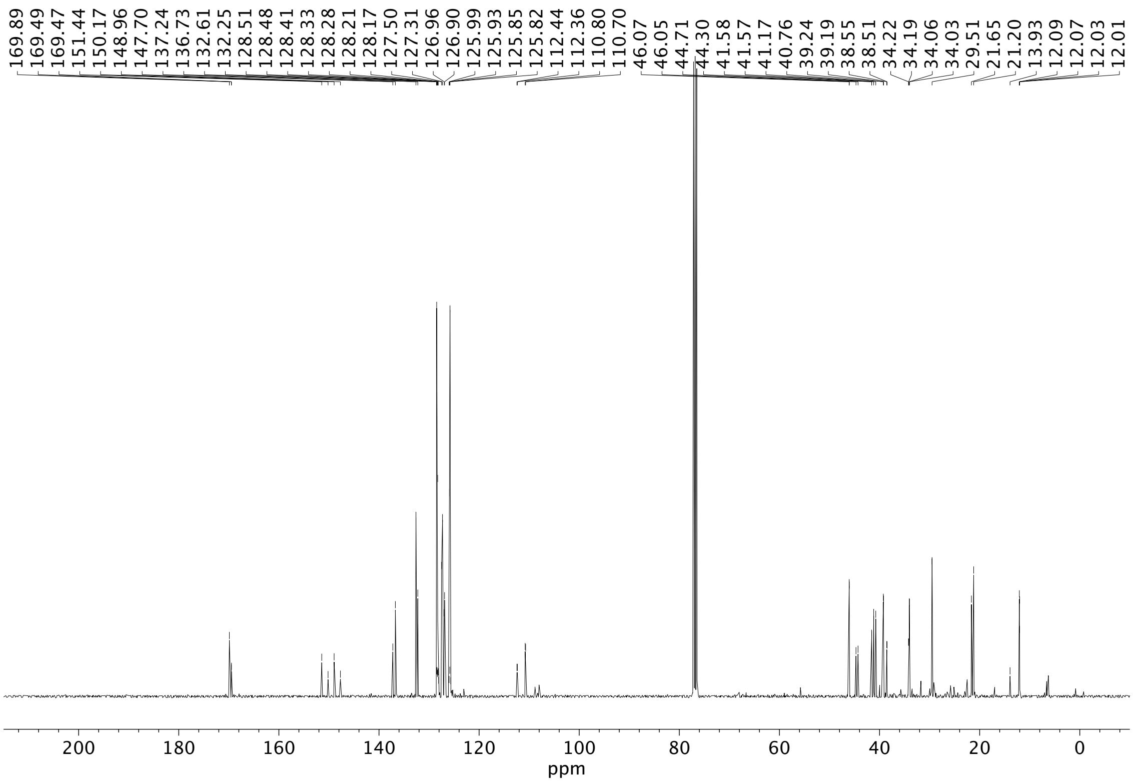

^{13}C NMR (100 MHz, $CDCl_3$) of compound **6i**.

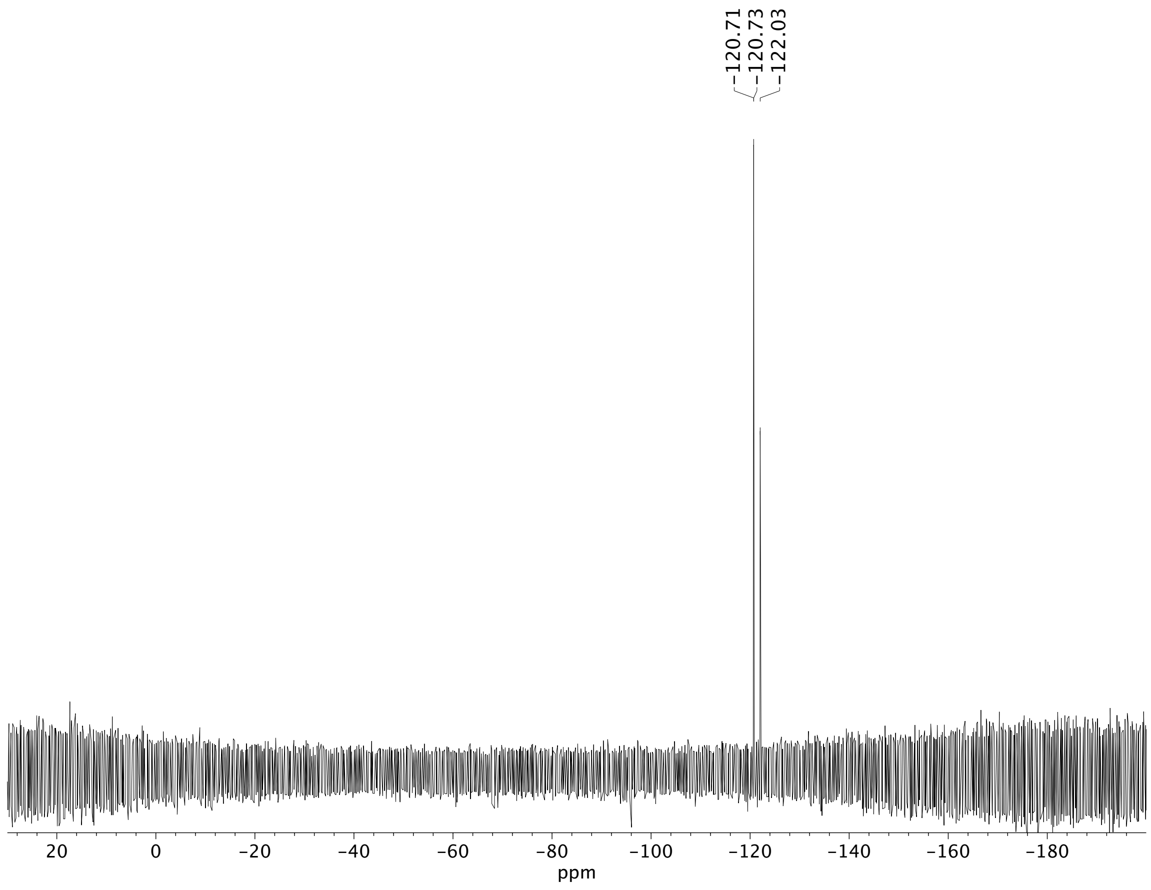

^{19}F NMR (282 MHz, $CDCl_3$) of compound **6i**.

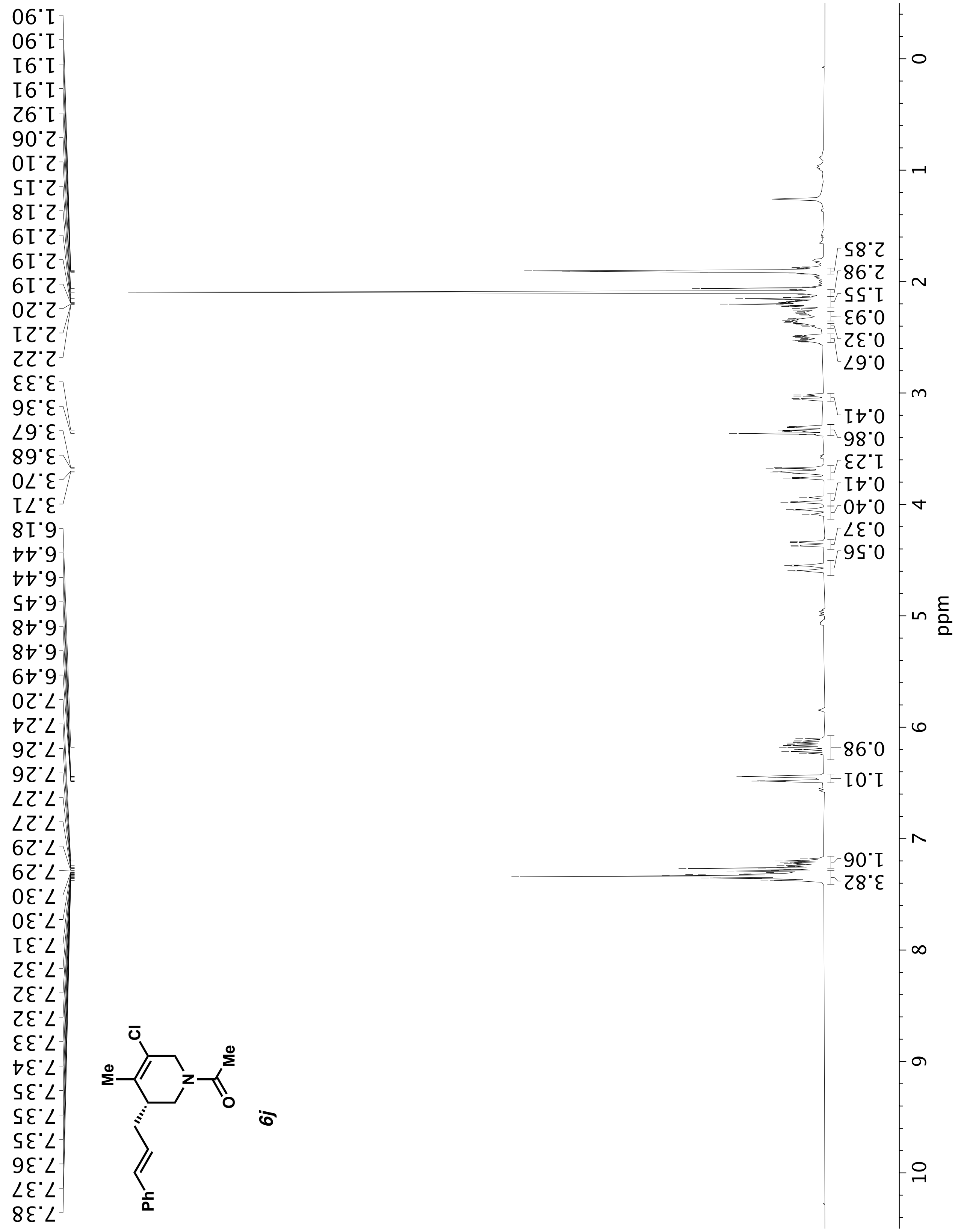

6j

^{1}H NMR (400 MHz, $CDCl_3$) of compound **6j**

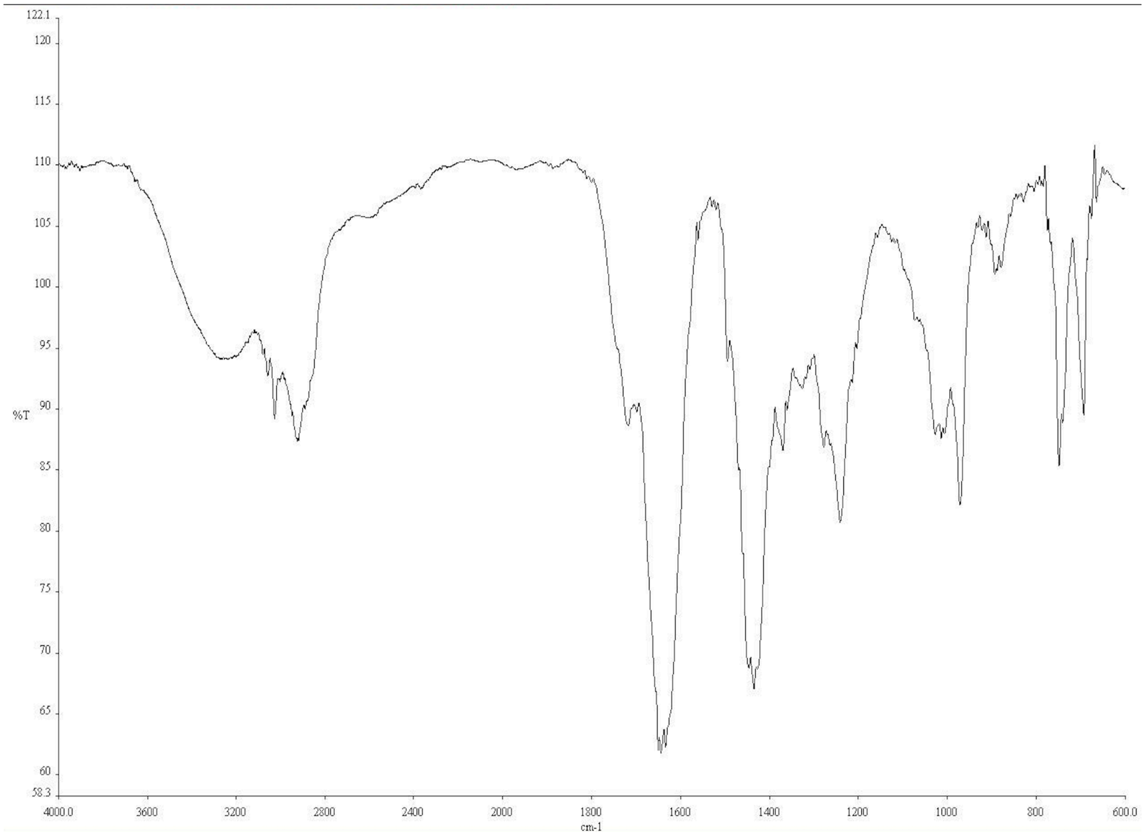

Infrared spectrum (Thin Film, NaCl) of compound **6j**.

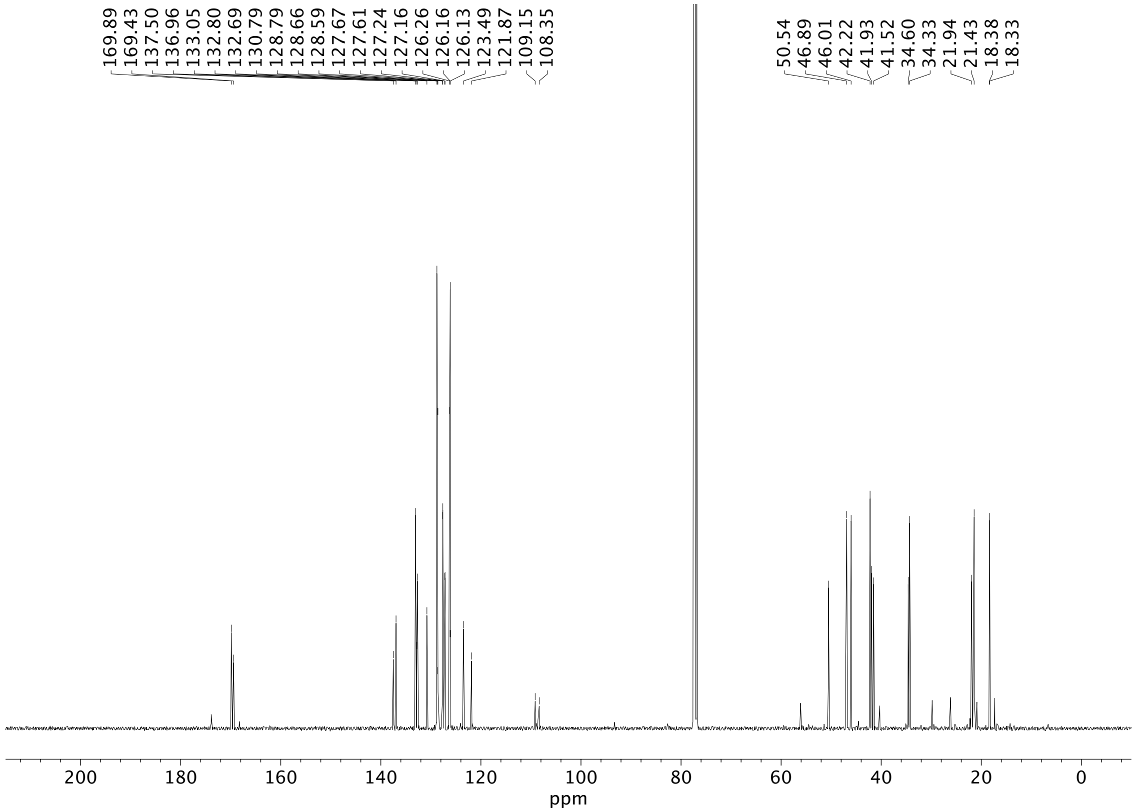

^{13}C NMR (100 MHz, $CDCl_3$) of compound **6j**.

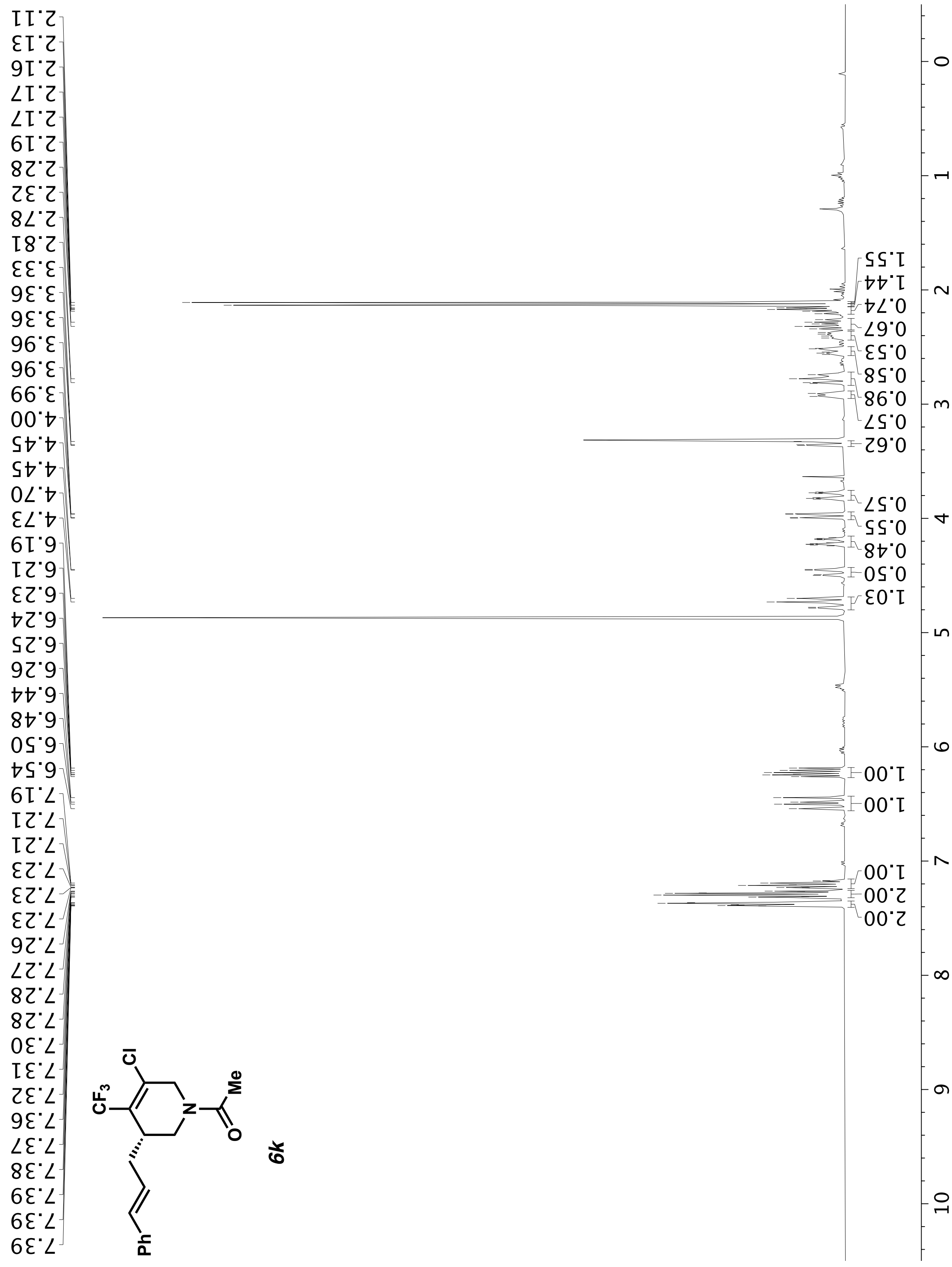

^{1}H NMR (400 MHz, CD_3OD) of compound **6k**.

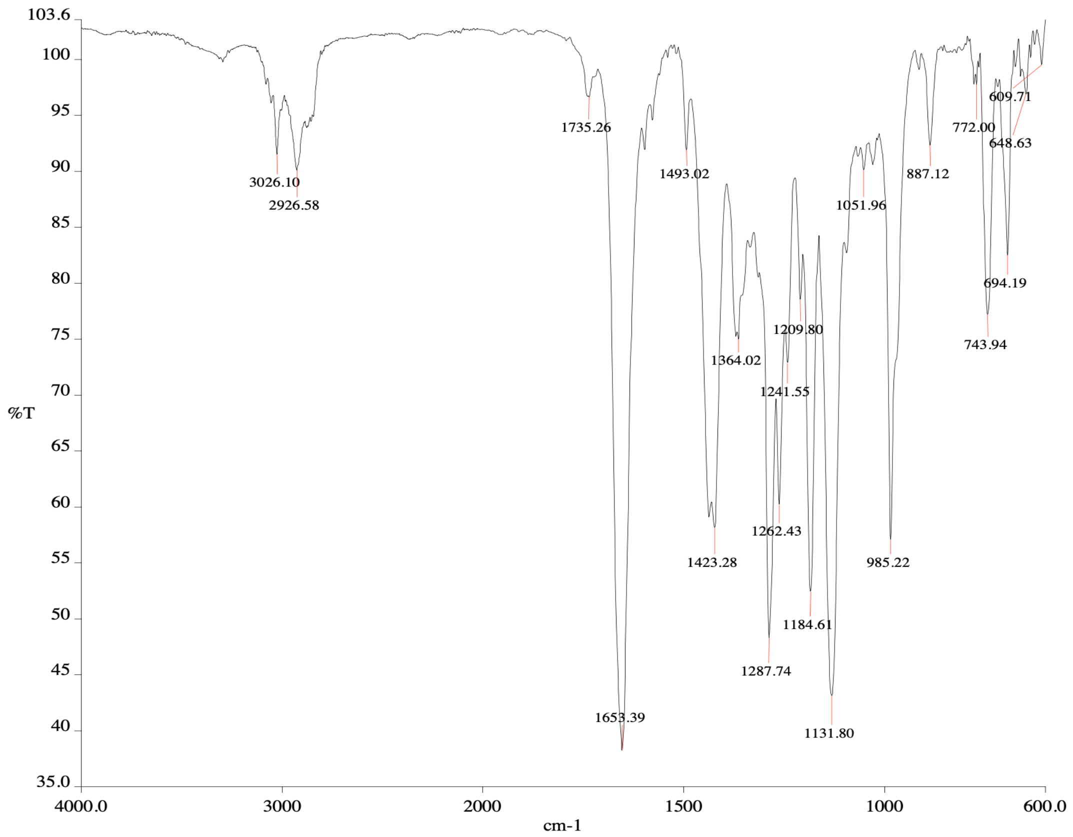

Infrared spectrum (Thin Film, NaCl) of compound **6k**.

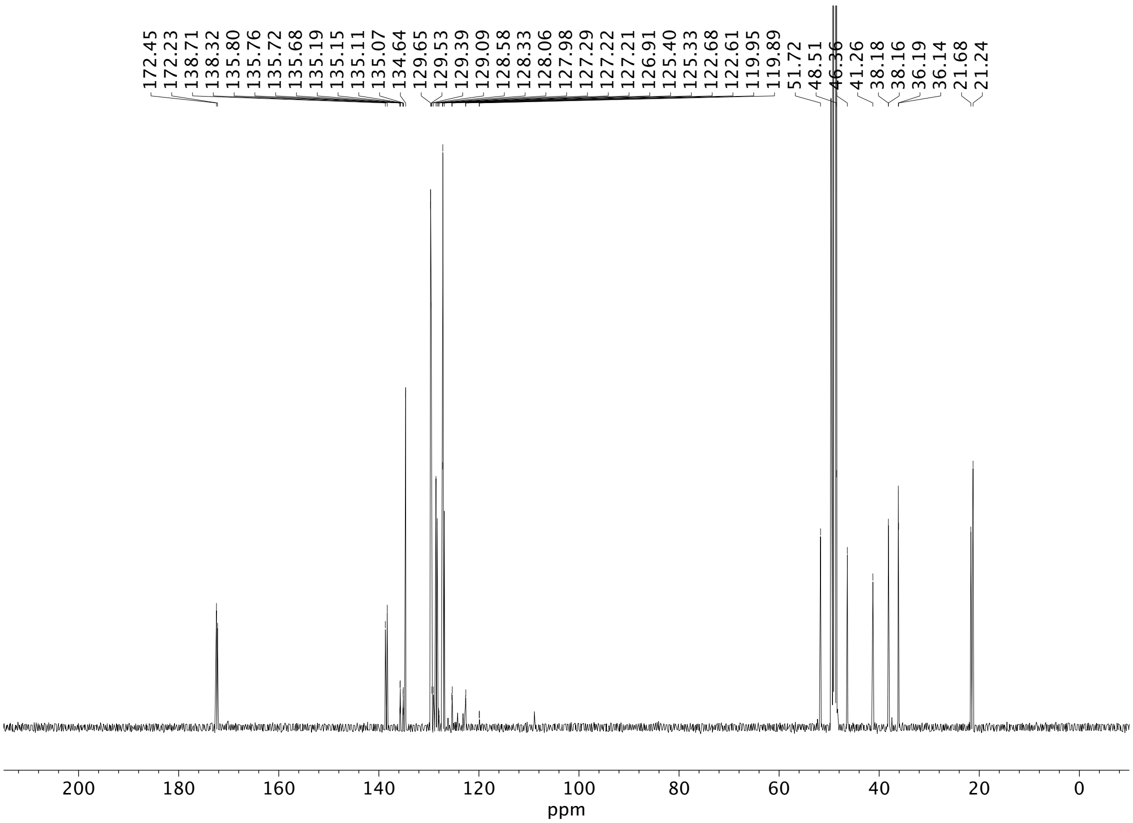

^{13}C NMR (100 MHz, CD_3OD) of compound **6k**.

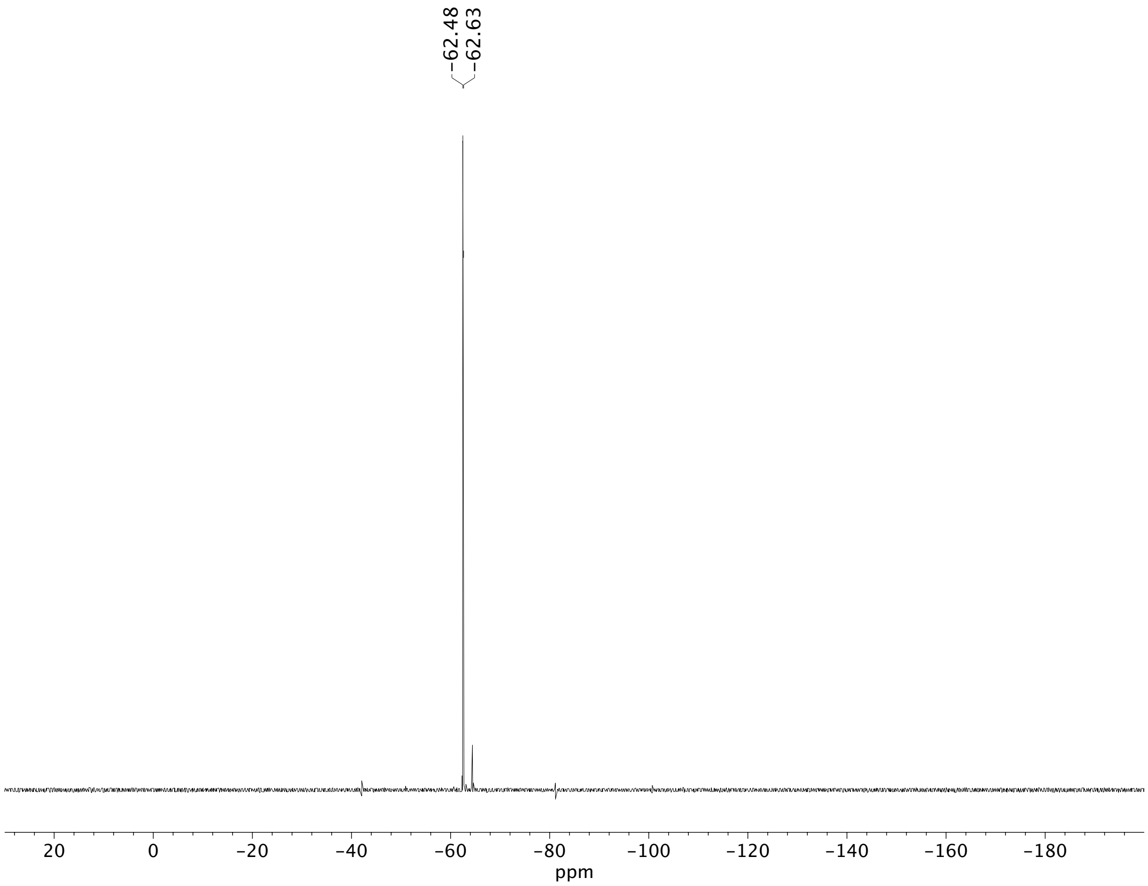

^{19}F NMR (282 MHz, CD_3OD) of compound **6k**.

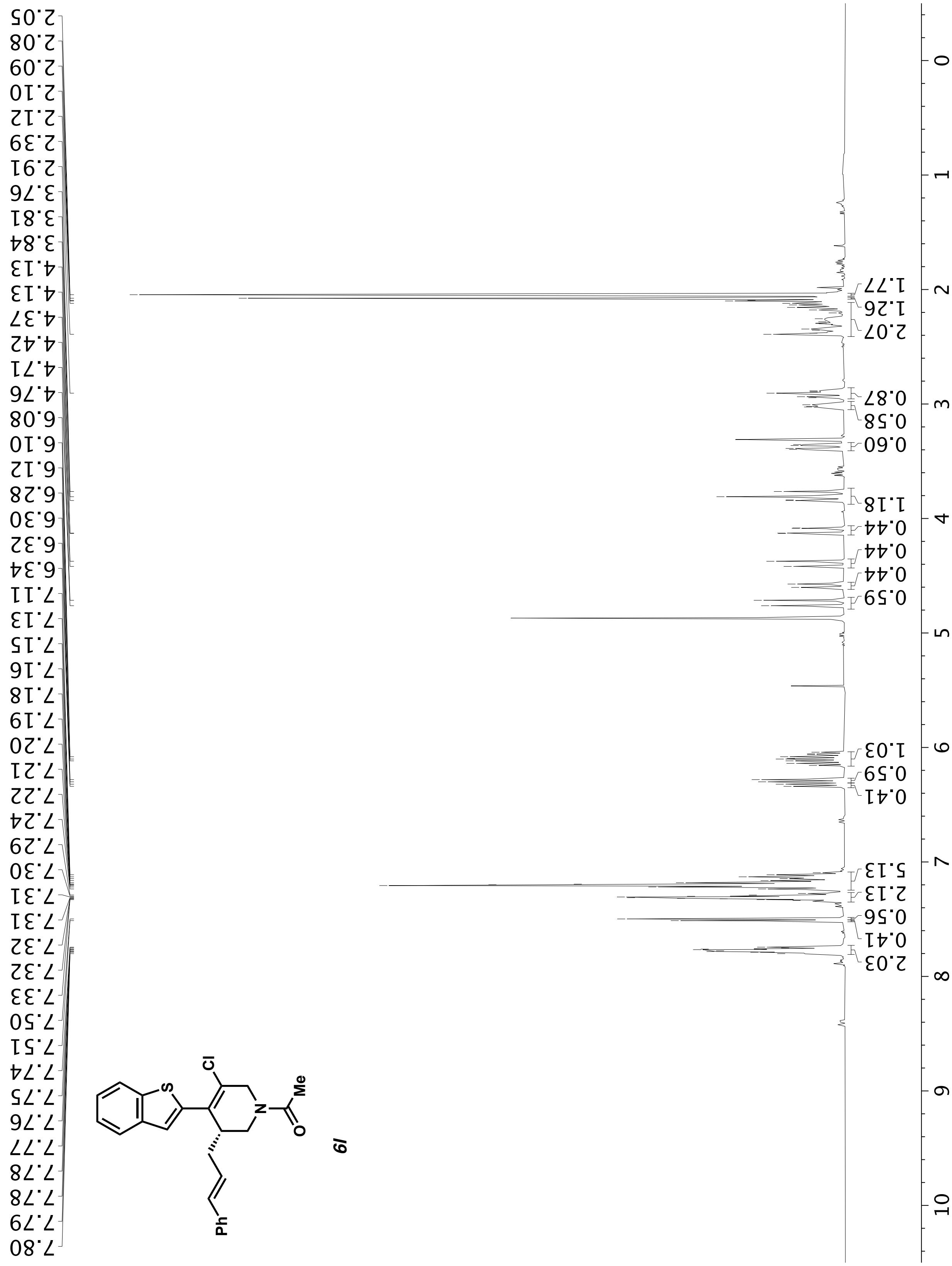

^{1}H NMR (400 MHz, CD_3OD) of compound **6l**.

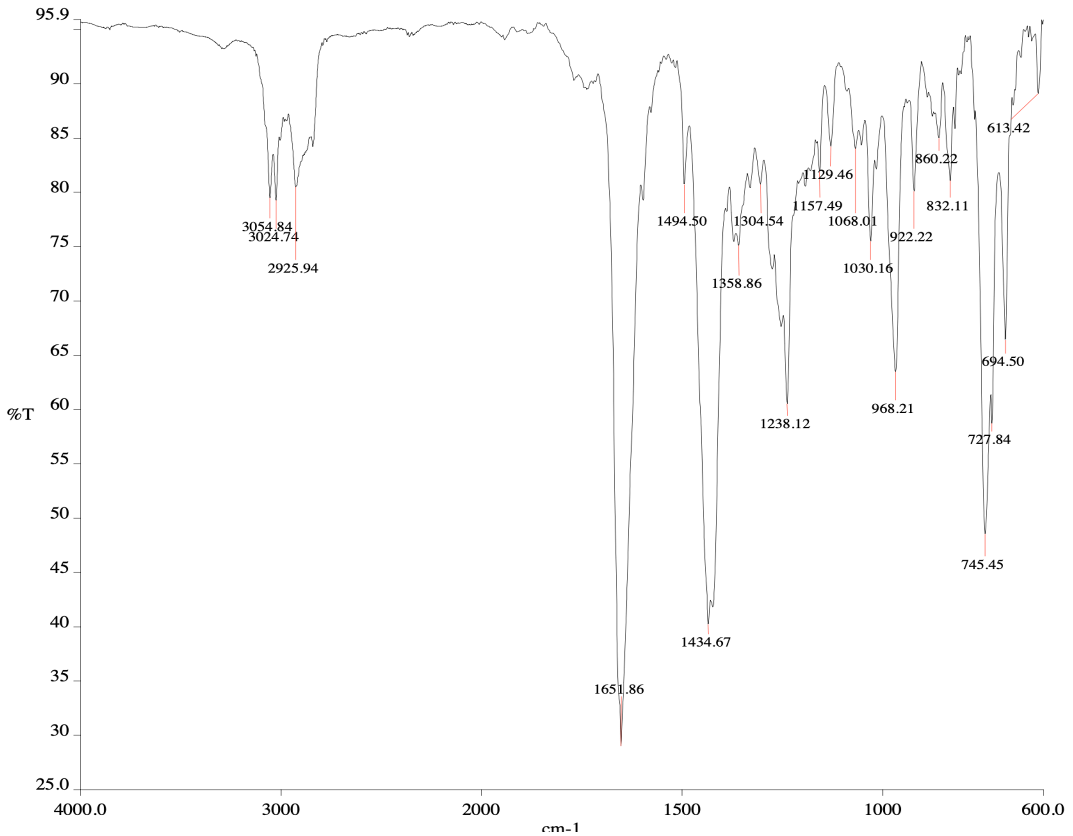

Infrared spectrum (Thin Film, NaCl) of compound **6l**.

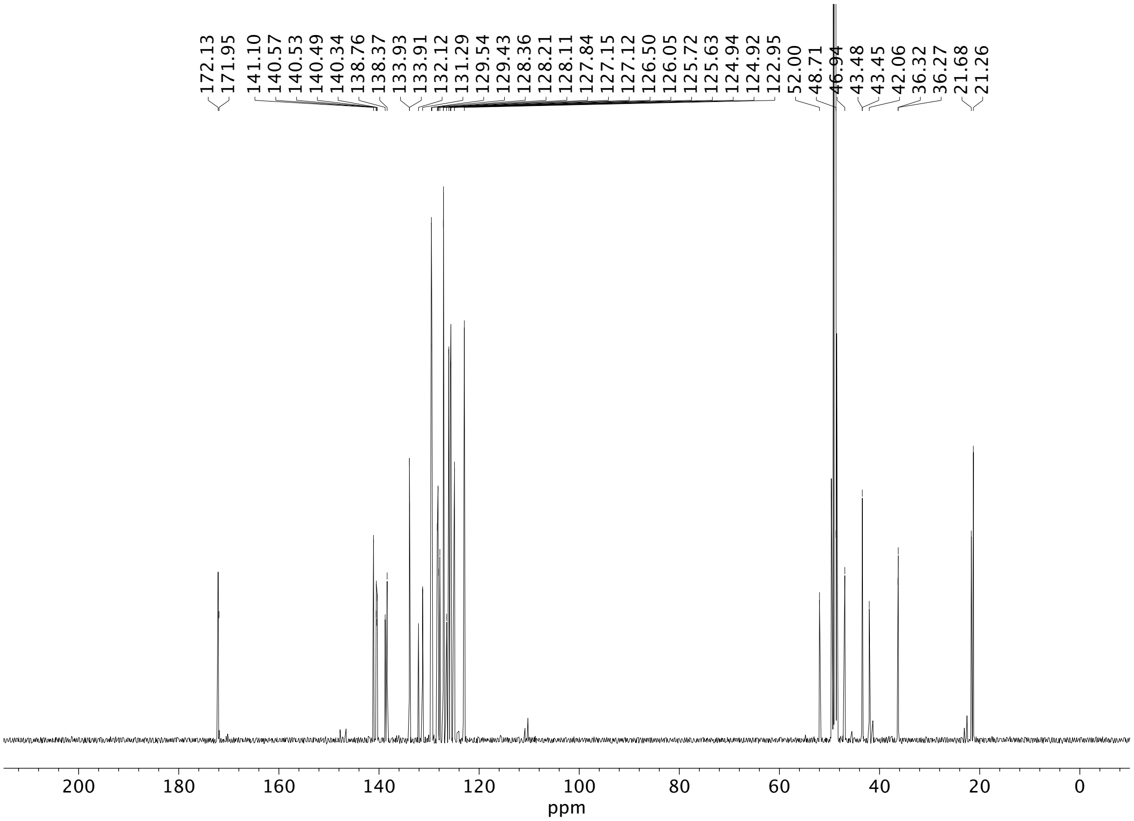

^{13}C NMR (100 MHz, CD_3OD) of compound **6l**.

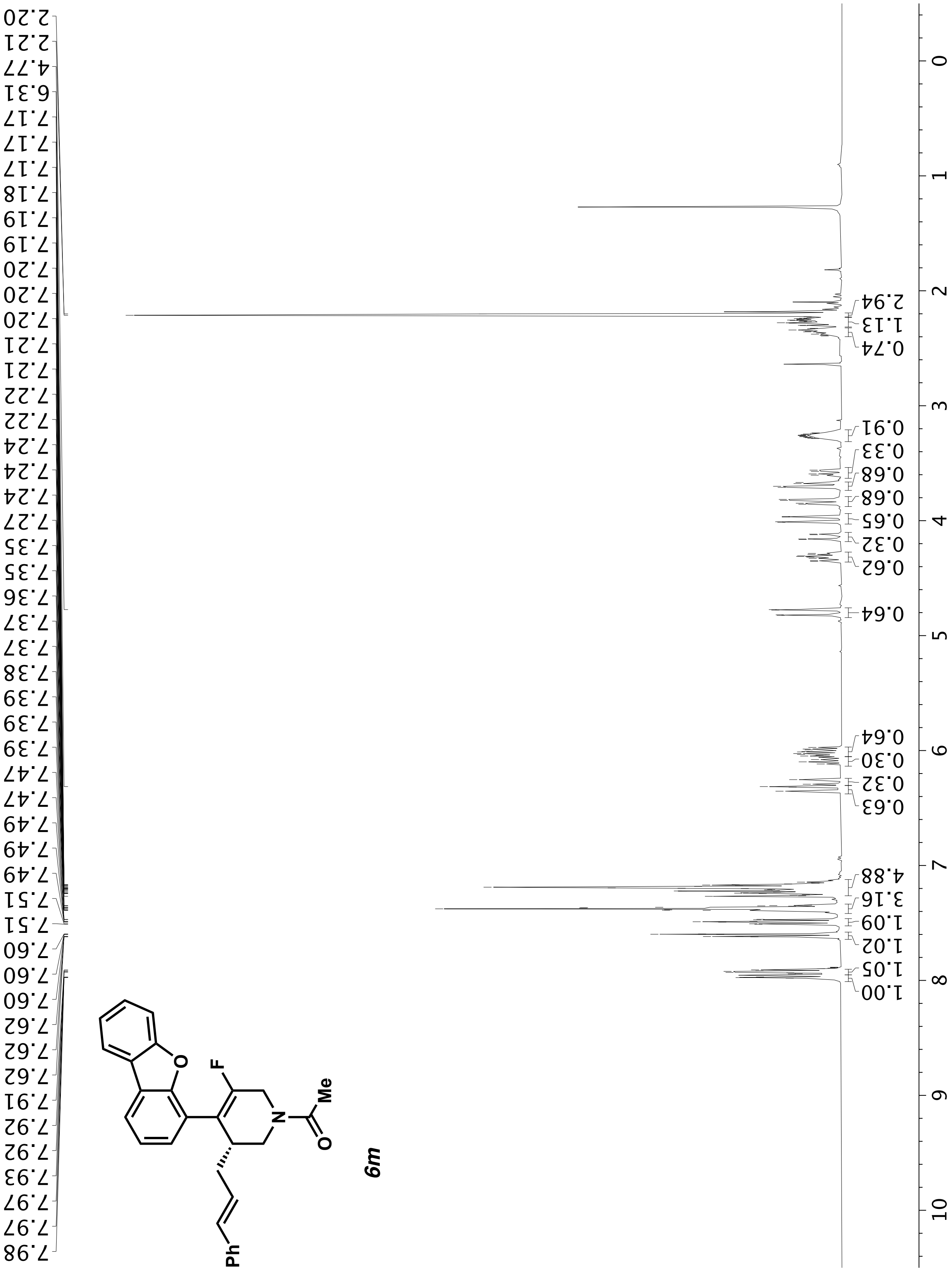

^{1}H NMR (400 MHz, $CDCl_3$) of compound **6m**.

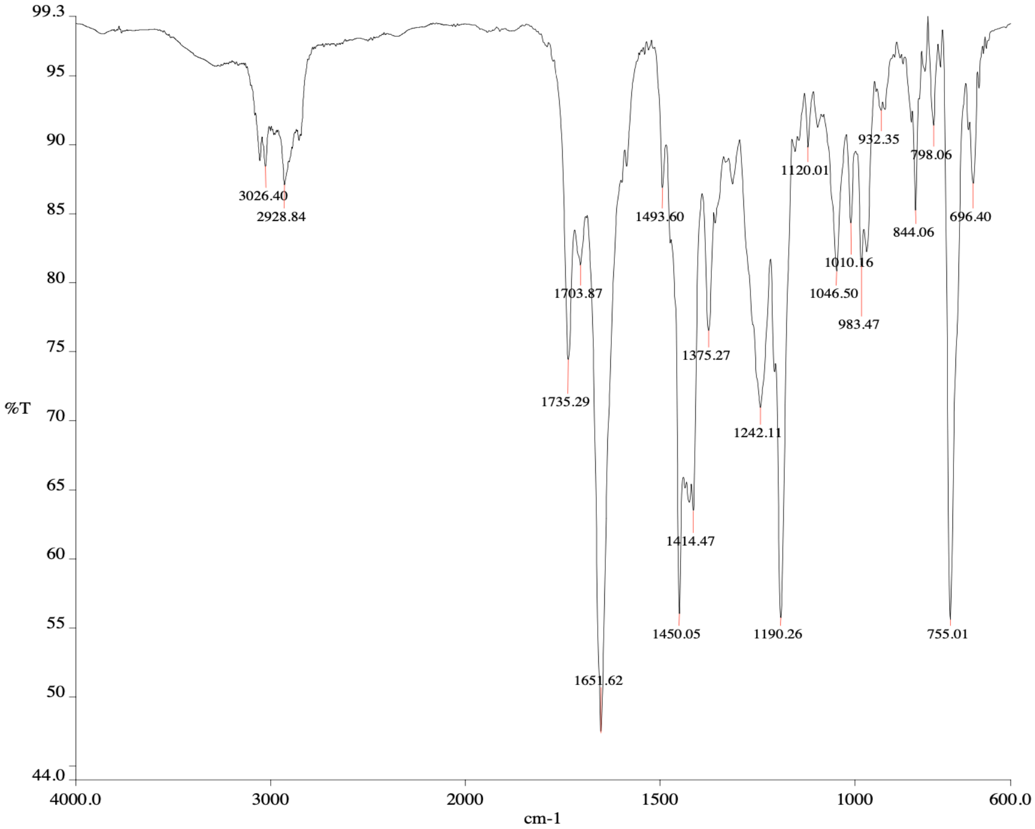

Infrared spectrum (Thin Film, NaCl) of compound **6m**.

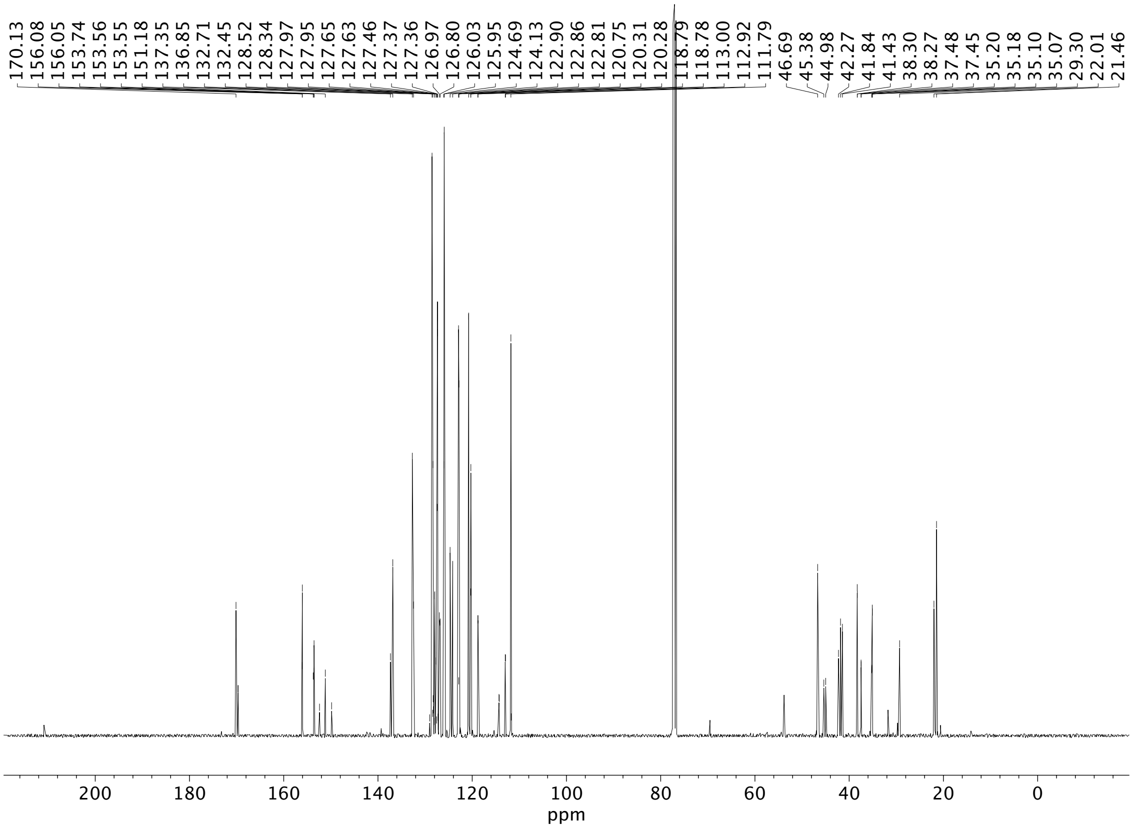

^{13}C NMR (100 MHz, $CDCl_3$) of compound **6m**.

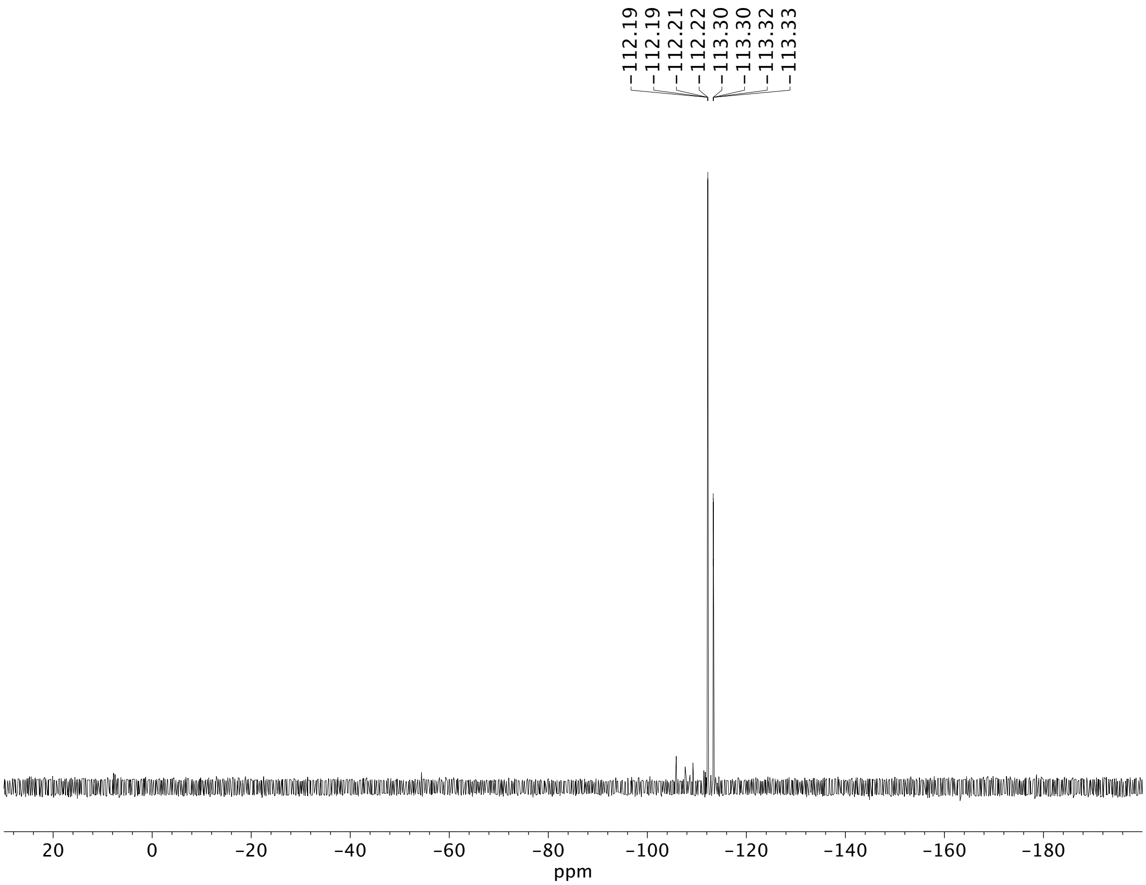

^{19}F NMR (282 MHz, $CDCl_3$) of compound **6m.**

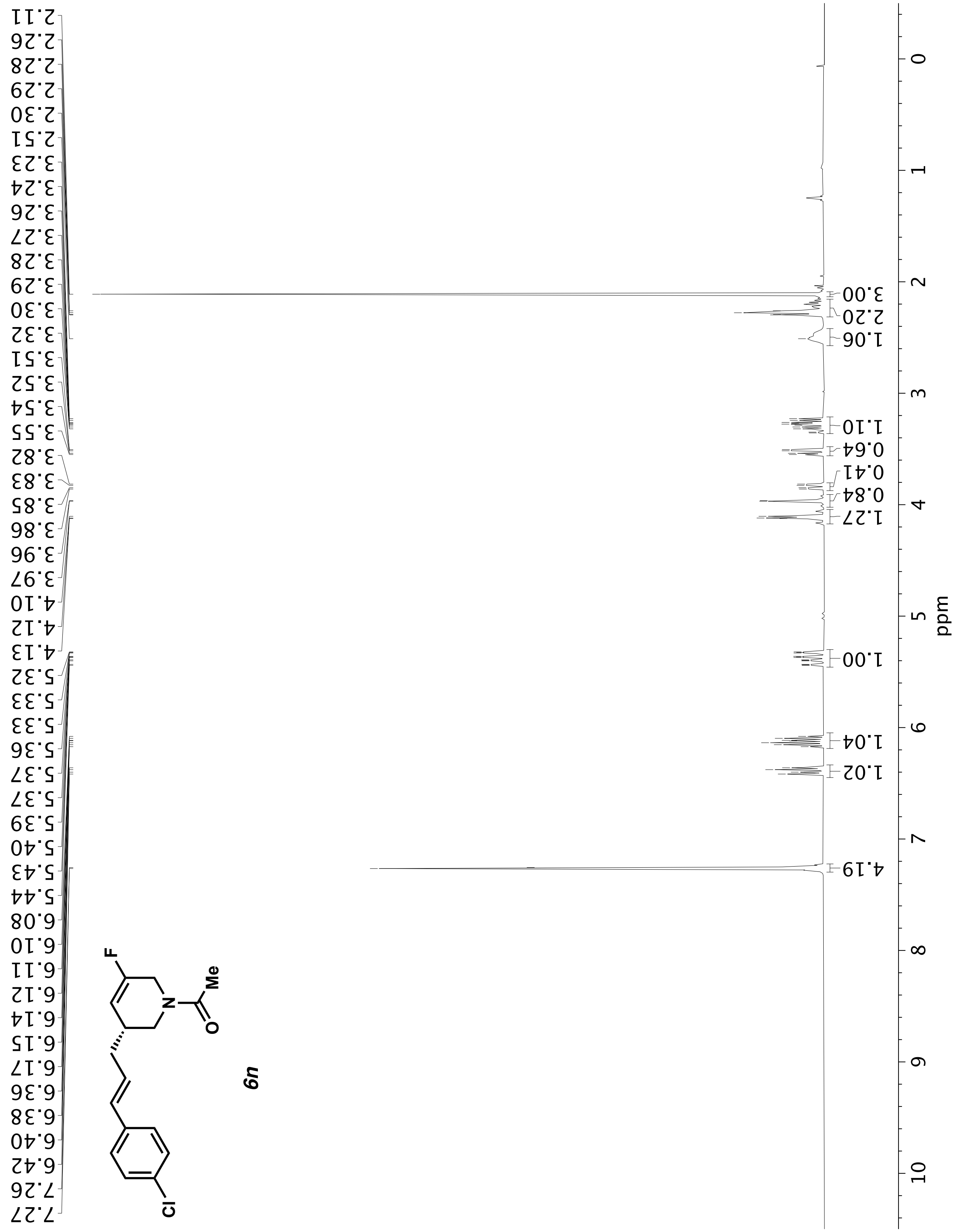

^{1}H NMR (400 MHz, $CDCl_3$) of compound **6n**.

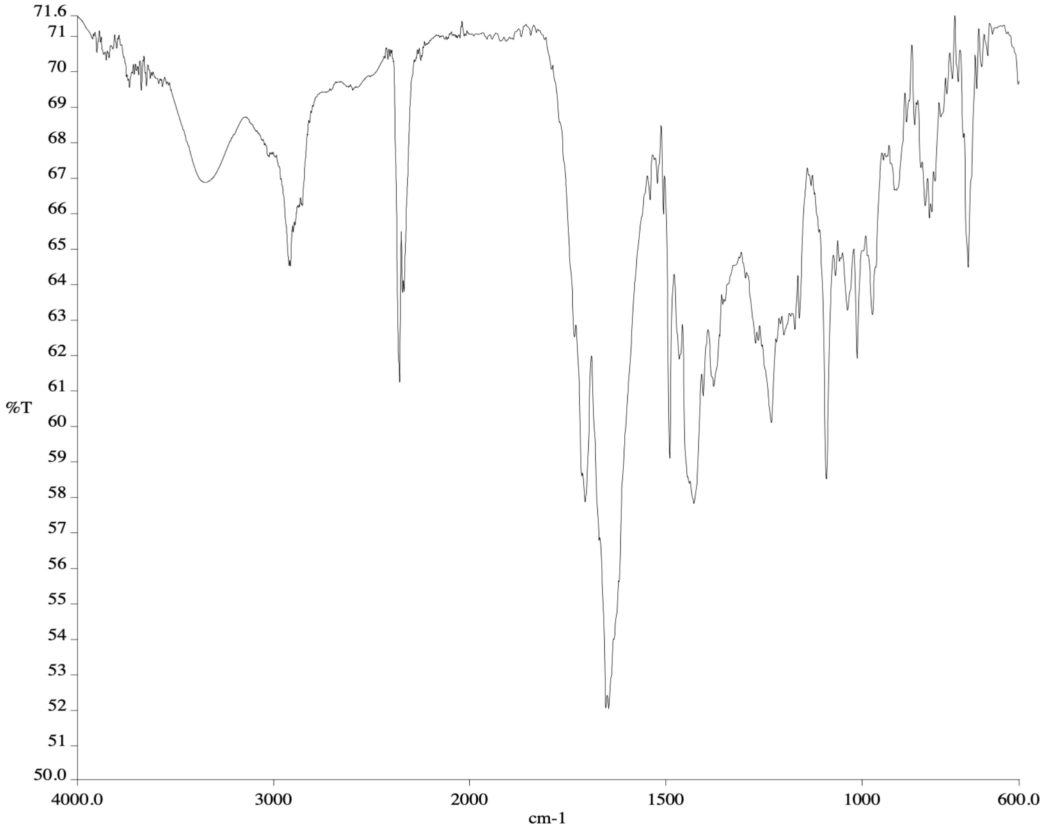

Infrared spectrum (Thin Film, NaCl) of compound **6n**.

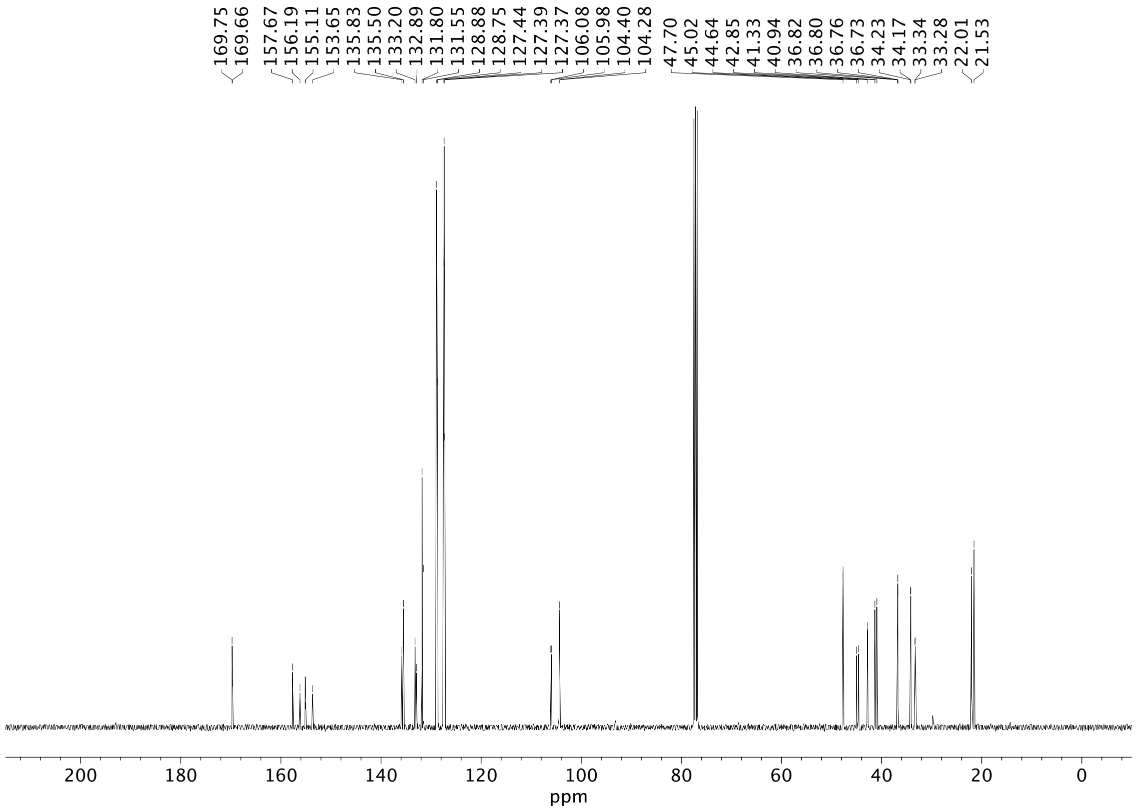

^{13}C NMR (100 MHz, $CDCl_3$) of compound **6n**.

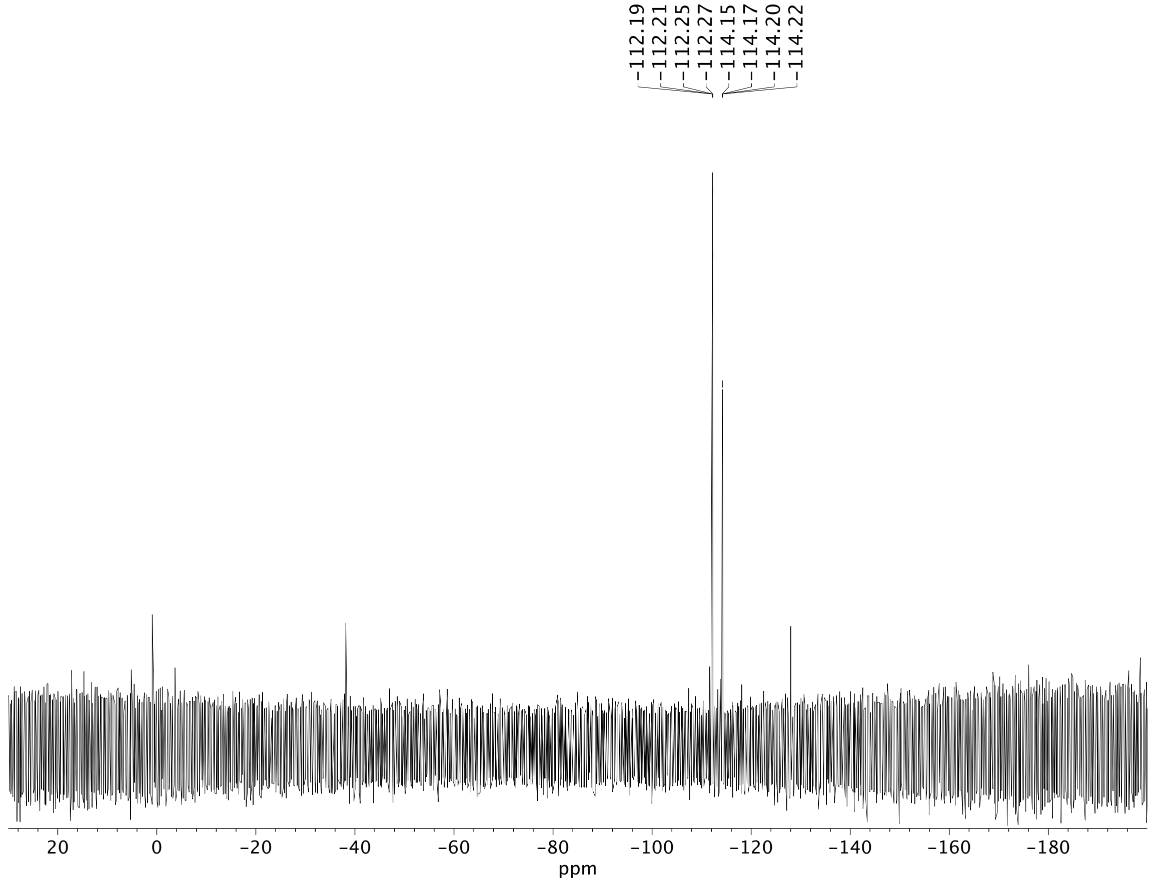

^{19}F NMR (282 MHz, $CDCl_3$) of compound **6n**.

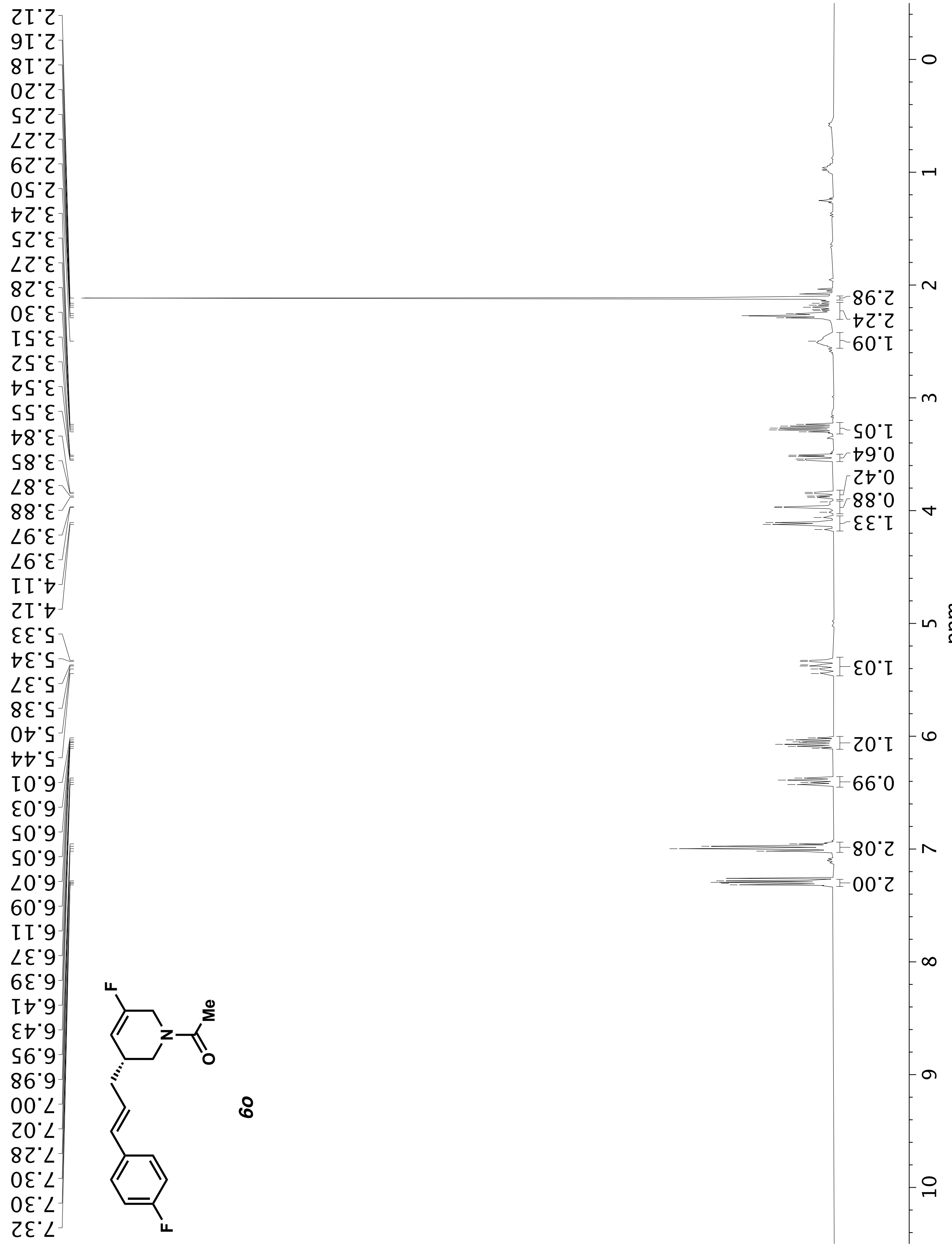

^{1}H NMR (400 MHz, $CDCl_3$) of compound **6o**.

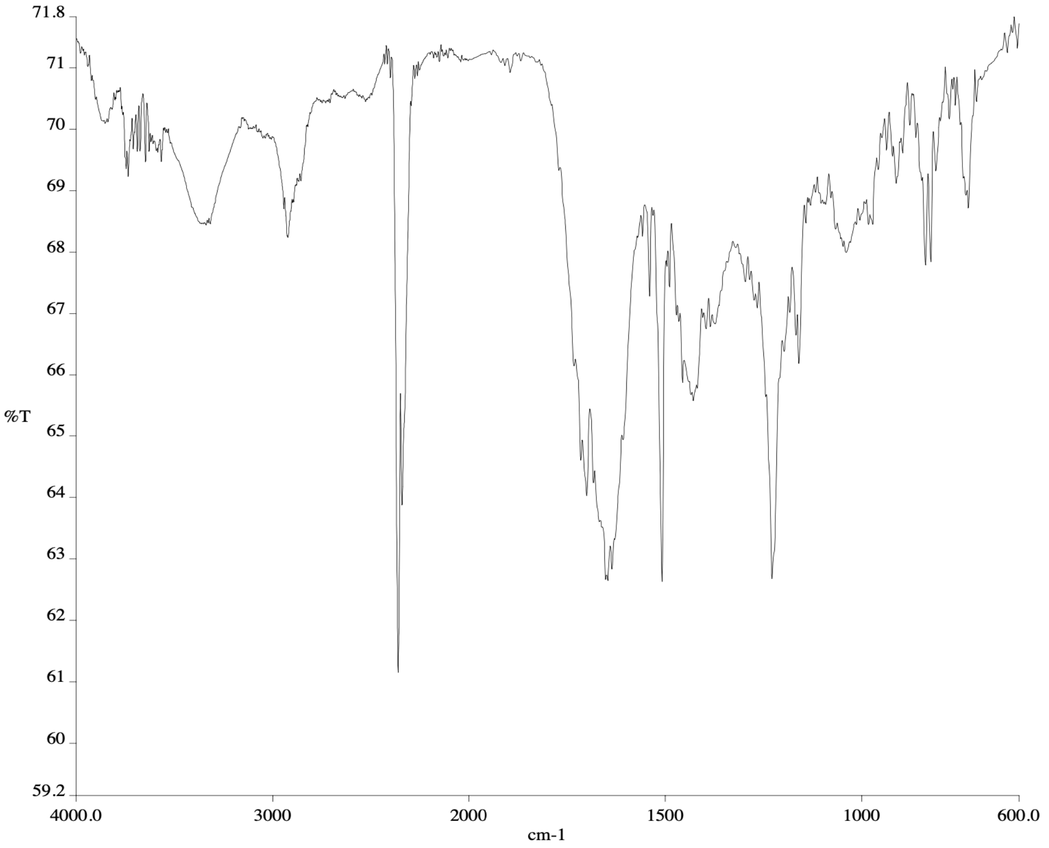

Infrared spectrum (Thin Film, NaCl) of compound **6o.**

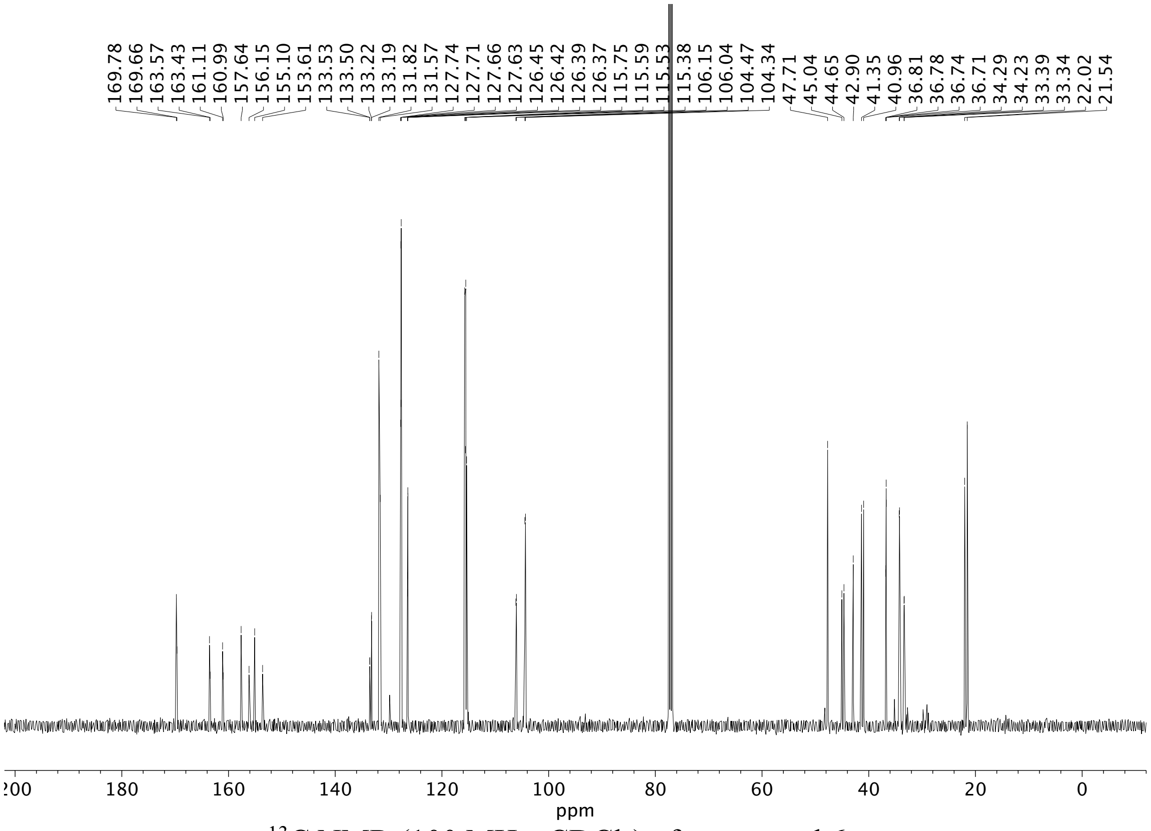

^{13}C NMR (100 MHz, $CDCl_3$) of compound **6o**.

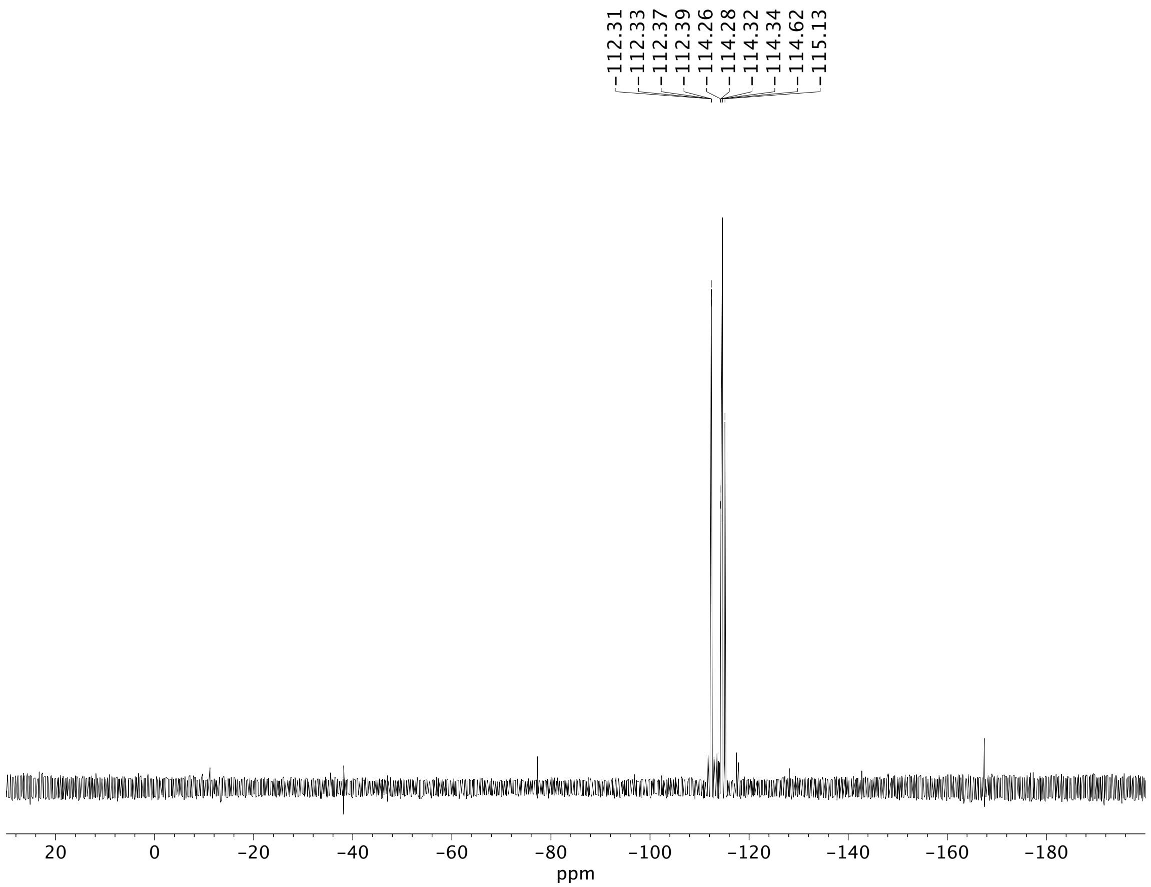

^{19}F NMR (282 MHz, $CDCl_3$) of compound **6o**.

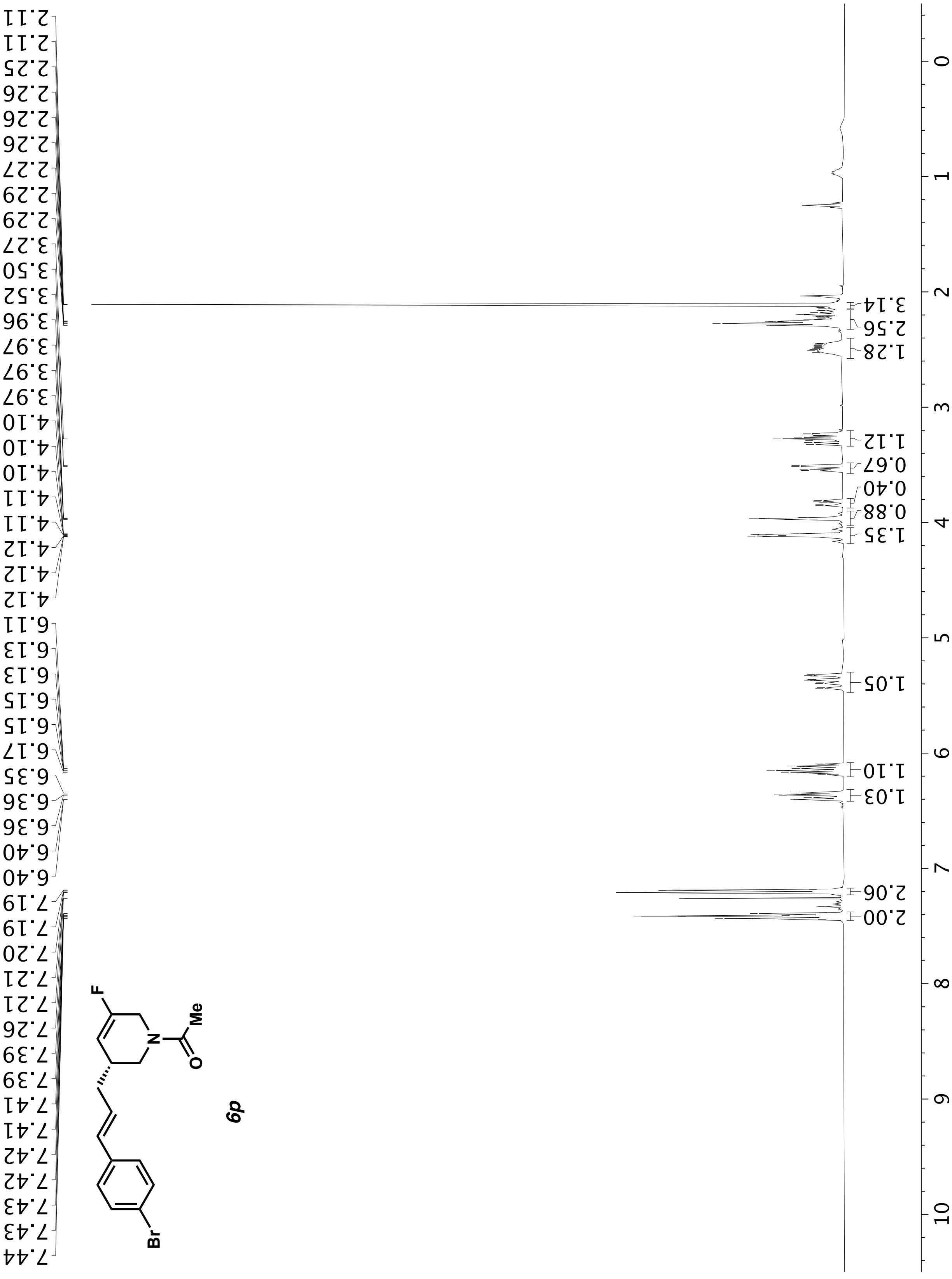

^{1}H NMR (400 MHz, $CDCl_3$) of compound **6p**.

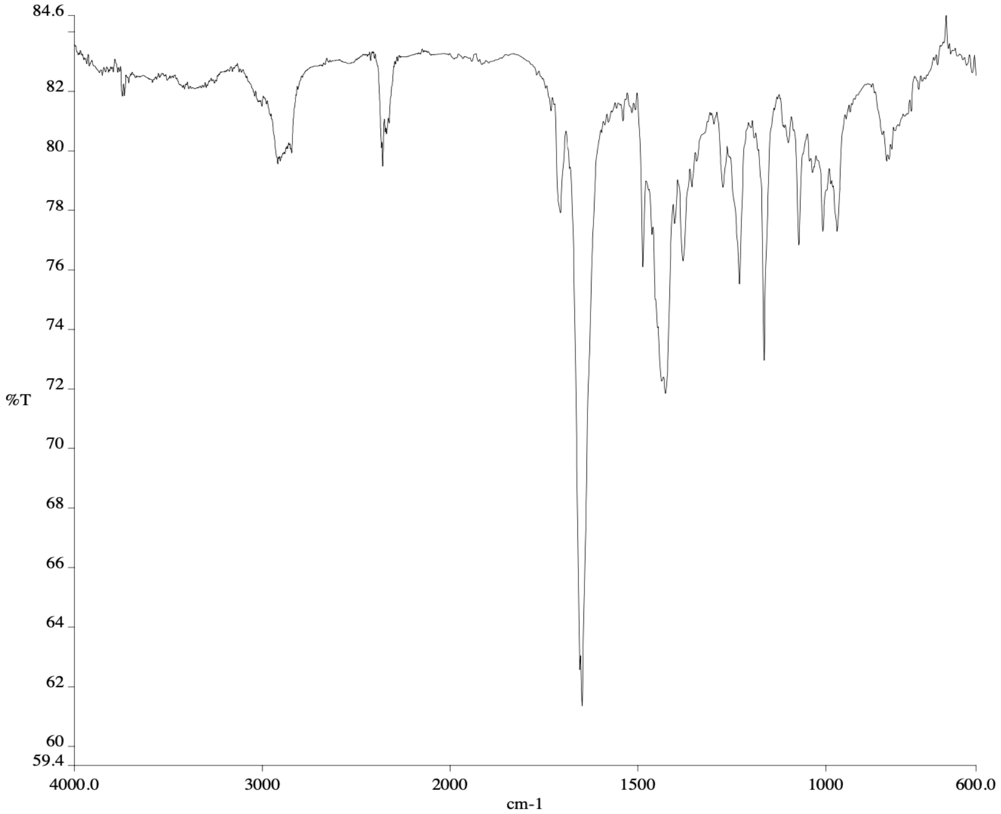

Infrared spectrum (Thin Film, NaCl) of compound **6p**.

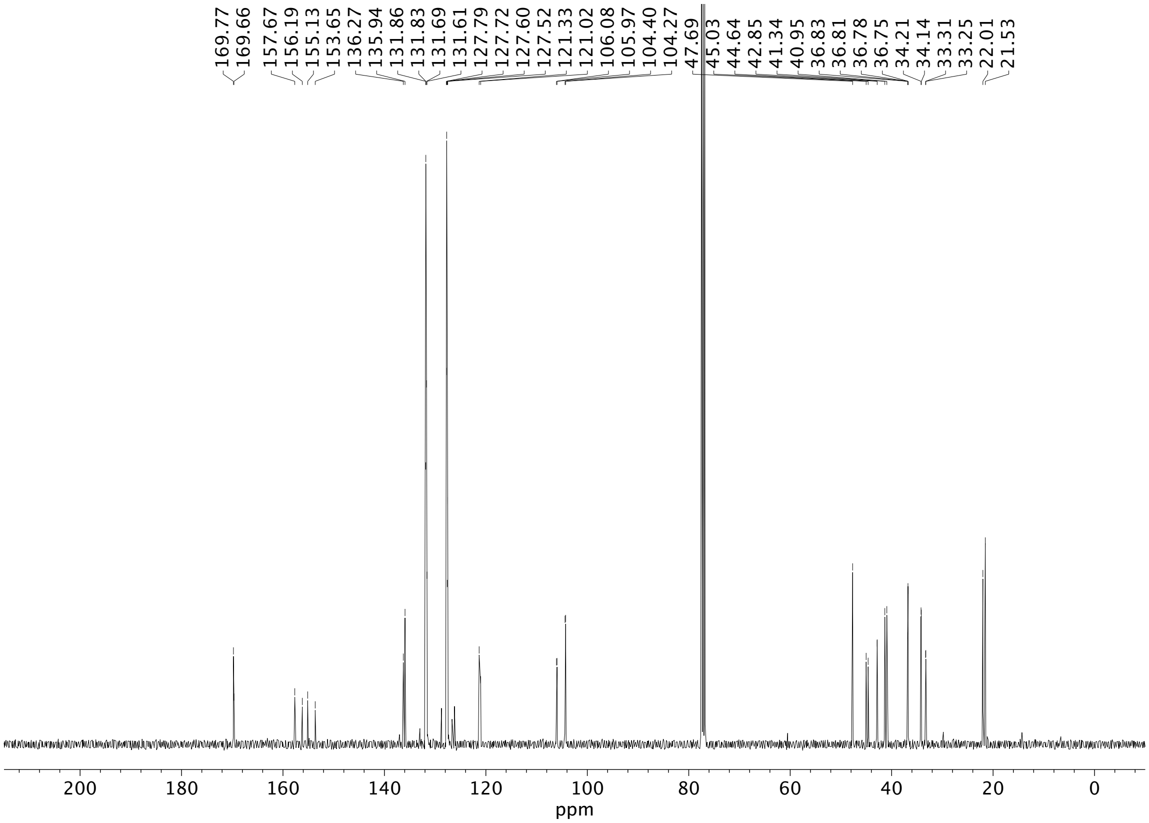

^{13}C NMR (100 MHz, $CDCl_3$) of compound **6p**.

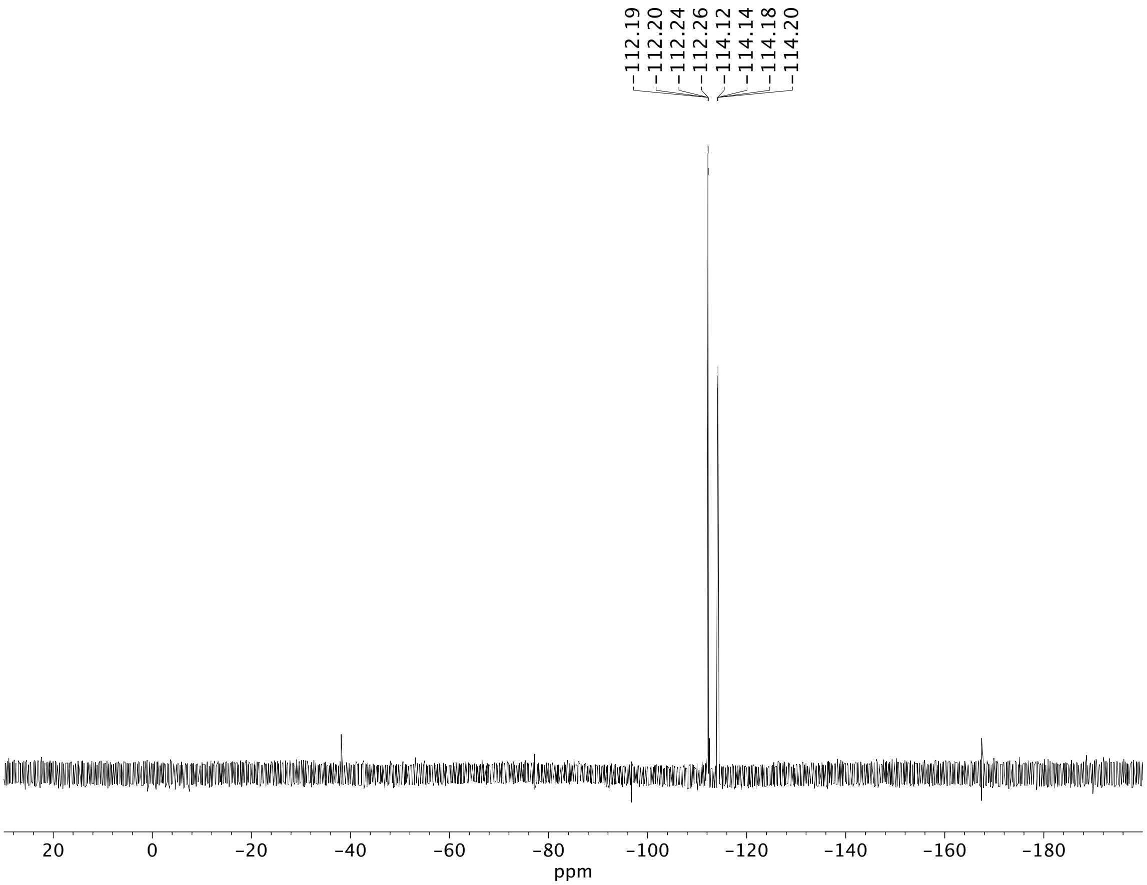

^{19}F NMR (282 MHz, $CDCl_3$) of compound **6p.**

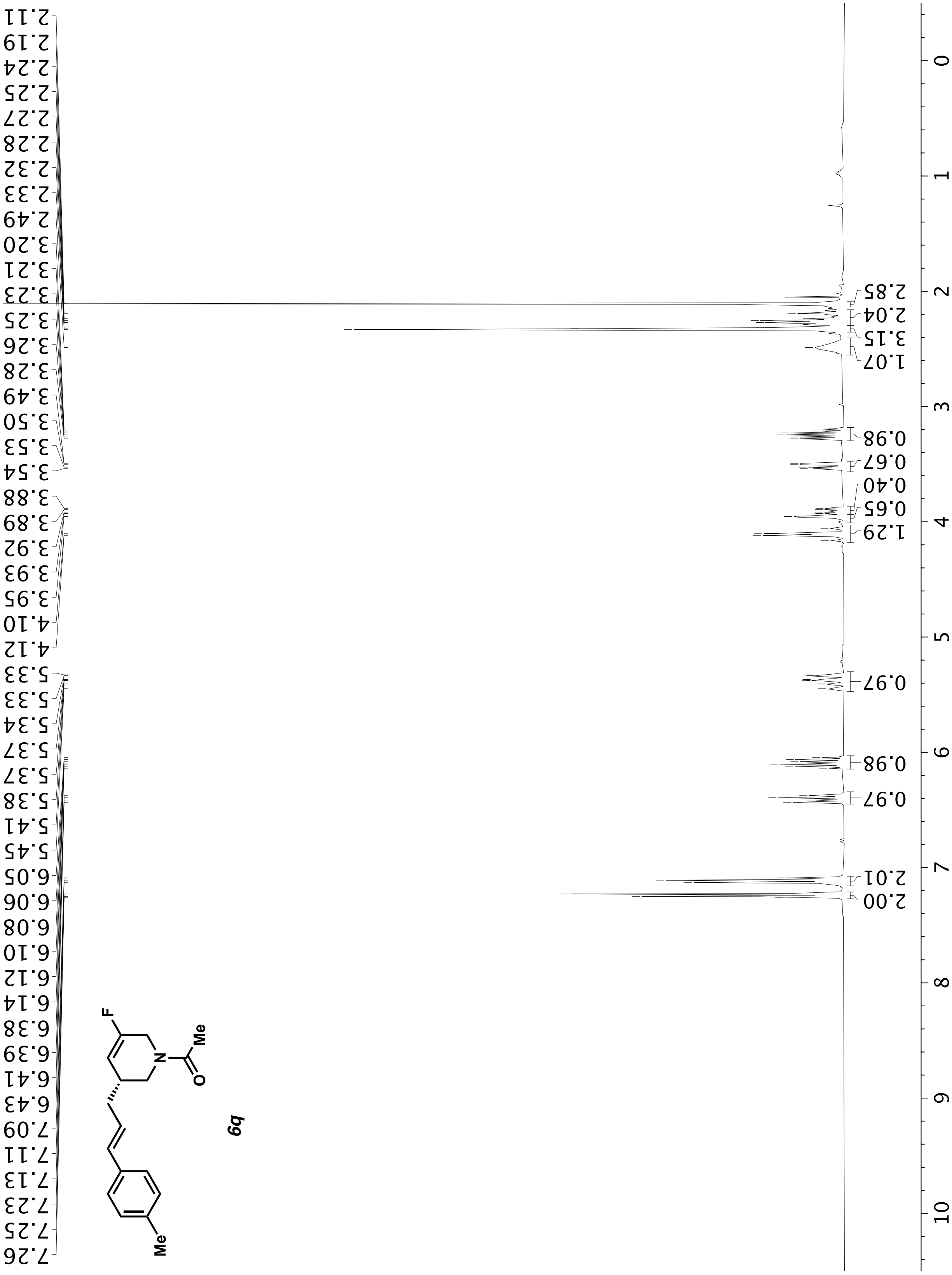

^{1}H NMR (400 MHz, $CDCl_3$) of compound **6q**.

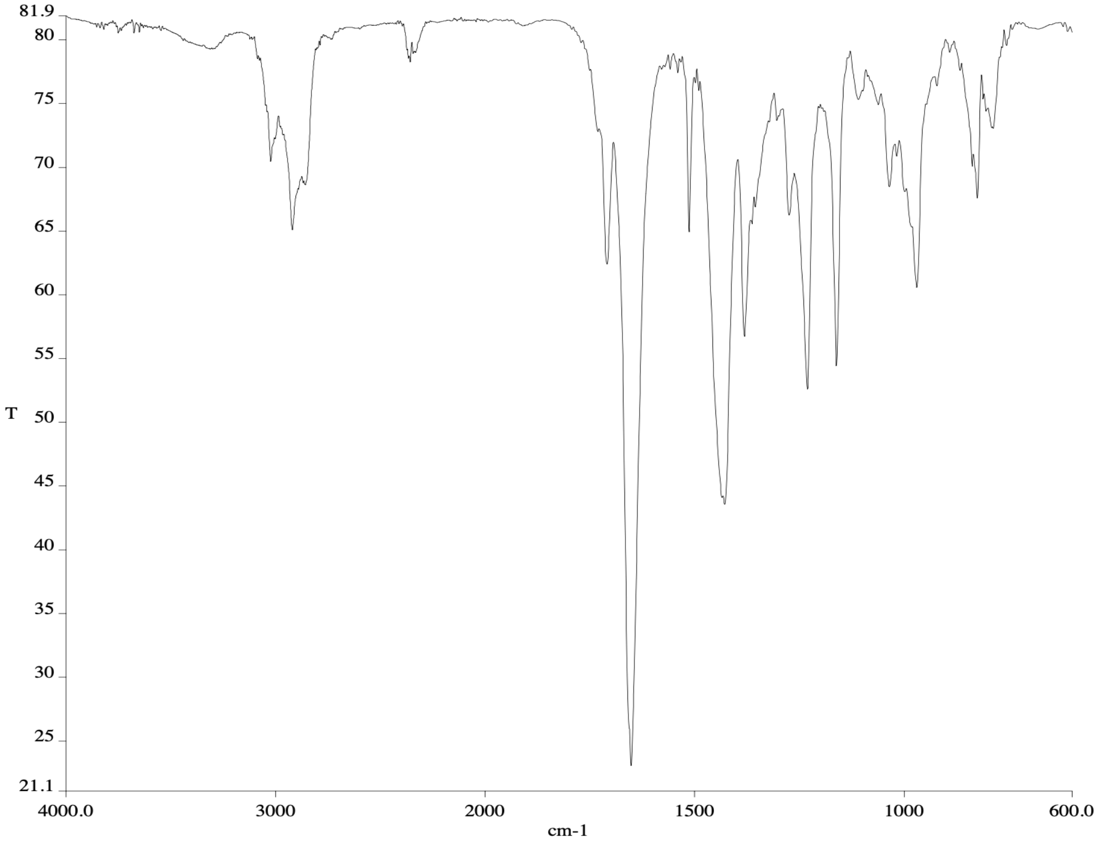

Infrared spectrum (Thin Film, NaCl) of compound **6q**.

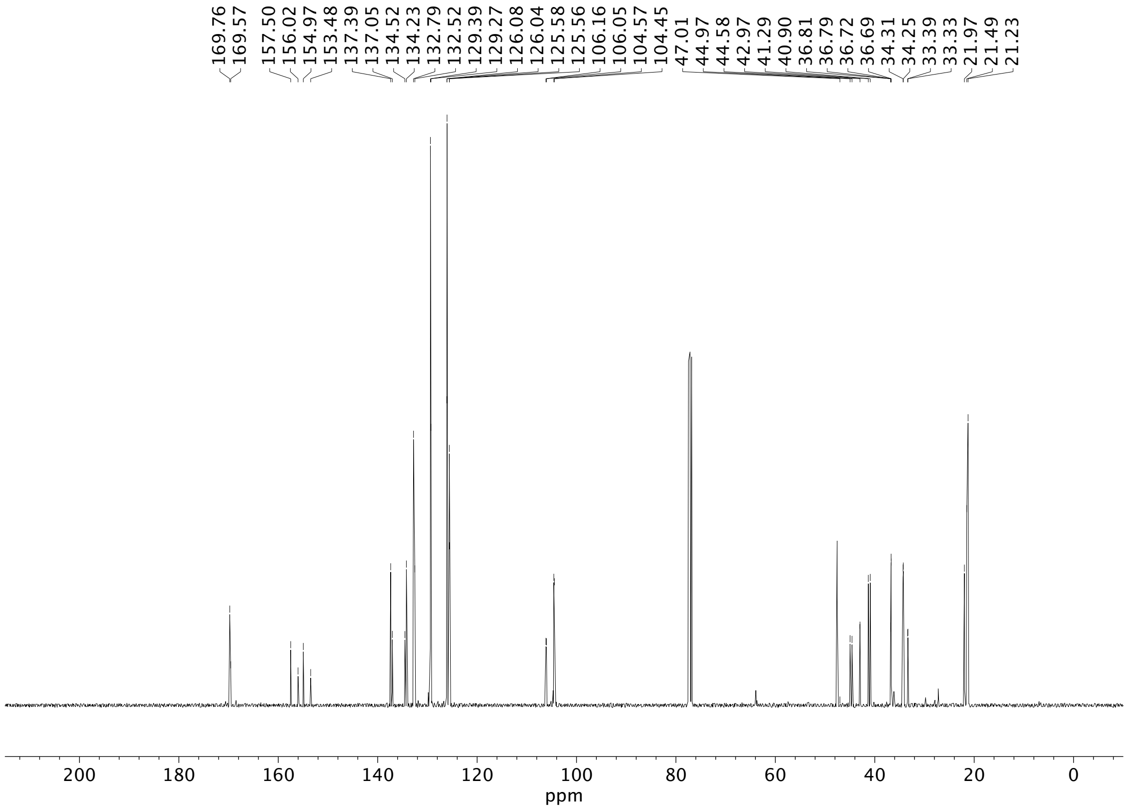

^{13}C NMR (100 MHz, $CDCl_3$) of compound **6q**.

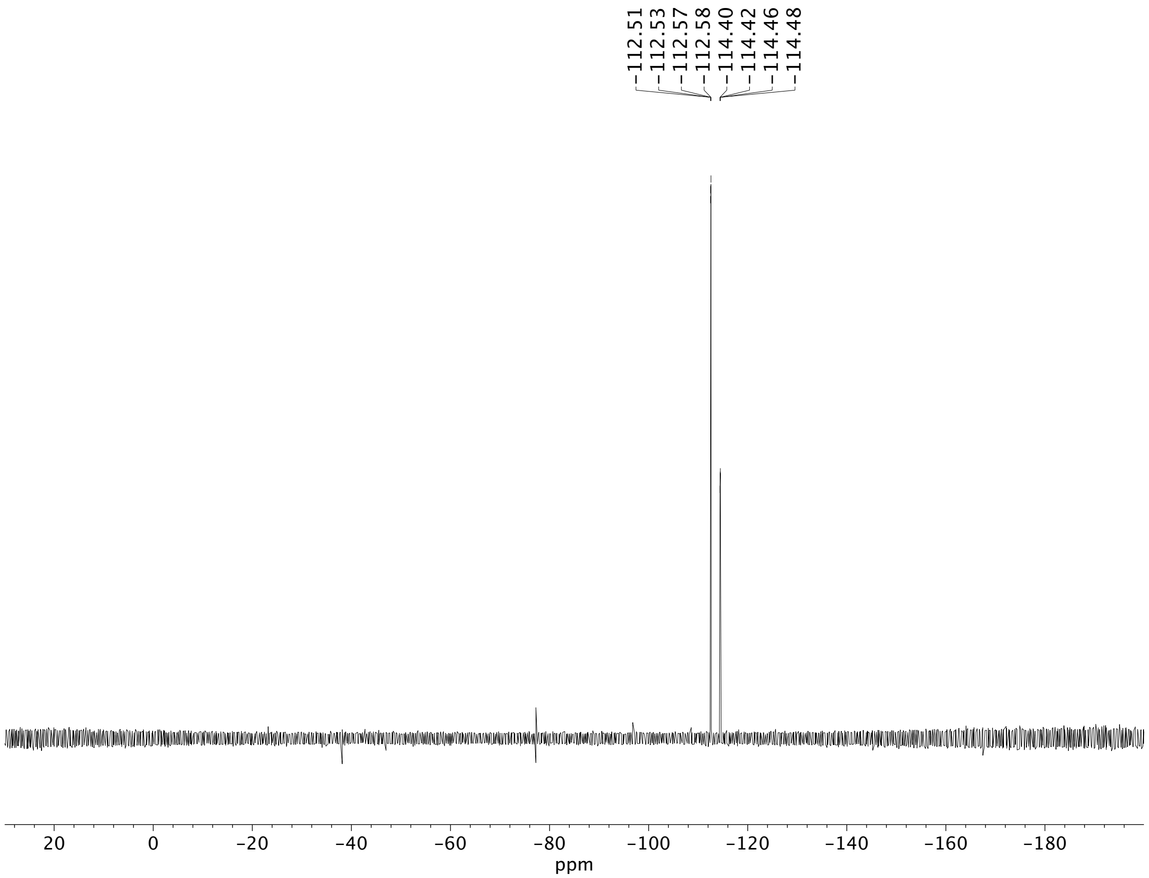

^{19}F NMR (282 MHz, $CDCl_3$) of compound **6q**.

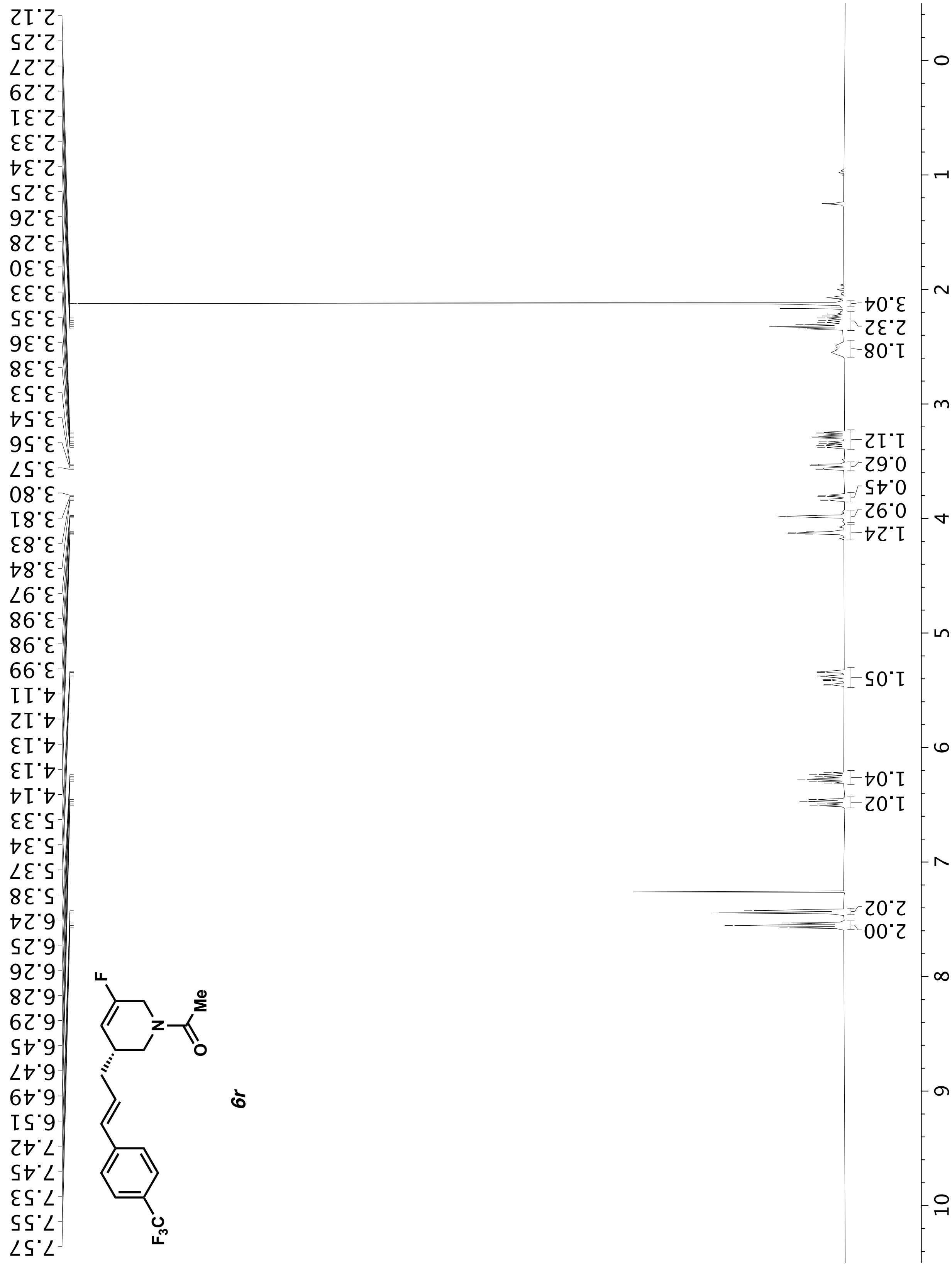

^{1}H NMR (400 MHz, $CDCl_3$) of compound **6r**.

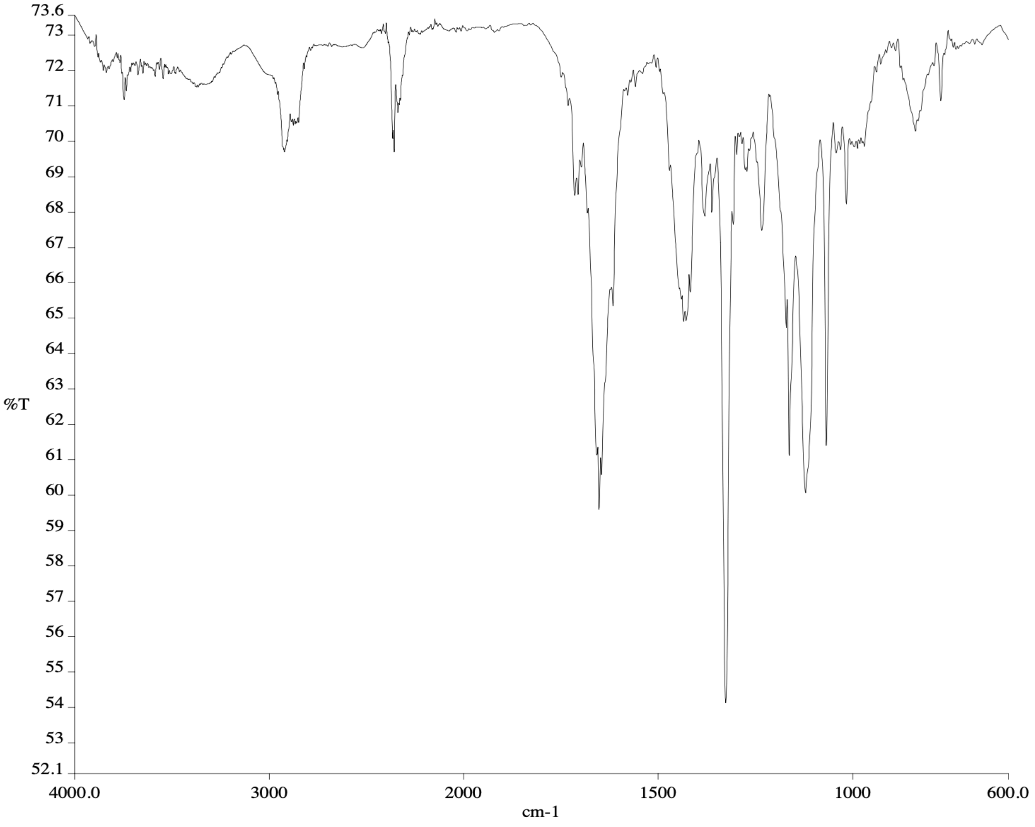

Infrared spectrum (Thin Film, NaCl) of compound **6r**.

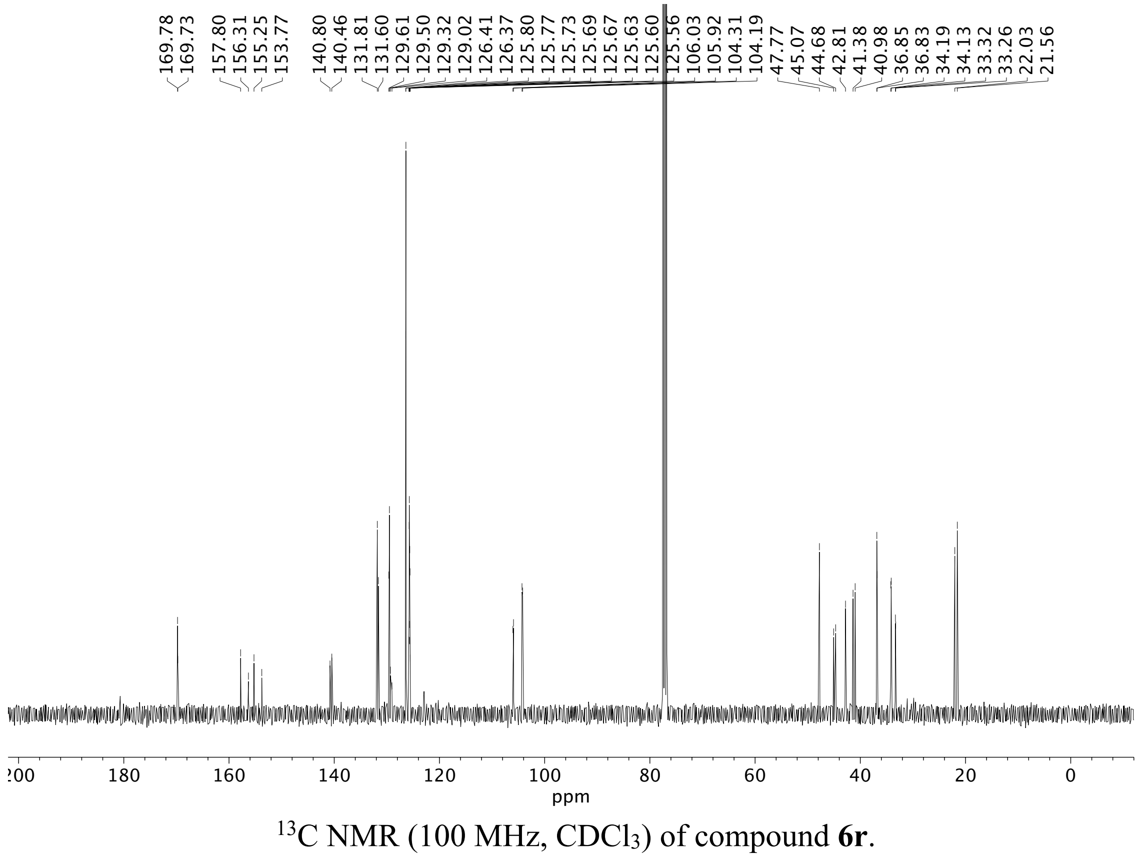

^{13}C NMR (100 MHz, $CDCl_3$) of compound **6r**.

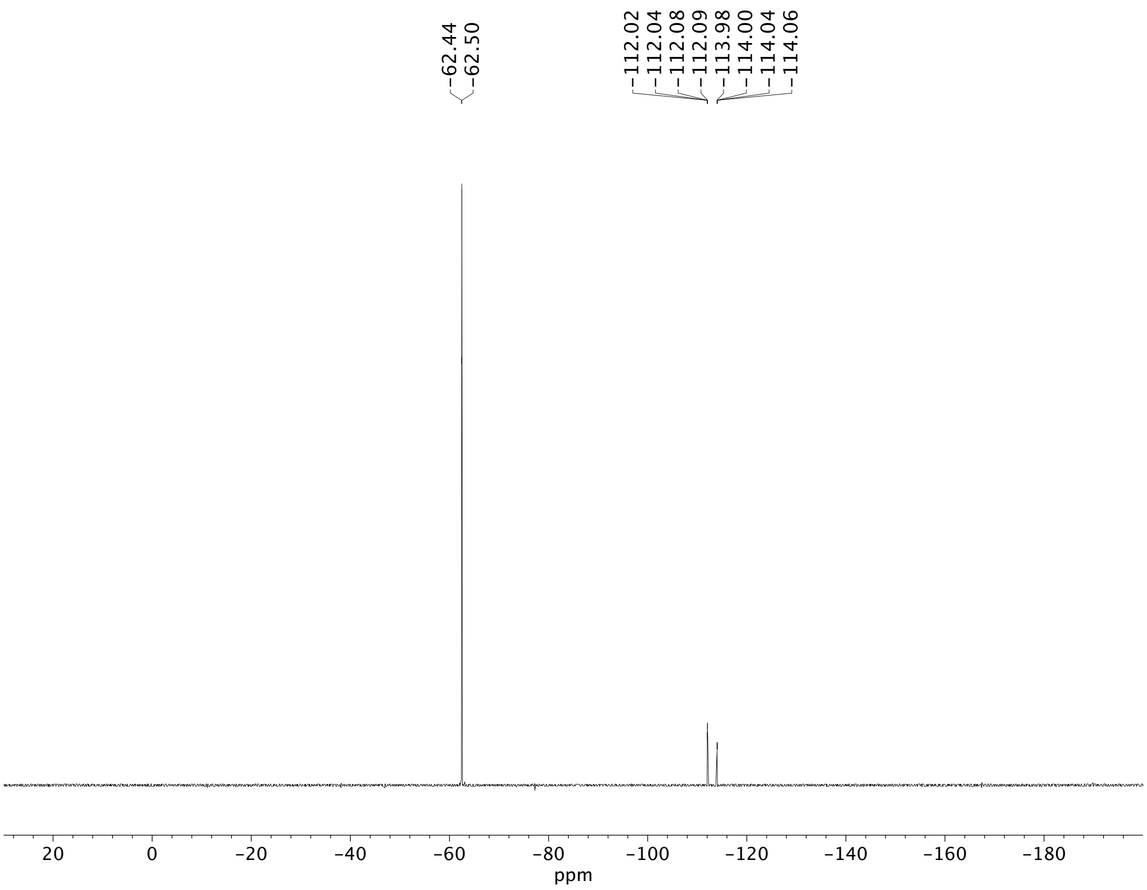

^{19}F NMR (282 MHz, $CDCl_3$) of compound **6r**.

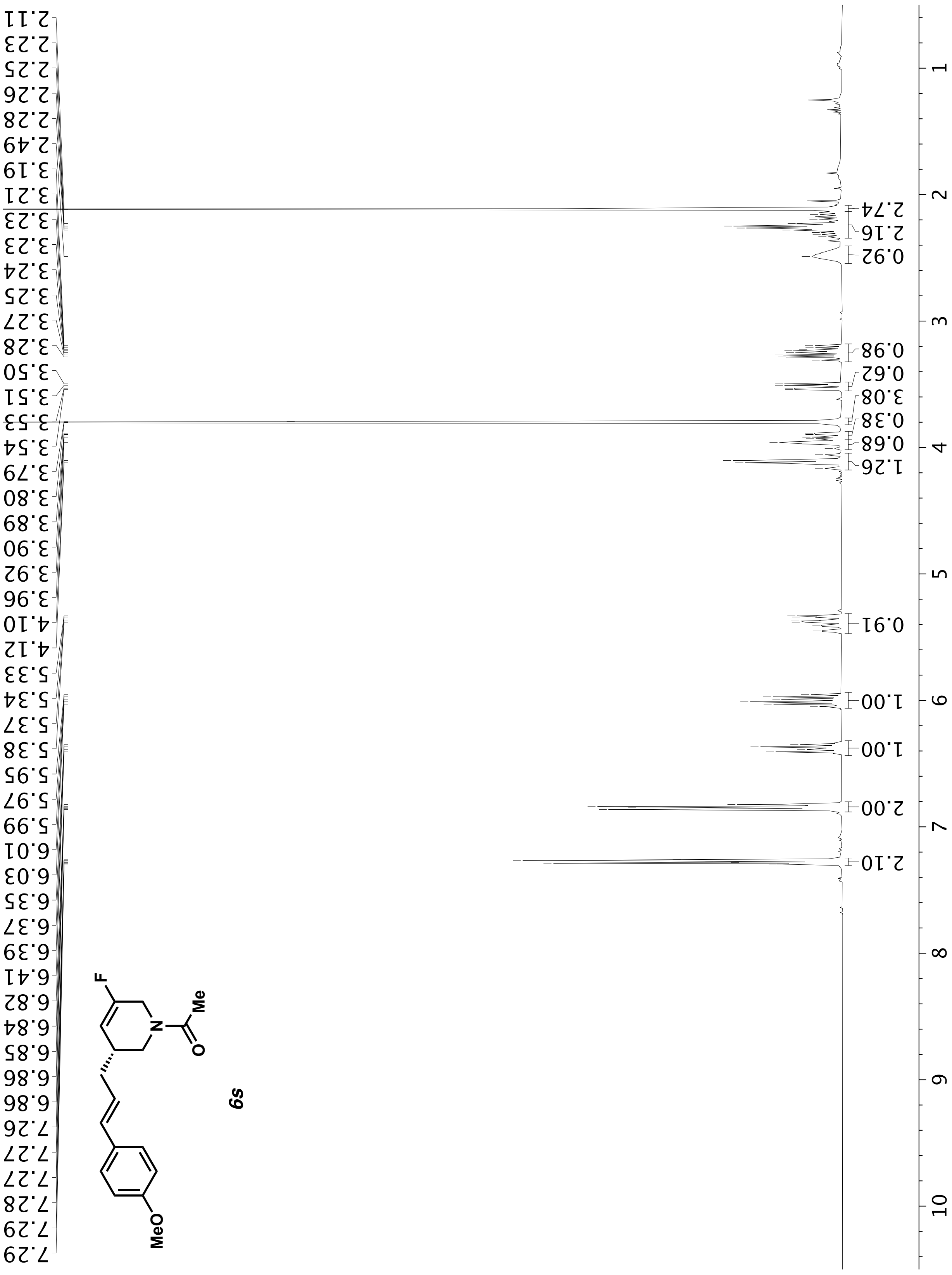

^{1}H NMR (400 MHz, $CDCl_3$) of compound **6s**.

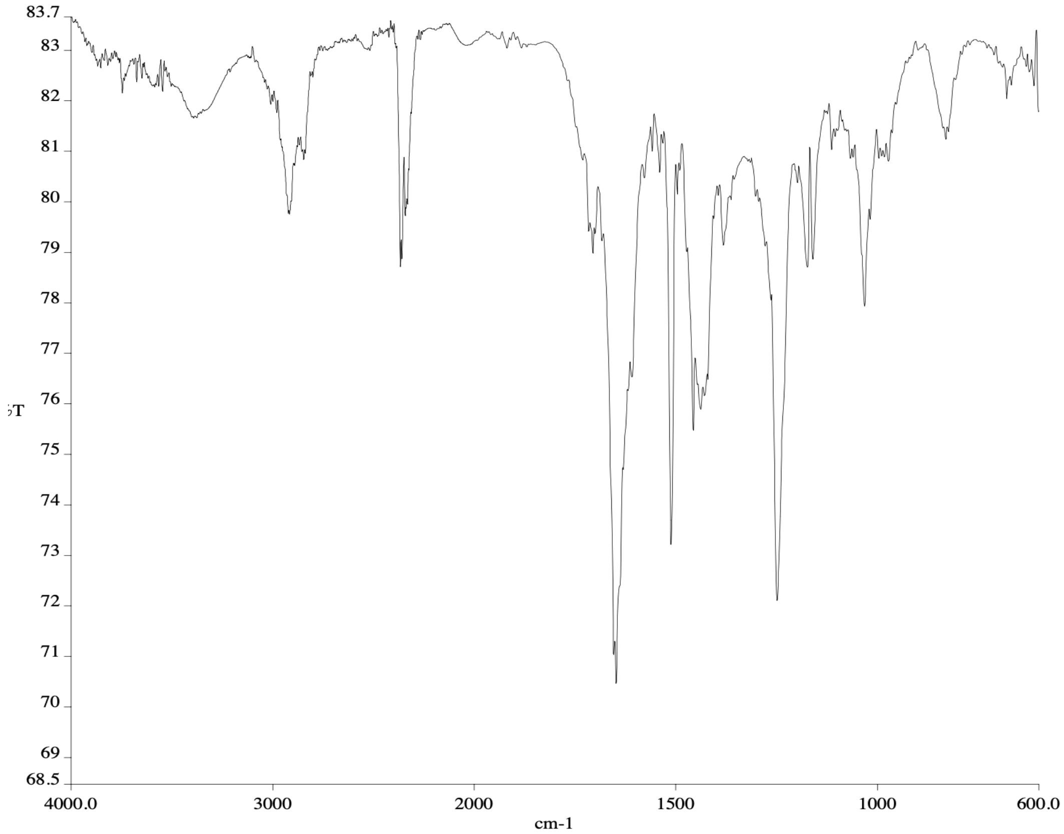

Infrared spectrum (Thin Film, NaCl) of compound **6s**.

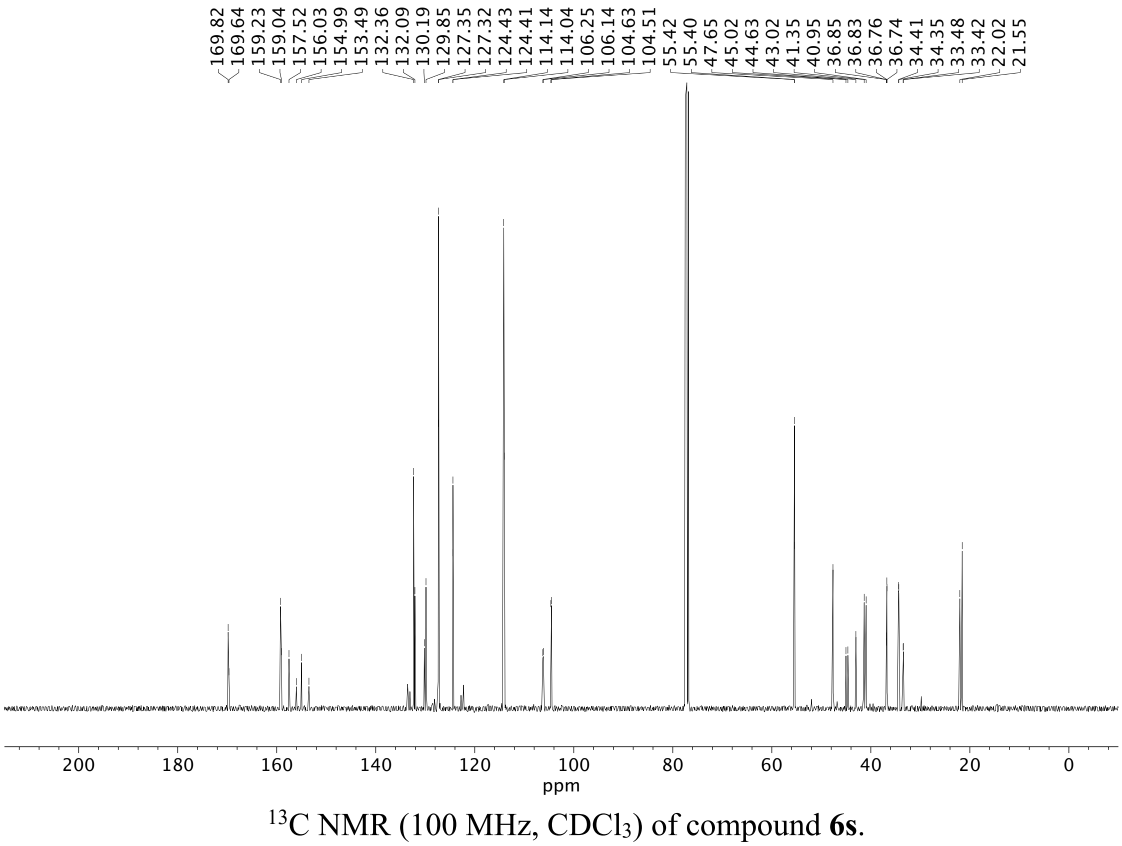

^{13}C NMR (100 MHz, $CDCl_3$) of compound **6s**.

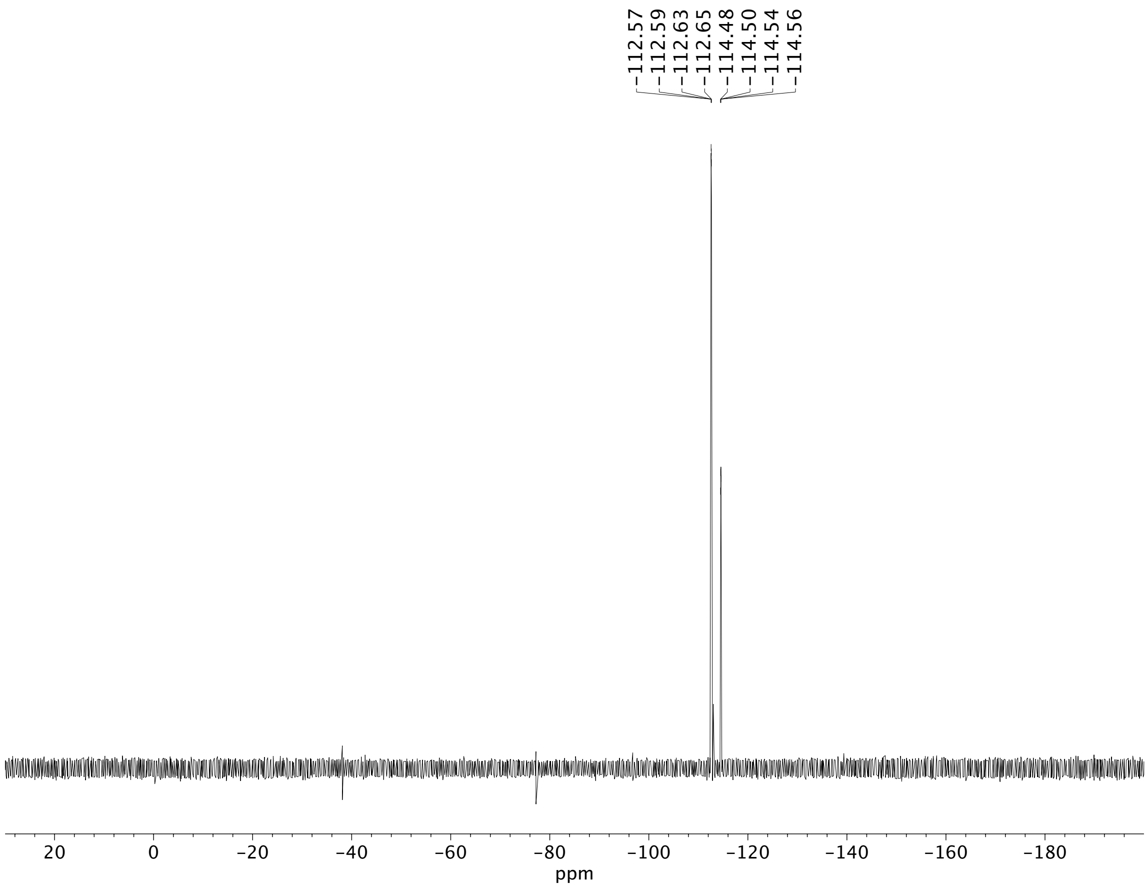

^{19}F NMR (282 MHz, $CDCl_3$) of compound **6s**.

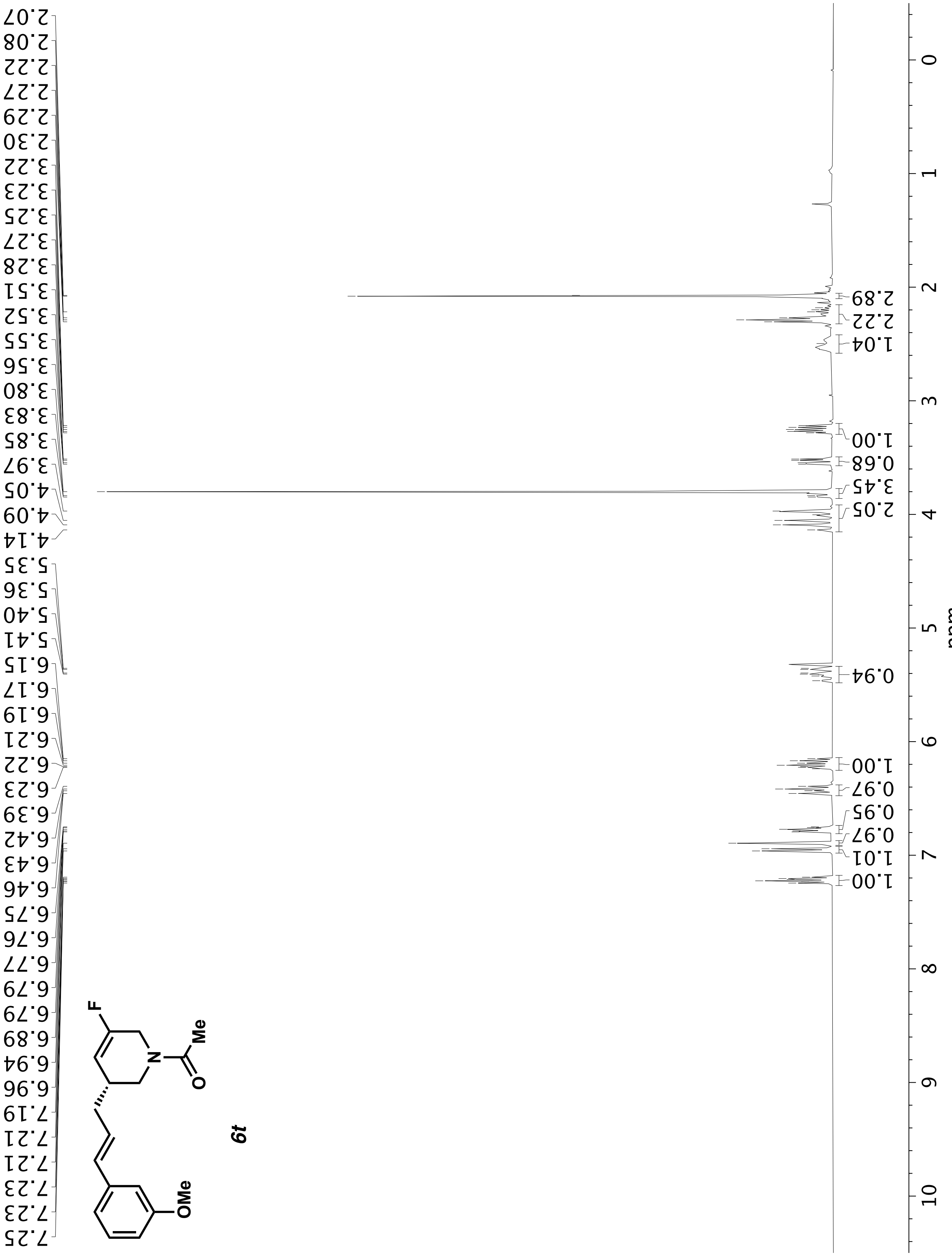

^{1}H NMR (400 MHz, CD_2Cl_2) of compound **6t**.

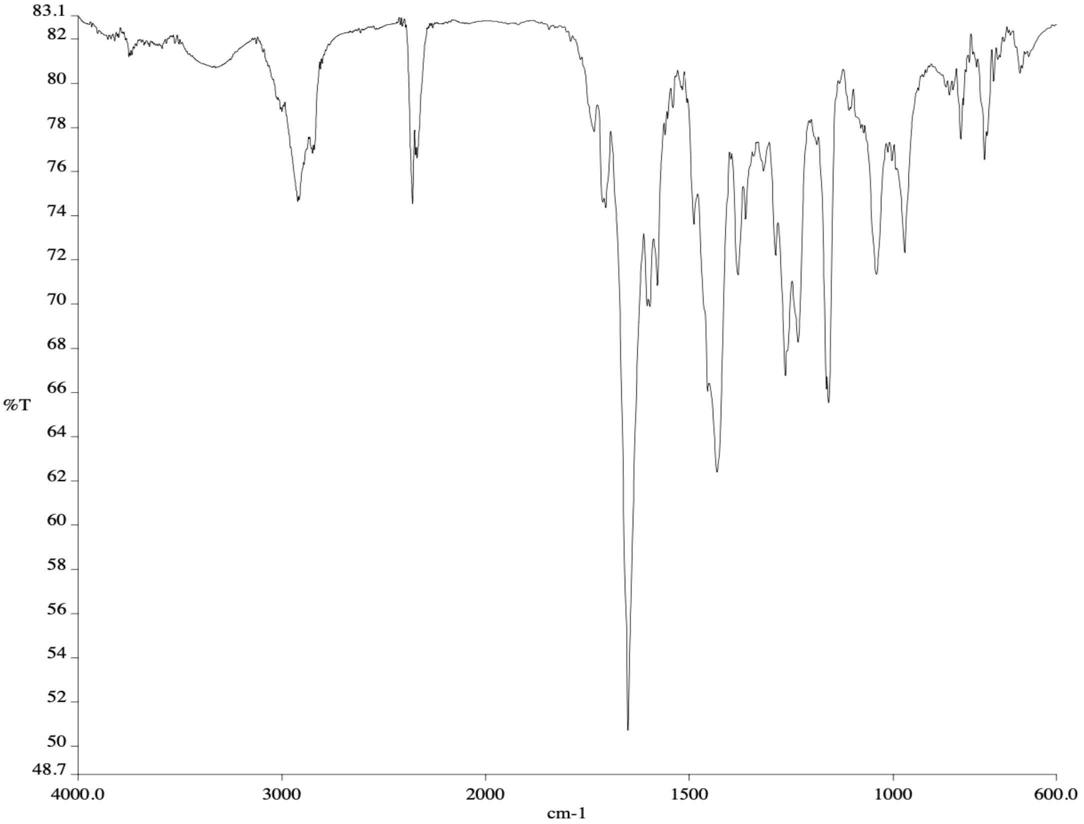

Infrared spectrum (Thin Film, NaCl) of compound **6t**.

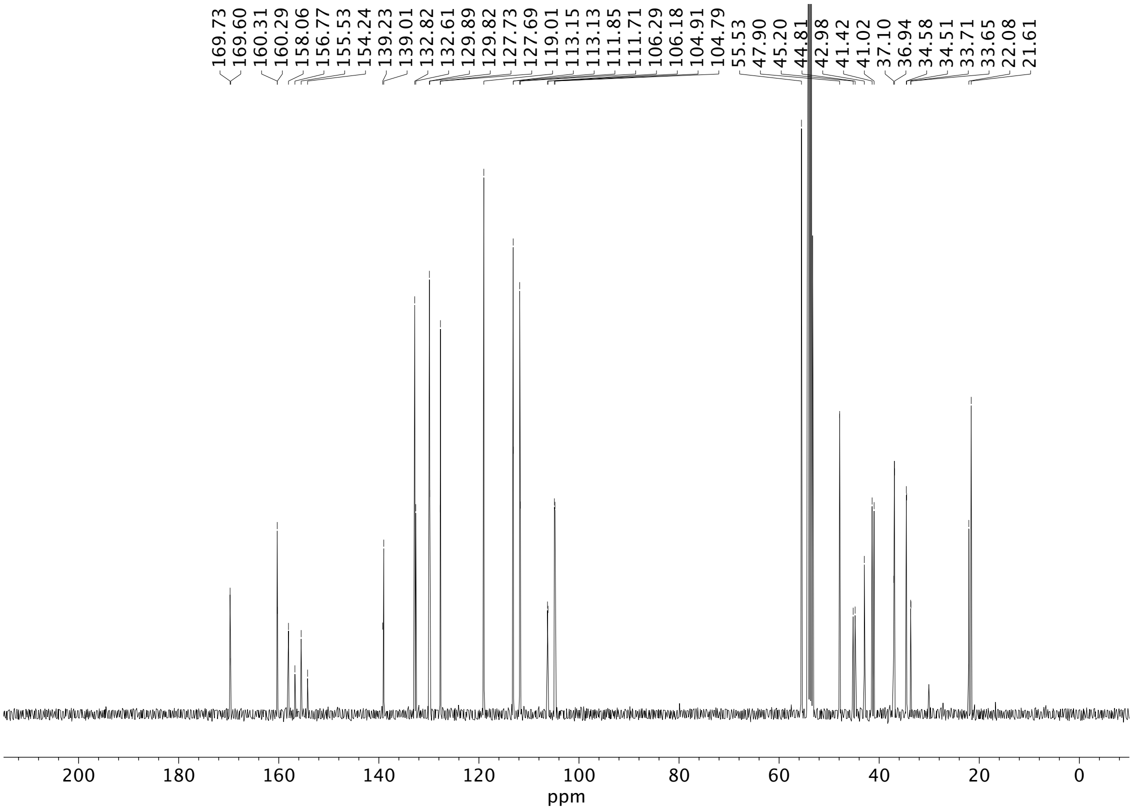

^{13}C NMR (100 MHz, CD_2Cl_2) of compound **6t**.

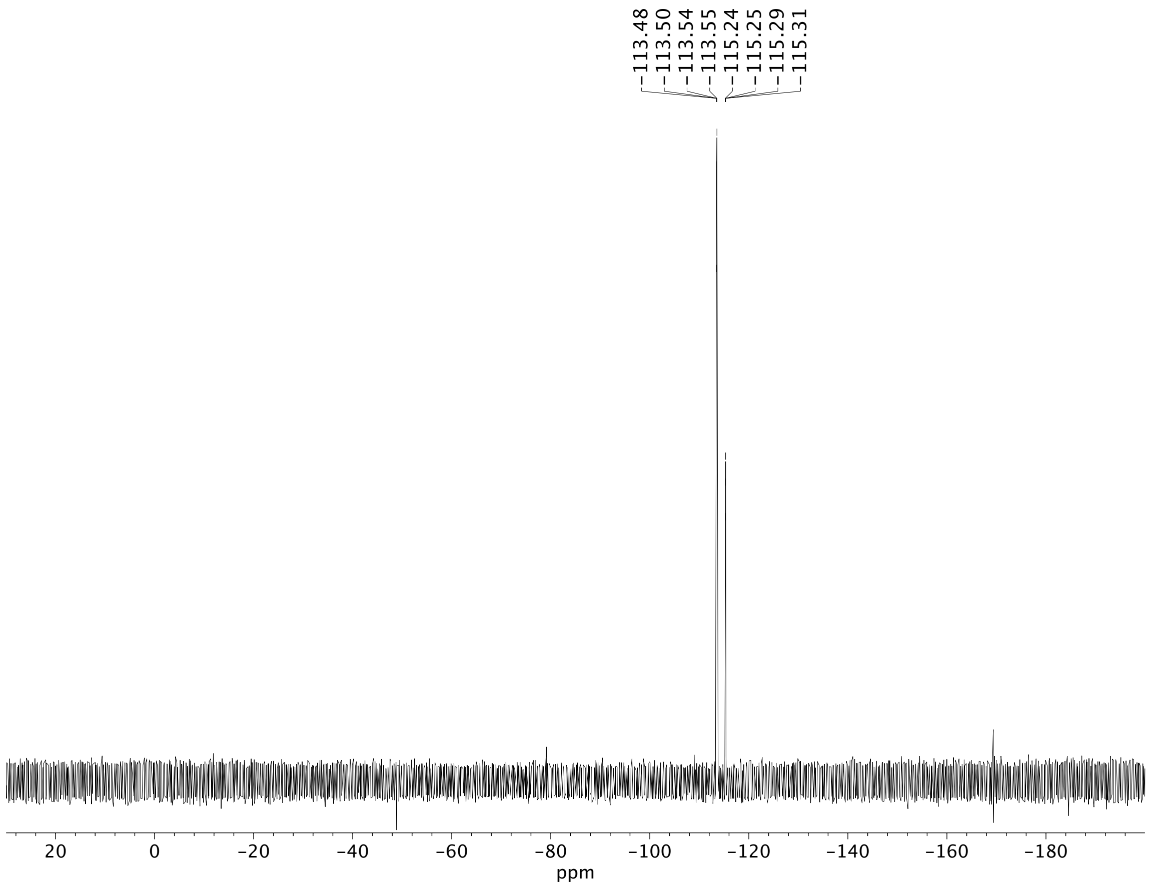

^{19}F NMR (282 MHz, CD_2Cl_2) of compound **6t.**

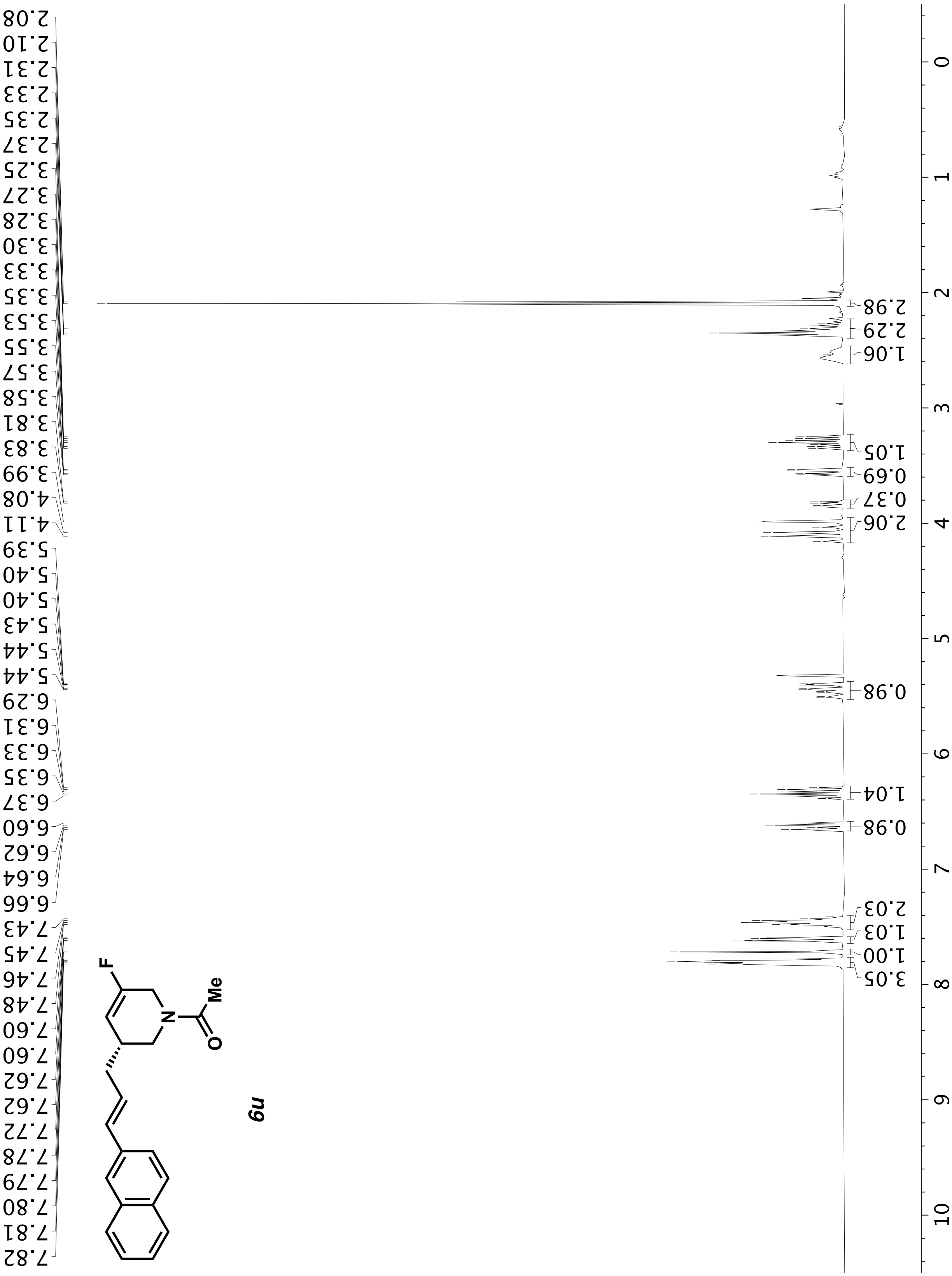

^{1}H NMR (400 MHz, CD_2Cl_2) of compound **6u.**

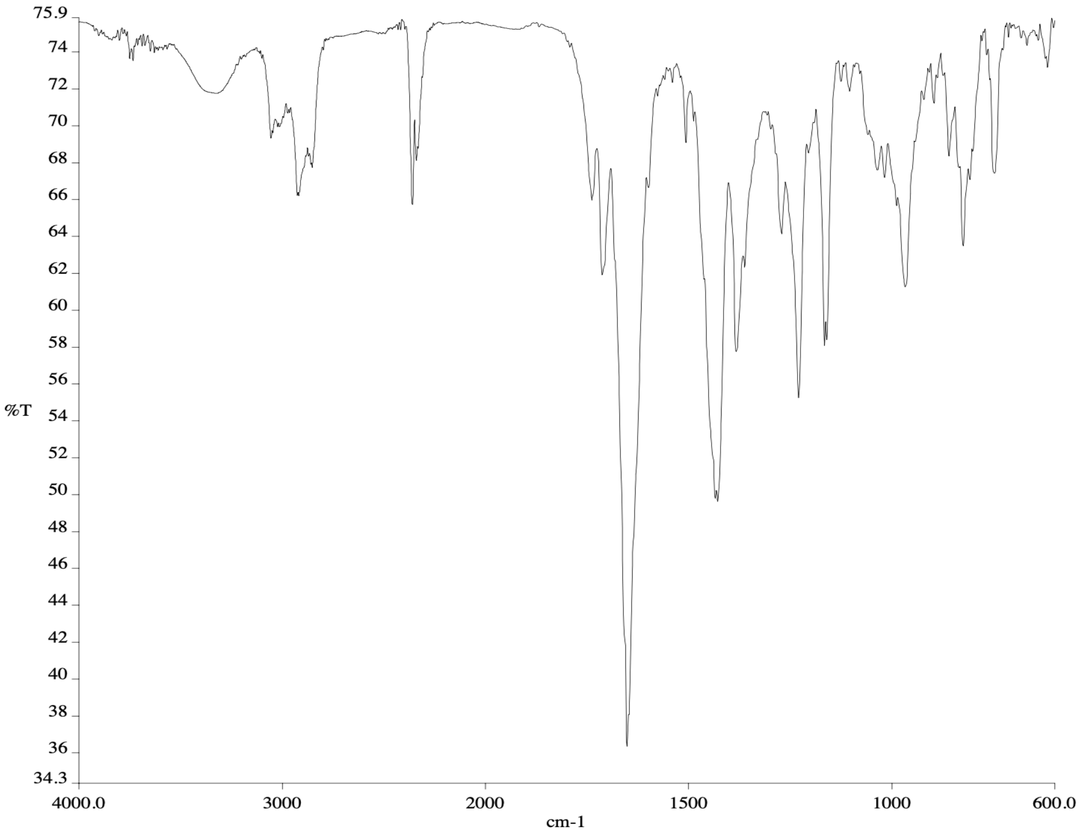

Infrared spectrum (Thin Film, NaCl) of compound **6u**.

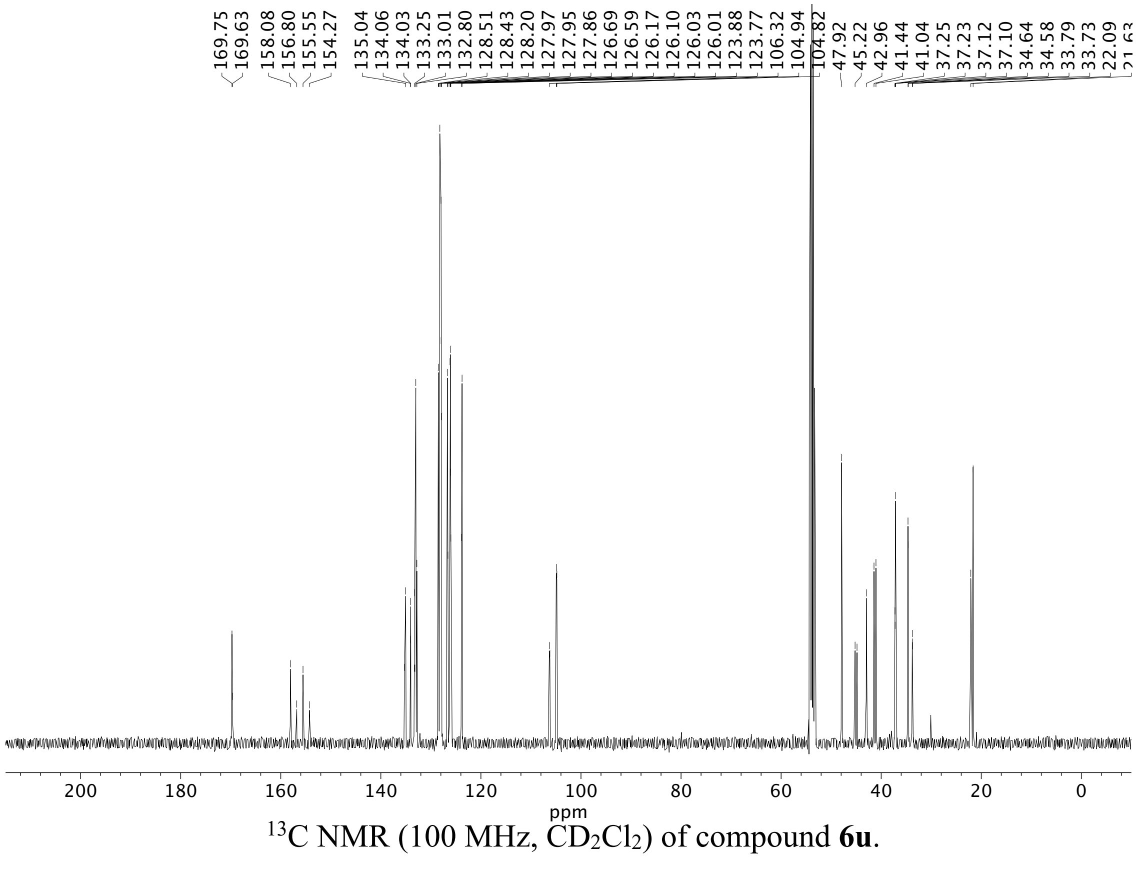

^{13}C NMR (100 MHz, CD_2Cl_2) of compound **6u**.

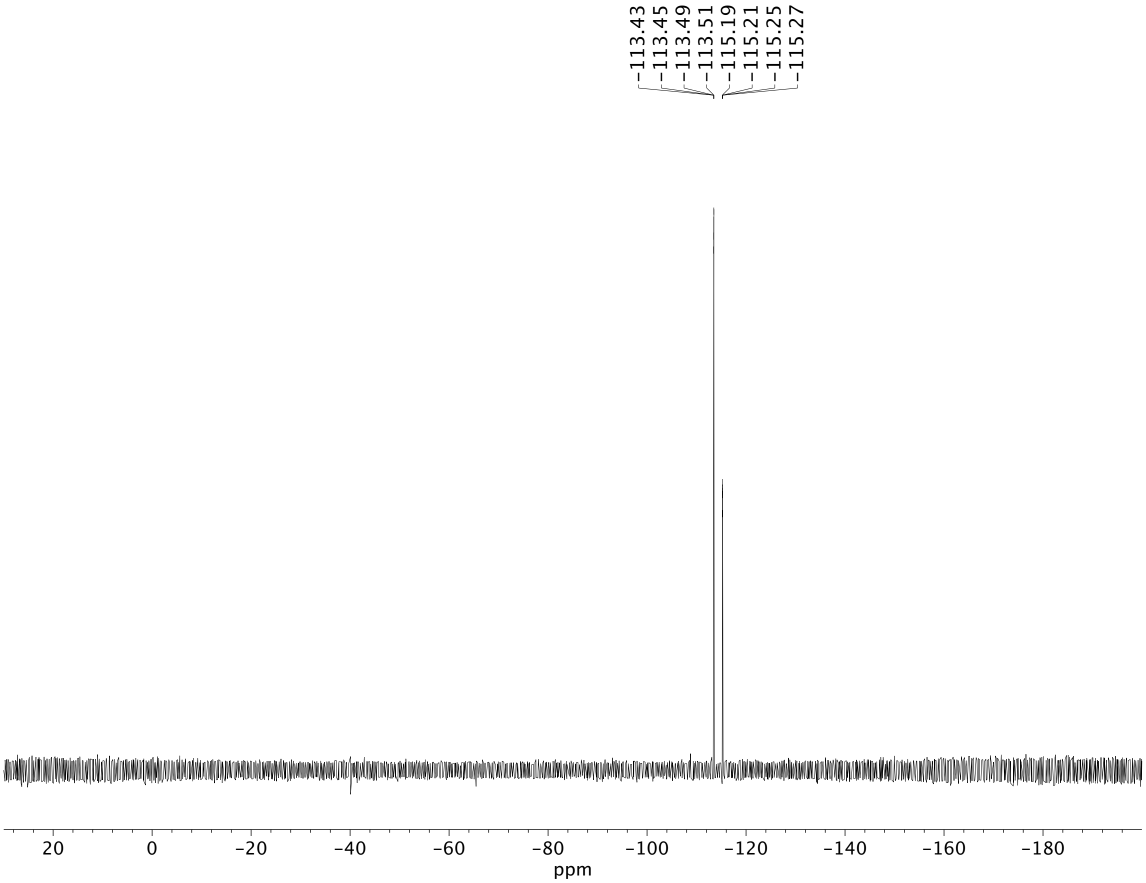

^{19}F NMR (282 MHz, CD_2Cl_2) of compound **6u.**

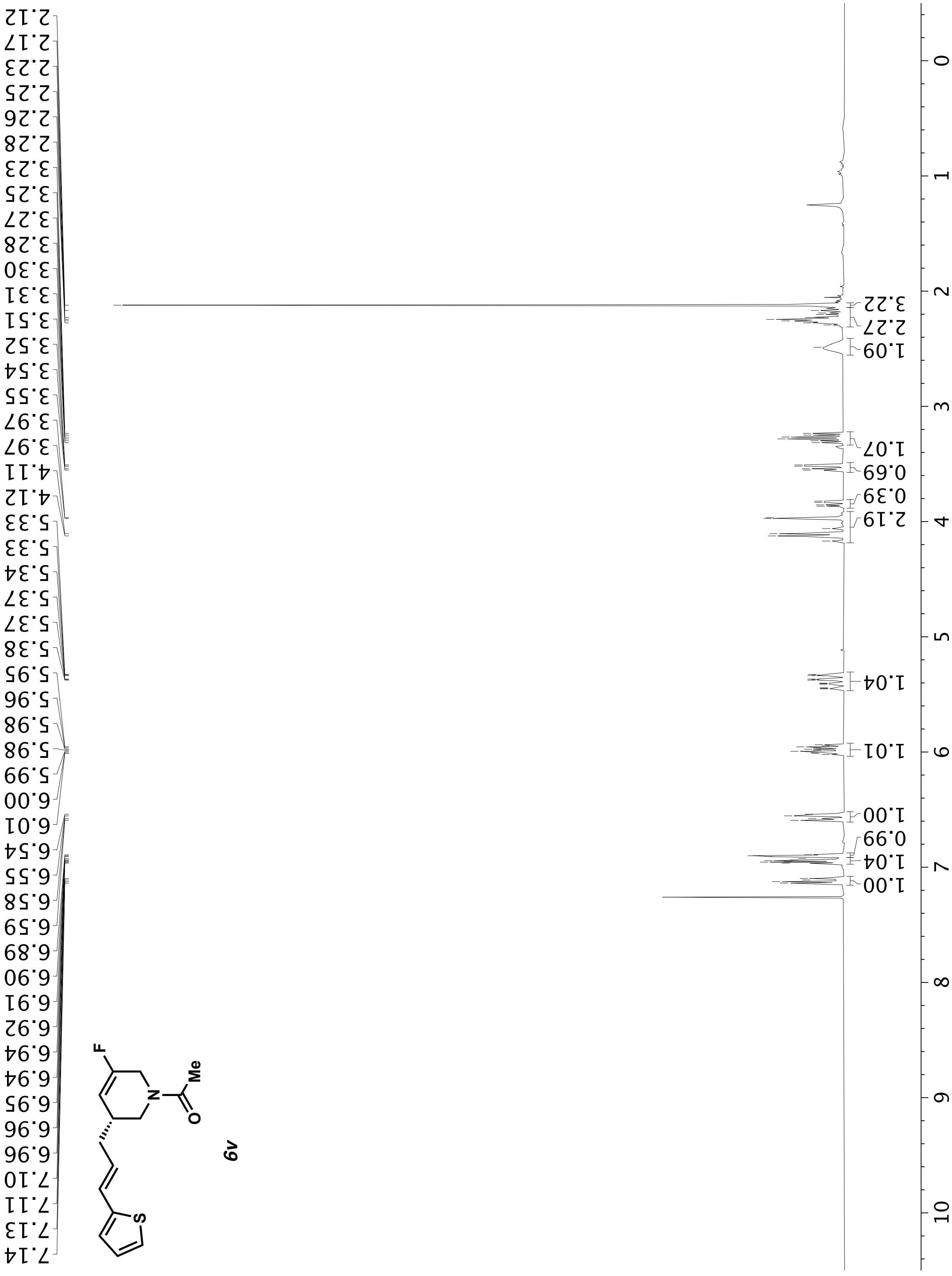

^{1}H NMR (400 MHz, $CDCl_3$) of compound **6v**.

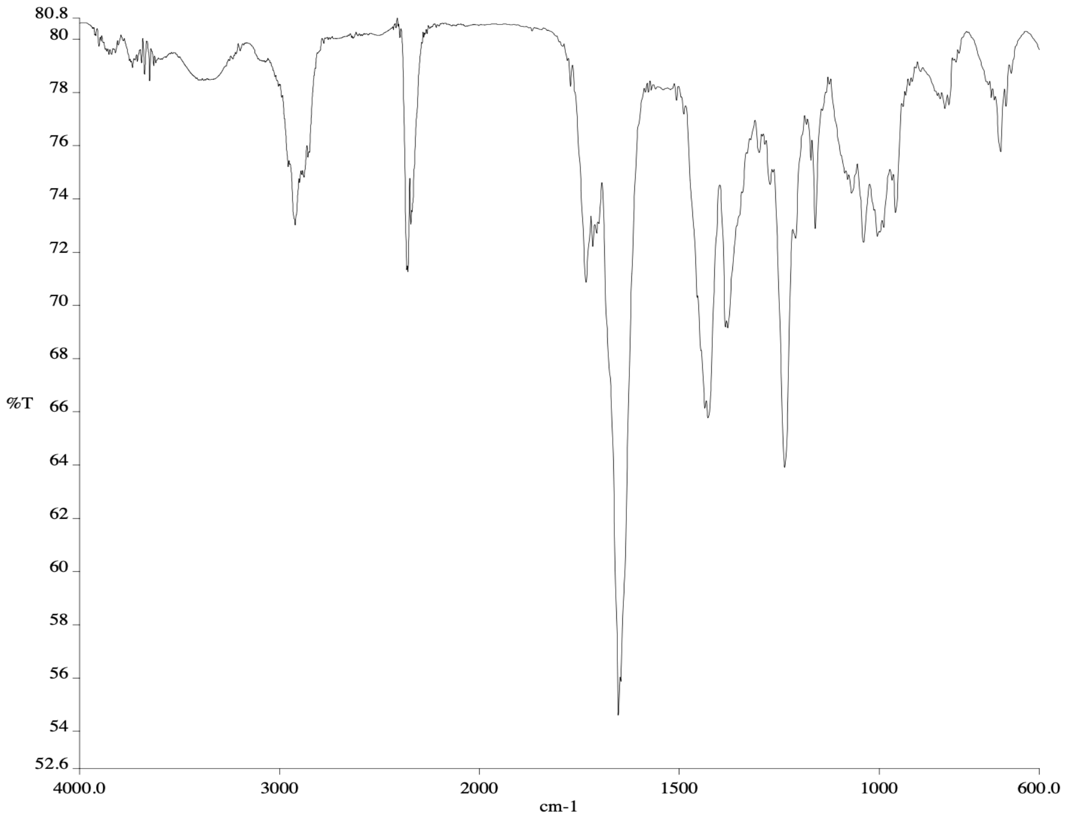

Infrared spectrum (Thin Film, NaCl) of compound **6v.**

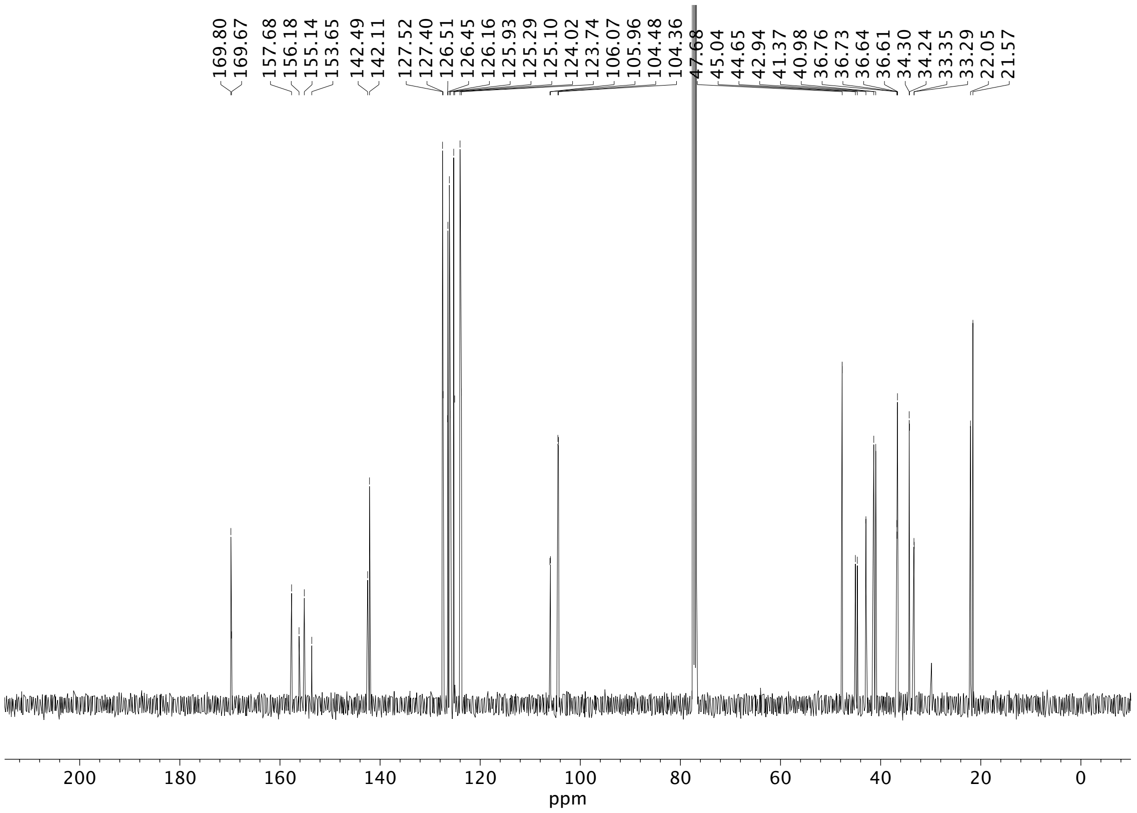

^{13}C NMR (100 MHz, $CDCl_3$) of compound **6v**.

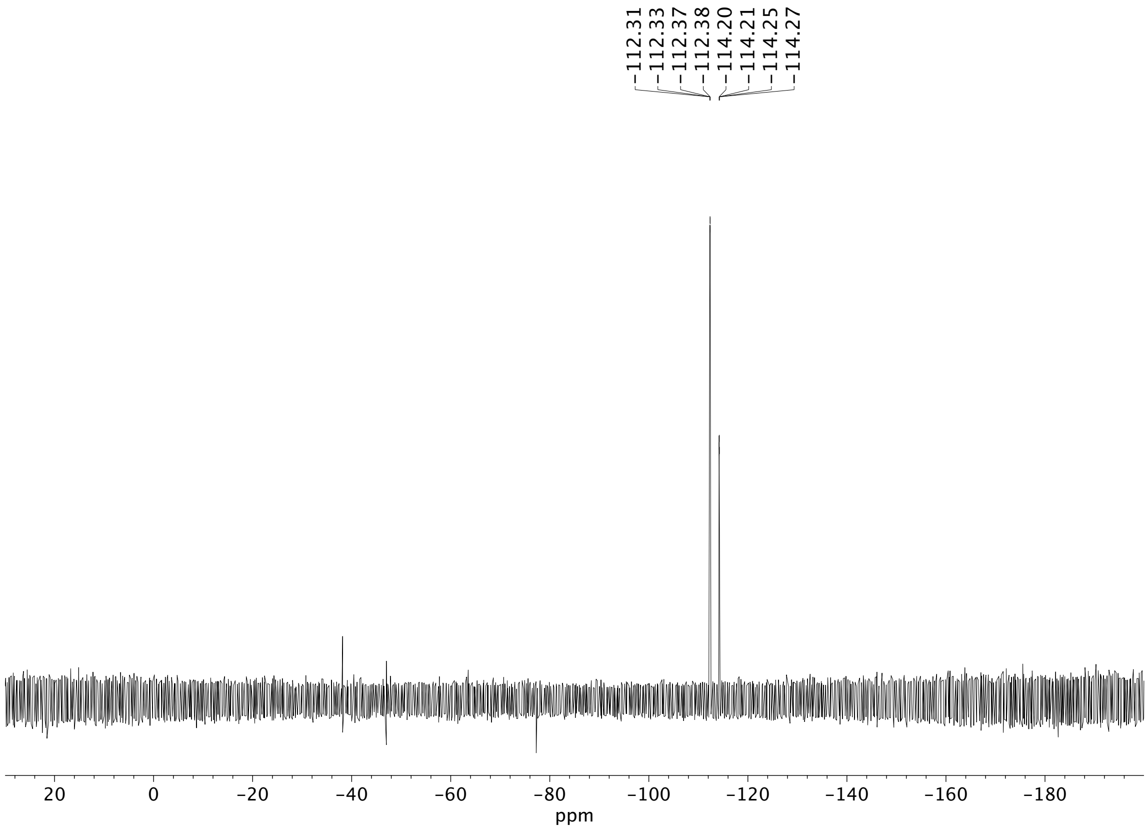

^{19}F NMR (282 MHz, $CDCl_3$) of compound **6v**.

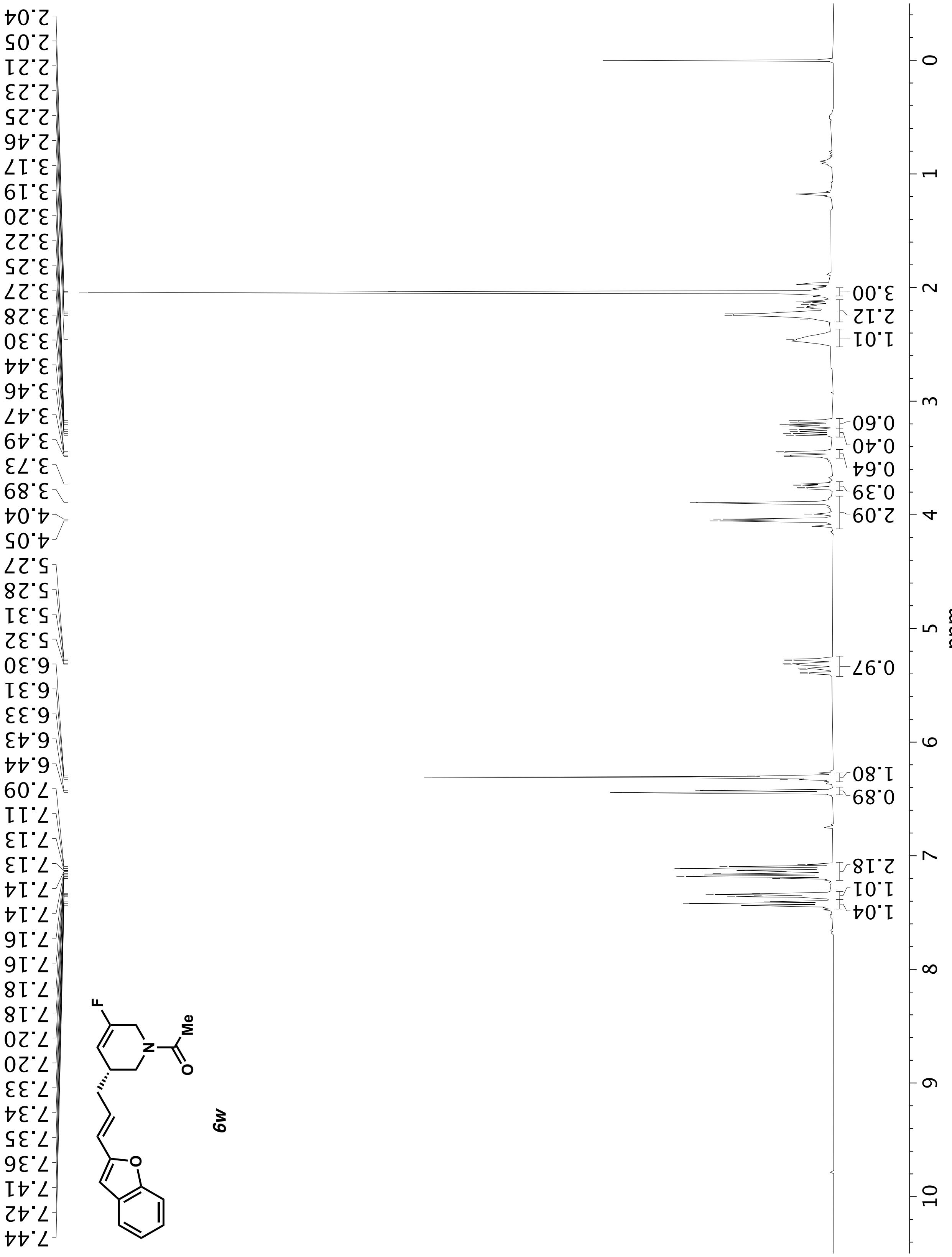

^{1}H NMR (400 MHz, $CDCl_3$) of compound **6w**.

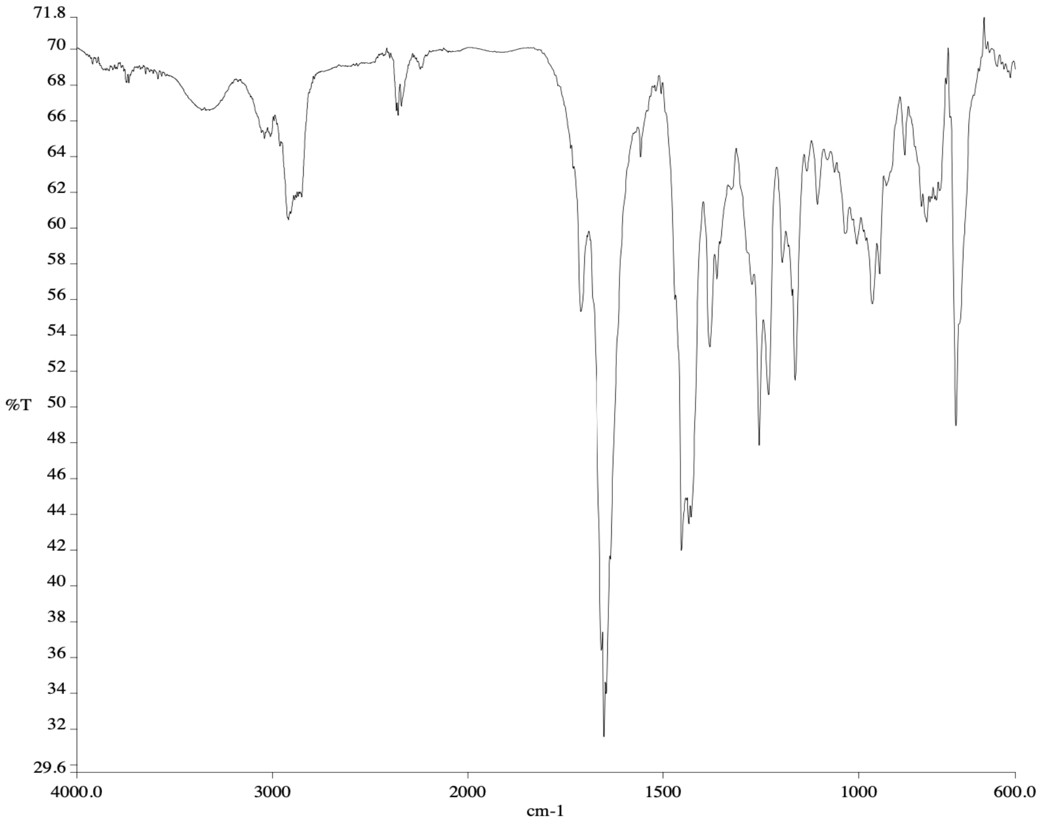

Infrared spectrum (Thin Film, NaCl) of compound **6w**.

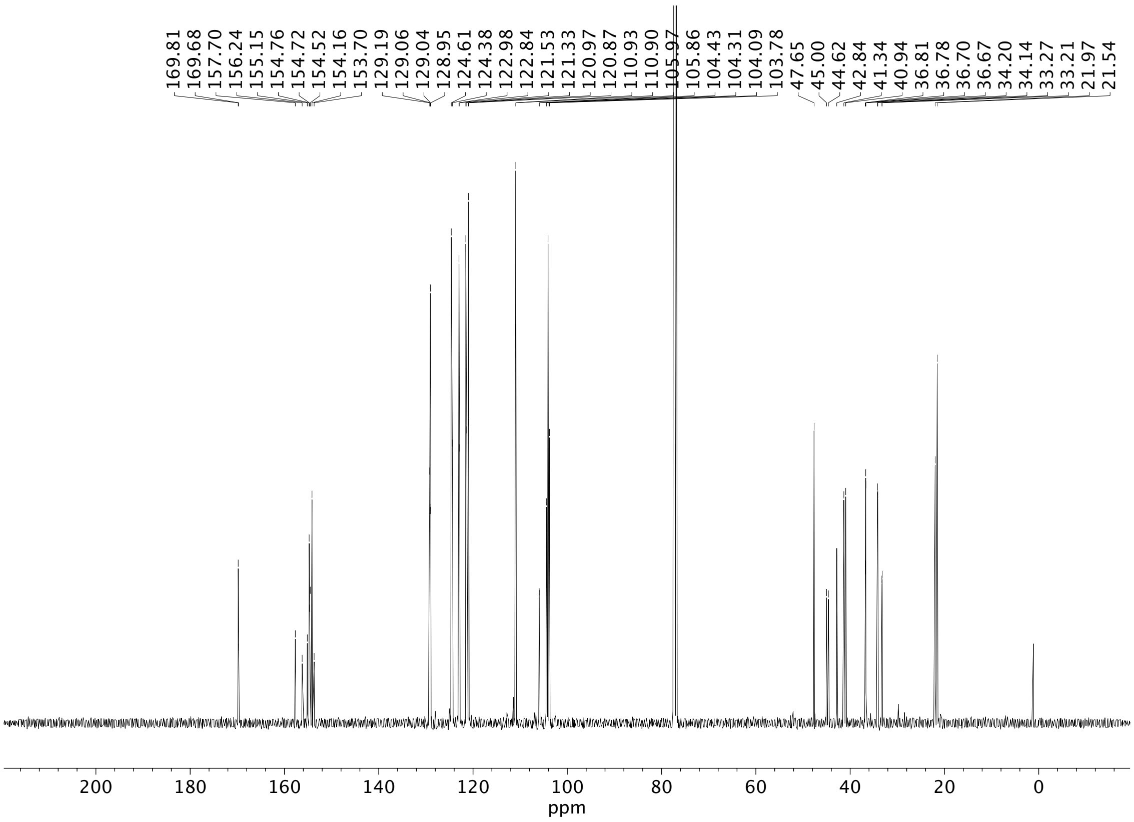

^{13}C NMR (100 MHz, $CDCl_3$) of compound **6w**.

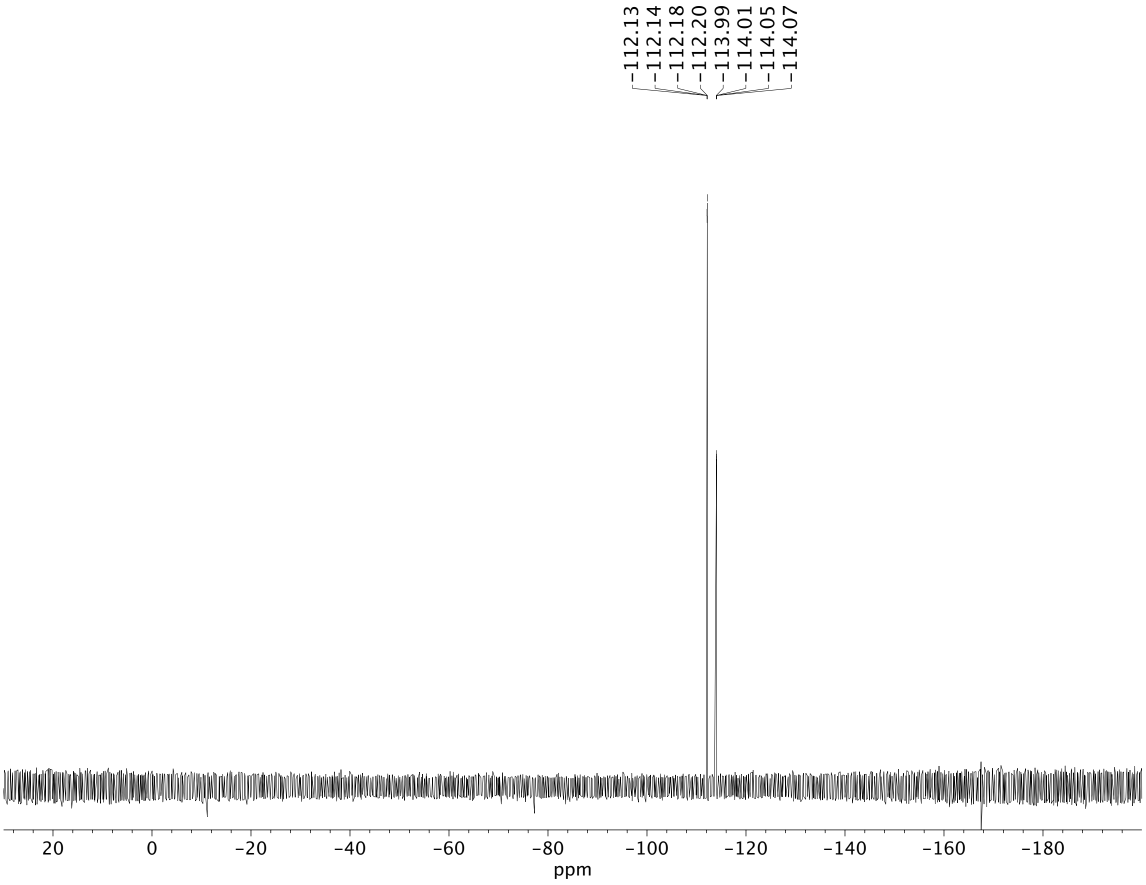

^{19}F NMR (282 MHz, $CDCl_3$) of compound **6w.**

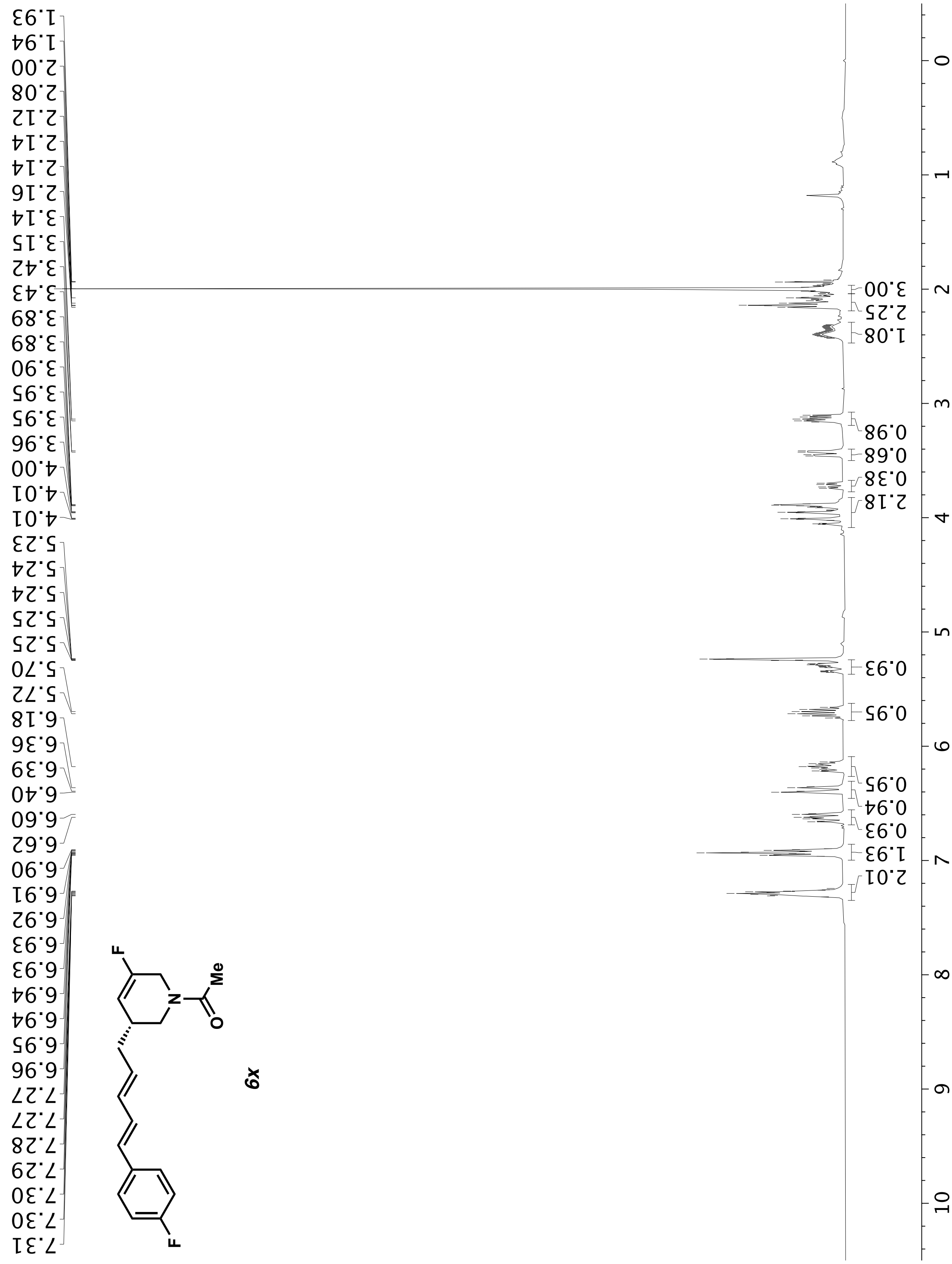

^{1}H NMR (400 MHz, CD_2Cl_2) of compound **6x**

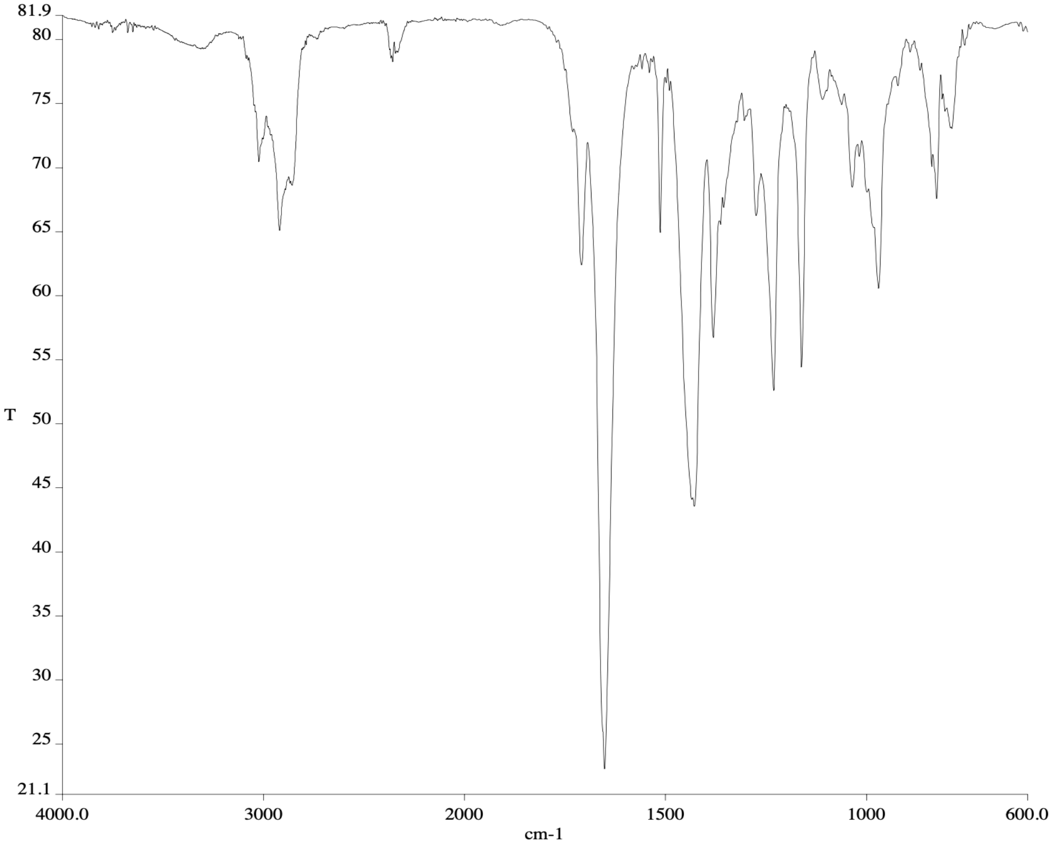

Infrared spectrum (Thin Film, NaCl) of compound **6x**.

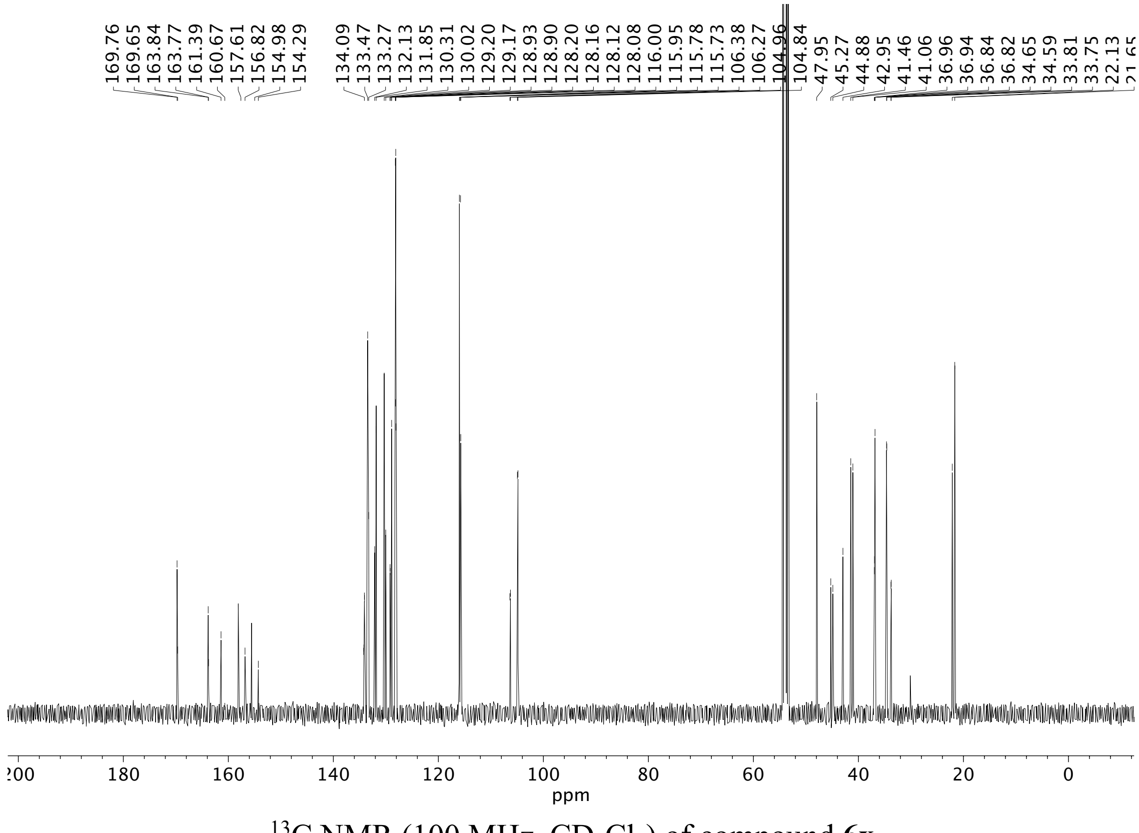

^{13}C NMR (100 MHz, CD_2Cl_2) of compound **6x**.

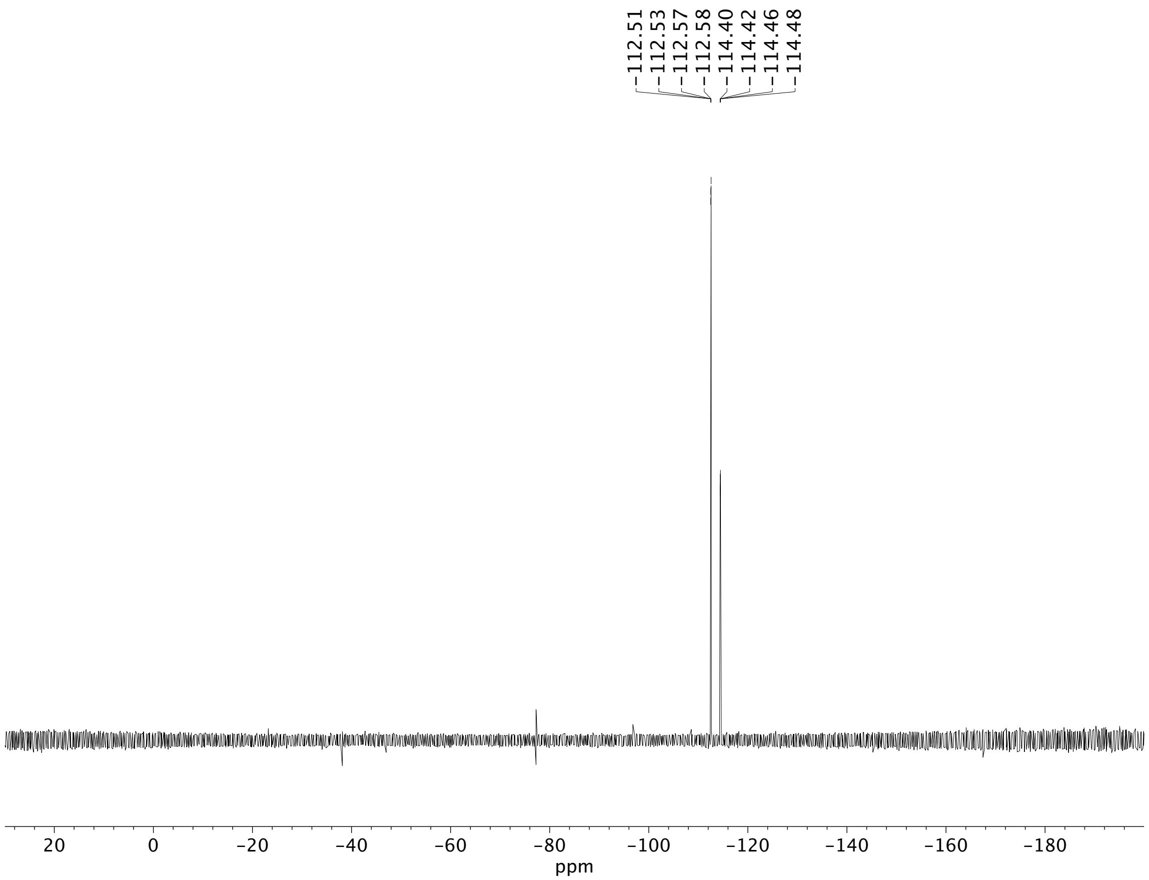

^{19}F NMR (282 MHz, CD_2Cl_2) of compound **6x**.

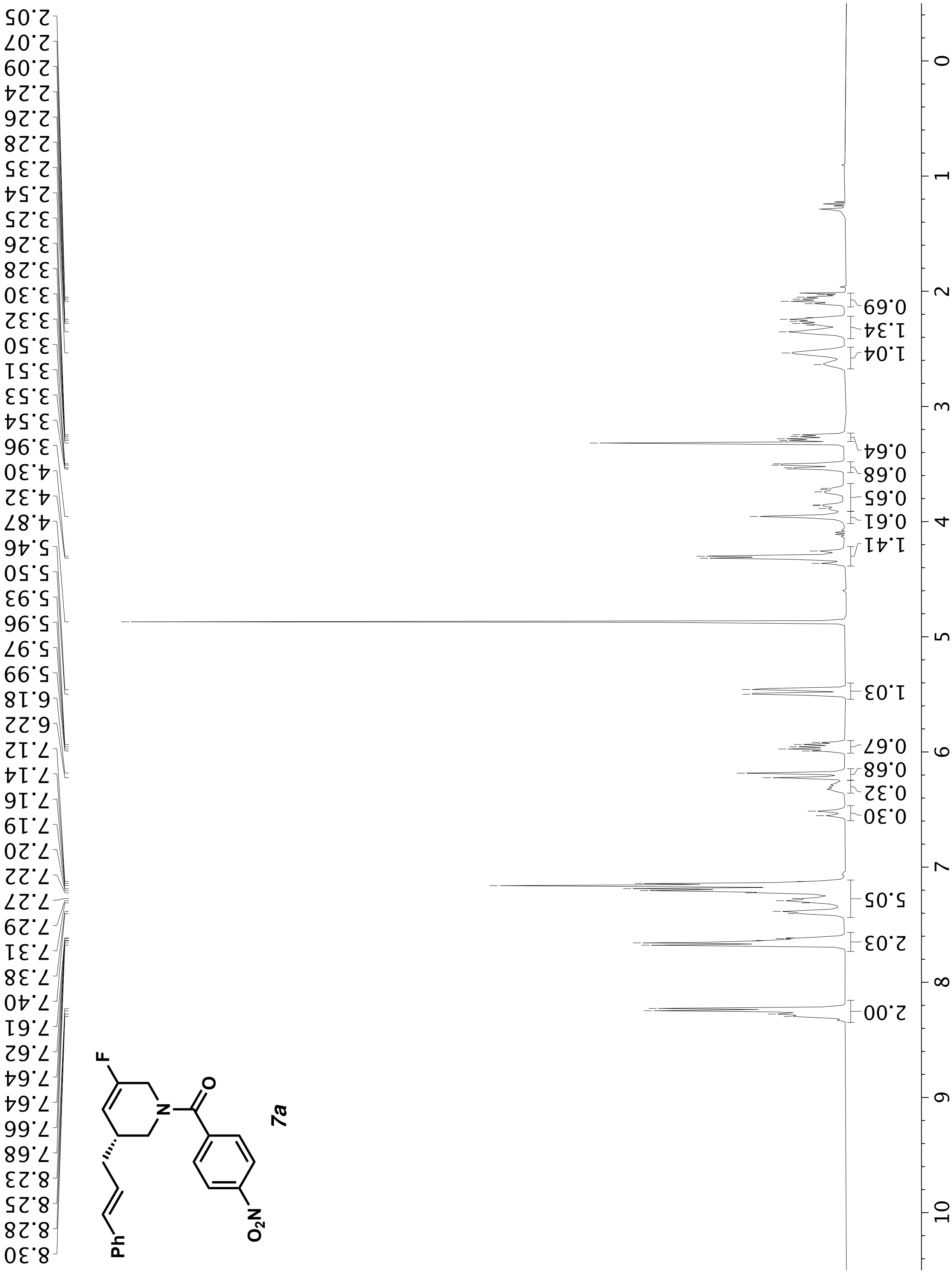

^{1}H NMR (500 MHz, CD_3OD) of compound **7a**.

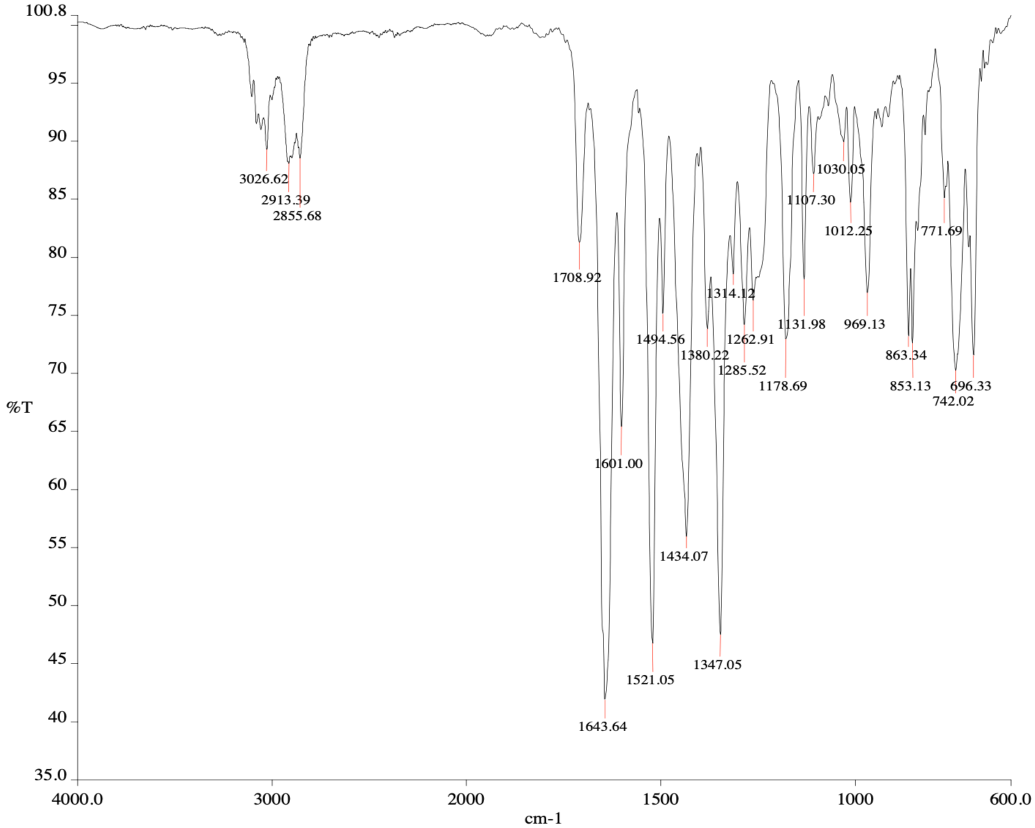

Infrared spectrum (Thin Film, NaCl) of compound **7a**.

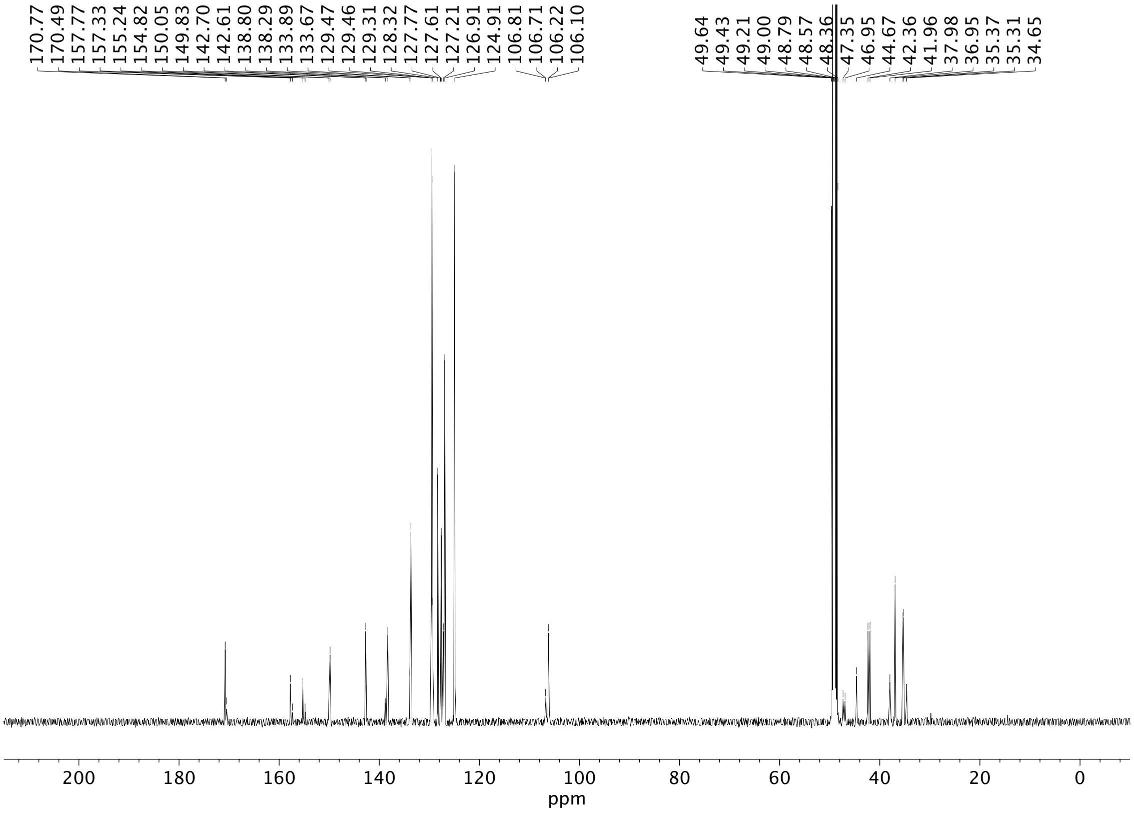

^{13}C NMR (100 MHz, CD_3OD) of compound **7a**.

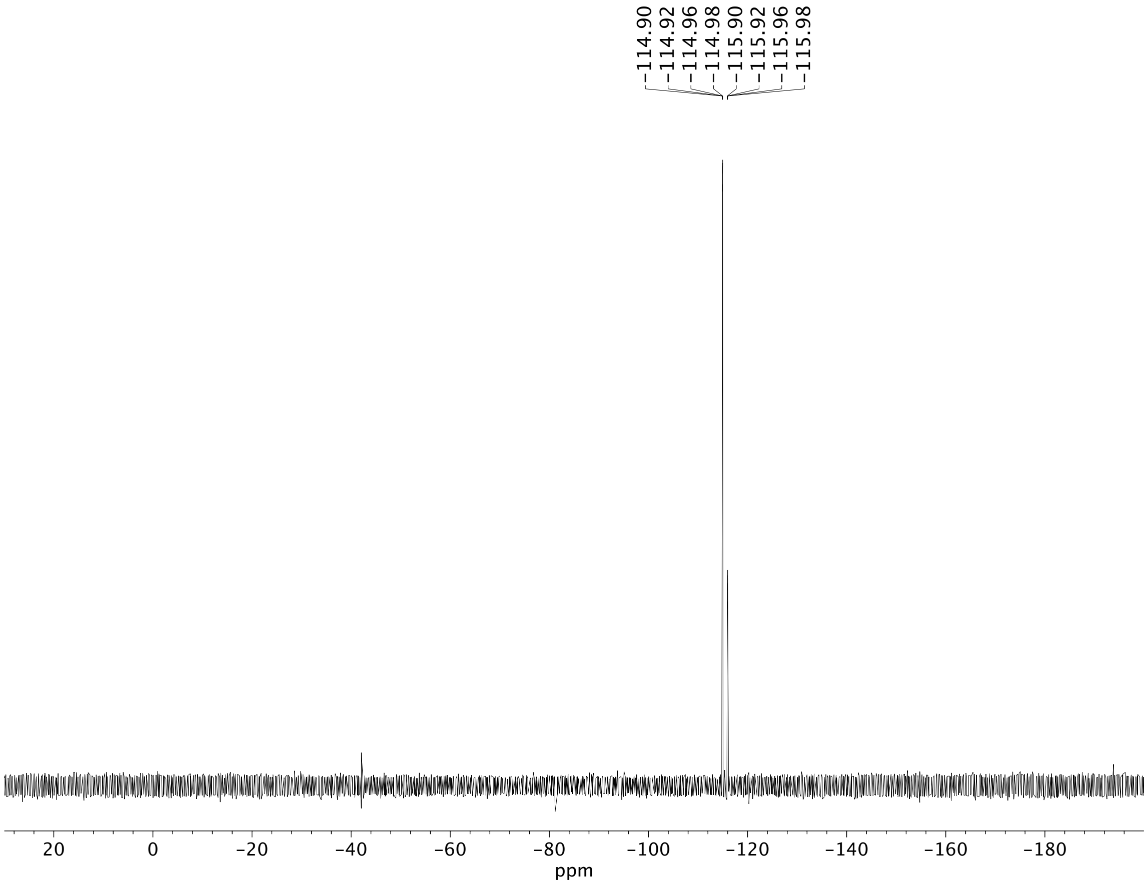

^{19}F NMR (282 MHz, CD_3OD) of compound **7a**.

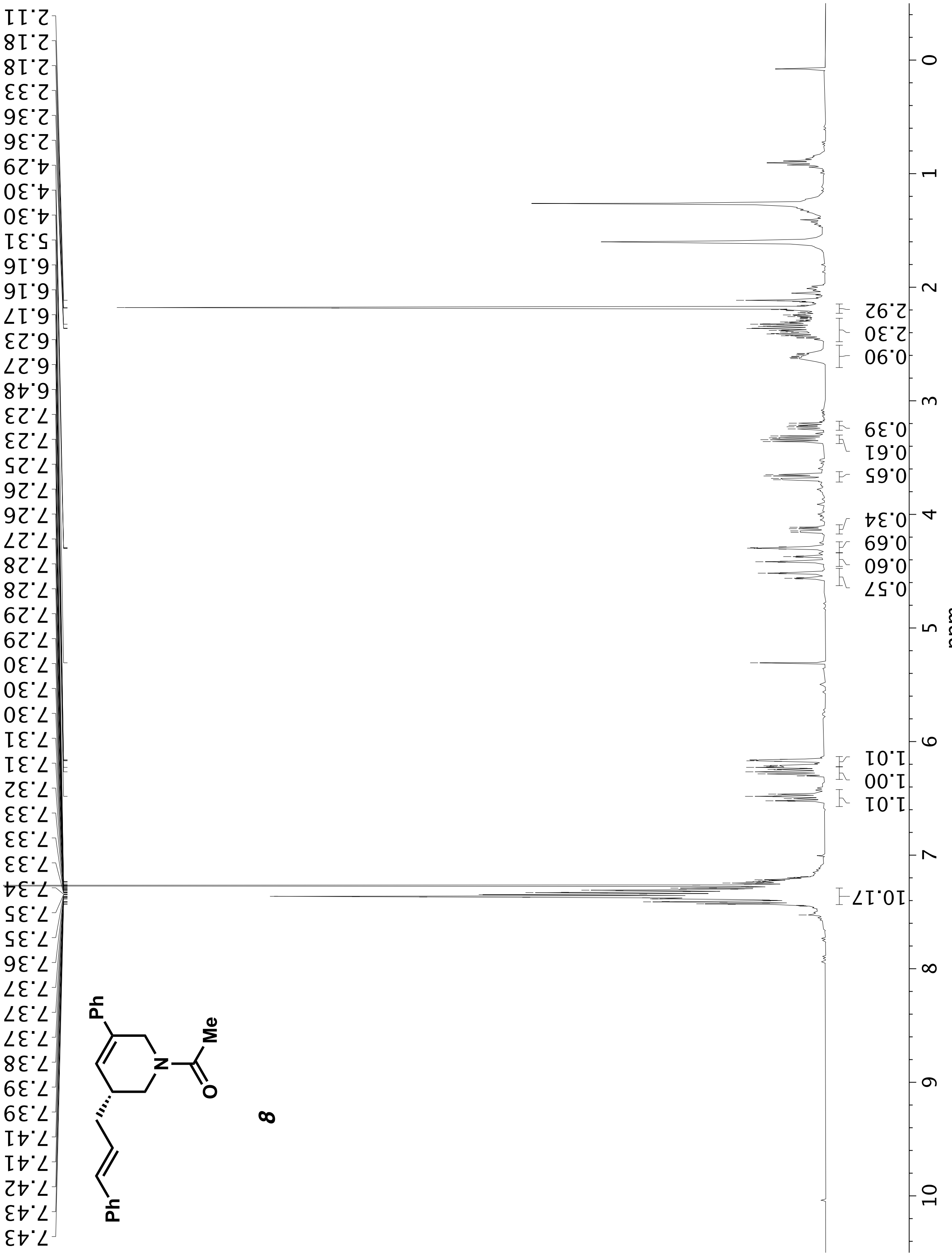

^{1}H NMR (400 MHz, $CDCl_3$) of compound **8**.

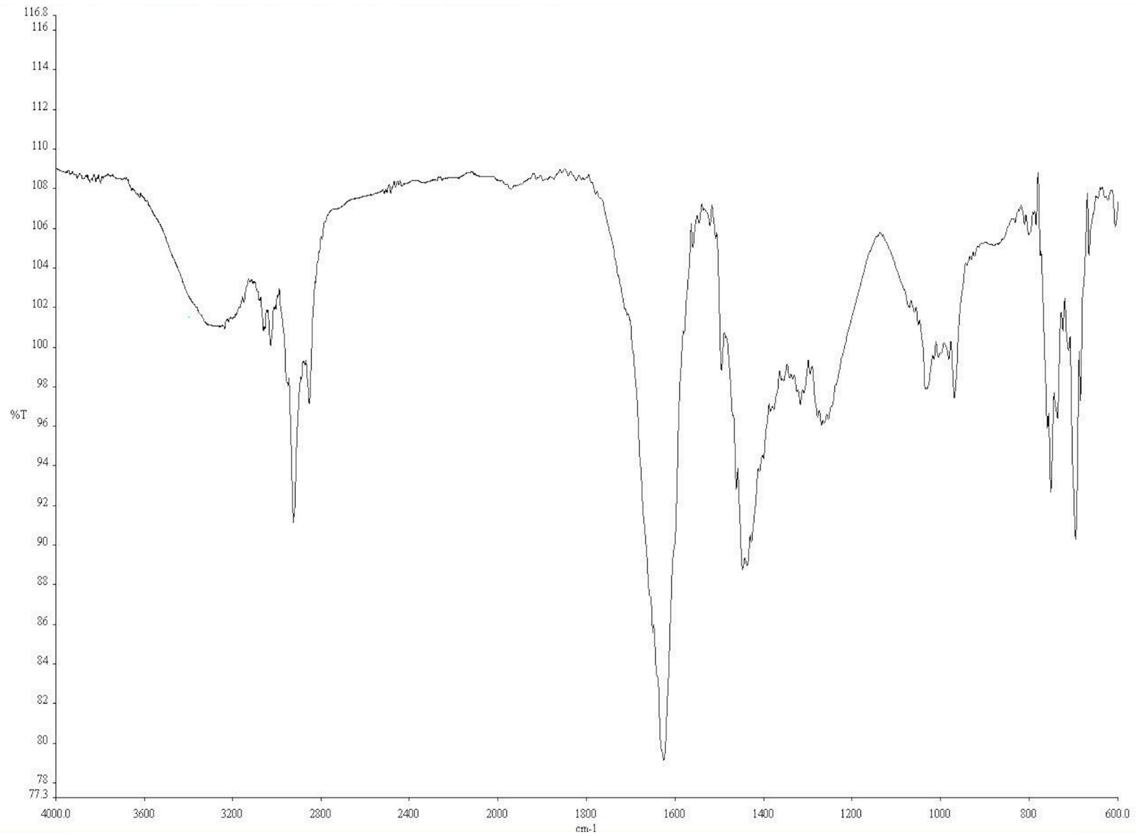

Infrared spectrum (Thin Film, NaCl) of compound **8**.

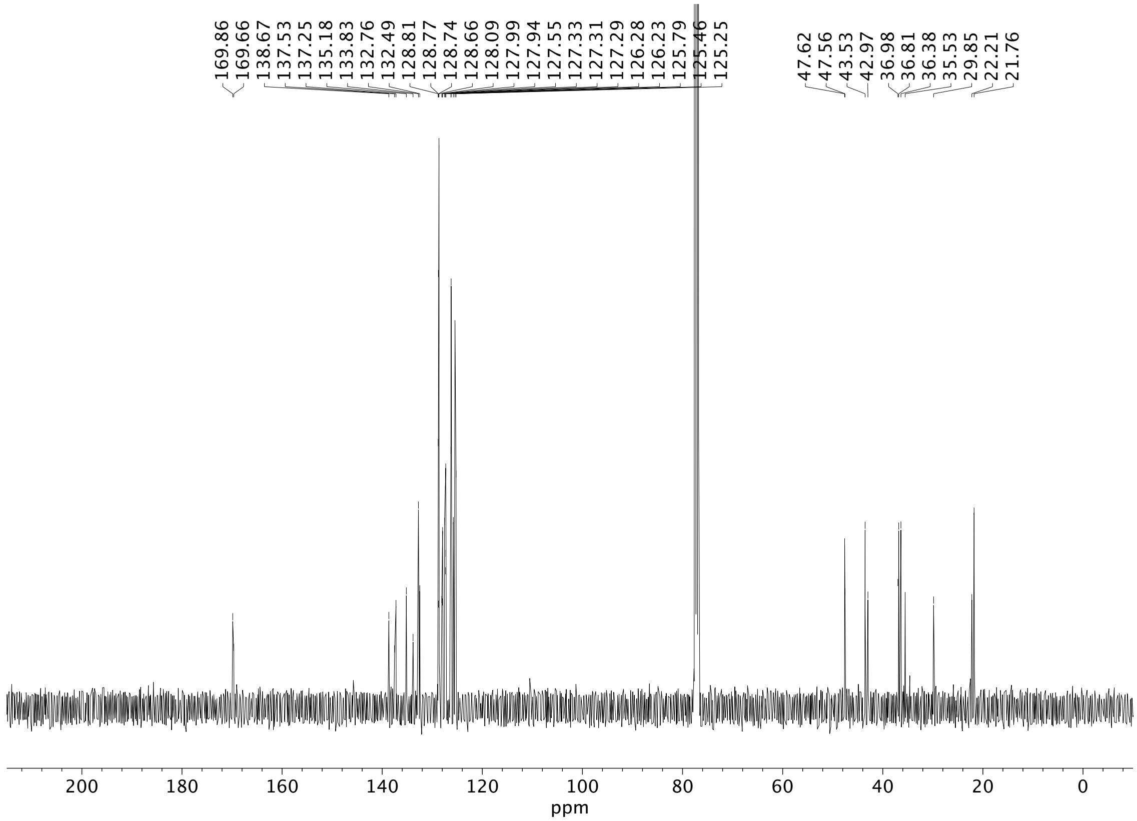

^{13}C NMR (100 MHz, $CDCl_3$) of compound **8**.

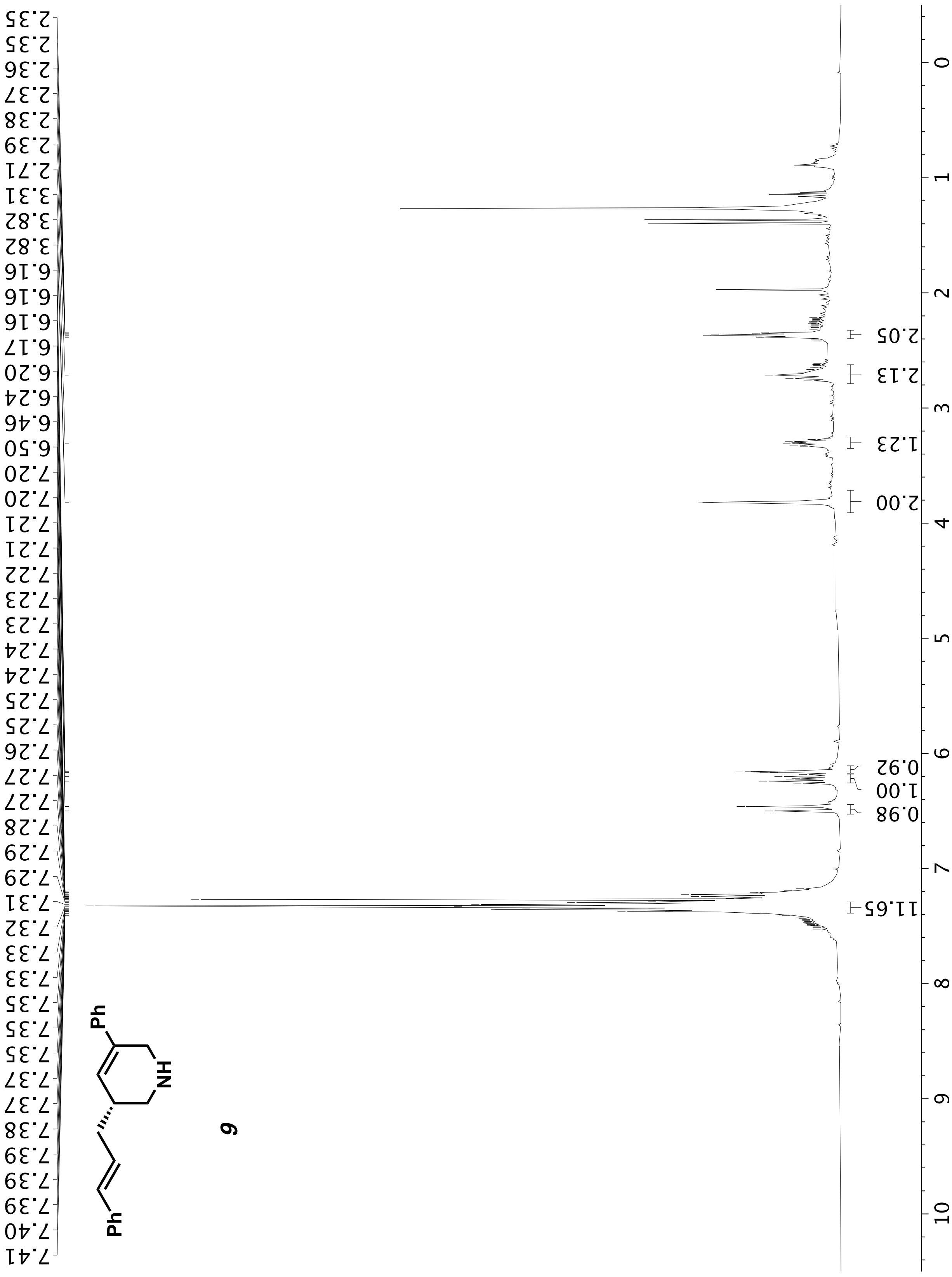

^1H NMR (400 MHz, $CDCl_3$) of compound **9**.

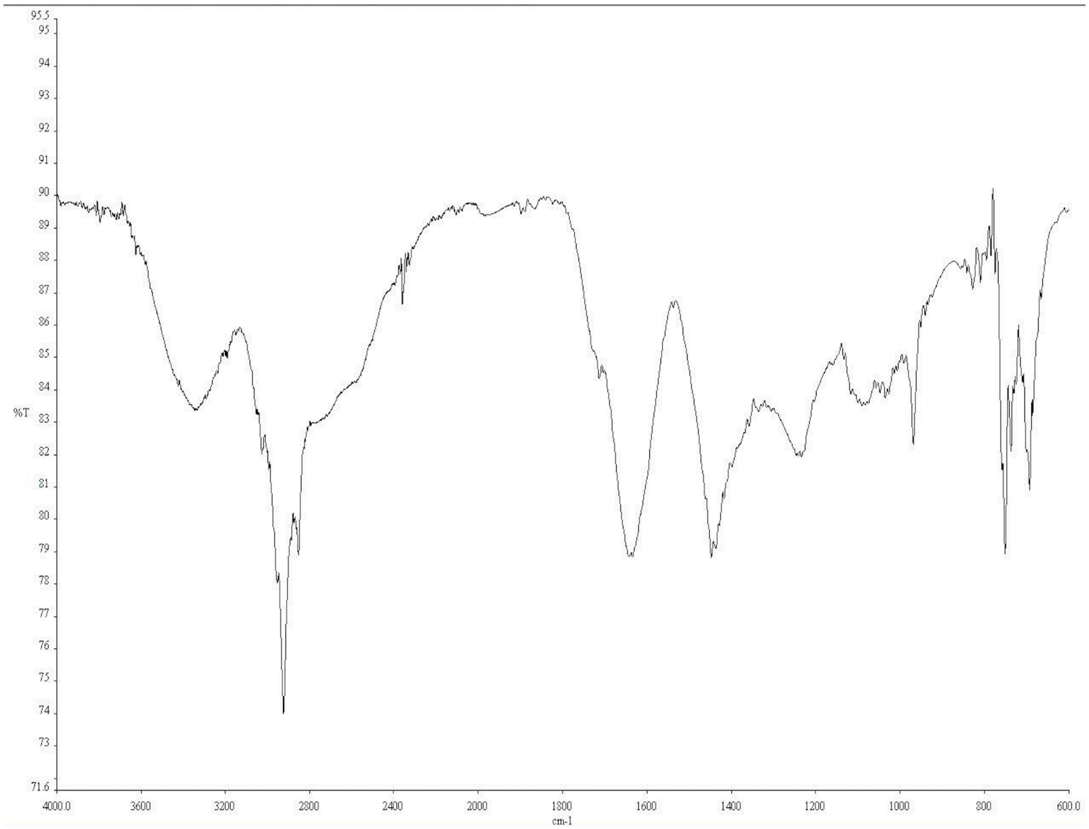

Infrared spectrum (Thin Film, NaCl) of compound **9**.

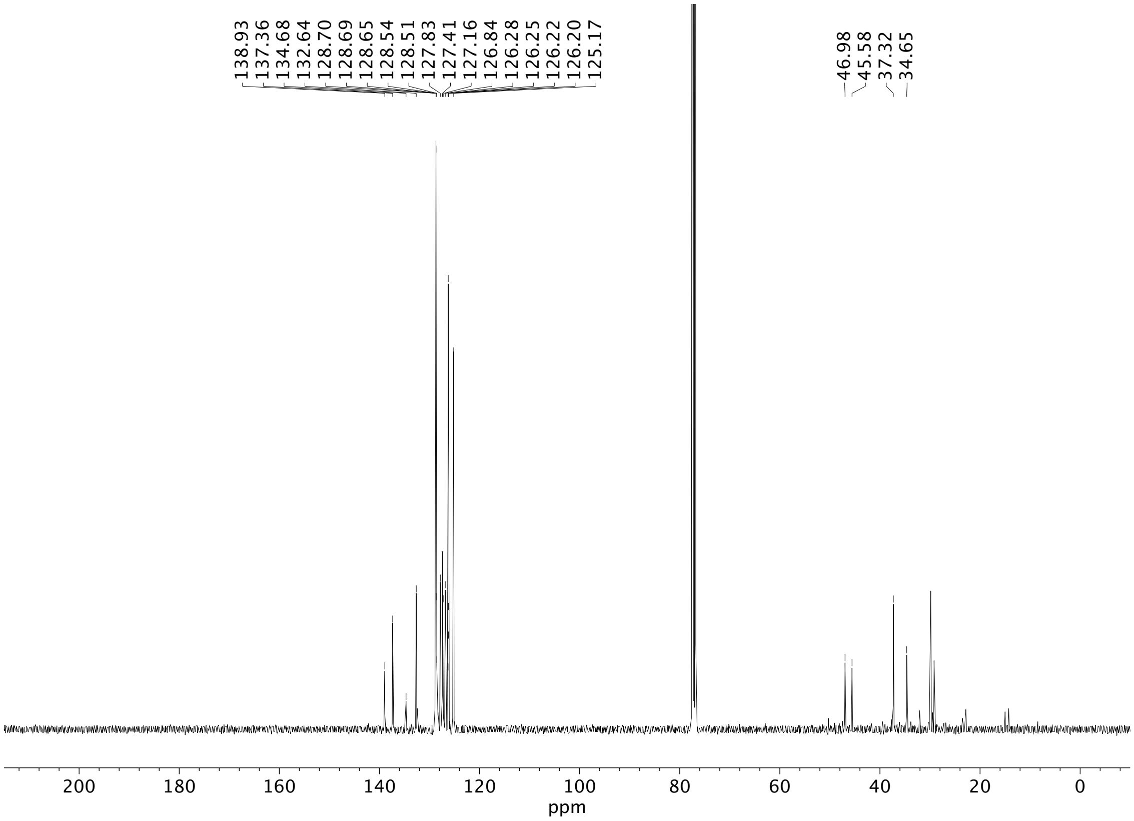

^{13}C NMR (100 MHz, $CDCl_3$) of compound **9**.

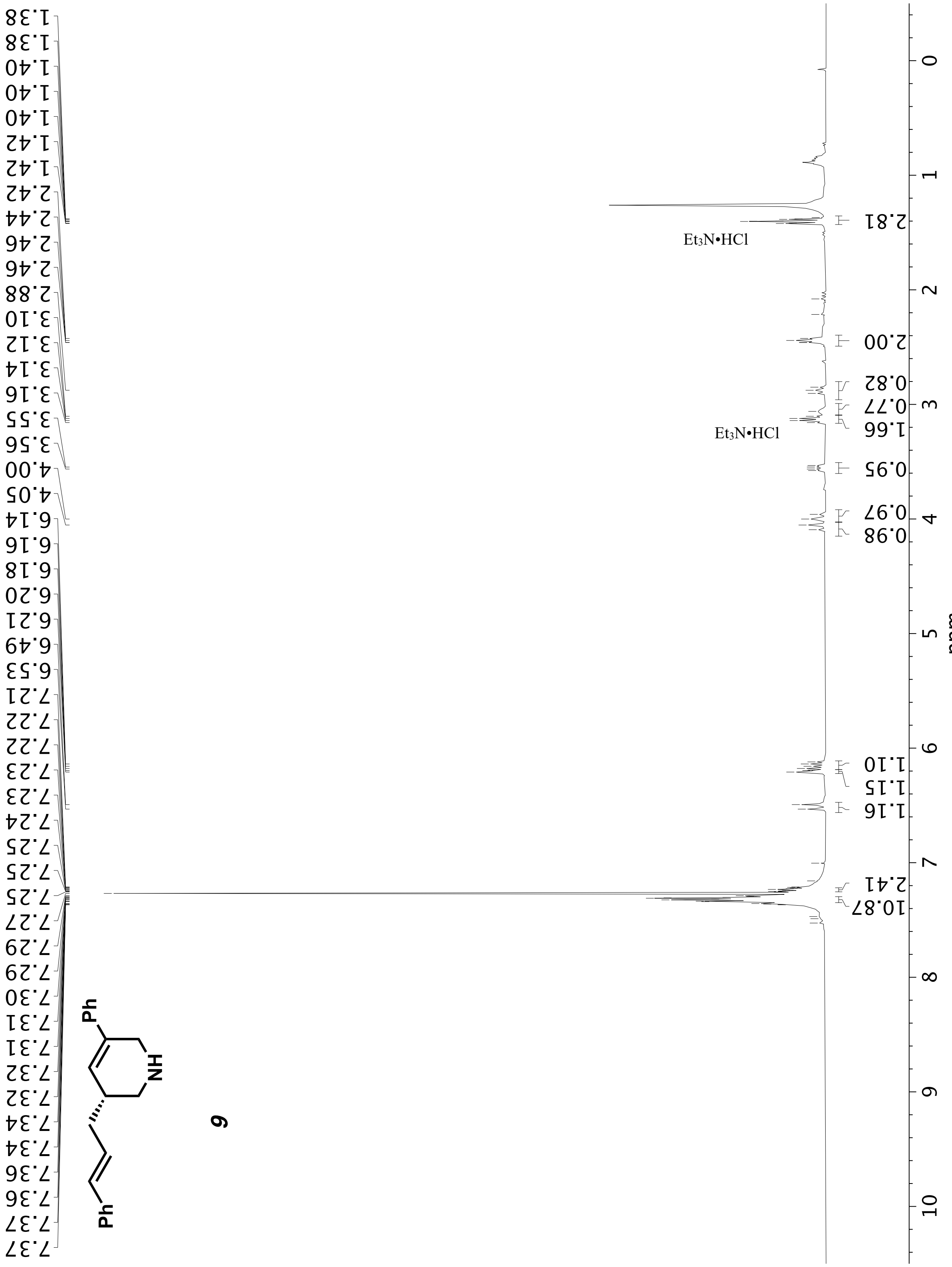

^{1}H NMR (400 MHz, $CDCl_3$) of compound **9**.

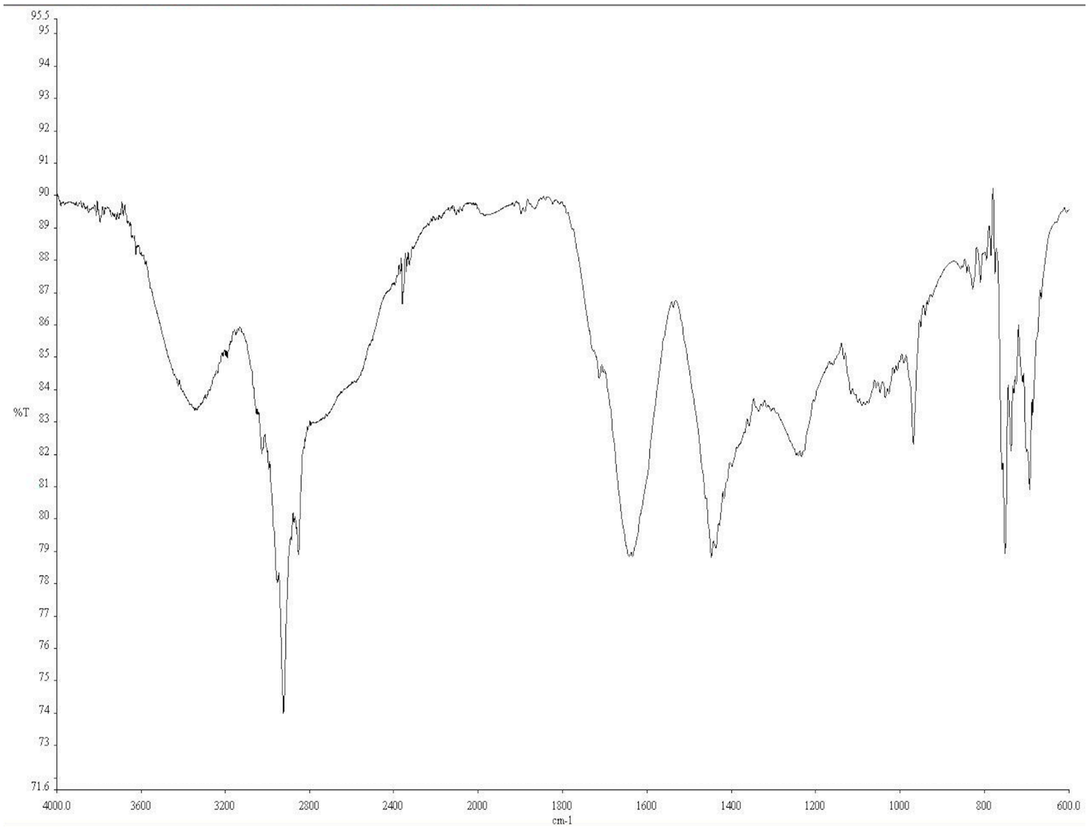

Infrared spectrum (Thin Film, NaCl) of compound **9**.

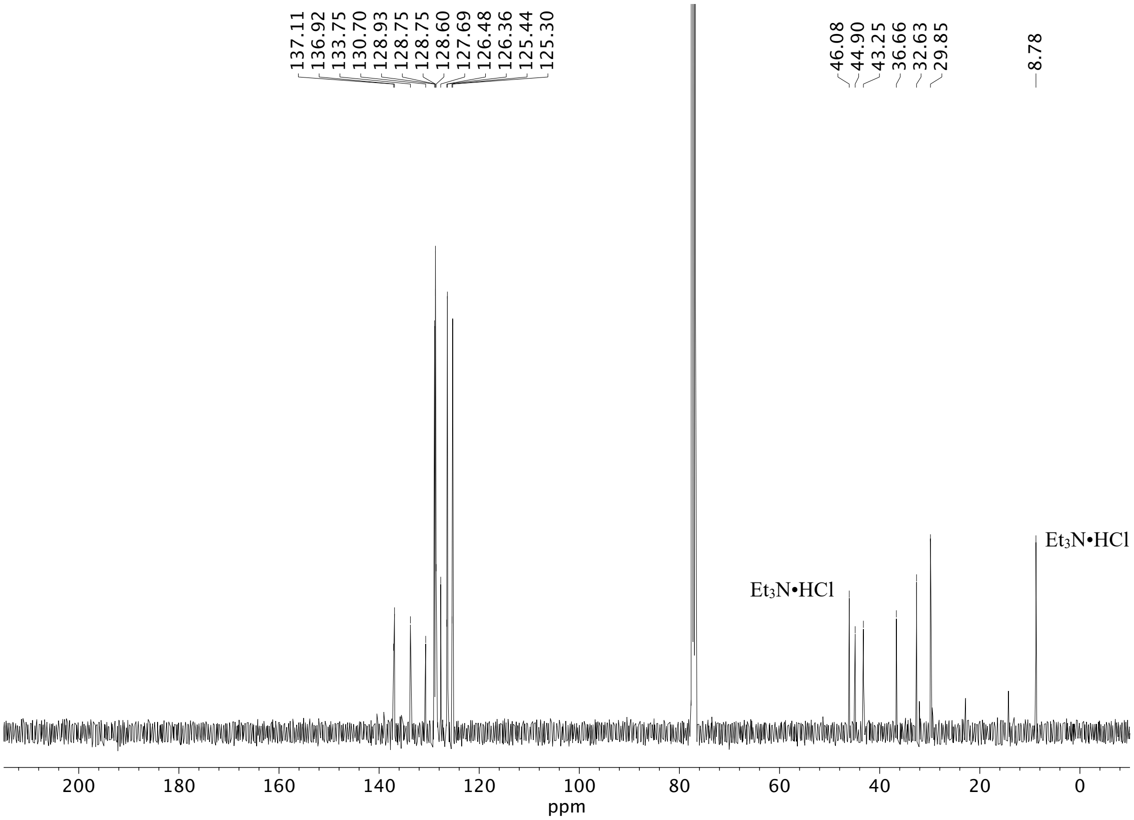

^{13}C NMR (100 MHz, $CDCl_3$) of compound **9**.

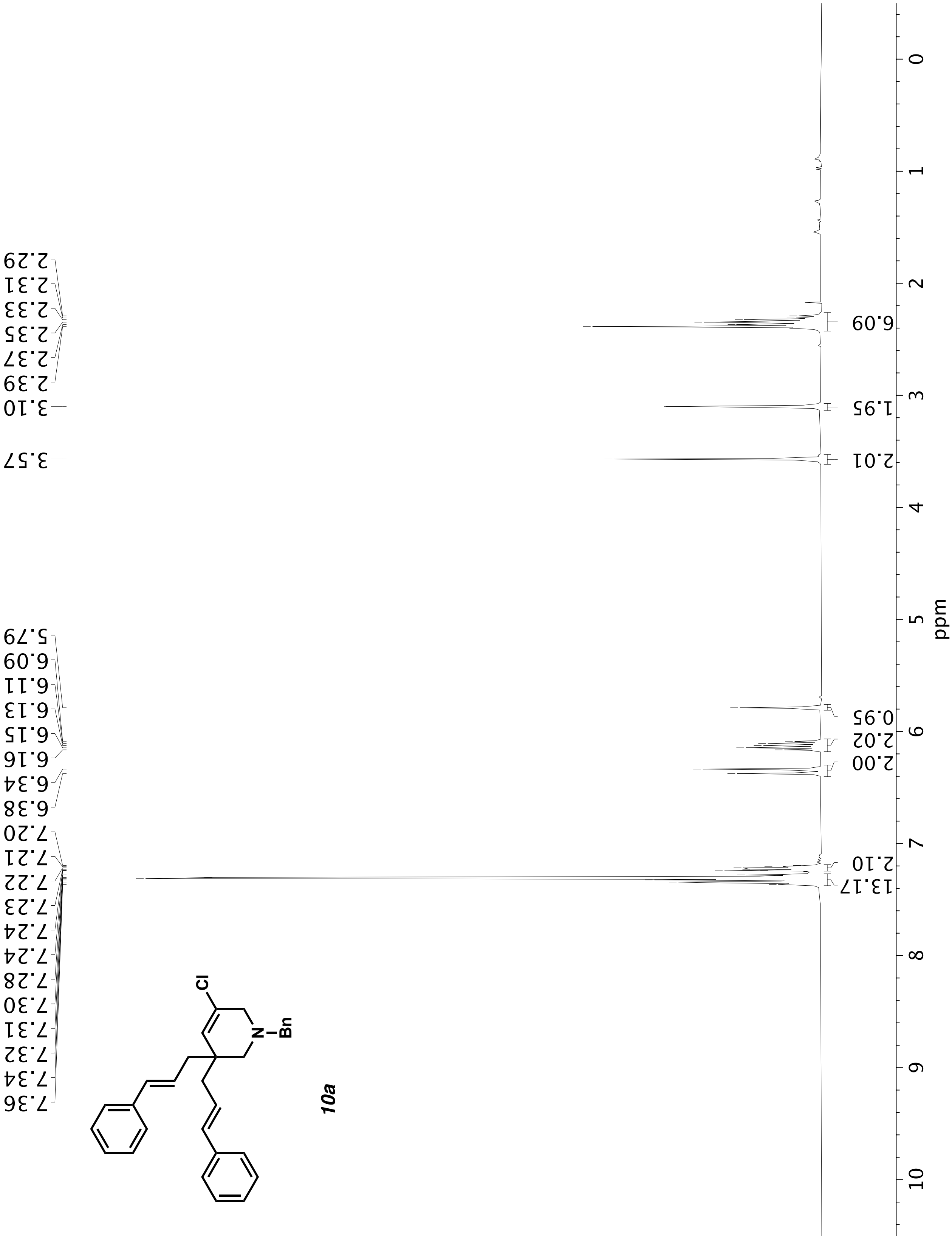

^{1}H NMR (400 MHz, $CDCl_3$) of compound **10a**.

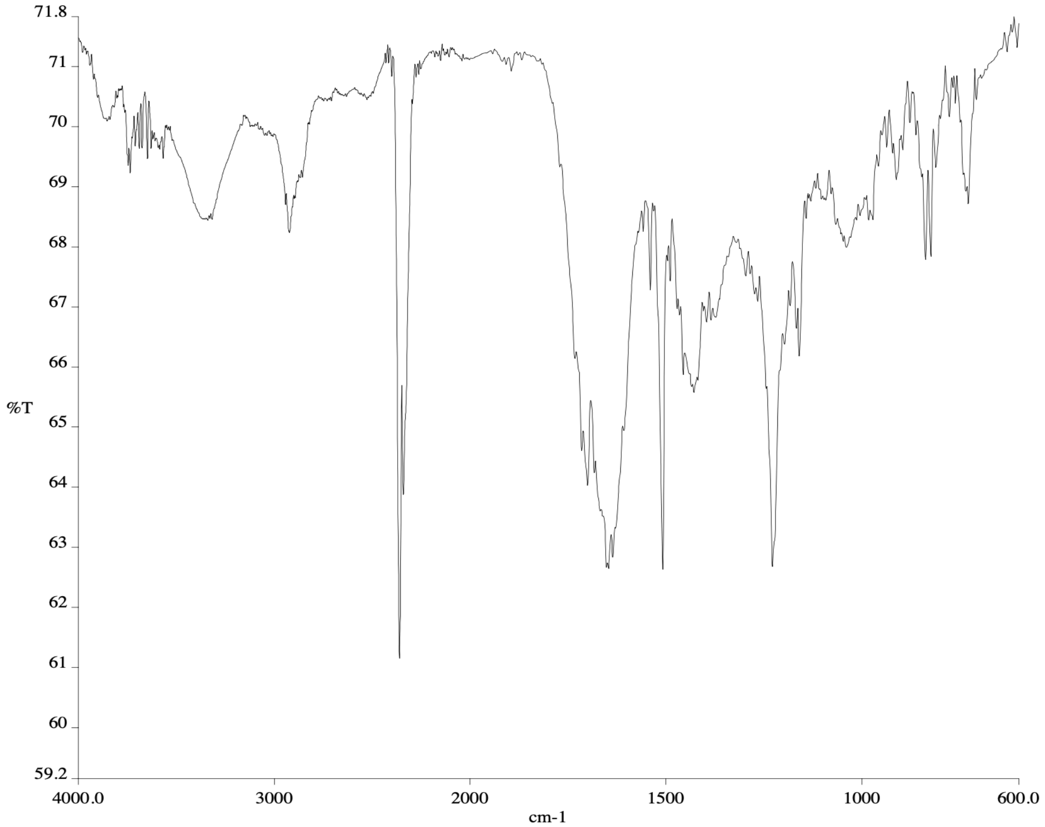

Infrared spectrum (Thin Film, NaCl) of compound **10a**.

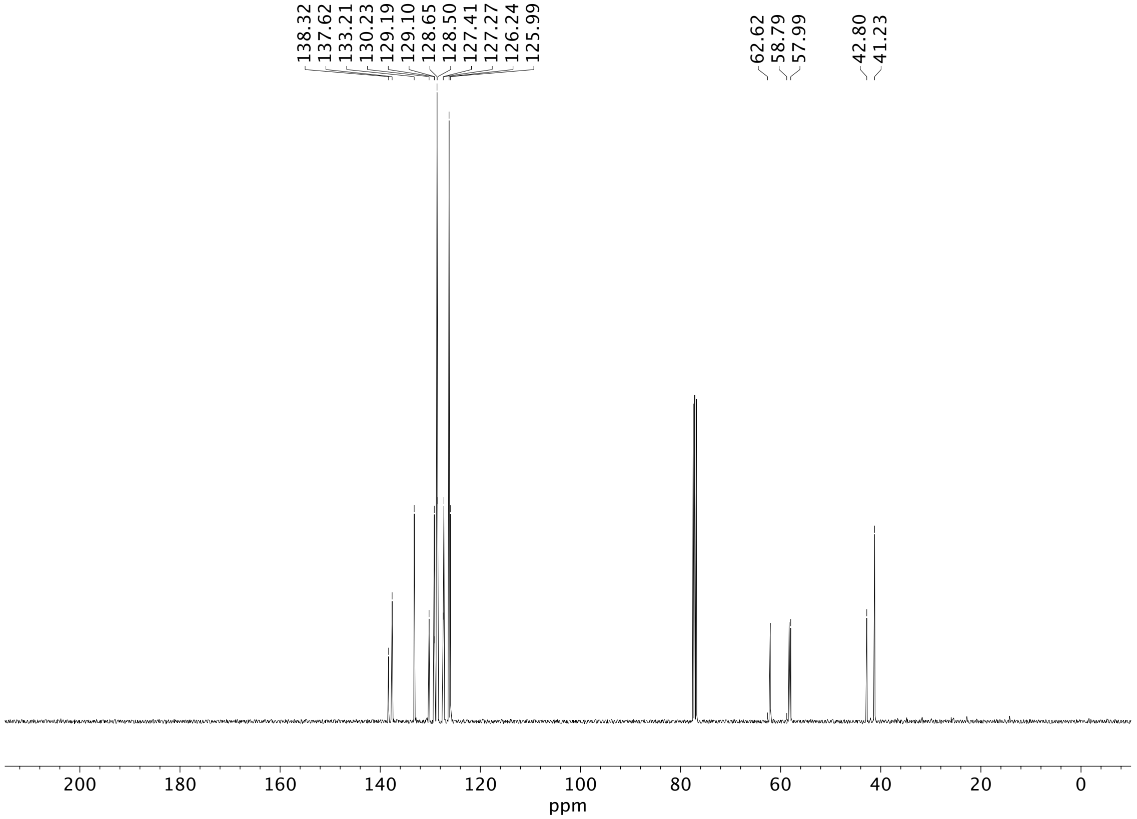

^{13}C NMR (100 MHz, $CDCl_3$) of compound **10a**.

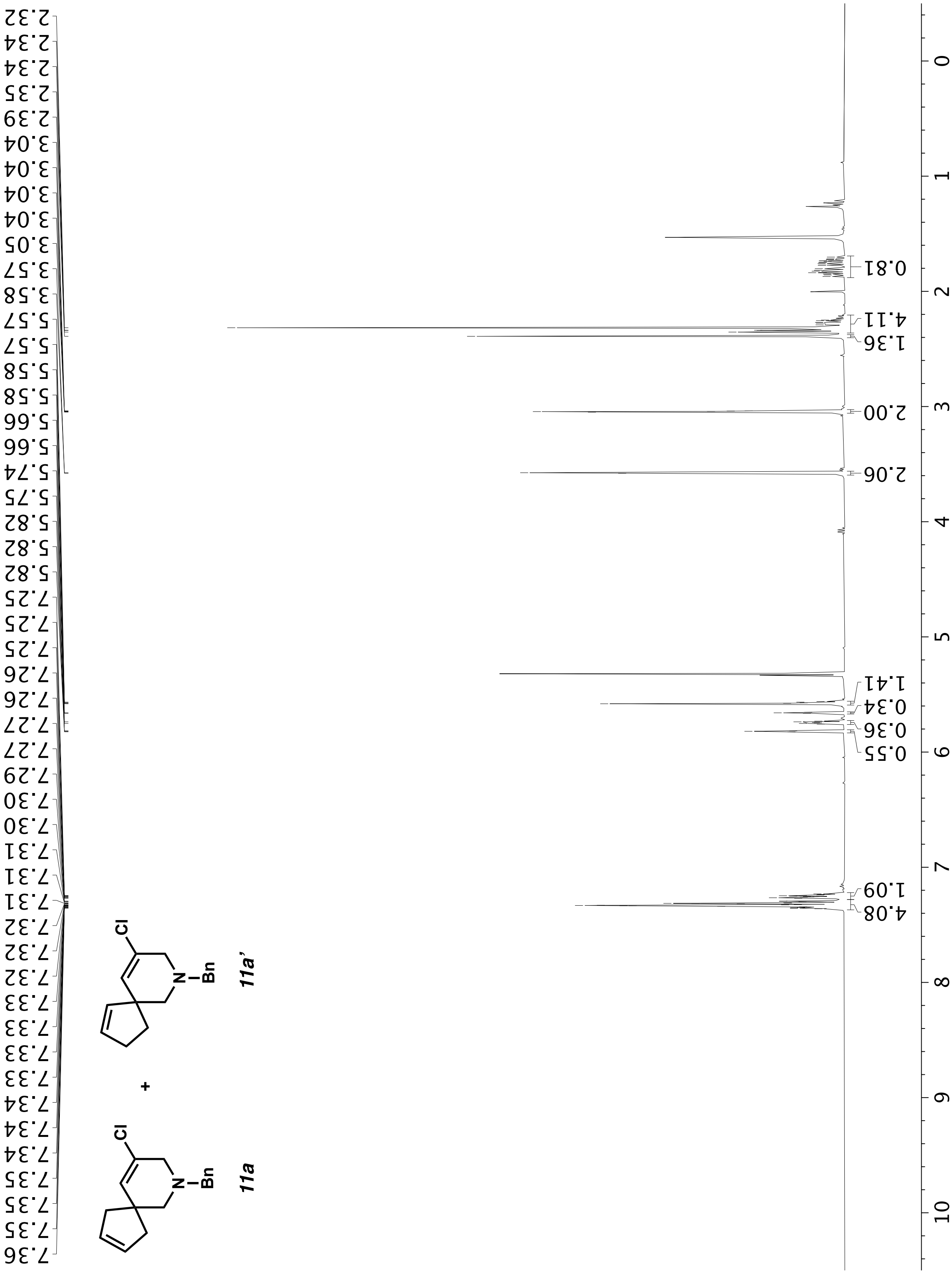

^{1}H NMR (400 MHz, $CDCl_3$) of compound **11a** and **11a'**

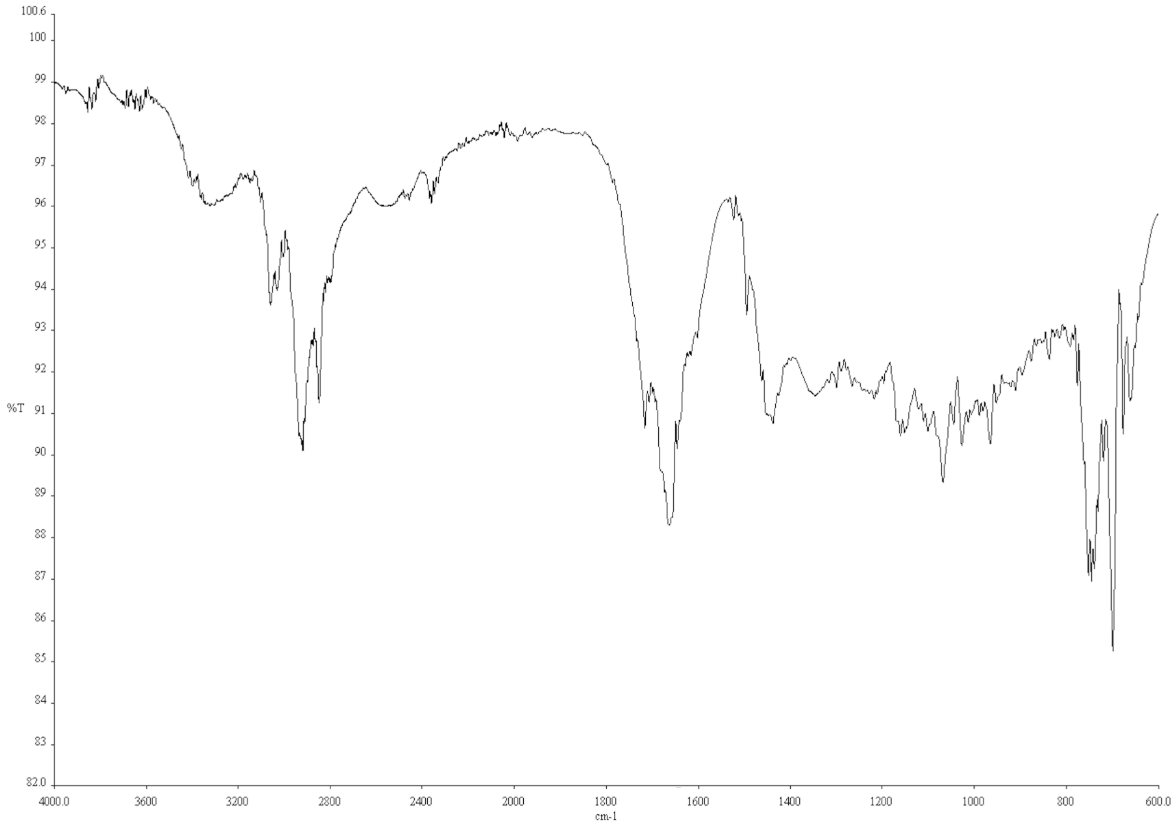

Infrared spectrum (Thin Film, NaCl) of compound **11a** and **11a’**.

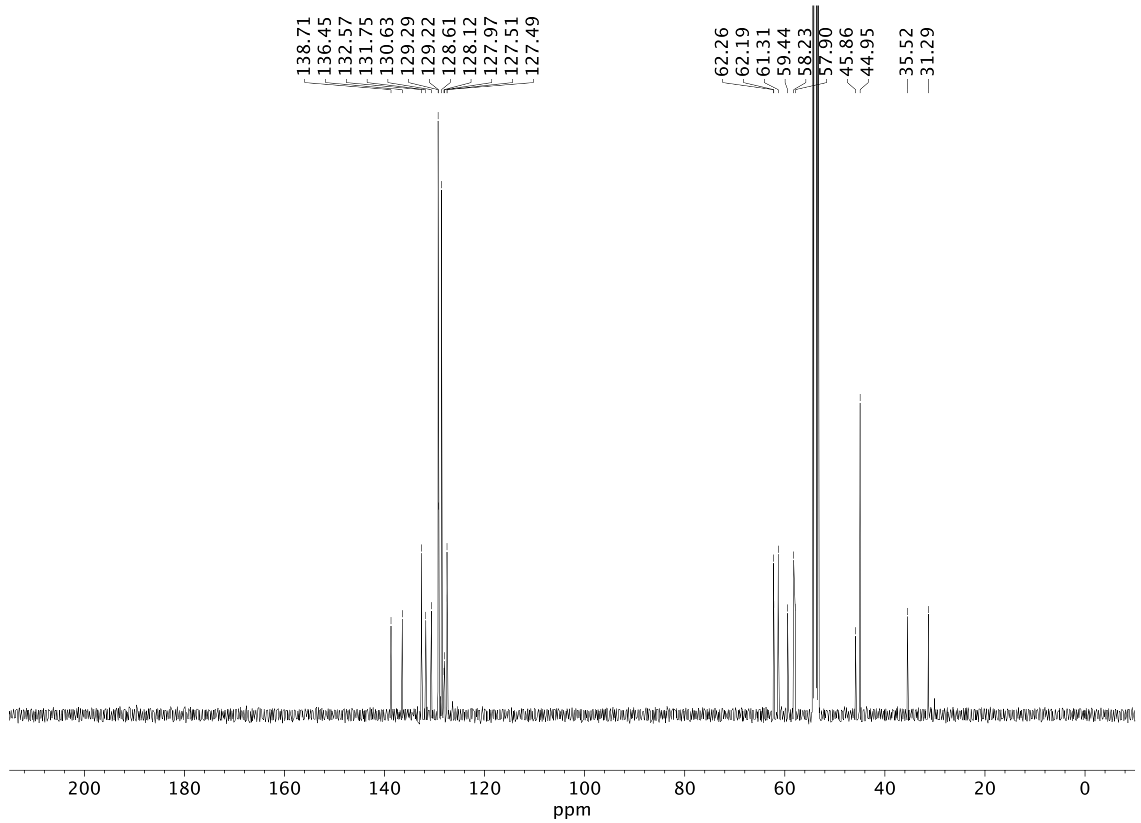

^{13}C NMR (100 MHz, $CDCl_3$) of compound **11a** and **11a’**.
